# Additions to *N*‐Sulfinylamines as an Approach for the Metal‐free Synthesis of Sulfonimidamides: *O*‐Benzotriazolyl Sulfonimidates as Activated Intermediates

**DOI:** 10.1002/anie.201911075

**Published:** 2019-11-11

**Authors:** Maximilian Bremerich, Christian M. Conrads, Tim Langletz, Carsten Bolm

**Affiliations:** ^1^ Institut für Organische Chemie RWTH Aachen University Landoltweg 1 52074 Aachen Germany

**Keywords:** diazonium salts, *N*-hydroxybenzotriazoles, *N*-tritylsulfinylamines, sulfonimidamides, sulfonimidates

## Abstract

Sulfonimidamides are obtained in moderate to very good yields from the key intermediates *O*‐benzotriazolyl sulfonimidates, which are formed by reacting aryldiazonium tetrafluoroborates, *N*‐tritylsulfinylamine, and *N*‐hydroxybenzotriazole hydrate in a process mediated by a tertiary amine. The formation of the sulfonimidate proceeds in inexpensive and environmentally benign dimethyl carbonate as the solvent, it does not require anhydrous conditions, and the product yields generally exceed 70 %. The substrate scope is broad, and a wide range of sensitive organic functionalities is well tolerated. The reactions probably proceed via aryl radicals formed from diazonium cations with assistance from both the tertiary amine and the sulfinylamine.

## Graphical abstract

***N*** **‐Tritylsulfinylamine,** aryldiazonium tetrafluoroborates, and *N*‐hydroxybenzotriazole hydrate react to give *O*‐benzotriazolyl sulfonimidates, which can be converted into sulfonimidamides in good yields. The two processes are mild and enable the preparation of a wide range of functionalized products. The synthetic utility was demonstrated by the syntheses of a novel azasaccharine derivate and a sulfonimidamide analogue of sildenafil.

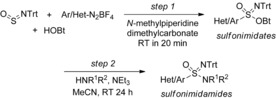

## Introduction

**Scheme 1:**
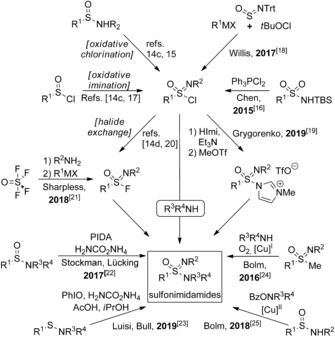
Overview of approaches towards sulfonimidamides (Trt=trityl).

Sulfonamides are among the most abundant and important structural motifs in medicinal and agricultural chemistry. They have a rich history in medical circles, with sulfonamides being one of the first commercial antibiotics ever available, for example.^1^ Furthermore, sulfonamide derivatives have exhibited high biological activities, including antiproliferal,^2^ diuretic,^3^ antihypertensive,^4^ hypoglycemic,^5^ antiinflammatory,^6^, ^7^ antiviral,^8^ and herbicidal properties.^9^ A recent report states the prevalence of the sulfamoyl group in sulfur‐containing drugs to be as high as 29 %.^10^ Among the various sulfonamide derivatives, their monoaza analogues, known as sulfonimidamides, have recently attracted increasing attention, in particular, because they are regarded as bioisosters of the parent compounds.^11^, ^12^ However, to date, only relatively few methods for the preparation of sulfonimidamides have been reported, with most of them still having severe synthetic disadvantages that prevent easy access to a wide variety of such attractive compounds.^13^ Most sulfonimidamide syntheses proceed via sulfoximinoyl chlorides as reactive intermediates (Scheme 1), which are commonly not isolated because of their sensitivity to hydrolysis and redox side reactions.^14^ In these methods, key steps are oxidative chlorinations14c, 15, 16 and/or iminations14c, 17 of lower‐valent sulfur compounds, and subsequent nucleophilic substitutions of the resulting sulfoximinoyl chlorides. However, the employment of strong oxidation or halogenation agents is often incompatible with sensitive functionalities and poses a risk in itself. In part, this is also true for the redox‐neutral approach introduced by Chen and Gibson, who used triphenylphosphine dichloride for the deoxychlorination of sulfonamides.^16^ Functional‐group sensitivity is also an issue when highly basic and nucleophilic organometallic reagents are applied, as demonstrated by Willis and co‐workers,^18^ because they not only require strictly anhydrous and/or otherwise inert conditions, but also do not tolerate important functional groups such as the carbonyl groups of esters, ketones and aldehydes. Grygorenko and co‐workers fine‐tuned the stability of the reactive intermediates by converting the sulfoximinoyl chlorides to the corresponding *N*‐methylimidazolium triflates.^19^ The same can be achieved by using their relatively stable fluorine‐containing counterparts. While the preparation of the latter from sulfoximinoyl chlorides by halide exchange has long been known,14d, 20 they can now directly be accessed by Sharpless’ SuFEx approach.^21^ Unfortunately, the practicability of this process is limited by the use of thionyl tetrafluoride (SOF_4_), which is a toxic and corrosive gas. Stockmann, Lücking, and co‐workers were able to overcome many of the aforementioned challenges by directly converting sulfinamides into sulfonimidamides through oxidative imination with in situ generated iminoiodinane(III) species.^22^ Recently, this chemistry was extended by Luis, Bull, and co‐workers, who applied sulfenamides as starting materials.^23^ Two very different approaches circumventing the formation of hydrolytically sensitive intermediates were introduced by us, both through the use of copper catalysis. The first one involved oxidative dealkylations of *N*H‐*S*‐aryl‐*S*‐alkyl sulfoximines in the presence of secondary amines, which led to tertiary *N*H‐sulfonimidamides, presumably via *S*‐centered sulfoximinoyl radicals.^24^ In the second, fully substituted sulfonimidamides were obtained by reacting *S*‐arylsulfinamides with *O*‐benzoyl hydroxylamine derivatives.^25^ Due to the limits of the current methods, we were interested in devising a new approach towards sulfonimidamides that is flexible and shows high functional‐group tolerance.

## Results and Discussion

**Scheme 2:**
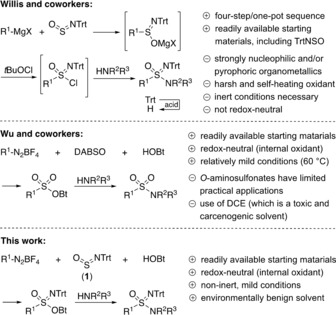
Approach towards sulfonimidamides reported by Willis and co‐workers (top), syntheses of *O*‐aminosulfonates and sulfonamides described by Wu and co‐workers (middle), and the fundamentals of the reaction sequence reported here (bottom).

In 2017, Willis and co‐workers reported groundbreaking findings on a one‐pot‐four‐step reaction sequence (Scheme 2, top)^18^, ^26^ that enables the preparation of sulfonimidamides through the addition of (hetero)aryl‐ or alkylmagnesium halides to the hydrolytically stable and easily accessible reagent *N*‐tritylsulfinylamine (**1**), followed by oxidation of the resulting sulfinimidate salts with *tert*‐butylhypochlorite to give the corresponding sulfoximinoyl chlorides. Subsequent treatment of the latter products with primary and secondary amines yielded *N*‐tritylsulfonimidamides, which were deprotected with strong acids to give *S*‐aryl and *S*‐alkyl‐*N*H‐sulfonimidamides in high yields. The redox cascade, in which the oxidation number of sulfur changed from +IV to +II back to +IV, is particularly conspicuous here. We were also interested by a report by Wu and co‐workers (Scheme 2, middle),^27^ who described the formation of sulfonamides via *O*‐aminosulfonates, which were obtained from diazonium tetrafluoroborates, the solid sulfur dioxide surrogate DABSO (DABCO⋅2SO_2_), and *N*,*N*‐disubstituted hydroxylamines, including 1‐hydroxybenzotriazole (HOBt). The proposed reaction mechanism included DABCO‐assisted extrusion of molecular nitrogen from the diazonium salt to yield an aryl radical and the DABCO radical cation. The former attacks the SO_2_ to form a sulfur‐centered sulfonyl radical, while the latter abstracts a hydrogen atom from the hydroxylamine to form the DABCO cation and an oxygen‐centered hydroxylamine radical. Finally, combination of the two radicals gives the *O*‐aminosulfonates in a remarkably exergonic process. When HOBt was employed as the hydroxylamine, the method enabled the one‐pot formation of sulfonamides by substitution with primary or secondary amines present in the reaction mixture. A similar mechanism was reported for the formation of sulfonamides from aryl diazonium salts, DABSO, sodium azide, and triphenylphosphine.^28^ Considering **1** as a protected monoaza analogue of SO_2_, we wondered about its use for the synthesis of *O*‐aminosulfonimidates and sulfonimidamides (Scheme 2, bottom). The realization of this strategy is reported here.

With the intention to use a representative starting material that could be easily detected and followed by NMR spectroscopy, the study was initiated by applying 4‐fluorophenyldiazonium tetrafluoroborate (**2 a**) as the aryl source. To our delight, **2 a** reacted with equimolar amounts of *N*‐tritylsulfinylamine (**1**) and HOBt hydrate in acetonitrile as hypothesized, affording the targeted *O*‐Bt‐*N*‐Trt‐sulfonimidate **3 a** in 42 % yield. The reaction occurred within seconds, as revealed by the rapid termination of nitrogen evolution. Varying the amount of DABCO (Table 1; with a 5:1 mixture of MeCN and THF as solvent for 20 min at ambient temperature; analysis by ^19^F qNMR spectroscopy) showed that it acts as mediator and that at least one equivalent of this base had to be added to achieve high conversion of **2 a** (>95 %), providing **3 a** in 60 % yield (Table 1, entry 5). With less DABCO (Table 1, entries 1 and 3–4) or with one equiv of CaCO_3_ and a catalytic amount of DABCO (Table 1, entry 6), the product formation was not more efficient.^29^ Performing the reaction at 60 °C yielded *N*‐tritylsulfonamide **3 aa** as the main product (Table 1, entry 6). Other detectable side products were detritylated sulfonamide **3 ab**, *N*‐trityl‐sulfinamide **3 ac,** and *O*‐Bt‐sulfonate **3 ad** (for details, see the Supporting Information).

**Table 1:** Influence of the amount of DABCO on the conversion of diazonium salt **2 a** and the yield of sulfonimidate **3 a**.^[a]^ 
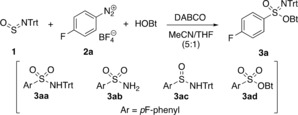

Entry	DABCO [equiv]	Conversion of **2 a** [%]^[b]^	Yield of **3 a** [%]^[b]^
1	0	22	20
**2**	**1.10**	**>95**	**60**
3	0.55	88	43
4	0.10	26	15
5^[c]^	0.10	23	17
6^[d]^	0.55	>95	0

[a] Performed at room temperature for 20 min. [b] Determined by ^19^F qNMR with 3,3′‐bis(trifluoromethyl)benzophenone as internal standard. [c] Use of CaCO_3_ (1.10 equiv) in addition to DABCO. [d] Performed at 60 °C. DABCO=1,4‐diazabicyclo[2.2.2]octane, THF=tetrahydrofuran.

Screening various tertiary amines (Table 2) showed that *N*‐methylpiperidine was superior to DABCO, providing **3 a** in a slightly higher yield (Table 2, entry 1 versus entry 7). Additionally, triethylamine and DIPEA gave **3 a** as the main product, but the yields were lower (Table 2, entries 2 and 3). Unexpectedly, tritylated sulfonamide **3 aa** was preferentially formed with quinuclidine, pyridine, and DMAP (Table 2, entries 4–6).

**Table 2:** Base and solvent screening. 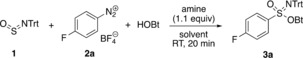

Entry	Amine	Solvent	Yield of **3 a** [%]^[a]^
1	DABCO	MeCN/THF 5:1	60
2	Et_3_N	MeCN/THF 5:1	47
3	DIPEA	MeCN/THF 5:1	44
4	quinuclidine	MeCN/THF 5:1	19^[b]^
5	pyridine	MeCN/THF 5:1	36^[b]^
6	DMAP	MeCN/THF 5:1	8^[b]^
7	*N*MePip	MeCN/THF 5:1	64
8	*N*MePip	MeCN	41
9	*N*MePip	Acetone	>95
**10**	***N*** **MePip**	**DMC**	**>95**
11	*N*MePip	Acetone/DMC 1:1	95

[a] Determined by ^19^F qNMR spectroscopy with 3,3′‐bis(trifluoromethyl)benzophenone as internal standard. [b] Tritylated sulfonamide **3 aa** was the main product. DIPEA=*N*,*N*‐diisopropylethylamine, DMAP=4‐dimethylaminopyridine, Pip=piperidine.

Investigation of solvent systems revealed that both acetone and dimethyl carbonate (DMC) are significantly better than MeCN or the MeCN/THF mixture used before and that both are equally suitable if applied as single‐component solvent (Table 2, entries 7–10). A 1:1 mixture of the two was not more effective (Table 2, entry 11). On the basis of these results and in light of its “green” solvent properties (non‐toxic, water‐immiscible, and environmentally benign),^30^ DMC was selected as medium for the subsequent studies.

As expected for a radical reaction, the presence of dioxygen diminished the yield, whereas a large excess of water did not. Furthermore, the reaction proved sensitive to scale, concentration of the reactants, and addition sequence. Thus, a 10‐fold increase in scale (from the initially used 0.05 mmol to 0.5 mmol) reduced the yield of **3 a** from more than 95 % to 63 % (as determined by ^19^F qNMR spectroscopy). A closer inspection revealed that the order in which the reactants are added is important. Hence, if a solution of HOBt hydrate and *N*‐methylpiperidine in DMC was added dropwise to a suspension of **1** and **2 a** (0.1 mmol L^−1^) in the same solvent, a spectroscopic yield of **3 a** of 77 % was observed. Inverting the addition mode gave **3 a** in only 56 % yield. The importance of a carefully adjusted reactant concentration became even more apparent when the diazonium salt concentration was further changed from the commonly used 0.1 mol L^−1^ to either 0.5 mol L^−1^ or 0.02 mol L^−1^, in which case **3 a** was isolated in 46 % and 69 % yield, respectively. Degassing the solvent led to **3 a** in 78 % yield (at a concentration of 0.02 mol L^−1^). Drying the solvent prior to use had no effect. Those final conditions proved robust for scale‐up, leading to 73 % yield of **3 a** (after column chromatography) on a 10 mmol scale.

**Scheme 3:**
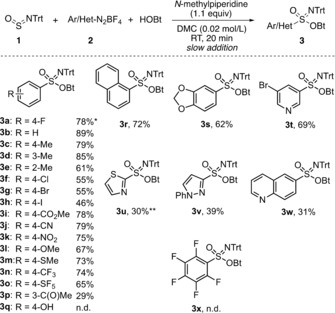
Substrate scope using various diazonium salts. * 73 % yield on a 10 mmol scale; ** use of an undried diazonium salt with a purity of ca. 85 %; n.d.=not detected.

With the optimized conditions in hand, the substrate scope was investigated (Scheme 3). Pleasingly, the reaction conditions were applicable to a wide range of diazonium salts. Phenylsulfonimidate **3 b** was isolated in 89 % yield. Methyl substituents at the *para* or *meta* position of the aryl group reduced the yield of the corresponding sulfonimidate (**3 c** and **3 d**, respectively) slightly. More significant was the effect of an *ortho*‐methyl group, which led to product **3 e** in only 61 % yield. In the series of compounds with *para*‐halo substituents, the yields of **3 a**, **3 f**–**h** dropped with increasing atomic number of the halogen atom. This may be due to the fact that the halogen‐bonding affinity of aryl halides increases with increasing polarizability from fluorine to iodine,^31^ and therefore halogen‐bonding effects between the nitrogen of one diazonium cation and the halogen substituent of another may lead to undesired coordination and thus to side reactions. This already suggests that electrostatic interactions play a role in the product formation, a hypothesis that was later substantiated in control experiments.

As generally expected in radical reactions, electronic effects had only a minor impact. Thus, diazonium salts with both electron‐donating and electron‐withdrawing substituents reacted well, providing the corresponding sulfonimidates **3 i**–**o** in yields ranging from 65 % (for **3 o** with a *para*‐SF_5_ group) to 79 % (for **3 j** with a *para*‐CN substituent). Surprisingly, acetyl‐containing product **3 p** was only obtained in 29 % yield. Due to the lack of reactivity of diazonium salts **2 q** (bearing a free phenolic OH‐group) and **2 x** (containing a pentafluorophenyl moiety), sulfonimidates **3 q** and **3 x** remained inaccessible. In contrast, diazonium salts **2 r** and **2 s**, which bear fused arenes, reacted well, providing 1‐naphthyl or benzo[d][1,3]dioxol‐5‐yl derivatives **3 r** and **3 s** in yields of 72 % and 62 %, respectively. For heteroaromatic sulfonimidates, the yields strongly diverged, and the individual nature of the heterocycle appeared to play a role. Thus, while 3‐bromopyridin‐5‐ylsulfonimidate **3 t** was obtained in 69 % yield, thiazol‐2‐yl, 3‐phenylpyrazol‐1‐yl, and quinolin‐6‐yl derivatives **3 u**–**w** were only formed in the 30 % yield range. At least in part, the latter results might be due to chemical instabilities of the diazonium salts, as particularly observed for **2 u**, which decomposed at temperatures above −10 °C during its synthesis.

**Figure 1:**
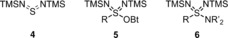
Relevant compounds in the control experiments.

With the goal of shedding light on the reaction mechanism, various control experiments were performed. First, the reaction between **1** and **2 a** was performed in the absence of additional base. In MeCN (for the result with a 5:1 mixture of MeCN and THF a solvent, see Table 1, entry 1), product **3 a** was obtained in low yield (15 %) after 1 h at ambient temperature, thus indicating the critical role of the tertiary base. When the reaction was performed in the presence of two equiv of TEMPO as radical scavengers, the formation of **3 a** was completely suppressed, thus suggesting the intermediacy of radicals as key components. Trapping of such radical by 4‐phenylstyrene (instead of HOBt) proved impossible. Attempts to substitute *N*‐tritylsulfinylamine (**1**) with bis(trimethylsilyl)sulfur diimide (**4**) in the coupling with **2 a** to target a representative of the virtually unknown arylsulfondiimidates **5** or arylsulfondiimidamides **6** 14d, 32 remained unsuccessful (Figure 1). No reaction occurred, thus indicating the importance of the oxygen in reagent **1**. Neither the reaction of **2 a** with **1** and HOBt nor the analogous one with **4** instead of **1** were catalyzed by the addition of copper(I) chloride (10 mol %) with the intention of promoting a Sandmeyer‐type coupling reaction via radicals.

**Scheme 4:**
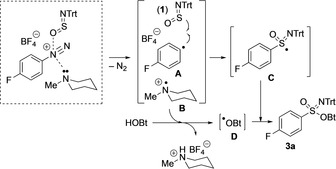
Mechanistic proposal.

The aforementioned observations led us to propose the mechanistic scenario depicted in Scheme 4. In a highly organized (transition) state, both the tertiary amine and sulfinylamine **1** coordinate to the nitrogen of the diazonium salt. Upon electron transfer from the tertiary amine to give radical cation **B**, dinitrogen is expelled and aryl radical **A** is formed. Being close to **1**, aryl radical **A** adds to the sulfur reagent to give sulfoximiminoyl radical **C**. Hydrogen abstraction from HOBt by radical cation **B** generates BtO radical **D** and the HBF_4_ salt of the tertiary amine. In an exergonic process, which provides the driving force of the process, combination of radicals **C** and **D** leads to product **3 a**.

**Scheme 5:**
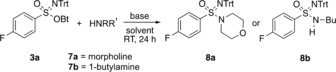
Conversion of sulfonimidate **3 a** into sulfonimidamides **8 a** and **8 b** through reaction with morpholine (**7 a**) or 1‐butylamine (**7 b**), respectively.

The isolated *N*‐trityl‐*O*‐Bt sulfonimidates **3** were white to yellowish solids that could be purified by conventional flash column chromatography in air at room temperature. The decomposition rate on silica is low, and they can be stored at −18 °C over months without significant signs of degradation, thus rending them highly attractive as intermediate for subsequent synthetic applications. Here, we developed their use in the preparation of sulfonimidamides **8** (Scheme 5).

For the initial optimization, morpholine (**7 a**) and 1‐butylamine (**7 b**) were selected as representative nucleophiles. To our delight, both reacted well with sulfonimidate **3 a**, provided that an additional base was added and that acetonitrile was applied as the solvent. For morpholine, sulfonimidamide **8 a** was obtained in up to 77 % yield (as determined by ^19^F qNMR spectroscopy), and **8 b**, stemming from 1‐butylamine, was formed in 86 % yield. As additional bases, *N*‐methylpiperidine and triethylamine were applied, respectively. Attempts to use other solvents than acetonitrile (DMC, acetone, DMF, or pyridine) led to lower conversions and yields. In the reaction of **3 a** with 1‐butylamine (**7 b**), triethylamine could be substituted with potassium carbonate without affecting the yield of **8 b**. Adding catalytic amounts (10 mol %) of potassium iodide or DMAP, which are known catalysts for the aminolysis of methyl esters,^33^ to a mixture **3 a** and **7 b** did not improve the yield **8 b**. Notably, the method did not require dry conditions, and hydrolysis products were not detected.

**Scheme 6:**
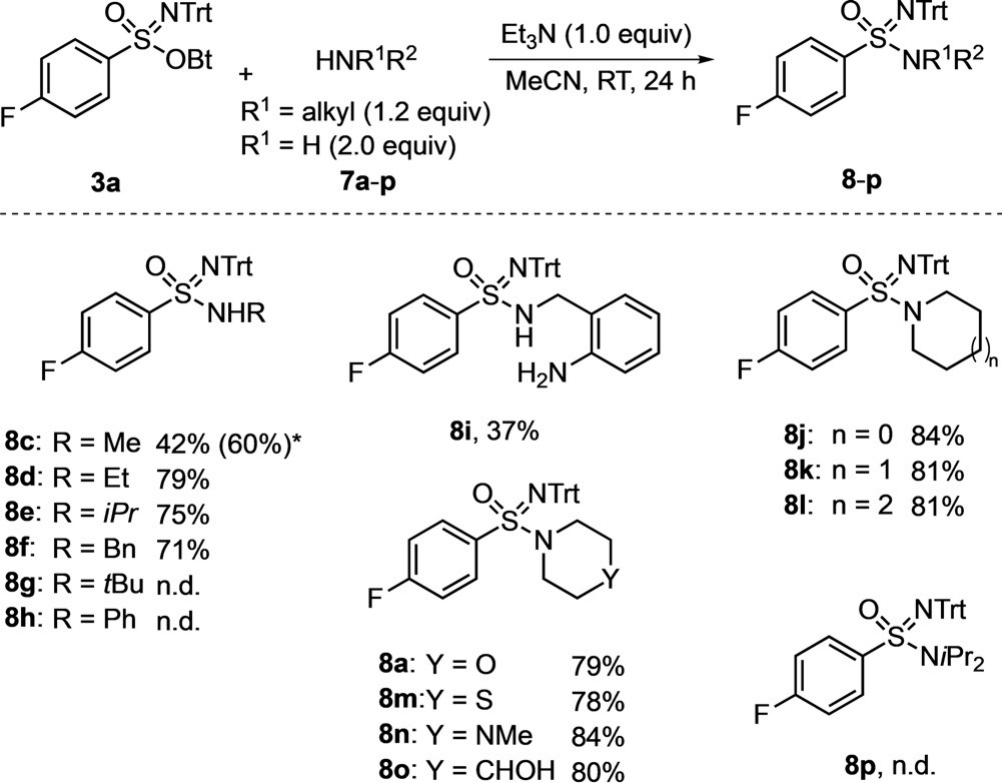
Conversion of sulfonimidate **3 a** into sulfonimidamides **8**. * Use of a large excess of the nucleophile; see text for details.

Scheme 6 summarizes the results of the substrate‐scope evaluation in reactions with **3 a**. As an additional base, 1 equiv of triethylamine was used in acetonitrile for 24 h at ambient temperature. The nucleophile quantities were 2 equiv for primary amines and 1.2 equiv for secondary amines. Most reactions proceeded well, affording the targeted sulfonimidamide **8** in good yields. In the series of primary amines, ethyl‐, *iso*‐propyl‐, and benzylamines led to the corresponding products **8 d**–**f** in yields of 79 %, 75 %, and 71 %, respectively. Applying methylamine (**7 c**) proved less effective under the standard reaction conditions, giving **8 c** in only 42 % yield. This result could be improved by carrying out the substitution in an 8 m ethanolic solution of methylamine (corresponding to an excess of about 28 equiv), which led to **8 c** in 60 % yield. Sulfonimidamides **8 g** and **8 h** remained inaccessible, presumably for steric reasons in the case of *tert*‐butyl amine (**7 g**) and due to low nucleophilicity for aniline (**7 h**). Attempts to apply **7 h** in DMF instead of MeCN, at higher temperatures or after prior deprotonation with sodium hydride, remained unsuccessful. Considering that benzyl amine (**7 f**) reacted well to give **8 f** while aniline (**7 h**) was inactive, 2‐aminobenzylamine (**7 i**), which contains both a benzylic and an anilinic nitrogen, was applied. As hypothesized, the reaction with **3 a** was chemoselective, providing sulfonimidamides **8 i** in 37 % yield. Alicyclic amines reacted almost uniformly, providing products in yields of about 80 %. As revealed by the results for sulfonimidamides **8 j**–**l** stemming from reactions with pyrrolidine (**7 j**), piperidine (**7 k**) and azepane (**7 l**), the ring size had no significant influence. In addition to morpholido sulfonimidamide **8 a**, thiomorpholido and *N*‐methylpiperazido sulfonimidamides **8 m** and **8 n** could be prepared, and again the yields of all products in this series were in the 80 % range. Particularly noteworthy is the 80 % yield for the formation of sulfonimidamide **8 o**, which is derived from **3 a** and 4‐hydroxypiperidine (**7 o**), since it revealed a strong reactivity preference for the *N*‐ over the *O*‐nucleophilic site of the bifunctional amine. This pronounced chemoselectivity is not only of synthetic interest but also explains the observed high resistance of the sufonimidates towards hydrolysis. The employment of other nucleophiles (thiolate, fluoride, and azide) led to product mixtures in which only the corresponding sulfinamide (4‐fluoro‐*N*‐tritylbenzenesulfinamide) could be identified.

**Scheme 7:**
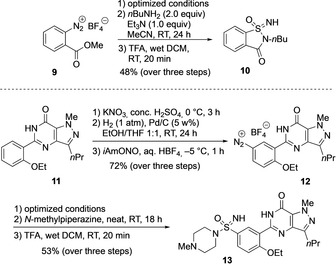
Syntheses of two analogues of pharmacologically relevant sulfonamides. TFA=trifluoroacetic acid, DCM=dichloromethane.

In order to demonstrate the practicability of the new sulfonimidamide synthesis, two analogues of pharmacologically relevant sulfonamides were prepared (Scheme 7). First, *endo*‐*N*‐*n*‐butyl‐substituted azasaccharine derivative **10** was obtained from 2‐carboxymethylphenyldiazonium tetrafluoroborate **9** in a total yield of 48 % over three steps. This product is of interest because Chen and co‐workers have shown that saccharine aza bioisosters such as compounds of type **10** exhibit promising preclinical properties.^34^ The second molecule in this series was an analogue of sildenafil, which is a commercial PDE5 inhibitor with a sulfonamide core. Diazonium salt **12** was prepared in an overall yield of 72 % over several steps from the commercially available **11**. Using the aforementioned optimized conditions for the sulfonimidate formation, followed by treatment with *N*‐methylpiperazine and acidic deprotection of the trityl group, **11** was converted into sulfonimidate **13** in 53 % yield over three steps.

## Conclusion

We have introduced new methods for the mild preparation of activated sulfonimidates and sulfonimidamides. First, aromatic or heteroaromatic diazonium salts are reacted with the bench‐stable reagent *N*‐tritylsulfinylamine (**1**) and 1‐hydroxybenzotriazole hydrate in the presence of *N*‐methylpiperidine to give a broad range of *N*‐trityl‐*O*‐benzotriazolylsulfonimidates **3**. Non‐toxic, environmentally benign dimethyl carbonate is the solvent. The reaction takes place at room temperature without the requirement for dry conditions. Degassing of the solvent and a low reactant concentration have a beneficial effect on the yields, but neither is critical for the success of the transformations. Many functional groups are tolerated, and the electronic properties of the substituents have only a minor effect on the product yields. In a subsequent step, the sulfonimidates **3** can be converted into the corresponding sulfonimidamides **8** through simple reactions with primary or secondary amines, whereby only aliphatic amines react. This chemoselectivity is without precedent. The formation of the sulfonimidates probably takes place through a radical mechanism involving pre‐organized aggregates of the reactants. A novel azasaccharine derivate and an unprecedented sulfonimidamide analogue of sildenafil were prepared by using these methods.

## Conflict of interest

The authors declare no conflict of interest.

## Supporting information

As a service to our authors and readers, this journal provides supporting information supplied by the authors. Such materials are peer reviewed and may be re‐organized for online delivery, but are not copy‐edited or typeset. Technical support issues arising from supporting information (other than missing files) should be addressed to the authors.

Supplementary
