# Supplementary material for: Additions to N‐Sulfinylamines as an Approach for the Metal‐free Synthesis of Sulfonimidamides: O‐Benzotriazolyl Sulfonimidates as Activated Intermediates
Source: Angew Chem Int Ed Engl. 2019 Nov 11;58(52):19014–20. doi: 10.1002/anie.201911075 (PMC6972565; doi:10.1002/anie.201911075)
Supplement: Supplementary file 1 — Supplementary [file ANIE-58-19014-s001.pdf]

Angewandte Chemie
Eine Zeitschrift der Gesellschaft Deutscher Chemiker

Supporting Information

Additions to *N*-Sulfinylamines as an Approach for the Metal-free Synthesis of Sulfonimidamides: *O*-Benzotriazolyl Sulfonimidates as Activated Intermediates

*Maximilian Bremerich, Christian M. Conrads, Tim Langletz, and Carsten Bolm**

anie_201911075_sm_miscellaneous_information.pdf

SUPPORTING INFORMATION

Table of Contents

1. General information S2
2. Optimization experiments S4
3. Experimental standard procedures S13
4. Synthesized compounds S16
5. References S53
6. NMR spectra S54

1. General information

Unless otherwise stated, the reactions were conducted under air. Glass equipment was not dried before use. If reactions were conducted under inert atmosphere, argon was used as inert gas.

Unless otherwise stated, chemicals were used as received without any further purification. The following solvents used for column chromatography were purchased at technical grade and were distilled at atmospheric pressure: dichloromethane, diethyl ether, ethyl acetate and pentane. Dimethyl carbonate (DMC) was distilled at reduced pressure before it was used for synthesis. Diethyl ether was dried by a solvent drying system (MBraun SPS5).

Separation Methods

Column Chromatography

Column chromatography was performed under up to 0.4 bar overpressure on silica gel (35 μm to 60 μm) purchased from Acros Organics.

Thin-Layer Chromatography (TLC)

For TLC analysis, ALUGRAM® Xtra SIL G/UV254 TLC plates (0.2 mm silica gel, 5 cm length) from VWR were used. In addition to UV fluorescence quench detection (254 nm), various staining reagents were used. A solution of ninhydrin in ethanol (1%) was used to detect primary amines. Secondary amines, sulfonamides and sulfonimidamides (with one free NH-group) could be visualized by a *t*BuOCl/tolidine stain. For this purpose, the TLC plate was first treated with a solution of *tert*-butyl hypochlorite in pentane (0.5%, has to be stored in the freezer), then dried under moderate heat (ca. 50 °C, heat gun), and finally dipped in into a solution of tolidine (1%) in 5% acetic acid with 0.1% sodium iodide. OBt-derivatives could be identified my simple, harsh heating of the TLC plate, upon which they formed brown spots.

Analytics

NMR Spectroscopy

NMR spectra were recorded with an Agilent VNMR 600 spectrometer (600 MHz for ^{1}H NMR spectra, 151 MHz for ^{13}C NMR spectra and 564 MHz for ^{19}F NMR spectra) and an Agilent VNMR 400 (400 MHz for ^{1}H NMR and 100 MHz for ^{13}C NMR spectra). $CDCl_3$ and CD_3CN were used as deuterated solvents. The chemical shifts (δ) are given in ppm (parts per million) referenced to the signals of the non-deuterated solvents (^{1}H NMR: δ($CHCl_3$) = 7.26 ppm, δ(CH_3CN) = 1.94 ppm. ^{13}C NMR: δ($CHCl_3$) = 77.2 ppm, δ(CH_3CN) = 118.6 ppm). Coupling constants (*J*) are given in Hz. Multiplets are named by the following abbreviations: singlet (s), doublet (d), triplet (t), quartet (q), multiplet (m).

Infrared Spectroscopy

IR spectra were recorded with a PerkinElmer Spectrum 100 FT-IR spectrometer with an UATR Diamond KRS-5 unit. The wavenumbers of the bands are given in cm^{-1}.

Mass spectrometry

Mass spectra were recorded with a Finnigan SSQ7000 or a Finnigan MAT-95 spectrometer. The peaks are given in atomic mass units per elementary charge (*m*/*z*). The intensity was given in percent with respect to the base peak. High-resolution mass spectrometry (HRMS) was performed with a Thermo Scientific LTQ Orbitrap XL spectrometer.

Determination of Melting Points

Melting points were determined using a Büchi Melting Point M-560 device. The melting points of diazonium salts were not determined because of their potentially explosive behavior. Moreover, the sulfonimidates **3** do not show sharp melting points, but gradually brown and decompose at elevated temperature.

2. Optimization experiments

General remarks

All reactions were analyzed by ^{19}F qNMR on an Agilent VNMR 600 spectrometer (564 MHz). Determination of yields and conversion were calculated against 3,3'-bis(trifluoromethyl)benzophenone as internal standard, which was added to the stock solution of the diazonium salt. The expected products were synthesized by known procedures, and their chemical shift in MeCN/CD_3CN mixtures was determined. If not indicated otherwise, all reactions were performed on air in non-dried solvents in 4-mL dram vials. MeCN/CD_3CN mixtures were used instead of pure MeCN to guarantee a proper lock signal. If solvents other than MeCN were used, CD_3CN was added after the end of the reaction. The yield for the unknown product was given assuming that it contains only one fluorine atom. All liquids were handled with calibrated Eppendorf pipettes.

Chemical shifts of the observed products:

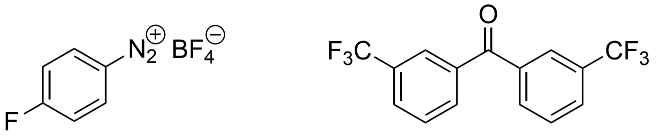

^{19}F NMR shift (ppm, in MeCN)

2a –85.1

–63.3

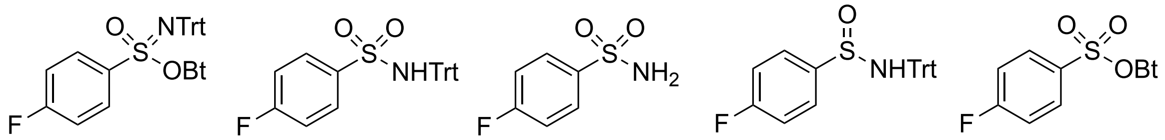

–103.7	–109.7	–108.6	–112.2	ca. –98
**3a**	**3aa**	**3ab**	**3ac**	**3ad**

Table S1: Influence of the stoichiometric ratio of DABCO on the product spectrum of the reaction.[a]

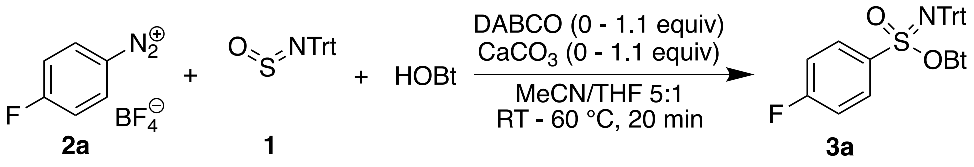

**Entry**	**DABCO [equiv]**	**Additive [equiv]**	**Conv. of 2a [%]**	**3a**	**3aa**	**3ab**	**3ac**	**3ad**	Unidentified compound
1	0	-	22	20	1	0	0	0	2
2	1.10	-	100	60	2	1	0	0	16
3	0.55	-	88	43	7	0	0	0	12
4	0.10	-	26	15	10	0	0	0	3
5	0.10	$CaCO_3$ (1.10)	23	17	11	0	0	0	4
6[b]	0.55	-	100	0	38	4	12	2	0

[a] All numbers in the table correspond to yields determined relative to the internal standard; scale: 60 μmol, mixing of standard solutions [A: diazonium salt (0.15 M) and *N*-tritylsulfinylamine (0.165 M) in MeCN (0.400 mL); B: HOBt hydrate (0.66 M) in THF (0.100 mL); C: DABCO (0.66 M) in MeCN (varying volumes)]; all experiments were filled to a volume of 0.600 mL with MeCN; [b] 60 °C.

Table S2: Screening of different tertiary amines.[a]

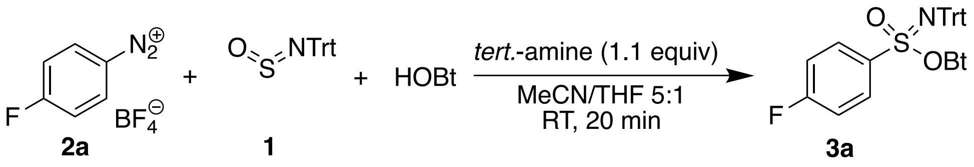

Entry	Amine	3a	3aa	3ab	3ac	Unidentified compound
1	DABCO	60	6	0	0	16
2	Et_3N	47	5	4	0	14
3	DIPEA	44	5	8	0	15
4	quinuclidine	19	46	1	0	19
5	pyridin	36	4	0	0	9
6	DMAP	8	40	0	0	13
7	DBU	53	3	11	1	24
8	*N*-methylmorpholine	51	6	3	0	11
9	*N*-methylpiperidine	64	4	3	0	15
10	*N*-methylimidazole	58	0	0	0	13
**11**	urotropine	40	1	4	0	8
**12**	TMEDA	53	6	9	0	23
**13**	(±)-Tröger's base	22	4	3	0	7
**14**	(-)-sparteine	35	9	2	0	17
**15**	(+)-quinidine	47	4	7	0	12

[a] All numbers in the table correspond to yields determined relative to the internal standard; scale: 60 μmol, mixing of standard solutions [A: diazonium salt (0.15 M) and *N*-tritylsulfinylamine (0.165 M) in MeCN (0.400 mL); B: HOBt hydrate (0.66 M) in THF (0.100 mL); C: bases (0.66M) in MeCN (0.100 mL].

Table S3: Screening of different solvents - part 1.[a]

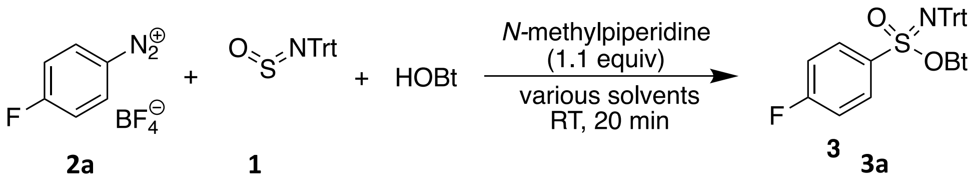

2a 1 3 3a

Entry	Solvent	3a	3aa	3ab	3ac	3ad	Unidentified compound
1	MeCN	41	2	0	0	0	0
2	acetone	60	1	1	0	0	3
3[b]	$CHCl_3$	17	2	0	0	0	1
4[c]	DCM	41	1	1	0	0	2
5	EtOAc	48	1	1	1	0	1
6	PhMe	9	0	0	0	0	0
7	DMC	61	1	2	0	0	0
8	DME	55	1	0	0	0	0
9	1,4-dioxane	35	2	1	0	0	0
10	THF	24	2	1	0	1	6
11	Et_2O	14	1	1	0	0	2
12	MeOH	5	1	1	0	0	0
13	EtOH	12	1	2	2	0	0

[a] All numbers in the table correspond to yields determined relative to the internal standard; scale: 60 μmol; experiments performed by mixing a solution of diazonium salt (0.15 M) and TrtNSO (0.165 M) in MeCN (0.400 mL) with a solution of HOBt hydrate (0.66 M) in THF (0.100 mL) and evaporation to dryness for 6 min under a stream of nitrogen, replenishing with the corresponding solvent (0.500 mL) and addition of *N*-methylpiperidine; [b] 82% conversion; [c] 98% conversion.

Table S4: Screening of different solvents - part 2.[a]

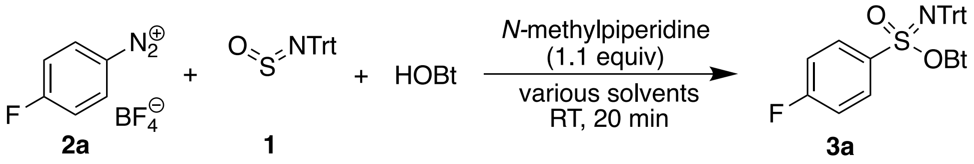

Entry	solvent	3a	3aa	3ab	3ac	3ad	Unidentified compound
1	DMC	61	5	5	0	0	4
2	EMK	51	0	0	0	0	21
3	DMF	25	5	11	0	0	31
4	DMAc	38	4	5	0	30	0
5	formamide	0	0	0	0	0	13
6	NMP	15	3	7	0	0	64
7	1,2-DCE	44	5	0	0	0	10
8	$MeNO_2$	0	0	0	35	0	0

[a] All numbers in the table correspond to yields determined relative to the internal standard; scale: 60 μmol; experiments performed by mixing a solution of diazonium salt (0.15 M) and TrtNSO (0.165 M) in MeCN (0.400 mL) with a solution of HOBt hydrate (0.66 M) in THF (0.100 mL); evaporating under reduced pressure, repleneshing with the corresponding solvent (0.500 mL) and addition of *N*-methylpiperidine under argon. In all experiments full turnover was observed.

Table S5: Influence of water and oxygen.[a]

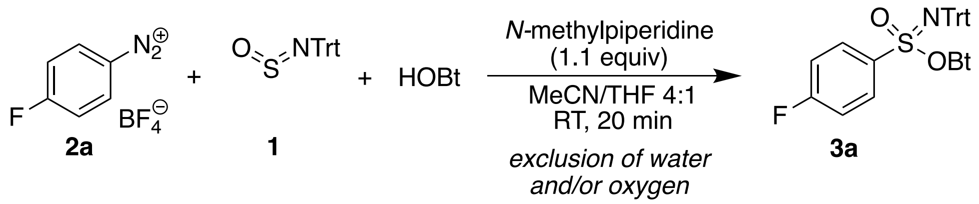

**Entry**	**Atmosphere**	**Water [equiv]**	**3a**	**3aa**	**3ab**	**3ac**	**3ad**	Unidentified compound
1	argon	-	56	2	2	0	0	15
2	argon	10	51	2	1	0	0	15
3	dioxygen	-	42	6	0	1	1	13
4	air	-	54	1	1	0	0	12

[a] All numbers in the table correspond to yields determined relative to the internal standard; scale: 50 μmol; experiments performed by mixing a solution of diazonium salt (0.125 M) and TrtNSO (0.138 M) in dry MeCN (0.400 mL) with a solution of HOBt hydrate (0.55 M) in dry THF (0.100 mL) and distribution to four different oven-dried NMR tubes with the indicated atmospheres, followed by the addition of *N*-methylpiperidine; In all experiments full turnover was observed.

Table S6: Variation of the stochiometric ratios of HOBt und TrtNSO.[a]

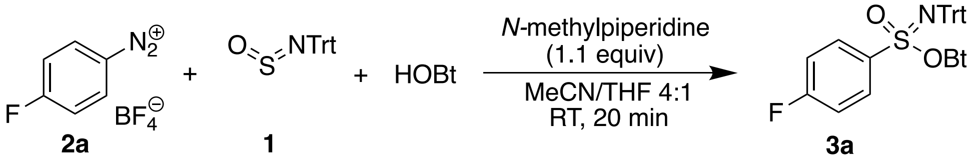

Entry	HOBt [equiv]	TrtNSO [equiv]	3a
1	0	1.10	0
2	0.50	1.10	19
3	1.10	1.10	50
4	1.50	1.10	60
5	2.00	1.10	58
6	2.50	1.10	58
7	3.00	1.10	52
8	4.00	1.10	57
9[b]	1.00	1.10	78
10[b]	1.00	1.30	79

[a] All numbers in the table correspond to yields determined relative to the internal standard; scale: 50 μmol, entries 1-8: experiments performed by mixing a solution of diazonium salt (0.125 M) and TrtNSO (0.138 M) in MeCN (0.400 mL) with solid HOBt hydrate (0 – 4.00 equiv), addition of THF (0.100 mL) and subjecting to ultrasound (30 s), followed by the addition of *N*-methylpiperidine. Entries 9-10: experiments performed by addition of *N*-methylpiperidine to a suspension of diazonium salt (0.10 M), TrtNSO (0.11 M or 0.13 M) and HOBt hydrate (0.10 M) in DMC (0.500 mL). In all experiments except entry 1 full turnover was observed; [b] in DMC.

Table S7: Identification of solvent synergies and the optimal reaction temperature.[a]

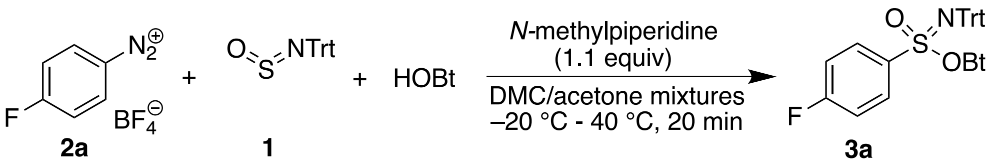

Entry	DMC [v%]	Acetone [v%]	T [°C]	3a [%]
1	50	50	-20	31
2	50	50	0	79
3	50	50	rt	95
4	50	50	40	85
5	0	100	rt	>95
6	100	0	rt	>95

[a] All numbers in the table correspond to yields determined relative to the internal standard; scale: 50 μmmol, c = 0.1 M; entries 1-4: experiments performed by mixing equal volumes (0.250 mL) of a solution of the diazonium salt (0.2 M), TrtNSO (0.22 M) and internal standard in acetone with a solution of *N*-methylpiperidine (0.22 M) and HOBt hydrate (0.26 M) in DMC (0.250 mL); entries 5-6: experiments performed by adding *N*-methylpiperidine to a solution/suspension (0.500 mL) of diazonium salt (0.1 M), TrtNSO (0.11 M), and HOBt hydrate (0.13 M) and internal standard in acetone or DMC; all solutions were brought to the indicated reaction temperature before mixing; in all experiments full conversion was observed.

Table S8: Influence of scale, concentration and addition mode.[a]

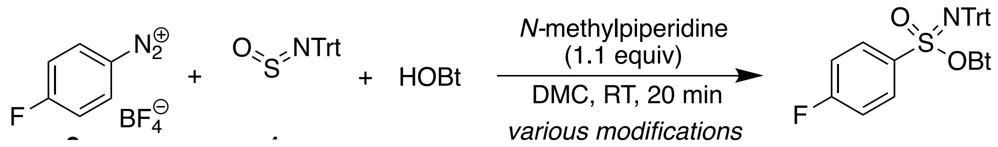

2a 1 3a

Entry	Scale [mmol]	Concentration [mmol/L]	Addition mode	3a [%][b]
1	0.5	0.1	A	63
2	0.5	0.1	B	77[c]
3	0.5	0.1	C	56[c]
4	0.5	0.1	C	59
5	0.5	0.5	B	46
6	0.5	0.02	B	69
7[d]	0.5	0.02	B	78 (82)[c]
8[d,e]	0.5	0.02	B	78
9[d]	10	0.02	B	74

[a] All reactions under argon; TrtNSO (1.1 equiv), HOBt hydrate (1.3 equiv); addition modes: A: Base was added in one portion to the reaction mixture; B: A solution of HOBt hydrate and base was added dropwise to a suspension of diazonium salt and TrtNSO; C: A suspension of diazonium salt and TrtNSO was added dropwise (glass pipette) to a solution of HOBt hydrate and *N*-methylpiperidine; [b] yield after flash column chromatography; [c] spectroscopic yield; [d] degassed solvent; [e] dry solvent.

3. Experimental standard procedures

Standard procedure A: Diazotization with sodium nitrite

This procedure was preferred for non-polar and liquid anilines.

Water (the same volume as the tetrafluoroboric acid) and the aniline (1 equiv) were mixed in a 25 mL round-bottom flask equipped with a magnetic stirrer bar and a PVC-coated internal thermometer at room temperature. Aqueous tetrafluoroboric acid (48 w%, 2.6 equiv) was added with a glass pipette. Subsequently, the mixture was cooled down to a temperature of circa –7 °C using an ice-salt bath. If the aniline did not dissolve, additional water or ethanol was added dropwise, until a homogenous solution was obtained. Then, a concentrated, cooled (ca. 0 °C) aqueous solution of sodium nitrite (1.0 equiv) was added over a period of circa 1 h dropwise in such a manner that the temperature never exceeded 0 °C. (The reaction was strongly exothermic; addition of a few drops $NaNO_2$ solution often led to an increase in temperature of around 0.5 °C). After the addition was finished (ca. 30 – 60 min), the mixture was stirred for one additional hour below 1 °C. The resulting slurry was sucked dry, and the obtained crude product was redissolved in a minimal amount of cold (–18 °C) acetone. Precipitation by the portion-wise addition of cold (–18 °C) diethyl ether afforded a purified product. This procedure was repeated once more, and the resulting pure product was filtered off and washed with a small amount of cold diethyl ether. The product was dried under reduced pressure at 0 °C in the dark and stored in the dark at –21 °C.

Standard procedure B: Diazotization with isoamyl nitrite

This procedure was preferred for more water-soluble or solid anilines.

The second procedure for the synthesis of diazonium compounds corresponds mainly to standard procedure A. Ethanol or *tert*-amyl alcohol were employed as solvent. Instead of an aqueous sodium nitrite solution, isoamyl nitrite (1.0 equiv) was added dropwise so that the temperature never rose above 0 °C. Since the reaction is far less exothermic than procedure A, the addition could be carried out faster and took around minutes. The slurry was afterwards stirred for one additional hour below 1 °C. The workup was the same as in standard procedure A.

Notes:

- Handling of the diazonium salts was exclusively performed with plastic spatulas. Care was taken to prevent static charge of those.
- NMR analysis of the diazonium salts was conducted by dissolving them in cold (ca. 4 °C) CD_3CN and subjecting them to the spectrometry within 5 min. Longer standing times caused the formation of visible amounts of degradation products, so did the use of other solvents [$(CD_3)_2CO$, $(CD_3)_2SO$].

Standard procedure C: Sulfonimidates

Dimethyl carbonate was degassed by three freeze-pump-thaw cycles. A 50 mL-Schlenk flask was charged with HOBt hydrate (100 mg, 0.65 mmol, 1.30 equiv), and the flask was purged with argon. Then, degassed dimethyl carbonate (12.5 mL) and *N*-methylpiperidine (67 μL, 0.55 mmol, 1.10 equiv) were added. The mixture was stirred until the solid was completely dissolved.

Another 50 mL-Schlenk-flask was charged with the diazonium salt (0.50 mmol, 1.00 equiv) and *N*-tritylsulfinylamine (168 mg, 0.55 mmol, 1.10 equiv). The flask was purged with argon, and degassed dimethyl carbonate (25 mL, initial concentration 0.02 M) was added. The HOBt solution was added over a period of 20 minutes to the suspension of the diazonium salt using a syringe pump under the exclusion of light and rapid stirring at room temperature. The faint yellow reaction mixture was stirred for additional 5 minutes. Then, the solvent was evaporated under reduced pressure (40 °C, rotovap). Solvent residues were removed by repeated azeotropic distillation with DCM. The crude product was purified by flash column chromatography (silica). Eluent mixtures where varied according to the product. The products were stored under argon at –21 °C.

Notes:

- For larger amounts of DMC, degassing can be achieved by bubbling inert gas through it (see upscaling of **3a**).
- HOBt hydrate does not dissolve in DMC unless a base is added. Ultrasound can be used to assist dissolving.
- The color of the reaction mixture is good indicator for the presence of oxygen; for non-colored diazonium salt, it should be faint yellow. In the presence of

oxygen, an orange or even red color can be observed. This rule cannot be applied for colored diazonium salts (like **2q**, **2r**, **2s**, **2w**).

- The water bath temperature of the rotovap should not exceed 40 °C, and the crude mixture should not be exposed to that temperature longer than necessary, since the yield can be considerably diminished.
- The sulfonimidates should not be exposed to silica longer than necessary. Especially, it should be avoided to adsorb them onto silica or Celite® prior to the chromatographic purification; loading the column can be achieved with solutions in a minimal amount of DCM.
- Pentane/EtOAc mixtures are unsuitable for a successful purification, since they coelute tritylamine, which tends to smear on silica. Pentane/DCM/Et_2O or pentane/DCM/MTBE mixtures are superior. Purity of the collected fractions should be assigned with the *t*BuOCl/tolidine stain (see above).
- For many cases, the sulfonimidates do not have to be isolated, but can be used crude.
- Because of the strong fragmentation tendency of the trityl- and the OBt-group, mass spectra of the sulfonimidates were dominated by the corresponding peaks, and showed only little difference with regards to the aryl substitution. Conventional mass spectrometry analysis was therefore sometimes omitted, and only HRMS experiments were performed.

Standard procedure D: Sulfonimidamides

In a 4 mL-dram vial, the sulfonimidate (0.30 mmol, 1.0 equiv), the amino nucleophile (0.36 mmol, 1.2 equiv for secondary amines, 0.6 mmol, 2.0 equiv for primary amines) and triethylamine (0.30 mmol, 1.0 equiv) were dissolved in acetonitrile (1 mL, 0.3 M). The vial was capped on air, and the resulting mixture was stirred at ambient temperature for 24 h. The orange mixture was then diluted with DCM (5 mL), adsorbed on Celite®, and the crude product was purified by column chromatography (SiO_2).

Notes:

- Occasionally, a white solid precipitates out of the mixture, and might also redissolve again. This has no influence on the yield.

4. Synthesized compounds

Diazonium salts

The following diazonium salts were purchased: 4-bromobenzenediazonium tetrafluoroborate (**2g**; Aldrich), 4-nitrobenzenediazonium tetrafluoroborate (**2k**; Alfa Aesar), and 4-methoxybenzenediazonium tetrafluoroborate (**2l**; Sigma-Aldrich). The purchased diazonium compounds were purified by precipitation from cold acetone/diethyl ether mixtures (repeated twice, see above) prior to their use in synthesis. 4-Chlorobenzenediazonium tetrafluoroborate (**2f**) was synthesized in the research group and was used without any further purification.

4-Fluorobenzenediazonium tetrafluoroborate (2a)

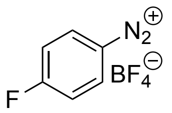

2a

The synthesis was performed according to standard procedure A starting from aniline (1480 mg, 13.3 mmol 1.00 equiv), aqueous tetrafluoroboric acid (48 w%, 36.6 mmol, 4.8 mL, 2.8 equiv), sodium nitrite (919 mg, 13.3 mmol, 1.0 equiv) and water (5 mL). Benzenediazonium tetrafluoroborate (2563 mg, 12.2 mmol, 92%) was obtained as white solid.

^{1}H NMR (600 MHz, CD_3CN): δ = 8.62 – 8.56 (m, 1H), 7.70 – 7.63 (m, 1H) ppm.

Benzenediazonium tetrafluoroborate (2b)

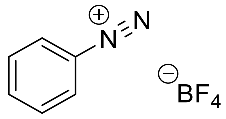

2b

The synthesis was performed according to standard procedure A starting from aniline (1509 mg, 16.2 mmol 1.00 equiv), aqueous tetrafluoroboric acid (48 w%, 3630 mg, 41.3 mmol, 5.4 mL, 2.6 equiv), sodium nitrite (1118 mg, 16.2 mmol, 1.0 equiv) and water (10 mL). Benzenediazonium tetrafluoroborate (2693 mg, 14.0 mmol, 87%) was obtained as white solid.

^{1}H NMR (400 MHz, CD_3CN): δ = 8.53 – 8.45 (m, 2H), 8.25 (tq, *J* = 7.8, 1.1 Hz, 1H), 7.93 (tt, *J* = 7.8, 1.0 Hz, 2H) ppm.

4-Methylbenzenediazonium tetrafluoroborate (2c)

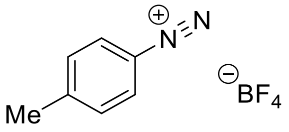

2c

The synthesis was performed according to standard procedure A starting from *p*-toluidine (2764 mg, 25.8 mmol, 1.0 equiv), aqueous tetrafluoroboric acid (48 w%, 5893 mg, 67.1 mmol, 8.8 mL, 2.6 equiv), sodium nitrite (1779 mg, 25.8 mmol, 1.0 equiv) and water (10 mL). 4-Methylbenzenediazonium tetrafluoroborate (5020 mg, 24.0 mmol, 95%) was obtained as white solid.

^{1}H NMR (400 MHz, CD_3CN): δ = 8.39 – 8.34 (m, 2H), 7.76 – 7.70 (m, 2H), 2.60 (s, 3H) ppm.

3-Methylbenzenediazonium tetrafluoroborate (2d)

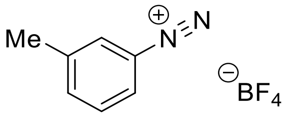

2d

The synthesis was performed according to standard procedure A starting from *m*-toluidine (1097 mg, 10.2 mmol, 1.0 equiv), aqueous tetrafluoroboric acid (48 w%, 3.5 mL, 27.0 mmol 2.6 equiv), sodium nitrite (706 mg, 10.2 mmol, 1.0 equiv) and water (4 mL). 3-Methylbenzenediazonium tetrafluoroborate (1617 mg, 7.85 mmol, 77%) was obtained as white solid.

^{1}H NMR (400 MHz, CD_3CN): δ = 8.35 – 8.25 (m, 2H), 8.07 (d, *J* = 7.9 Hz, 1H), 7.81 (t, *J* = 8.1 Hz, 1H), 2.52 (s, 3H) ppm.

2-Methylbenzenediazonium tetrafluoroborate (2e)

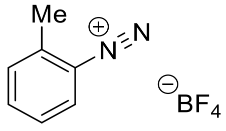

2e

The synthesis was performed according to standard procedure A starting from *o*-toluidine (553 mg, 5.16 mmol, 1.0 equiv), tetrafluoroboric acid (48 w%, 1.8 mL, 13.4 mmol, 2.6 eq), sodium nitrite (356 mg, 5.16 mmol, 1.0 equiv) and water (2 mL). 2-Methylbenzenediazonium tetrafluoroborate (673 mg, 3.27 mmol, 63%) was obtained as white solid.

^{1}H NMR (400 MHz, CD_3CN): δ = 8.42 (dd, *J* = 8.4, 1.3 Hz, 1H), 8.13 (td, *J* = 7.8, 1.3 Hz, 1H), 7.80 – 7.69 (m, 2H), 2.73 (s, 3H) ppm.

4-Iodobenzenediazonium tetrafluoroborate (2h)

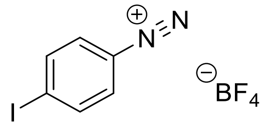

2h

The synthesis was performed according to standard procedure A starting from *p*-iodoaniline (1008 mg, 4.60 mmol, 1.0 equiv), aqueous tetrafluoroboric acid (48 w% , 1051 mg, 12.0 mmol, 2.6 equiv, 1.6 mL), sodium nitrite (317 mg, 4.60 mmol, 1.0 equiv), ethanol (2 mL) and water (2 mL). (4-Iodophenyl)diazonium tetrafluoroborate (767 mg, 2.41 mmol, 52%) was obtained as white solid.

^{1}H NMR (400 MHz, CD_3CN): δ = 8.36 – 8.29 (m, 1H), 8.18 – 8.12 (m, 1H) ppm.

4-(Methoxycarbonyl)benzenediazonium tetrafluoroborate (2i)

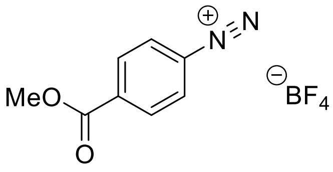

2i

The synthesis was performed according to standard procedure B starting from methyl-*p*-anthranilate (1482 mg, 9.8 mmol, 1.0 equiv), aqueous tetrafluoroboric acid (48 w%, 3.3 mL, 25.5 mmol, 2.6 equiv), isoamyl nitrite (1.44 mL, 10.8 mmol, 1.1 equiv), water (2 mL) and *tert*-amyl alcohol (4 mL). 4-(Methoxycarbonyl)benzene-diazonium tetrafluoroborate (1088 mg, 4.35 mmol, 44%) was obtained as white solid.

^{1}H NMR (400 MHz, CD_3CN): δ = 8.61 (dt, *J* = 9.1, 1.7 Hz, 2H), 8.44 – 8.37 (m, 2H), 3.97 (s, 3H) ppm.

4-Cyanobenzenediazonium tetrafluoroborate (2j)

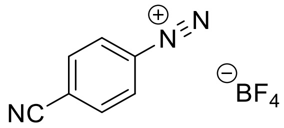

2j

The synthesis was performed according to standard procedure B starting from 4-aminobezonitrile (1104 mg, 9.34 mmol, 1.0 equiv), aqueous tetrafluoroboric acid (48 w%, 3.2 mL, 24.3 mmol, 2.6 equiv), isoamyl nitrite (1.37 mL, 10.3 mmol, 1.1 equiv), ethanol (8 mL) and water (2 mL). 4-Cyanobenzenediazonium tetrafluoroborate (1703 mg, 7.85 mmol, 84%) was obtained as white solid.

^{1}H NMR (400 MHz, CD_3CN): δ = 8.65 – 8.59 (m, 2H), 8.28 – 8.22 (m, 2H) ppm.

4-(Methylthio)benzenediazonium tetrafluoroborate (2m)

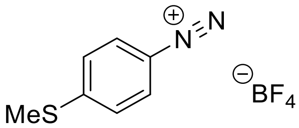

2m

The synthesis was performed according to standard procedure A starting from 4-thiomethylaniline (373 mg, 2.68 mmol, 1.0 equiv), aqueous tetrafluoroboric acid (48 w%, 0.9 mL, 6.97 mmol, 2.6 equiv), sodium nitrite (185 mg, 2.68 mmol, 1.0 equiv) and water (1 mL). 4-(Methylthio)benzenediazonium tetrafluoroborate (398 mg, 1.67 mmol, 62%) was obtained as a yellow powder.

^{1}H NMR (400 MHz, CD_3CN): δ = 8.28 – 8.22 (m, 1H), 7.68 – 7.63 (m, 1H), 2.66 (s, 2H) ppm.

4-(Trifluoromethyl)benzenediazonium tetrafluoroborate (2n)

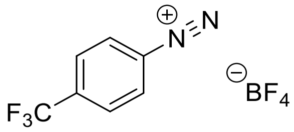

2n

The synthesis was performed according to standard procedure A starting from aminobenzotrifluoride (1576 mg, 9.78 mmol, 1.0 equiv), aqueous tetrafluoroboric acid (48 w%, 3.3 mL, 25.5 mmol, 2.6 equiv,), sodium nitrite (675 mmol, 9.78 mg, 1.0

equiv) and water (4 mL). (4-Trifluoromethyl)benzenediazonium tetrafluoroborate (1217 mg, 4.69 mmol, 48%) was obtained as white solid.

^{1}H NMR (400 MHz, CD_3CN): δ = 8.73 – 8.67 (m, 1H), 8.26 – 8.19 (m, 1H) ppm.

4-(Pentafluoro-λ^6-sulfaneyl)benzenediazonium tetrafluoroborate (2o)

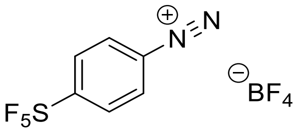

2o

The synthesis was performed according to standard procedure B starting from 4-(pentafluoro-λ^6-sulfaneyl)aniline (1502 mg, 6.85 mmol, 1.0 equiv), aqueous tetrafluoroboric acid (48 w%, 2.3 mL, 17.8 mmol, 2.6 equiv), isoamyl nitrite (1.01 mL, 7.54 mmol, 1.1 equiv) and ethanol (2 mL). 4-(Pentafluoro-λ^6-sulfaneyl)-benzenediazonium tetrafluoroborate (1766 mg, 5.55 mmol, 81%) was obtained as white solid.

^{1}H NMR (400 MHz, CD_3CN): δ = 8.76 – 8.69 (m, 1H), 8.40 – 8.33 (m, 1H) ppm.

3-Acetylbenzenediazonium tetrafluoroborate (2p)

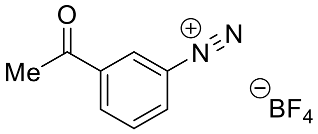

2p

The synthesis was performed according to standard procedure A starting from 3-aminoacetophenone (1207 mg, 8.93 mmol, 1.0 equiv), aqueous tetrafluoroboric acid (48 w%, 3.0 mL, 23.2 mmol, 2.6 equiv), sodium nitrite (616 mg, 8.93 mmol, 1.0 equiv) and water (3 mL). 3-Acetylbenzenediazonium tetrafluoroborate (2089 mg, 3.25 mmol, 36%) was obtained as white solid.

^{1}H NMR (400 MHz, CD_3CN): δ = 8.98 (t, *J* = 2.0 Hz, 1H), 8.72 (dt, *J* = 8.1, 1.3 Hz, 1H), 8.63 (ddd, *J* = 8.3, 2.2, 1.1 Hz, 1H), 8.06 (t, *J* = 8.2 Hz, 1H), 2.67 (s, 3H) ppm.

4-Hydroxybenzenediazonium tetrafluoroborate (2q)

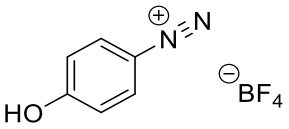

2q

The synthesis was performed according to standard procedure B starting from *p*-hydroxyaniline (1058 mg, 9.69 mmol, 1.0 equiv), tetrafluoroboric acid (54 w% in Et_2O, 3.3 mL, 24.1 mmol, 2.5 equiv), isoamyl nitrite (1.43 mL, 10.7 mmol, 1.1 equiv) and ethanol (2 mL). 4-Hydroxybenzenediazonium tetrafluoroborate (660 mg, 3.17 mmol, 33%) was obtained as light purple powder.

^{1}H NMR (400 MHz, CD_3CN): δ = 8.83 (s, 1H), 8.35 – 8.26 (m, 2H), 7.24 – 7.15 (m, 2H) ppm.

Naphthalene-1-diazonium tetrafluoroborate (2r)

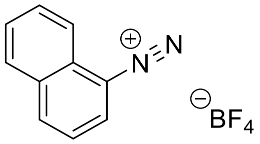

2r

The synthesis was performed according to standard procedure A starting from 1-naphthylamine (1236 mg, 8.63 mmol, 1.0 equiv), aqueous tetrafluoroboric acid (48 w%, 2.9 mL, 22.5 mmol, 2.6 equiv), isoamyl nitrite (1.27 mL, 9.5 mmol, 1.1 equiv), ethanol (8 mL), water (2 mL). 1-Naphtyldiazonium tetrafluoroborate (1724 mg, 7.12 mmol, 83%) was obtained as a dark purple powder.

^{1}H NMR (400 MHz, CD_3CN): δ = 8.97 (dd, *J* = 7.9, 1.2 Hz, 1H), 8.84 (dt, *J* = 8.3, 1.0 Hz, 1H), 8.34 (dd, *J* = 8.3, 1.1 Hz, 1H), 8.27 (dq, *J* = 8.5, 0.9 Hz, 1H), 8.09 (ddd, *J* = 8.4, 7.0, 1.2 Hz, 1H), 8.00 – 7.91 (m, 2H) ppm.

Benzo[*d*][1,3]dioxole-5-diazonium tetrafluoroborate (2s)

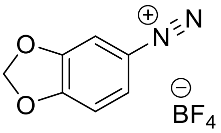

2s

The synthesis was performed according to standard procedure A starting from benzo[*d*][1,3]dioxol-5-amine (1805 mg, 13.2 mmol, 1.0 equiv), aqueous tetrafluoro-

boric acid (48 w%, 4.5 mL, 34.3 mmol, 2.6 equiv), sodium nitrite (908 mg, 13.2 mmol, 1.0 equiv), water (5 mL). Benzo[*d*][1,3]dioxole-5-diazonium tetrafluoroborate (1717 mg, 7.28 mmol, 55%) was obtained as a brown powder.

^{1}H NMR (400 MHz, CD_3CN): δ = 8.21 (dd, *J* = 8.8, 2.2 Hz, 1H), 7.70 (d, *J* = 2.2 Hz, 1H), 7.25 (d, *J* = 8.8 Hz, 1H), 6.36 (s, 2H) ppm.

5-Bromopyridine-3-diazonium tetrafluoroborate (2t)

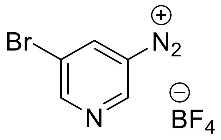

2t

The synthesis was performed according to standard procedure A starting from 5-bromopyridin-3-amine (1000 mg, 5.78 mmol, 1.0 equiv), aqueous tetrafluoroboric acid (48 w%, 2.0 mL, 15.0 mmol, 2.6 equiv), sodium nitrite (399 mg, 5.78 mmol, 1.0 equiv) and water (3 mL). 5-Bromopyridine-3-diazonium tetrafluoroborate (838 mg, 3.08 mmol, 53%) was obtained as yellowish crystals.

^{1}H NMR (400 MHz, CD_3CN): δ = 9.55 (d, *J* = 2.2 Hz, 1H), 9.37 (d, *J* = 2.1, 1H), 8.99 (t, *J* = 2.1 Hz, 1H) ppm.

Thiazole-2-diazonium tetrafluoroborate (2u)

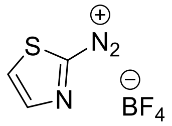

2u

The synthesis was performed according to standard procedure A starting from thiazol-2-amine (1000 mg, 9.99 mmol, 1.0 equiv), aqueous tetrafluoroboric acid (48 w%, 3.4 mL, 26.0 mmol, 2.6 equiv) and sodium nitrite (689 mg, 9.99 mmol, 1.0 equiv). No additional solvent was added. During the recrystallization, gas evolution was observed at temperatures above –10 °C. Therefore, the diazonium salt was used wet and no yield was determined. It was assumed to be 85% pure.

No NMR spectrum was recorded.

1-Phenyl-1*H*-pyrazole-3-diazonium tetrafluoroborate (2v)

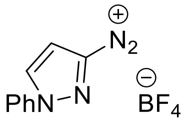

2v

The synthesis was performed according to standard procedure B starting from 1-phenyl-1*H*-pyrazol-4-amine (568 mg, 3.57 mmol, 1.0 equiv), aqueous tetrafluoroboric acid (48 w%, 1.2 mL, 9.28 mmol, 2.6 equiv), isoamyl nitrite (0.52 mL, 3.92 mmol, 1.1 equiv) and water (3 mL). 1-Phenyl-1*H*-pyrazole-3-diazonium tetrafluoroborate (566 mg, 2.19 mmol, 61%) was obtained as off-white crystals.

^{1}H NMR (400 MHz, CD_3CN): δ = 8.58 (d, *J* = 3.0 Hz, 1H), 7.87 (ddd, *J* = 7.7, 3.5, 2.1 Hz, 3H), 7.71 – 7.63 (m, 3H) ppm.

Quinoline-6-diazonium tetrafluoroborate (2w)

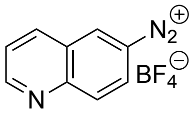

The synthesis was performed according to standard procedure A starting from 6-aminoquinoline (400 mg, 2.77 mmol, 1.0 equiv), aqueous tetrafluoroboric acid (48 w%, 0.9 mL, 7.2 mmol, 2.6 equiv), sodium nitrite (191 mg, 2.77 mmol, 1.0 equiv) and water (1 mL). Quinoline-6-diazonium tetrafluoroborate (674 mg, 1.28 mmol, 46%) was obtained as bright orange crystals.

^{1}H NMR (400 MHz, CD_3CN): δ = 9.32 (q, *J* = 1.9 Hz, 2H), 8.65 (dd, *J* = 8.4, 1.8 Hz, 1H), 8.46 (t, *J* = 1.7 Hz, 2H), 7.86 (dd, *J* = 8.5, 4.3 Hz, 1H) ppm.

2,3,4,5,6-Pentafluorobenzenediazonium tetrafluoroborate (2x)

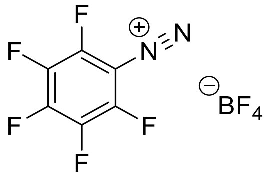

2x

The synthesis was performed according to standard procedure A starting from 2,3,4,5,6-pentafluoroaniline (1003 mg, 5.48 mmol, 1.0 equiv), aqueous tetrafluoroboric acid (48 w%, 1.9 mL, 14.3 mmol, 2.6 equiv), sodium nitrite (378 mg,

5.48 mmol, 1.0 equiv) and ethanol (3 mL). 2,3,4,5,6,-Pentafluorobenzenediazonium tetrafluoroborate (339 mg, 1.20 mmol, 22%) was obtained as beige solid.

^{19}F NMR (376 MHz, CD_3CN): δ = –143.1 (s, 1F), –153.5 (d, *J* = 321 Hz, 2F), –160.3 (s, 1F) ppm.

2-(Methoxycarbonyl)benzenediazonium tetrafluoroborate (9)

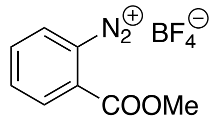

9

The synthesis was performed according to standard procedure B starting from methyl o-anthranilate (1528 mg, 10.1 mmol, 1.00 equiv), aqueous tetrafluoroboric acid (48 w%, 3.4 mL, 26.3 mmol, 2.6 equiv), Methanol (3.0 mL) and isoamyl nitrite (1.49 mL, 11.1 mmol, 1.1 equiv). 2-(Methoxycarbonyl)benzenediazonium tetrafluoroborate (2413 mg, 9.65 mmol, 96%) was obtained as white powder.

^{1}H NMR (400 MHz, CD_3CN): δ = 8.70 (dd, *J* = 8.3, 1.2 Hz, 1H), 8.44 (dd, *J* = 7.9, 1.4 Hz, 1H), 8.37 (td, *J* = 7.8, 1.2 Hz, 1H), 8.17 (ddd, *J* = 8.2, 7.6, 1.4 Hz, 1H), 4.07 (s, 3H) ppm.

4-Ethoxy-3-(1-methyl-7-oxo-3-propyl-6,7-dihydro-1*H*-pyrazolo[4,3-*d*]pyrimidin-5-yl)benzenediazonium tetrafluoroborate (12)

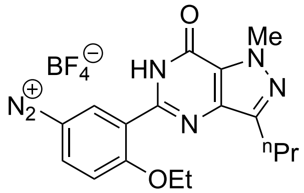

12

The synthesis was performed according to standard procedure B starting from 5-(5-amino-2-ethoxyphenyl)-1-methyl-3-propyl-1,6-dihydro-7*H*-pyrazolo[4,3-*d*]pyrimidin-7-one (458 mg, 1.40 mmol, 1.0 equiv), aqueous tetrafluoroboric acid (48 w%, 0.5 mL, 3.6 mmol, 2.6 equiv), ethanol (0.5 mL) and isoamyl nitrite (0.21 mL, 1.54 mmol, 1.1 equiv). 4-Ethoxy-3-(1-methyl-7-oxo-3-propyl-6,7-dihydro-1*H*-pyrazolo[4,3-*d*]pyrimidin-5-yl)benzenediazonium tetrafluoroborate (459 mg, 1.08 mmol, 77%) was obtained as creme-colored powder.

^{1}H NMR (400 MHz, CD_3CN): δ = 10.43 (s, 1H), 9.04 (d, *J* = 2.8 Hz, 1H), 8.54 (dd, *J* = 9.4, 2.8 Hz, 1H), 7.55 (d, *J* = 9.5 Hz, 1H), 4.50 (q, *J* = 7.0 Hz, 2H), 4.18 (s, 3H), 2.85 (t, *J* = 7.5 Hz, 2H), 1.82 (h, *J* = 7.4 Hz, 2H), 1.53 (t, *J* = 7.0 Hz, 3H), 0.98 (t, *J* = 7.4 Hz, 3H) ppm.

Sulfonimidates

1*H*-Benzo[*d*][1,2,3]triazol-1-yl 4-fluoro-*N*-tritylbenzenesulfonimidate (3a)

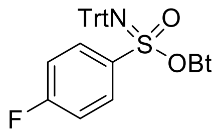

3a

The synthesis was performed according to standard procedure C starting from 4-fluorobenzenediazonium tetrafluoroborate (105 mg, 0.50 mmol, 1.0 equiv), *N*-tritylsulfinylamine (168 mg, 0.55 mmol, 1.1 equiv), HOBt hydrate (100 mg, 0.65 mmol, 1.3 equiv), *N*-methylpiperidine (67 µL, 0.55 mmol, 1.1 equiv). The crude product was purified by flash column chromatography (SiO_2, 3 x 30cm, pentane/DCM/Et_2O 8:1:1). The title compound (208 mg, 0.39 mmol, 78%) was obtained as white foam.

^{1}H NMR (400 MHz, $CDCl_3$): δ = 8.09 – 8.02 (m, 2H), 7.92 (dd, *J* = 7.4, 1.6 Hz, 1H), 7.68 – 7.60 (m, 6H), 7.48 – 7.20 (m, 13H), 6.69 – 6.63 (m, 1H) ppm. **^{13}C{^{1}H} NMR** (151 MHz, $CDCl_3$): δ = 166.3 (d, *J* = 258.0 Hz), 146.0, 142.8, 132.0 (d, *J* = 3.0 Hz), 131.7 (d, *J* = 9.7 Hz), 129.1, 128.3, 128.0, 127.4, 124.6, 119.6, 116.8 (d, *J* = 23.0 Hz), 131.7 (d, *J* = 9.7 Hz), 129.1, 128.3, 128.0, 127.4, 124.6, 119.6, 116.8 (d, *J* = 23.0 Hz), 110.0, 75.0 ppm. **^{19}F NMR** (376 MHz, $CDCl_3$): δ = –101.6 (d, *J* = 7.7 Hz) ppm. **MS** (ESI): *m*/*z* (%) = 573 (20), 557 $[M+Na]^+$ (100), 416 (94), 284 (17), 258 (50), 243 (70), 165 (17). **HRMS** (ESI): $[M+Na]^+$ = $[C_{31}H_{23}O_2N_4FNaS]^+$, *m*/*z* calculated: 557.1418, found 557.1411. **IR** (ATR): $\tilde{\nu}$ = 3060, 2668, 2323, 2160, 2113, 1957, 1901, 1809, 1594, 1489, 1445, 1361, 1308, 1280, 1201, 1078, 1030, 998, 935, 897, 846, 743, 697 cm^{-1}. **TLC**: R_f (SiO_2, pentane/DCM/Et_2O 8:1:1) = 0.15.

Up-Scaling

The scaled-up synthesis was performed according to standard procedure C except of some minor changes, starting from 4-fluorobenzenediazonium tetrafluoroborate (2099 mg, 10.0 mmol, 1.00 equiv), *N*-tritylsulfinylamine (3359 mg, 11.0 mmol, 1.10

equiv), HOBt hydrate (1991 mg, 13.0 mmol, 1.30 equiv) and *N*-methylpiperidine (1.34 mL, 11.0 mmol, 1.10 equiv). Instead of using the freeze-pump-thaw procedure as utilized in smaller scales, DMC (ca. 750 mL) was degassed by purging with nitrogen for 45 minutes. 1*H*-Benzo[*d*][1,2,3]triazol-1-yl 4-fluoro-*N*-tritylbenzenesulfonimidate was obtained as a yellowish foam (98% purity, 3965 mg, 7.3 mmol, 73%).

1*H*-Benzo[*d*][1,2,3]triazol-1-yl *N*-tritylbenzenesulfonimidate (3b)

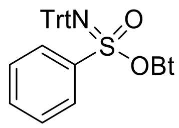

3b

The synthesis was performed according to standard procedure C starting from benzenediazonium tetrafluoroborate (96 mg, 0.50 mmol, 1.0 equiv), *N*-tritylsulfinylamine (168 mg, 0.55 mmol, 1. equiv), HOBt hydrate (100 mg, 0.65 mmol, 1.3 equiv) and *N*-methylpiperidine (67 µL, 0.55 mmol, 1.1 equiv). The crude product was purified by flash column chromatography (SiO_2, 3 x 30 cm, pentane/DCM/Et_2O 8:1:1). The title compound (230 mg, 0.45 mmol, 89%) was obtained as white foam.

^{1}H NMR (600 MHz, $CDCl_3$): δ = 8.04 (d, *J* = 8.0 Hz, 2H), 7.92 – 7.88 (m, 1H), 7.72 (t, *J* = 7.5 Hz, 1H), 7.69 – 7.61 (m, 6H), 7.58 (t, *J* = 7.8 Hz, 2H), 7.42 (t, *J* = 7.7 Hz, 6H), 7.37 (t, *J* = 7.3 Hz, 3H), 7.29 (tt, *J* = 7.2, 5.8 Hz, 2H), 6.63 – 6.59 (m, 1H) ppm. **^{13}C{^{1}H} NMR** (151 MHz, $CDCl_3$): δ = 146.2, 142.8, 136.2, 134.5, 129.5, 129.2, 128.7, 128.3, 127.9, 127.8, 127.3, 124.5, 119.6, 110.0, 74.9 ppm. **MS** (ESI): *m*/*z* (%) = 539 $[M+Na]^+$ (12), 398 (10), 258 (100), 243 (62), 165 (26). **HRMS** (ESI): $[M+Na]^+$ = $[C_{31}H_{24}O_2N_4NaS]^+$, *m*/*z* calculated 539.1512, found 539.1505. **IR** (ATR): $\tilde{\nu}$ = 3061, 2651, 2312, 2158, 2119, 2073, 2009, 1964, 1907, 1783, 1589, 1489, 1445, 1364, 1285, 1238, 1203, 1154, 1080, 1033, 1004, 939, 898, 837, 744, 700, 675 cm^{-1}. **TLC:** R_f (SiO_2, pentane/DCM/Et_2O 8:1:1) = 0.25.

1*H*-Benzo[*d*][1,2,3]triazol-1-yl 4-methyl-*N*-tritylbenzenesulfonimidate (3c)

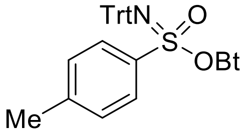

3c

The synthesis was performed according to standard procedure C starting from 4-methylbenzenediazonium tetrafluoroborate (103 mg, 0.50 mmol, 1.0 equiv), *N*-

tritylsulfinylamine (168, 0.55 mmol, 1.1 equiv), HOBt hydrate (100 mg, 0.65 mmol, 1.3 equiv) and *N*-methylpiperidine (67 µL, 0.55 mmol, 1.1 equiv). The crude product was purified by flash column chromatography (SiO_2, 3 x 30 cm, pentane/DCM/Et_2O 6:1:1). 1*H*-Benzo[*d*][1,2,3]triazol-1-yl 4-methyl-*N*-tritylbenzenesulfonimidate (210 mg, 0.40 mmol, 79%) was obtained as a white foam.

^{1}H NMR (600 MHz $CDCl_3$): δ = 7.90 – 7.84 (m, 3H), 7.63 – 7.57 (m, 6H), 7.41-7.30 (m, 11H), 7.26 (ddd, *J* = 11.8, 8.1, 6.5 Hz, 2H), 6.61 – 6.57 (m, 1H), 2.46 (s, 3H) ppm. **^{13}C{^{1}H} NMR** (151 MHz, $CDCl_3$): δ = 146.3, 145.8, 142.9, 133.2, 130.1, 129.2, 128.8, 128.4, 127.9, 127.8, 127.3, 124.5, 119.6, 110.2, 74.8, 21.8 ppm. **MS** (ESI): *m*/*z* (%) = 602 (66), 553 $[M+Na]^+$ (12), 412 (12), 258 (100), 243 (45). **HRMS** (ESI): $[M+Na]^+$ $[C_{32}H_{26}O_2N_4NaS]^+$, *m*/*z* calculated 553.1669, found 553.1670. **IR** (ATR): $\tilde{\nu}$ = 3060, 2923, 2321, 2168, 2084, 1976, 1916, 1809, 1592, 1488, 1443, 1400, 1299, 1203, 1179, 1081, 1033, 938, 900, 816, 741, 696, 660 cm^{-1}. **TLC:** R_f (SiO_2, pentane/DCM/Et_2O 6:1:1) = 0.23.

1*H*-Benzo[*d*][1,2,3]triazol-1-yl 3-methyl-*N*-tritylbenzenesulfonimidate (3d)

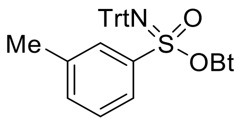

3d

The synthesis was performed according to standard procedure C starting from 3-methylbenzenediazonium tetrafluoroborate (103 mg, 0.50 mmol, 1.0 equiv), *N*-tritylsulfinylamine (168 mg, 0.55 mmol, 1.1 equiv), HOBt hydrate (100 mg, 0.65 mmol, 1.3 equiv) and *N*-methylpiperidine (67 µL, 0.55 mmol, 1.1 equiv). The crude product was purified by flash column chromatography (SiO_2, 3 x 30 cm, pentane/DCM/Et_2O 8:1:1). The product (225 mg, 0.42 mmol, 85%) was obtained as a white foam.

^{1}H NMR (400 MHz, $CDCl_3$): δ = 7.89 – 7.83 (m, 1H), 7.80 (d, *J* = 7.9 Hz, 1H), 7.76 (s, 1H), 7.62 – 7.55 (m, 6H), 7.50 (d, *J* = 7.6 Hz, 1H), 7.45 (d, *J* = 7.7 Hz, 1H), 7.42 – 7.29 (m, 9H), 7.29 – 7.22 (m, 2H), 6.58 – 6.52 (m, 1H), 2.41 (s, 3H) ppm. **^{13}C{^{1}H} NMR** (101 MHz, $CDCl_3$): δ = 146.3, 142.9, 139.8, 136.2, 135.4, 129.3, 129.3, 129.0, 128.4, 128.0 127.8, 127.8, 127.3, 126.1, 124.5, 119.7, 110.1, 74.9, 21.5 ppm. **MS** (ESI): *m*/*z* (%) = 553 (3) $[M+Na]^+$, 389 (11, 323 (100), 258 (36), 243 (30), 169 (27). **HRMS** (ESI): $[M+Na]^+$ = $[C_{32}H_{26}O_2N_4NaS]^+$, *m*/*z* calculated 553.1669, found 553.1679. **IR** (ATR): $\tilde{\nu}$ = 3060, 2321, 2159, 2004, 1956, 1807, 1596, 1487, 1444,

1360, 1282, 1191, 1079, 1034, 997, 902, 857, 741, 696 cm^{-1}. **TLC**: R_f (pentane/DCM/Et_2O 6:1:1) = 0.30.

1*H*-Benzo[*d*][1,2,3]triazol-1-yl 2-methyl-*N*-tritylbenzenesulfonimidate (3e)

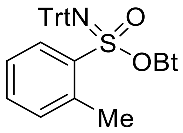

3e

The synthesis was performed according to standard procedure C starting from 2-toluyldiazonium tetrafluoroborate (103 mg, 0.50 mmol, 1.0 equiv), *N*-tritylsulfinylamine (168 mg, 0.55 mmol, 1.1 equiv), HOBt hydrate (100 mg, 0.65 mmol, 1.3 equiv) and *N*-methylpiperidine (67 µL, 0.55 mmol, 1.1 equiv). The crude product was purified by flash column chromatography (SiO_2, 3 x 30 cm, pentane/DCM/Et_2O 8:1:1). The title compound (265 mg, 0.31 mmol, 61%) was obtained as white foam.

^{1}H NMR (400 MHz, $CDCl_3$): δ = 7.84 (dd, *J* = 8.3, 3.4 Hz, 2H), 7.60 (d, *J* = 7.6 Hz, 6H), 7.58 – 7.48 (m, 2H), 7.39 (t, *J* = 7.5 Hz, 6H), 7.35 – 7.20 (m, 6H), 6.65 (d, *J* = 8.0 Hz, 1H), 3.06 (s, 3H) ppm. **^{13}C{^{1}H} NMR** (101 MHz, $CDCl_3$): δ = 146.1, 142.8, 139.5, 135.7, 134.2, 133.3, 129.6, 129.3, 128.5, 127.9, 127.2, 126.3, 124.4, 119.6, 110.2, 75.7, 21.5 ppm. **MS** (ESI): *m*/*z* (%) = 553 $[M+Na]^+$ (2), 412 (4), 258 (100), 180 (57). **HRMS** (ESI): $[M+Na]^+$ = $[C_{32}H_{26}O_2N_4NaS]^+$, calculated 553.1669, found 553.1662. **IR** (ATR): $\tilde{\nu}$ = 3060, 2322, 2159, 2033, 1996, 1954, 1816, 1595, 1488, 1445, 1347, 1280, 1188, 1079, 1033, 999, 904, 848, 807, 740, 697 cm^{-1}. **TLC**: R_f (pentane/DCM/Et_2O 6:1:1) = 0.30

1*H*-Benzo[*d*][1,2,3]triazol-1-yl 4-chloro-*N*-tritylbenzenesulfonimidate (3f)

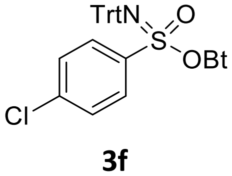

3f

The synthesis was performed according to standard procedure C starting from 4-chlorobenzenediazonium tetrafluoroborate (103 mg, 0.50 mmol, 1.0 equiv), *N*-tritylsulfinylamine (168 mg, 0.55 mmol, 1.1 equiv), HOBt hydrate (100 mg, 0.65 mmol, 1.3 equiv) and *N*-methylpiperidine (67 µL, 0.55 mmol, 1.1 equiv). The crude product was purified by flash column chromatography (SiO_2, 3 x 30 cm,

pentane/DCM/Et_2O 10:1:1). The first column (SiO_2, 3 x 30 cm, pentane/DCM/Et_2O 8:1:1) yielded an impure product. The title compound (155 mg, 0.28 mmol, 55%) was obtained as white foam.

^{1}H NMR (600 MHz, $CDCl_3$): δ = 7.98 – 7.91 (m, 2H), 7.92 – 7.87 (m, 1H), 7.62 – 7.56 (m, 6H), 7.56 – 7.53 (m, 2H), 7.43 – 7.38 (m, 6H), 7.39 – 7.32 (m, 3H), 7.30 (dddd, *J* = 15.0, 8.2, 7.0, 1.3 Hz, 2H), 6.63 – 6.58 (m, 1H) ppm. **^{13}C{^{1}H} NMR** (151 MHz, $CDCl_3$): δ = 146.0, 142.9, 141.4, 134.8, 130.2, 129.8, 129.2, 128.4, 128.0, 128.0, 127.4, 124.6, 119.7, 110.0, 75.1 ppm. **MS** (ESI): *m*/*z* (%) = 258 (100), 243 (11), 180 (8). **HRMS** (ESI): $[M+Na]^+$ = $[C_{31}H_{23}ClN_4O_2NaS]^+$, *m*/*z* calculated 573.1123, found 573.1137. **IR** (ATR): $\tilde{\nu}$ = 3062, 2635, 2324, 2163, 2107, 1982, 1910, 1785, 1579, 1488, 1443, 1364, 1280, 1200, 1084, 1009, 901, 827, 745, 696 cm^{-1}. **TLC**: R_f (SiO_2, pentane/DCM/Et_2O 8:1:1) = 0.38.

1*H*-Benzo[*d*][1,2,3]triazol-1-yl 4-bromo-*N*-tritylbenzenesulfonimidate (3g)

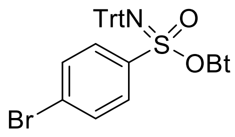

3g

The synthesis was performed according to standard procedure C starting from 4-bromodiazonium tetrafluoroborate (135 mg, 0.50 mmol, 1.0 equiv), *N*-tritylsulfinylamine (168 mg, 0.55 mmol, 1.1 equiv), HOBt hydrate (100 mg, 0.65 mmol, 1.3 equiv) and *N*-methylpiperidine (67 µL, 0.55 mmol, 1.1 equiv). The crude product was purified by flash column chromatography (SiO_2, 3 x 30 cm, pentane/DCM/Et_2O 12:1:1). The first column (SiO_2, 3 x 30 cm, pentane/DCM/Et_2O 8:1:1) yielded an impure product. The title compound (165 mg, 0.28 mmol, 55%) was obtained as a white foam.

^{1}H NMR (600 MHz, $CDCl_3$): δ = 7.91 – 7.88 (m, 1H), 7.87 (d, *J* = 8.5 Hz, 2H), 7.73 – 7.70 (m, 2H), 7.61 – 7.56 (m, 6 H), 7.43 -7.38 (m, 6H), 7.38 -7.34 (m, 3H), 7.34 -7.27 (m, 2H), 6.62 – 6.58 (m, 1H) ppm. **^{13}C{^{1}H} NMR** (151 MHz, $CDCl_3$): δ = 146.0, 142.9, 135.4, 132.8, 130.3, 130.1, 129.2, 128.4, 128.0, 127.4, 124.6, 119.7, 110.0, 75.2 ppm. **MS** (ESI): *m*/*z* (%) = 617 $[M+Na]^+$ (8), 478 (5), 258 (100), 228 (36), 165 (32). **HRMS** (ESI): $[M+Na]^+$ = $[C_{31}H_{23}O_2N_4BrNaS]^+$, *m*/*z* calculated 617.0617, found 617.0621. **IR** (ATR): $\tilde{\nu}$ = 3060, 2753, 2339, 2230, 2186, 2072, 1992, 1916, 1788, 1571, 1489, 1443, 1365, 1278, 1198, 1072, 1005, 899, 822, 740, 698 cm^{-1}. **TLC**: R_f (SiO_2, pentane/DCM/Et_2O 8:1:1) = 0.43.

1*H*-Benzo[*d*][1,2,3]triazol-1-yl 4-iodo-*N*-tritylbenzenesulfonimidate (3h)

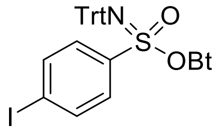

3h

The synthesis was performed according to standard procedure C starting from 4-iodobenzenediazonium tetrafluoroborate (159 mg, 0.50 mmol, 1.0 equiv), *N*-tritylsulfinylamine (168 mg, 0.55 mmol, 1.1 equiv), HOBt hydrate (100 mg, 0.65 mmol, 1.3 equiv) and *N*-methylpiperidine (67 µL, 0.55 mmol, 1.1 equiv). The crude product was purified by flash column chromatography twice. The first column (SiO_2, 3 x 30 cm, pentane/DCM/Et_2O 12:1:1) yielded an impure product. The second column (SiO_2, 2.5 x 20 cm, pentane/DCM/MTBE 24:2:1) yielded the title compound (148 mg, 0.23 mmol, 46%) as white solid.

^{1}H NMR (600 MHz, $CDCl_3$): δ = 7.95 – 7.90 (m, 2H), 7.89 – 7.84 (m, 1H), 7.68 – 7.63 (m, 2H), 7.54 – 7.48 (m, 6H), 7.40 – 7.30 (m, 9H), 7.29 – 7.26 (m, 2H), 6.57 – 6.49 (m, 1H) ppm. **^{13}C{^{1}H} NMR** (151 MHz, $CDCl_3$): δ = 146.1, 143.0, 138.9, 136.2, 130.1, 129.2, 128.4, 128.1, 127.5, 124.7, 119.8, 110.1, 103.0, 75.2 ppm. **HRMS** (ESI): $[M+Na]^+$ = $[C_{31}H_{23}O_2N_4INaS]^+$, *m*/*z* calculated 665.0479, found 665.0481. **IR** (ATR): $\tilde{\nu}$ = 3063, 2656, 2314, 2180, 2080, 1994, 1916, 1812, 1564, 1483, 1412, 1275, 1210, 1086, 1003, 940, 896, 824, 738, 699 cm^{-1}. **TLC**: R_f (SiO_2, pentane/DCM/MTBE 24:2:1) = 0.15.

Methyl-4-{*S*-[(1*H*-benzo[*d*][1,2,3]triazol-1-yl]oxy}-*N*-tritylsulfonimidoyl)benzoate (3i)

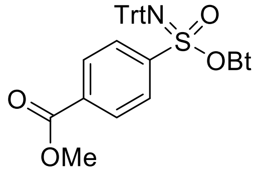

3i

The synthesis was performed according to standard procedure C starting from 4-(methoxycarbonyl)benzenediazonium tetrafluoroborate (135 mg, 0.50 mmol, 1.00 equiv), *N*-tritylsulfinylamine (168 mg, 0.55 mmol, 1.1 equiv), HOBt hydrate (100 mg, 0.65 mmol, 1.3 equiv) and *N*-methylpiperidine (67 µL, 0.55 mmol, 1.1 equiv). The crude product was purified by flash column chromatography (SiO_2, 3 x 30 cm, pentane/DCM/Et_2O 5:1:1). The title compound (243 mg, 0.41 mmol, 82%) was

obtained as white foam. Considering that the product remained impure the yield was corrected to 78%.

^{1}H NMR (600 MHz, $CDCl_3$): δ = 8.28 – 8.20 (m, 2H), 8.08 (dd, *J* = 8.6, 2.0 Hz, 2H), 7.92 – 7.83 (m, 1H), 7.56 (dt, *J* = 8.6, 2.0 Hz, 6H), 7.38 (dd, *J* = 8.5, 6.7 Hz, 6 H), 7.36 – 7.32 (m, 3H), 7.30 – 7.27 (m, 2H), 6.59 – 6.53 (m, 1H), 3.98 (d, *J* = 2.0 Hz, 3H) ppm. **^{13}C{^{1}H} NMR** (151 MHz, $CDCl_3$): δ = 165.4, 146.0, 142.9, 140.2, 135.4, 130.6, 129.2, 128.9, 128.4, 128.1, 128.0, 127.5, 124.7, 119.0, 110.0, 75.3, 52.9 ppm. **HRMS** (ESI): $[M+Na]^+$ = $[C_{33}H_{26}O_4N_4NaS]^+$, *m*/*z* calculated 597.1567, found 597.1567. **IR** (ATR): $\tilde{\nu}$ = 3058, 2954, 2288, 2208, 2164, 2032, 1983, 1943, 1770, 1731, 1595, 1490, 1441, 1363, 1279, 1198, 1108, 1083, 1014, 962, 898, 856, 832, 741, 701 cm^{-1}.

1*H*-Benzo[*d*][1,2,3]triazol-1-yl 4-cyano-*N*-tritylbenzenesulfonimidate (3j)

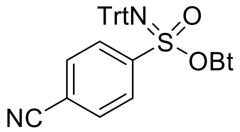

3j

The synthesis was performed according to standard procedure C starting from (4-cyano)benzenediazonium tetrafluoroborate (108 mg, 0.50 mmol, 1.0 equiv), *N*-tritylsulfinylamine (168 mg, 0.55 mmol, 1.1 equiv), HOBt hydrate (100 mg, 0.65 mmol, 1.3 equiv) and *N*-methylpiperidine (67 µL, 0.55 mmol, 1.1 equiv). The crude product was purified twice by flash column chromatography (first column: SiO_2, 3 x 30 cm, pentane/DCM/Et_2O 4:1:1; second column: SiO_2, 2.5 x 20 cm, pentane/DCM/MTBE 8:2:1). The title compound (213 mg, 0.39 mmol, 79%) was obtained as white foam.

^{1}H NMR (400 MHz, $CDCl_3$): δ = 8.10 (d, *J* = 8.2 Hz, 2H), 7.87 (d, *J* = 7.9 Hz, 3H), 7.53 – 7.42 (m, 7H), 7.36 (d, *J* = 7.5 Hz, 9 H), 7.31 – 7.27 (m, 1H), 6.56 – 6.49 (m, 1H) ppm. **^{13}C{^{1}H} NMR** (151 MHz, $CDCl_3$): δ = 145.7, 142.8, 140.5, 133.1, 129.4, 129.0, 128.3, 128.2, 128.0, 127.5, 124.8, 119.8, 118.1, 117.0, 109.7, 75.4 ppm. **HRMS** (ESI): $[M+Na]^+$ = $[C_{32}H_{23}O_2N_5NaS]^+$, *m*/*z* calculated 564.1465, found 564.1464. **IR** (ATR): $\tilde{\nu}$ = 3061, 2326, 2235, 2167, 2096, 2023, 1974, 1898, 1806, 1595, 1488, 1444, 1368, 1283, 1203, 1078, 1032, 937, 897, 837, 744, 699, 675 cm^{-1}. **TLC**: R_f (SiO_2, pentane/DCM/MTBE 8:2:1) = 0.15.

1*H*-Benzo[*d*][1,2,3]triazol-1-yl 4-nitro-*N*-tritylbenzenesulfonimidate (3k)

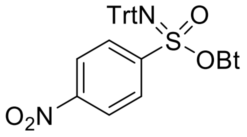

3k

The synthesis was performed according to standard procedure C starting from 4-nitrobenzenediazonium tetrafluoroborate (118 mg, 0.50 mmol, 1.0 equiv), *N*-tritylsulfinylamine (168 mg, 0.55 mmol, 1.1 equiv), HOBt hydrate (100 mg, 0.65 mmol, 1.3 equiv) and *N*-methylpiperidine (67 µL, 0.55 mmol, 1.1 equiv). The crude product was purified by flash column chromatography (SiO_2, 3 x 30 cm, pentane/DCM/Et_2O 8:1:1). The title compound (212 mg, 0.38 mmol, 75%) was obtained as a yellow foam.

^{1}H NMR (600 MHz, $CDCl_3$): δ = 8.40 (d, *J* = 8.9 Hz, 2H), 8.23 – 8.18 (m, 2H), 7.91 – 7.86 (m, 1H), 7.58 -7.50 (m, 6H), 7.43 -7.28 (m, 11H), 6.60 – 6.56 (m, 1H) ppm. **^{13}C{^{1}H} NMR** (151 MHz, $CDCl_3$): δ = 151.1, 145.7, 142.9, 142.0, 130.2, 129.1, 128.4, 128.3, 128.1, 127.6, 124.9, 124.6, 119.8, 109.8, 75.6 ppm. **MS** (ESI): *m*/*z* (%) = 397 (3), 258 (100), 242 (14), 180 (10), 142 (4). **HRMS** (ESI): $[M+Na]^+$ = $[C_{31}H_{23}N_5O_4NaS]^+$, *m*/*z* calculated 584.1363, found 584.1372. **IR** (ATR): $\tilde{\nu}$ = 3063, 2864, 2680, 2325, 2165, 2083, 925, 1799, 1687, 1602, 1530, 1491, 1444, 1351, 1197, 1079, 1004, 941, 899, 851, 742, 693 cm^{-1}. **TLC**: R_f (SiO_2, pentane/DCM/Et_2O 4:1:1) = 0.46.

1*H*-Benzo[*d*][1,2,3]triazol-1-yl 4-methoxy-*N*-tritylbenzenesulfonimidate (3l)

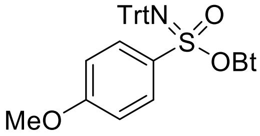

3l

The synthesis was performed according to standard procedure C starting from (4-methoxy)benzenediazonium tetrafluoroborate (111 mg, 0.50 mmol, 1.0 equiv), *N*-tritylsulfinylamine (168 mg, 0.55, 1.1 equiv), HOBt hydrate (100 mg, 0.65 mmol, 1.3 equiv) and *N*-methylpiperidine (67µL, 0.55 mmol, 1.1 equiv). The crude product was purified by flash column chromatography (SiO_2, 3 x 30 cm, pentane/DCM/MTBE 16:4:1). The title compound (184 mg, 0.34 mmol, 67%) was obtained as white solid.

^{1}H NMR (600 MHz, $CDCl_3$): δ = 7.93 – 7.82 (m, 3H), 7.58 – 7.52 (m, 6H), 7.39 – 7.29 (m, 9H), 7.28 -7.23 (m, 2H), 7.03 – 6.97 (m, 2H), 6.59 – 6.54 (m, 1H), 3.89 (s, 3H) ppm. **^{13}C{^{1}H} NMR** (151 MHz, $CDCl_3$): δ = 164.6, 146.4, 143.0, 131.2, 129.3, 128.0, 127.3, 124.5. 119.7, 114.8, 110.3, 74.8, 55.9 ppm. **MS** (EI): *m*/*z* (%) = 257 (100), 243 (66), 180 (77), 165 (56), 77 (38). **HRMS** (ESI): $[M+Na]^+$ = $[C_{32}H_{26}O_3N_4NaS]^+$, *m*/*z* calculated 569.1618, found 569.1614. **IR** (ATR): $\tilde{\nu}$ = 3061, 2940, 2844, 2247, 2163, 1903, 1771, 1591, 1492, 1444, 1362, 1264, 1204, 1089, 1025, 906, 833, 803, 733, 700, 675 cm^{-1}. **TLC**: R_f (SiO_2, pentane/DCM/Et_2O 8:1:1) = 0.23.

1*H*-Benzo[*d*][1,2,3]triazol-1-yl 4-(methylthio)-*N*-tritylbenzenesulfonimidate (3m)

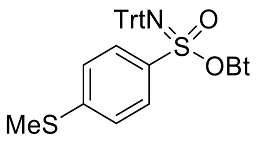

3m

The synthesis was performed according to standard procedure C starting from (4-methylthio)benzenediazonium tetrafluoroborate (119 mg, 0.50 mmol, 1.0 equiv), *N*-tritylsulfinylamine (168 mg, 0.55 mmol, 1.1 equiv), HOBt hydrate (100 mg, 0.65 mmol, 1.3 equiv) and *N*-methylpiperidine (67 µL, 0.55 mmol, 1.1 equiv). The crude product was purified by flash column chromatography (SiO_2, 3 x 30 cm, pentane/DCM/MTBE 16:4:1). The title compound (204 mg, 0.36 mmol, 73%) was obtained as white foam.

^{1}H NMR (600 MHz, $CDCl_3$): δ = 7.87 – 7.83 (m, 1H), 7.83 – 7.79 (m, 2H), 7.57 – 7.51 (m, 6H), 7.38 – 7.34 (m, 6H), 7.34 – 7.30 (m, 5H), 7.29 – 7.22 (m, 2H), 6.58 – 6.52 (m, 1H), 2.53 (s, 3H) ppm. **^{13}C{^{1}H} NMR** (151 MHz, $CDCl_3$): δ = 148.8, 146.3, 143.0, 131.5, 129.3, 129.1, 128.5, 128.0, 127.9, 127.4, 125.4, 124.6, 119.7, 110.2, 74.9, 14.8 ppm. **MS** (EI): *m*/*z* (%) = 257 (90), 243 (100), 180 (60), 165 (62), 77 (33). **HRMS** (ESI): $[M+Na]^+$ = $[C_{33}H_{26}O_2N_4NaS_2]^+$, *m*/*z* calculated 585.1389, found 585.1384. **IR** (ATR): $\tilde{\nu}$ = 3060, 2923, 2245, 2161, 1956, 1815, 1575, 1488, 1442, 1362, 1280, 1203, 1183, 1080, 1033, 1006, 905, 815, 734, 699 cm^{-1}. **TLC**: R_f (SiO_2, pentane/DCM/Et_2O 8:1:1) = 0.34.

1*H*-Benzo[*d*][1,2,3]triazol-1-yl 4-(trifluoromethyl)-*N*-tritylbenzenesulfonimidate (3n)

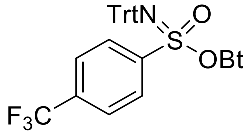

3n

The synthesis was performed according to standard procedure C starting from (4-trifluoromethyl)benzenediazonium tetrafluoroborate (130 mg, 0.50 mmol, 1.0 equiv), *N*-tritylsulfinylamine (168 mg, 0.55 mmol, 1.1 equiv), HOBt hydrate (100 mg, 0.65 mmol, 1.3 equiv) and *N*-methylpiperidine (67 µL, 0.55 mmol, 1.1 equiv). The crude product was purified by flash column chromatography (SiO_2, 3 x 30 cm, pentane/DCM/Et_2O 8:1:1). The title compound (215 mg, 0.37 mmol, 74%) was obtained as white foam.

^{1}H NMR (600 MHz, $CDCl_3$): δ = 8.12 (d, *J* = 8.3 Hz, 2H), 7.90 – 7.81 (m, 3H), 7.51 (ddd, *J* = 8.9, 4.9, 2.5 Hz, 6H), 7.42 – 7.31 (m, 9H), 7.31 – 7.26 (m, 2H), 6.51 (dt, *J* = 5.2, 3.2 Hz, 1H) ppm. **^{13}C{^{1}H} NMR** (151 MHz, $CDCl_3$): δ = 146.0, 143.0, 140.0, 136.0 (q, *J* = 33.1 Hz), 129.5, 129.2, 128.4, 128.3, 128.1, 127.6, 126.7, 124.8, 119.9, 109.9, 75.4 ppm. **^{19}F NMR** (564 MHz, $CDCl_3$): δ = –63.2 ppm. **HRMS** (ESI): $[M+Na]^+$ = $[C_{32}H_{23}O_2N_4F_3NaS]^+$, *m*/*z* calculated 607.1386, found 607.1386. **IR** (ATR): $\tilde{\nu}$ = 3060, 2322, 2094, 2025, 1943, 1741, 1598, 1490, 1444, 1370, 1320, 1174, 1133, 1063, 1013, 938, 898, 841, 744, 701 cm^{-1}. **TLC**: R_f (SiO_2, pentane/DCM/Et_2O 8:1:1) = 0.15.

1*H*-Benzo[*d*][1,2,3]triazol-1-yl 4-(pentafluoro-λ^6-sulfaneyl)-*N*-tritylbenzene-sulfonimidate (3o)

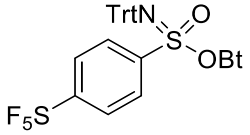

3o

The synthesis was performed according to standard procedure C starting from 4-(pentafluoro-λ^6-sulfaneyl)benzenediazonium tetrafluoroborate (159 mg, 0.50 mmol, 1.0 equiv), *N*-tritylsulfinylamine (168 mg, 0.55 mmol, 1.1 equiv), HOBt hydrate (100 mg, 0.65 mmol, 1.3 equiv) and *N*-methylpiperidine (67 µL, 0.55 mmol, 1.1 equiv). The crude product was purified by flash column chromatography (SiO_2, 3 x 30 cm, pentane/DCM/Et_2O 8:1:1). The title compound (217 mg, 0.34 mmol, 68%) was

obtained as white foam. Since the product contained 5% of an impurity, the yield was corrected to 65%.

^{1}H NMR (600 MHz, $CDCl_3$): δ = 8.10 (d, *J* = 8.6 Hz, 2H), 8.00 – 7.94 (m, 2H), 7.90 – 7.84 (m, 1H), 7.50 (dd, *J* = 8.2, 1.5 Hz, 6H), 7.41 – 7.32 (m, 9H), 7.29 (ddd, *J* = 6.8, 3.0, 1.1 Hz, 2H), 6.52 – 6.47 (m, 1H) ppm. **^{13}C{^{1}H} NMR** (151 MHz, $CDCl_3$): δ =145.9, 143.0, 139.0, 129.6, 129.2, 128.4, 128.3, 128.2, 127.6, 127.5, 124.9, 119.9, 109.9, 75.6 ppm. **^{19}F NMR** (564 MHz, $CDCl_3$): δ = 81.3 (p, *J* = 150.8, 150.3 Hz), 62.6 (d, *J* = 150.7 Hz) ppm. **HRMS** (ESI): $[M+Na]^+$ = $[C_{31}H_{23}O_2N_4F_5NaS_2]^+$, *m*/*z* calculated 665.10748, found 665.10754. **IR** (ATR): $\tilde{\nu}$ = 3061, 2323, 2204, 2052, 2052, 2000, 1943, 1744, 1594, 1489, 1444, 1372, 1286, 1204, 1079, 1034, 1001, 939, 899, 839, 738, 699, 668 cm^{-1}. **TLC** R_f (SiO_2, pentane/DCM/Et_2O 8:1:1) = 0.35.

1*H*-Benzo[*d*][1,2,3]triazol-1-yl 3-acetyl-*N*-tritylbenzenesulfonimidate (3p)

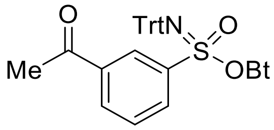

3p

The synthesis was performed according to standard procedure C starting from 3-acetylbenzenediazonium tetrafluoroborate (117 mg, 0.50 mmol, 1.0 equiv), *N*-tritylsulfinylamine (168 mg, 0.55 mmol, 1.1 equiv), HOBt hydrate (100 mg, 0.65 mmol, 1.3 equiv) and *N*-methylpiperidine (67 µL, 0.55 mmol, 1.1 equiv). The crude product was purified by flash column chromatography (SiO_2, 3 x 30 cm, pentane/DCM/Et_2O 4:1:1). The title compound (81 mg, 0.15 mmol, 29%) was obtained as brownish foam.

^{1}H NMR (400 MHz, $CDCl_3$): δ = 8.46 (s, 1H), 8.30 (d, *J* = 7.8 Hz, 1H), 8.21 (d, *J* = 7.9 Hz, 1H), 7.90 – 7.82 (m, 1H), 7.71 (t, *J* = 7.9 Hz, 1H), 7.62 – 7.47 (m, 6H), 7.46 – 7.32 (m, 9H), 7.32 – 7.27 (m, 2H), 6.62 – 6.51 (m, 1H), 2.58 (s, 3H) ppm. **^{13}C{^{1}H} NMR** (101 MHz, $CDCl_3$): δ = 195.8, 146.0, 142.9, 138.0, 137.4, 133.8, 132.8, 130.1, 129.2, 128.8, 128.4, 128.2, 128.1, 127.5, 124.8, 119.8, 110.0, 75.3, 26.7 ppm. **HRMS** (ESI): $[M+Na]^+$ = $[C_{33}H_{26}O_3N_4NaS]^+$, *m*/*z* calculated 581.1618, found 581.1609. **IR** (ATR): $\tilde{\nu}$ = 3856, 3283, 3063, 2923, 2659, 2323, 2067, 1990, 1912, 1738, 1689, 1594, 1491, 1445, 1357, 1329, 1256, 1200, 1153, 1086, 1024, 963, 902, 870, 802, 745, 697 cm^{-1}. **TLC:** R_f (SiO_2, pentane/DCM/Et_2O 4:1:1) = 0.22.

1*H*-Benzo[*d*][1,2,3]triazol-1-yl *N*-tritylnaphthalene-1-sulfonimidate (3r)

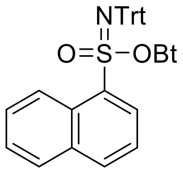

3r

The synthesis was performed according to standard procedure C starting from 1-naphtyldiazonium tetrafluoroborate (121 mg, 0.50 mmol, 1.0 equiv), *N*-tritylsulfinylamine (168 mg, 0.55 mmol, 1.1 equiv), HOBt hydrate (100 mg, 0.65 mmol, 1.3 equiv) and *N*-methylpiperidine (67 µL, 0.55 mmol, 1.1 equiv). The crude product was purified by flash column chromatography (SiO_2, 3 x 30 cm, pentane/DCM/MTBE 12:2:1). The title compound (203 mg, 0.36 mmol, 72%) was obtained as beige foam.

^{1}H NMR (600 MHz, $CDCl_3$): δ = 9.31 (d, *J* = 8.6 Hz, 1H), 8.17 (d, *J* = 8.1 Hz, 1H), 8.04 (d, *J* = 8.1 Hz, 1H), 7.99 (d, *J* = 7.4 Hz, 1H), 7.80 (dt, *J* = 9.2, 5.7 Hz, 2H), 7.72 (t, *J* = 7.4 Hz, 1H), 7.59 (d, *J* = 7.8 Hz, 6H), 7.42 (t, *J* = 7.7 Hz, 1H), 7.34 (dt, *J* = 26.5, 7.3 Hz, 9H), 7.22 (q, *J* = 5.3, 4.8 Hz, 2H), 6.49 (q, *J* = 5.1, 4.5 Hz, 1H) ppm. **^{13}C{^{1}H} NMR** (151 MHz, $CDCl_3$): δ = 146.3, 142.8, 136.2, 134.8, 132.6, 130.1, 129.4, 129.2, 129.0, 128.8, 128.5, 128.0, 127.8, 127.5, 127.3, 126.5, 124.4, 124.0, 119.7, 110.2, 75.9 ppm. **HRMS** (ESI): $[M+Na]^+$ = $[C_{35}H_{26}O_2N_4NaS]^+$, *m*/*z* calculated 589.16687, found 589.16693. **IR** (ATR): $\tilde{\nu}$ = 3426, 3059, 2925, 2322, 2185, 2157, 2108, 2063, 1963, 1815, 1726, 1594, 1491, 1445, 1349, 1279, 1189, 1078, 1031, 999, 934, 897, 830, 802, 744, 698 cm^{-1}. **TLC**: R_f (SiO_2, pentane/DCM/MTBE 12:2:1) = 0.18.

1*H*-Benzo[*d*][1,2,3]triazol-1-yl *N*-tritylbenzo[*d*][1,3]dioxole-5-sulfonimidate (3s)

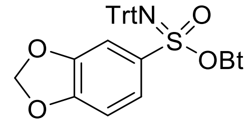

3s

The synthesis was performed according to standard procedure C starting from benzo[*d*][1,3]dioxole-5-diazonium tetrafluoroborate (118 mg, 0.50 mmol, 1.0 equiv), *N*-tritylsulfinylamine (168 mg, 0.55 mmol, 1.1 equiv), HOBt hydrate (100 mg, 0.65 mmol, 1.3 equiv) and *N*-methylpiperidine (67 µL, 0.55 mmol, 1.1 equiv). The title compound (175 mg, 0.31 mmol, 62%) was obtained as white foam.

^{1}H NMR (600 MHz, $CDCl_3$): δ = 7.85 (d, *J* = 7.3 Hz, 1H), 7.66 -7.46 (m, 7H), 7.46 – 7.27 (m, 10 H), 7.24 – 7.17 (m, 2H), 6.83 (d, *J* = 8.3 Hz, 1H), 6.57 (d, *J* = 8.1 Hz, 1H), 6.14 (s, 2H) ppm. **^{13}C{^{1}H} NMR** (151 MHz, $CDCl_3$): δ = 153.2, 148.6, 146.2, 142.8, 129.1, 127.9, 127.8, 127.7, 127.2, 125.2, 124.4, 119.6, 110.2 108.9, 108.2, 102.8, 74.8 ppm. **HRMS** (ESI): $[M+Na]^+$ = $[C_{32}H_{24}O_4N_4NaS]^+$, *m/z* calculated 583.1411, found 583.1412. **IR** (ATR): $\tilde{\nu}$ = 3061, 2912, 2646, 2322, 2223, 2165, 2082, 2021, 1962, 1760, 1599, 1479, 1360, 1245, 1199, 1114, 1081, 1034, 930, 896, 813, 746, 700 cm^{-1}. **TLC** (SiO_2, pentane/DCM/MTBE 12:2:1) = 0.17.

1*H*-Benzo[*d*][1,2,3]triazol-1-yl 5-bromo-*N*-tritylpyridine-3-sulfonimidate (3t)

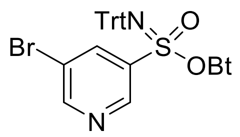

3t

The synthesis was performed according to standard procedure C starting from 5-bromopyridine-3-diazonium tetrafluoroborate (136 mg, 0.50 mmol, 1.0 equiv), *N*-tritylsulfinylamine (168 mg, 0.55 mmol, 1.1 equiv), HOBt hydrate (100 mg, 0.65 mmol, 1.3 equiv) and *N*-methylpiperidine (67 µL, 0.55 mmol, 1.1 equiv). The crude product was purified by flash column chromatography (SiO_2, 3 x 30 cm, pentane/DCM/Et_2O 7:1:1). The title compound (206 mg, 0.35 mmol, 69%) was obtained as yellowish foam.

^{1}H NMR (600 MHz, $CDCl_3$): δ = 9.12 (d, *J* = 2.0 Hz, 1H), 8.98 (d, *J* = 2.1 Hz, 1H), 8.32 (t, *J* = 2.1 Hz, 1H), 7.92 – 7.86 (m, 1H), 7.50 – 7.47 (m, 6 H), 7.41 – 7.28 (m, 11H), 6.58 – 6.50 (m, 1H) ppm. **^{13}C{^{1}H} NMR** (151 MHz, $CDCl_3$): δ = 156.0, 147.5, 145.7, 143.0, 138.6, 134.7, 129.1, 128.5, 128.5, 128.4, 128.2, 127.7, 124.9, 121.0, 120.0, 109.8, 75.8 ppm. **HRMS** (ESI): $[M+Na]^+$ = $[C_{30}H_{22}O_2N_5BrNaS]^+$, *m/z* calculated 618.0570, found 618.0544. **IR** (ATR): $\tilde{\nu}$ = 3059, 2324, 2182, 2081, 1987, 1807, 1595, 1555, 1490, 1443, 1375, 1289, 1199, 1081, 1013, 936, 895, 848, 800, 744, 695, 656 cm^{-1}. **TLC** R_f (SiO_2, pentane/DCM/Et_2O 7:1:1) = 0.18.

1*H*-Benzo[*d*][1,2,3]triazol-1-yl *N*-tritylthiazole-2-sulfonimidate (3u)

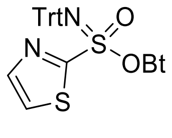

3u

The synthesis was performed according to standard procedure C starting from thiazole-2-diazonium tetrafluoroborate (used wet since it was unstable, assumed purity 85%, 118 mg, 0.50 mmol, 1.0 equiv), *N*-tritylsulfinylamine (169 mg, 0.56 mmol, 1.1 equiv), HOBt hydrate (100 mg, 0.65 mmol, 1.3 equiv) and *N*-methylpiperidine (67 µL, 0.56 mmol, 1.1 equiv). The product was purified by flash column chromatography (SiO_2, 3 x 30 cm, pentane/DCM/MTBE 3:1:1). The title compound (80 mg, 0.15 mmol, 30%) was obtained as brown foam.

^{1}H NMR (600 MHz, $CDCl_3$): δ = 8.02 (d, *J* =3.1 Hz, 1H), 7.88 – 7.86 (m, 1H), 7.82 (d, *J* = 3.1 Hz, 1H), 7.55 – 7.50 (m, 6H), 7.38 – 7.34 (m, 6H), 7.34 – 7.27 (m, 5H), 6.68 (dd, *J* = 7.5, 1.5 Hz, 1H) ppm. **^{13}C{^{1}H} NMR** (151 MHz, $CDCl_3$): δ = 160.4, 145.8, 145.2, 143.0, 129.1, 128.7, 128.3, 128.2, 127.6, 124.8, 119.9, 109.9, 75.8 ppm. **HRMS** (ESI): $[M+Na]^+$ = $[C_{28}H_{21}O_2N_5NaS_2]^+$, *m*/*z* calculated 546.1029, found 546.1019. **IR** (ATR): $\tilde{\nu}$ = 3061, 2323, 2159, 2094, 1950, 1771, 1655, 1595, 1488, 1444, 1385, 1316, 1279, 1214, 1155, 1071, 1033, 999, 937, 898, 847, 745, 699, 668 cm^{-1}. **TLC**: R_f (SiO_2, pentane/DCM/MTBE 3:1:1) = 0.20.

1*H*-Benzo[*d*][1,2,3]triazol-1-yl 1-phenyl-*N*-trityl-1*H*-pyrazole-3-sulfonimidate (3v)

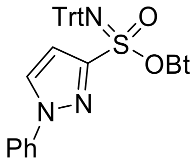

3v

The synthesis was performed according to standard procedure C starting from 1-phenyl-1H-pyrazole-5-diazonium tetrafluoroborate (129 mg, 0.50 mmol, 1.0 equiv), *N*-tritylsulfinylamine (168 mg, 0.55 mmol, 1.1 equiv), HOBt hydrate (100 mg, 0.65 mmol, 1.30 equiv) and *N*-methylpiperidine (67 µL, 0.55 mmol, 1.1 equiv). The crude product was purified by flash column chromatography (SiO_2, 3 x 30 cm, pentane/DCM/MTBE 10:4:1). The title compound (114 mg, 0.20 mmol, 39%) was obtained as yellowish foam.

^{1}H NMR (600 MHz, $CDCl_3$): δ = 8.01 (d, *J* = 2.5 Hz, 1H), 7.89 – 7.84 (m, 1H), 7.64 (t, *J* = 5.2 Hz, 2H), 7.62 – 7.56 (m, 6H), 7.46 (td, *J* = 8.0, 2.6 Hz, 3 H), 7.36 (dt, *J* = 8.2, 4.8 Hz, 8H), 7.31 (dd, *J* = 8.4, 6.1 Hz, 3H), 7.05 (d, *J* = 2.5 Hz, 1H), 6.69 – 6.66 (m, 1H) ppm. **^{13}C{^{1}H} NMR** (151 MHz, $CDCl_3$): δ = 147.6, 146.3, 143.1, 139.3, 129.7, 129.6, 129.2, 128.6, 128.5, 128.0, 127.9, 127.4, 124.5, 120.5, 119.7, 110.9, 110.3, 75.0 ppm. **HRMS** (ESI): $[M+Na]^+$ = $[C_{34}H_{26}O_2N_6NaS]^+$, *m*/*z* calculated 605.1730,

found 605.1723. **IR** (ATR): $\tilde{\nu}$ = 3059, 2926, 2323, 2193, 2085, 2000, 1888, 1758, 1596, 1496, 1444, 1375, 1269, 1216, 1185, 1077, 981, 943, 900, 847, 747, 696, 655 cm^{-1}. **TLC**: R_f (SiO_2, pentane/DCM/MTBE 10:4:1) = 0.26.

1*H*-Benzo[*d*][1,2,3]triazol-1-yl *N*-tritylquinoline-6-sulfonimidate (3w)

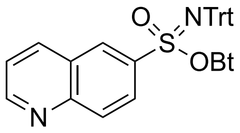

3w

The synthesis was performed according to standard procedure C starting from quinoline-6-diazonium tetrafluoroborate (121 mg, 0.50 mmol, 1.0 equiv), *N*-tritylsulfinylamine (168 mg, 0.55 mmol, 1.1 equiv), HOBt hydrate (100 mg, 0.65 mmol, 1.30 equiv) and *N*-methylpiperidine (67 µL, 0.55 mmol, 1.1 equiv). The crude product was purified by flash column chromatography (SiO_2, 3 x 30 cm, pentane/DCM/MTBE 10:4:1). The title compound (88 mg, 0.16 mmol, 31%) was obtained as off-white foam.

^{1}H NMR (600 MHz, $CDCl_3$): δ = 9.10 (dd, *J* = 4.0, 1.8 Hz, 1H), 8.48 (dd, *J* = 9.0, 2.2 Hz, 1H), 8.38 (d, *J* = 9.0 Hz, 1H), 8.25 (d, *J* = 2.2 Hz, 1H), 8.16 (d, *J* = 8.3 Hz, 1H), 7.83 (dd, *J* = 7.0, 2.7 Hz, 1H), 7.61 – 7.55 (m, 6H), 7.53 (ddd, *J* = 8.4, 4.3, 1.5 Hz, 1H), 7.38 (t, *J* = 7.8 Hz, 6H), 7.36 – 7.32 (m, 3H), 7.32 – 7.22 (m, 2H), 6.56 – 6.52 (m, 1H) ppm. **^{13}C{^{1}H} NMR** (151 MHz, $CDCl_3$): δ = 153.8, 150.0, 146.0, 142.8, 137.6, 133.9, 131.8, 130.6, 129.1, 128.3, 128.0, 127.9, 127.4, 127.3, 127.0, 124.5, 122.8, 119.6, 109.9, 75.1 ppm. **HRMS** (ESI): $[M+Na]^+$ = $[C_{34}H_{25}O_2N_5NaS]^+$, calcd. 590.1621, found 590.1610. **IR** (ATR): 3059, 2649, 2325, 2203, 2162, 2081, 2046, 1987, 1949, 1913, 1803, 1730, 1683, 1595, 1490, 1444, 1356, 1280, 1183, 1122, 1077, 1032, 998, 940, 899, 836, 798, 745, 699, 668 cm^{-1}. **TLC:** R_f (SiO_2, pentane/DCM/Et_2O 1:1:1) = 0.17.

Sulfonimidamides

4-(4-Fluoro-*N*-tritylphenylsulfonimidoyl)morpholine (8a)

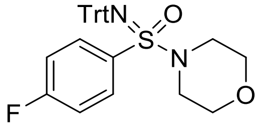

8a

The synthesis was performed according to standard procedure D starting from the reactants 1*H*-benzo[*d*][1,2,3]triazol-1-yl 4-fluoro-N-tritylbenzenesulfonimidate (164 mg, 0.30 mmol, 1.0 equiv) and morpholine (31 μL, 0.36 mmol, 1.2 equiv). The reaction mixture was stirred for 24 h at RT. The crude product was purified by flash column chromatography (SiO_2, 2.5 x 20 cm, pentane/EtOAc 8:1). (4-Fluoro-*N*-tritylphenylsulfonimidoyl)morpholine (116 mg, 0.24 mmol, 79%) was obtained as a white foam.

^{1}H NMR (600 MHz, $CDCl_3$): δ = 7.97 – 7.91 (m, 2H), 7.54 (dd, *J* = 7.7, 1.8 Hz, 5 H), 7.29 – 7.24 (m, 7H), 7.23 – 7.19 (m, 5H), 3.20 (dt, *J* = 11.3, 4.7 Hz, 2H), 3.12 (dt, *J* = 11.3, 4.7 Hz, 2H), 2.65 (t, *J* = 4.7 Hz, 4H) ppm. **^{13}C{^{1}H} NMR** (151 MHz, $CDCl_3$): δ = 164.7 (d, *J* = 253.4 Hz), 147.9, 134.2 (d, *J* = 3.1 Hz), 129.8 (d, *J* = 9.1 Hz), 129.2, 127.5, 126.5, 115.9 (d, *J* = 22.3 Hz), 72.2, 65.9, 46.4 ppm. **^{19}F NMR** (564 MHz, $CDCl_3$): δ = –107.5 (tt, *J* = 8.9, 5.2 Hz) ppm. **HRMS** (ESI): $[M+Na]^+$ = $[C_{29}H_{27}O_2N_2FNaS]^+$, calculated 509.1670, found 509.1670. **IR** (ATR): $\tilde{\nu}$ = 3059, 3024, 2966, 2853, 2322, 2070, 2013, 1960, 1904, 1776, 1589, 1488, 1447, 1397, 1301, 1259, 1177, 1150, 1110, 1068, 1031, 926, 897, 837, 751, 701, 667 cm^{-1}. **TLC**: R_f (SiO_2, pentane/EtOAc 8:1) = 0.25. **Melting point:** 166.3 °C.

4-Fluoro-*N*-methyl-*N'*-tritylbenzenesulfonimidamide (8c)

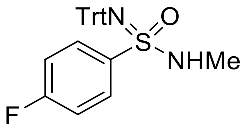

8c

The synthesis was not performed according to standard procedure D for sulfonimidamides. Instead, 1*H*-benzo[*d*][1,2,3]triazol-1-yl 4-fluoro-*N*-tritylbenzene-sulfonimidate (164 mg, 0.30 mmol, 1.0 equiv) was dissolved in a solution of methyl amine (1.0 mL, 8.0 mmol, 26.7 equiv, 8 M in EtOH). The mixture was stirred for 2 h at RT. The resulting suspension was diluted with DCM, absorbed on Celite® and the

crude product was purified by column chromatography (SiO_2, 2.5x20 cm, pentane/DCM/MTBE 20:2:1). 4-Fluoro-*N*-methyl-*N*'-tritylbenzenesulfonimidamide (78 mg, 0.18 mmol, 60%) was obtained as a white solid.

^{1}H NMR (600 MHz, $CDCl_3$): δ = 7.98 (ddd, *J* = 8.7, 5.3, 2.5 Hz, 2H), 7.57 (dd, *J* = 7.6, 1.7 Hz, 6H), 7.28 – 7.23 (m, 7H), 7.20 (td, *J* = 6.7, 3.1 Hz, 3H), 7.12 (t, *J* = 8.6 Hz, 2H), 3.42 (s, 1H), 2.19 (s, 3H) ppm. **^{13}C{^{1}H} NMR** (151 MHz, $CDCl_3$): δ = 164.3 (d, *J* = 252.6 Hz), 147.7, 138.6 (d, *J* = 3.2 Hz), 129.6 (d, *J* = 9.1 Hz), 129.0, 127.5, 126.4, 115.7 (d, *J* = 22.3 Hz), 71.9, 29.5 ppm. **^{19}F NMR** (564 MHz, $CDCl_3$): δ = –108.2 (td, *J* = 8.3, 4.2 Hz) ppm. **HRMS** (ESI): $[M+Na]^+$ = $[C_{26}H_{23}ON_2FNaS]^+$, *m*/*z* calculated 453.1407, found 453.1404. **IR** (ATR): $\tilde{\nu}$ = 3281, 3055, 2925, 2854, 2656, 2319, 2163, 2062, 2017, 1957, 1896, 1738, 1589, 1488, 1443, 1399, 1291, 1224, 1143, 1076, 1008, 939, 901, 833, 750, 696 cm^{-1}. **TLC**: R_f (SiO_2, 2.5x20 cm, pentane/DCM/MTBE 20:2:1) = 0.11. **Melting point**: 174.9 °C (degradation).

N-Ethyl-4-fluoro-*N*'-tritylbenzenesulfonimidamide (8d)

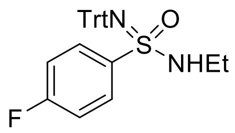

8d

The synthesis was performed according to standard procedure D starting from 1*H*-benzo[*d*][1,2,3]triazol-1-yl 4-fluoro-*N*-tritylbenzenesulfonimidate (164 mg, 0.30 mmol, 1.0 equiv) and ethyl amine solution (180 μL, 0.36 mmol, 1.2 equiv, 2 M in MeOH). 4-Fluoro-*N*-ethyl-*N*'-tritylbenzenesulfonimidamide (105 mg, 0.236 mmol, 79%) was obtained as a white foam.

^{1}H NMR (600 MHz, $CDCl_3$): δ = 8.05 – 7.95 (m, 2H), 7.61 – 7.54 (m, 6H), 7.32 – 7.22 (m, 7H), 7.23 – 7.17 (m, 2H), 7.12 (t, *J* = 8.6 Hz, 2H), 3.31 (s, 1H), 2.65 – 2.46 (m, 2H), 0.67 (t, *J* = 7.2 Hz, 3H) ppm. **^{13}C{^{1}H} NMR** (151 MHz, $CDCl_3$): δ = 164.4 (d, *J* = 252.4 Hz), 147.9, 139.8 (d, *J* = 3.1 Hz), 129.6 (d, *J* = 8.9 Hz), 129.1, 127.6, 126.5, 115.8 (d, *J* = 22.6 Hz), 72.1, 38.3, 14.6 ppm. **^{19}F NMR** (564 MHz, $CDCl_3$): δ = –108.3 (tt, *J* = 8.8, 5.0 Hz) ppm. **HRMS** (ESI): $[M+Na]^+$ = $[C_{27}H_{25}ON_2FNaS]^+$, *m*/*z* calculated 467.1564, found 467.1568. **IR** (ATR): $\tilde{\nu}$ = 3242, 3027, 2929, 2869, 2313, 2163, 2077, 1960, 1877, 1738, 1590, 1490, 1443, 1404, 1271, 1143, 1069, 1019, 964, 895, 835, 753, 691 cm^{-1}. **TLC:** R_f (SiO_2, pentane/EtOAc 12:1) = 0.20. **Melting point:** 141.0 °C (discoloration).

4-Fluoro-*N*-isopropyl-*N*'-tritylbenzenesulfonimidamide (8e)

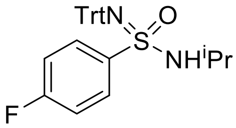

8e

The synthesis was performed according to standard procedure D starting from 1*H*-benzo[*d*][1,2,3]triazol-1-yl 4-fluoro-*N*-tritylbenzenesulfonimidate (164 mg, 0.30 mmol, 1 equiv) and isopropyl amine (52 μL, 0.60 mmol, 2.0 equiv). The crude product was purified by flash column chromatography (SiO_2, 2.5 x 20 cm, pentane/EtOAc 15:1). 4-Fluoro-*N*-isopropyl-*N*'-tritylbenzenesulfonimidamide (114 mg, 0.23 mmol, 75%) was obtained as a white foam.

^{1}H NMR (600 MHz, $CDCl_3$): δ = 7.98 (dt, *J* = 8.3, 4.4 Hz, 2H), 7.64 – 7.50 (m, 6H), 7.31 – 7.23 (m, 6H), 7.21 (q, *J* = 7.2, 5.6 Hz, 3H), 7.15 – 7.07 (m, 2H), 3.44 (s, 1H), 3.13 (dq, *J* = 15.0, 8.6, 7.4 Hz, 1H), 0.76 (t, *J* = 5.2 Hz, 3H), 0.62 (t, *J* = 5.1 Hz, 3H) ppm. **^{13}C{^{1}H} NMR** (151 MHz, $CDCl_3$): δ = 164.3 (d, *J* = 252.2 Hz), 148.1, 141.5 (d, *J* = 2.9 Hz), 129.4, 129.4 (d, *J* = 8.9 Hz), 129.1, 127.6, 126.5, 115.6 (d, *J* = 22.3 Hz), 72.3, 45.9, 23.7, 23.1 ppm. **^{19}F NMR** (564 MHz, $CDCl_3$): δ = –108.6 (dq, *J* = 13.1, 7.6, 6.2 Hz) ppm. **HRMS** (ESI): $[M+Na]^+$ = $[C_{28}H_{27}ON_2FNaS]^+$, *m/z* calculated 481.1720, found 481.1718. **IR** (ATR): $\tilde{\nu}$ = 3346, 3062, 2971, 2930, 2873, 2315, 2162, 2045, 1971, 1899, 1811, 1591, 1488, 1446, 1402, 1272, 1232, 1175, 1131, 1091, 1030, 991, 939, 871, 834, 751, 699 cm^{-1}. **TLC**: R_f (SiO_2, pentane/EtOAc 15:1) = 0.20. **Melting point:** 136.2 °C.

N-Benzyl-4-fluoro-*N*'-tritylbenzenesulfonimidamide (8f)

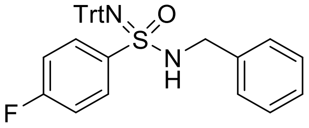

8f

The synthesis was performed according to standard procedure D starting from the reactants 1*H*-benzo[*d*][1,2,3]triazol-1-yl 4-fluoro-*N*-tritylbenzenesulfonimidate (164 mg, 0.30 mmol, 1.00 equiv) and benzyl amine (64 mg, 0.60 mmol, 2.0 equiv). The crude product was purified by flash column chromatography (SiO_2, 2.5 x 20 cm, pentane/DCM/EtOAc 32:4:1). *N*-Benzyl-4-fluoro-*N*'-tritylbenzenesulfonimidamide (108 mg, 0.21 mmol, 71%) was obtained as a white solid.

^{1}H NMR (400 MHz, $CDCl_3$): δ = 7.99 (dd, *J* = 8.6, 5.1 Hz, 2H), 7.57 (d, *J* = 7.6 Hz, 6H), 7.33 – 7.04 (m, 14H), 6.86 – 6.75 (m, 2H), 3.77 (s, 1H), 3.73 (d, *J* = 13.9 Hz, 1H), 3.58 (d, *J* = 13.5, 1H) ppm. **^{13}C{^{1}H} NMR** (101 MHz, $CDCl_3$): δ = 164.5 (d, *J* = 252.6 Hz), 147.9, 139.7 (d, *J* = 3.1 Hz), 136.8, 129.7 (d, *J* = 9.1 Hz), 129.1, 128.5, 127.9, 127.7, 127.6, 126.6, 115.8 (d, *J* = 22.4 Hz), 72.3, 47.7 ppm. **^{19}F NMR** (376 MHz, $CDCl_3$): δ = –108.0 (p, *J* = 6.7 Hz) ppm. **HRMS** (ESI): $[M+Na]^+$ = $[C_{32}H_{27}ON_2FNaS]^+$, *m/z* calculated 529.1720, found 529.1720. **IR** (ATR): $\tilde{\nu}$ = 3268, 3056, 3028, 2916, 2664, 2320, 2165, 2054, 1996, 1952, 1896, 1817, 1745, 1591, 1488, 1443, 1413, 1317, 1231, 1151, 1084, 1031, 948, 898, 829, 743, 699 cm^{-1}. **TLC**: R_f (SiO_2, pentane/DCM/EtOAc 32:4:1) = 0.13. **Melting point:** 155.8 °C.

***N*-(2-Aminobenzyl)-4-fluoro-*N'*-tritylbenzenesulfonimidamide (8i)**

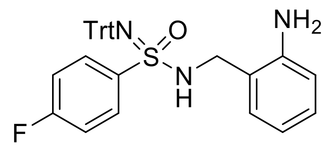

8i

The synthesis was performed according to standard procedure D starting from 1*H*-benzo[*d*][1,2,3]triazol-1-yl 4-fluoro-N-tritylbenzenesulfonimidate (164 mg, 0.30 mmol, 1 equiv) and 2-aminobenzylamine (66 μL, 0.5 mmol, 2.0 equiv). The crude product was purified by flash column chromatography (SiO_2, 2.5 x 20 cm, pentane/DCM/EtOAc 16:4:1). The title compound (58 mg, 0.11 mmol, 37%) was obtained as white foam.

^{1}H NMR: (600 MHz, $CDCl_3$): δ = 7.98 (ddt, *J* = 6.9, 4.9, 2.7 Hz, 2H), 7.86 (dt, *J* = 8.0, 2.6 Hz, 1H), 7.58 – 7.50 (m, 7H), 7.40 – 7.31 (m, 10H), 7.31 – 7.21 (m, 4H), 6.55 (dt, *J* = 6.9, 2.5 Hz, 1H), 2.05 (s, 1H) ppm. **^{13}C{^{1}H} NMR** (151 MHz, $CDCl_3$): δ = 166.5 (d, *J* = 257.9 Hz), 146.1, 143.0, 132.2 (d, *J* = 3.1 Hz), 131.8 (d, *J* = 9.7 Hz), 129.2, 128.4, 128.1, 127.5, 124.7, 119.8, 117.0 (d, *J* = 23.0 Hz), 110.1, 82.1 (benzylic CH_2, only visible in the ATP), 75.1 ppm. **^{19}F NMR** (564 MHz, $CDCl_3$): δ = –101.8 (dq, *J* = 12.8, 5.5 Hz) ppm. **MS** (ESI): *m/z* (%): 557 (12), 416 (21), 257 (34), 243 $[Trt]^+$ (100), 180 (44), 165 (40). **HRMS** (ESI): $[M+Na]^+$ = $[C_{32}H_{28}ON_3FNaS]^+$, *m/z* calculated 544.1829, found 544.1828. **IR** (ATR): $\tilde{\nu}$ = 3866, 3281, 3062, 2924, 2856, 2729, 2479, 2293, 2245, 2184, 2153, 2084, 2049, 2006, 1985, 1901, 1735, 1666, 1590, 1489, 1446, 1367, 1323, 1288, 1205, 1152, 1086, 1017, 940, 902, 864, 836, 744, 700, 673 cm^{-1}. **TLC**: R_f (SiO_2, pentane/DCM/EtOAc 16:4:1) = 0.11. **Melting point:** 145.6 °C (degradation, gas evolution; discoloration above 80 °C).

1-(4-Fluoro-*N*-tritylphenylsulfonimidoyl)pyrrolidine (8j)

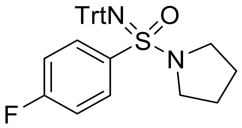

8j

The synthesis was performed according to standard procedure D starting from 1*H*-benzo[*d*][1,2,3]triazol-1-yl 4-fluoro-N-tritylbenzenesulfonimidate (160 mg, 0.29 mmol, 1.0 equiv) and pyrrolidine (25 mg, 0.35 mmol, 1.2 equiv). The reaction mixture was stirred for 24 h at RT. The crude product was purified by flash column chromatography (SiO_2, 2.5 x 20 cm, pentane/EtOAc 15:1). (4-Fluoro-*N*-tritylphenylsulfonimidoyl)pyrrolidine (116 mg, 0.25 mmol, 84%) was obtained as a white solid.

^{1}H NMR (600 MHz, $CDCl_3$): δ = 8.01 (ddd, *J* = 8.8, 5.6, 2.8 Hz, 2H), 7.60 (dd, *J* = 7.7, 1.7 Hz, 6H), 7.36 – 7.24 (m, 7H), 7.24 – 7.14 (m, 4H), 2.81 (td, *J* = 7.0, 3.6 Hz, 4H), 1.40 (td, *J* = 8.0, 6.5, 4.6 Hz, 4H) ppm. **^{13}C{^{1}H} NMR** (151 MHz, $CDCl_3$): δ = 164.3 (d, *J* = 251.8 Hz), 147.9, 137.3 (d, *J* = 3.2 Hz), 129.4 (d, *J* = 8.9 Hz), 129.3, 127.3, 126.3, 115.6 (d, *J* = 22.2 Hz), 71.9, 47.7, 25.1 ppm. **^{19}F NMR** (564 MHz, $CDCl_3$): δ = –108.58 – –108.71 (m) ppm. **HRMS** (ESI): $[M+Na]^+$ = $[C_{28}H_{27}ON_2FNaS]^+$, *m*/*z* calculated 493.1720, found 493.1720. **IR** (ATR): $\tilde{\nu}$ = 3061, 3025, 2970, 2931, 2849, 2325, 2163, 2031, 1966, 1903, 1777, 1587, 1486, 1444, 1398, 1299, 1276, 1222, 1169, 1143, 1087, 1011, 938, 895, 835, 750, 699, 673 cm^{-1}. **TLC:** R_f (SiO_2, pentane/EtOAc 15:1) = 0.21. **Melting point:** 143.2 °C.

1-(4-Fluoro-*N*-tritylphenylsulfonimidoyl)piperidine (8k)

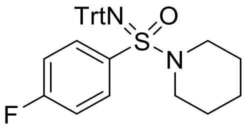

8k

The synthesis was performed according to standard procedure D starting from the reactants 1*H*-benzo[*d*][1,2,3]triazol-1-yl 4-fluoro-*N*-tritylbenzenesulfonimidate (164 mg, 0.30 mmol, 1.0 equiv) and piperidine (31 mg, 0.36 mmol, 1.2 equiv).the reaction mixture was stirred for 24 h at rt. The crude product was purified by flash column chromatography (SiO_2, 2.5 x 20 cm, pentane/EtOAc 20:1). (4-Fluoro-*N*-tritylphenylsulfonimidoyl)piperidine (118 mg, 0.24 mmol, 81%) was obtained as a white solid.

^{1}H NMR (600 MHz, $CDCl_3$): δ = 7.93 (ddd, *J* = 8.7, 5.2, 3.0 Hz, 2H), 7.59 – 7.52 (m, 6H), 7.26 (td, *J* = 7.5, 2.7 Hz, 6H), 7.19 (dtd, *J* = 11.1, 8.6, 8.0, 2.4 Hz, 5H), 2.73 – 2.61 (m, 4H), 1.19 – 1.11 (m, 4H), 1.08 (d, *J* =9.0 Hz, 2H) ppm. **^{13}C{^{1}H} NMR** (151 MHz, $CDCl_3$): δ = 164.3 (d, *J* = 252.1 Hz), 148.1, 135.5 (d, *J* = 2.9 Hz), 129.7 (d, *J* = 8.8 Hz), 129.3, 127.4, 126.3, 115.6 (d, *J* = 22.2 Hz), 72.1, 47.2, 24.9, 23.6 ppm. **^{19}F NMR** (564 MHz, $CDCl_3$): δ = –108.6 (h, *J* = 7.3 Hz) ppm. **HRMS** (ESI): $[M+Na]^+$ = $[C_{30}H_{29}ON_2FNaS]^+$, *m*/*z* calculated 507.1877, found 507.1877. **IR** (ATR): $\tilde{\nu}$ = 3060, 2936, 2830, 2321, 2186, 2106, 1904, 1737, 1589, 1488, 1445, 1399, 1302, 1273, 1223, 1173, 1151, 1091, 1033, 916, 836, 752, 699, 66 cm^{-1}. **TLC**: R_f (SiO_2, pentane/EtOAc 20:1) = 0.22. **Melting point:** 156.5 – 157.6 °C.

1-(4-Fluoro-*N*-tritylphenylsulfonimidoyl)azepane (8l)

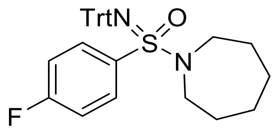

8l

The synthesis was performed according to standard procedure D starting from the reactants 1*H*-benzo[*d*][1,2,3]triazol-1-yl 4-fluoro-*N*-tritylbenzenesulfonimidate (164 mg, 0.30 mmol, 1.0 equiv) and azepane (36 mg, 0.36 mmol, 1.2 equiv).the reaction mixture was stirred for 24 h at rt. The crude product was purified by flash column chromatography (SiO_2, 2.5 x 20 cm, pentane/EtOAc 20:1). (4-Fluoro-*N*-tritylphenylsulfonimidoyl)azepane (121 mg, 0.24 mmol, 81%) was obtained as a white solid.

^{1}H NMR (600 MHz, $CDCl_3$): δ = 7.97 (ddd, *J* = 9.1, 5.1 2.2 Hz, 2H), 7.58 – 7.51 (m, 5H), 7.28 – 7.24 (m, 7H), 7.19 (td, *J* = 7.2, 1.6 Hz, 3H), 7.13 (td, *J* = 8.6, 1.7 Hz, 2H), 3.01 – 2.87 (m, 4H), 1.36 – 1.20 (m, 8H) ppm. **^{13}C{^{1}H} NMR** (151 MHz, $CDCl_3$): δ = 164.1 (d, *J* = 251.7 Hz), 148.1, 139.5 (d, *J* = 3.1 Hz), 129.3, 129.2, 127.4, 126.3, 115.6 (d, *J* = 22.2 Hz), 72.0, 48.3, 28.2, 27.1 ppm. **^{19}F NMR** (564 MHz, $CDCl_3$): δ = –109.1 (h, *J* = 6.2 Hz) ppm. **HRMS** (ESI): $[M+Na]^+$ = $[C_{31}H_{31}ON_2FNaS]^+$, *m*/*z* calculated 521.2033, found 521.2033. **IR** (ATR): $\tilde{\nu}$ = 3058, 2929, 2857, 2685, 2327, 2184, 2107, 1898, 1589, 1486, 1445, 1398, 1271, 1226, 1173, 1145, 1090, 1032, 1007, 940, 887, 835, 751, 698 cm^{-1}. **TLC:** R_f (pentane/EtOAc 20:1) = 0.26. **Melting point:** 146.7 °C.

4-(4-Fluoro-*N*-tritylphenylsulfonimidoyl)thiomorpholine (8m)

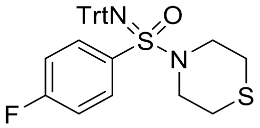

8m

The synthesis was performed according to standard procedure D starting from the reactants 1*H*-benzo[*d*][1,2,3]triazol-1-yl 4-fluoro-*N*-tritylbenzenesulfonimidate (164 mg, 0.30 mmol, 1 equiv) and thiomorpholine (36 μL, 0.36 mmol, 1.2 equiv). The reaction mixture was stirred for 24 h at rt. The crude product was purified by flash column chromatography (SiO_2, 2.5 x 20 cm, pentane/DCM/MTBE 16:2:1). (4-Fluoro-*N*-tritylphenylsulfonimidoyl)thiomorpholine (117 mg, 0.23 mmol, 78%) was obtained as a white foam.

^{1}H NMR (600 MHz, $CDCl_3$): δ = 7.97 – 7.91 (m, 2H), 7.58 – 7.50 (m, 6H), 7.33 – 7.24 (m, 7H), 7.21 (tt, *J* = 8.6, 3.0 Hz, 4H), 2.98 (q, *J* = 10.2, 9.7 Hz, 4H), 2.16 (dt, *J* = 13.4, 5.0 Hz, 2H), 2.05 (dt, *J* = 12.8, 5.1 Hz, 2H) ppm. **^{13}C{^{1}H} NMR** (151 MHz, $CDCl_3$): δ = 164.6 (d, *J* = 253.2 Hz), 147.8, 135.8 (d, *J* = 3.2 Hz), 129.4 (d, *J* = 8.9 Hz), 129.3, 127.5, 126.6, 116.0 (d, *J* = 22.6 Hz), 72.2, 48.1, 26.9 ppm. **^{19}F NMR** (564 MHz, $CDCl_3$): δ = –107.7 (tt, *J* = 8.9, 5.2 Hz) ppm. **HRMS** (ESI): $[M+Na]^+$ = $[C_{29}H_{27}ON_2FNaS_2]^+$, *m*/*z* calculated 525.1441, found 525.1441. **IR** (ATR): $\tilde{\nu}$ = 3059, 2957, 2917, 2846, 2325, 2186, 2102, 2047, 2010, 1984, 1898, 1772, 1588, 1487, 1446, 1409, 1369, 1298, 1273, 1231, 1174, 1148, 1089, 1051, 1024, 965, 928, 878, 835, 764, 744, 697, 660 cm^{-1}. **TLC**: R_f (SiO_2, pentane/DCM/MTBE 16:2:1) = 0.28. **Melting point:** 171.7 °C (partial degradation).

1-(4-Fluoro-*N*-tritylphenylsulfonimidoyl)-4-methylpiperazine (8n)

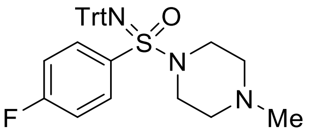

8n

The synthesis was performed according to standard procedure D starting from the reactants 1*H*-benzo[*d*][1,2,3]triazol-1-yl 4-fluoro-*N*-tritylbenzenesulfonimidate (164 mg, 0.30 mmol, 1.0 equiv) and *N*-methylpiperazine (36 mg, 0.36 mmol, 1.2 equiv). The reaction mixture was stirred for 24 h at RT. The crude product was purified by flash column chromatography (SiO_2, 2.5 x 20 cm, DCM w/ 1% EtOH + 0.5% Et_3N). 1-

(4-Fluoro-*N*-tritylphenylsulfonimidoyl)-4-methylpiperazine (126 mg, 0.25 mmol, 84%) was obtained as a white foam.

^{1}H NMR (600 MHz, $CDCl_3$): δ = 7.93 (ddd, *J* = 8.7, 5.3, 2.5 Hz, 2H), 7.62 – 7.51 (m, 6H), 7.33 – 7.24 (m, 7H), 7.24 – 7.15 (m, 4H), 2.73 (s, 4H), 2.09 (s, 3H), 2.04 – 1.86 (m, 4H) ppm. **^{13}C{^{1}H} NMR** (151 MHz, $CDCl_3$): δ = 164.5 (d, *J* = 252.8 Hz), 147.9, 134.4 (d, *J* = 3.5 Hz), 129.8 (d, *J* = 8.9 Hz), 129.3, 127.5, 126.1, 115.6 (d, *J* = 22.7 Hz), 72.2, 53.8, 46.2, 45.7 ppm. **^{19}F NMR** (564 MHz, $CDCl_3$): δ = –108.0 (p, *J* = 6.8 Hz). **HRMS** (ESI): $[M+Na]^+$ = $[C_{30}H_{30}ON_3FNaS]^+$, *m*/*z* calculated 522.1986, found 522.1987. **IR** (ATR): $\tilde{\nu}$ = 3059, 2936, 2844, 2799, 2322, 2184, 2107, 2003, 1903, 1589, 1488, 1278, 1227, 1175, 1145, 1090, 1033, 1005, 925, 836, 750, 700, 668 cm^{-1}. **TLC**: R_f (SiO_2, DCM w/ 1% EtOH + 0.5% Et_3N) = 0.18. **Melting point:** 166.2 °C (partial degradation).

1-(4-Fluoro-*N*-tritylphenylsulfonimidoyl)piperidin-4-ol (8o)

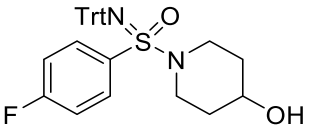

8o

The synthesis was performed according to standard procedure D starting from the reactants 1*H*-benzo[*d*][1,2,3]triazol-1-yl 4-fluoro-*N*-tritylbenzenesulfonimidate (164 mg, 0.30 mmol, 1.0 equiv) and 4-hydroxypiperidine (36 mg, 0.36 mmol, 1.2 equiv). The reaction mixture was stirred for 24 h at RT. The crude product was purified by flash column chromatography (SiO_2, 2.5 x 20 cm, DCM w/ 1% EtOH). (4-Fluoro-*N*-tritylphenylsulfonimidoyl)piperidine-4-ol (120 mg, 0.24 mmol, 80%) was obtained as a white foam.

^{1}H NMR (600 MHz, $CDCl_3$): δ = 7.93 (dd, *J* = 8.6, 5.0 Hz, 2H), 7.53 (d, *J* = 7.8 Hz, 6H), 7.30 – 7.22 (m, 7H), 7.19 (dd, *J* = 8.1, 5.3 Hz, 4H), 3.41 (tt, *J* = 8.0, 3.8 Hz, 1H), 3.07 (tt, *J* = 11.9, 4.6 Hz, 2H), 2.39 (dt, *J* = 20.9, 12.0 Hz, 2H), 1.47 – 1.33 (m, 2H), 1.15 (s, 1H), 1.09 (dtd, *J* = 12.3, 8.4, 3.6 Hz, 1H), 0.97 (dtd, *J* = 12.6, 8.6, 3.7 Hz, 1H) ppm. **^{13}C{^{1}H} NMR** (151 MHz, $CDCl_3$): δ = 164.5 (d, *J* = 252.6 Hz), 148.0, 135.4 (d, *J* = 2.5 Hz), 129.6 (d, *J* = 8.8 Hz), 129.3, 127.5, 126.4, 115.8 (d, *J* = 22.2 Hz), 72.2, 66.6, 43.6 (d, *J* = 28.9 Hz), 33.1 (d, *J* = 22.4 Hz) ppm. **^{19}F NMR** (564 MHz, $CDCl_3$): δ = –108.1 (td, *J* = 8.8, 4.5 Hz) ppm. **HRMS** (ESI): $[M+Na]^+$ = $[C_{30}H_{29}O_2N_2FNaS]^+$, *m*/*z* calculated 523.1826, found 523.1826. **IR** (ATR): $\tilde{\nu}$ = 3748, 3474, 3063, 2998, 2927, 2849, 2324, 2208, 2165, 2073, 2048, 1990, 1894, 1588, 1486, 1447, 1396, 1298,

1275, 1229, 1167, 1146, 1085, 1034, 994, 895, 833, 751, 700 cm^{-1}. **TLC**: R_f (SiO_2, DCM/EtOH 99:1) = 0.23. **Melting point:** >109.6 °C (degradation, gas evolution).

Other synthesized compounds

N-Tritylsulfinylamine (1)

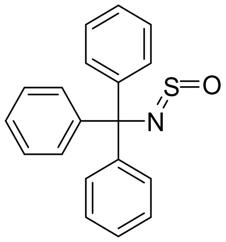

1

The synthesis followed a procedure published by *Willis* and co-workers.[1]

In an oven-dried Schlenk-flask, tritylamine (9010 mg, 30.5 mmol, 1.0 equiv) was added to dry diethyl ether (150 mL) under argon. Then, triethylamine (dried over KOH, 8.5 mL, 61 mmol, 2.0 equiv) was added and the mixture was cooled to 0 °C. Subsequently, thionyl chloride (distilled, 2.22 mL, 30.5 mmol, 1.0 equiv) was added dropwise. The mixture was stirred for additional 2 h at 0 °C. The precipitated solid was filtered off through Celite® and washed with diethyl ether. The crude product was dissolved in pentane/EtOAc 95:5 and filtered through silica. The solvent was evaporated without heating under reduced pressure. TrtNSO (4446 mg, 14.6 mmol, 48%) was obtained as white crystals.

The NMR spectra corresponded to the spectra published by *Willis* and coworkers.[1]

1-Butyl-1-imino-1,2-dihydro-3*H*-1λ^4-benzo[*d*]isothiazol-3-one 1-oxide (10)

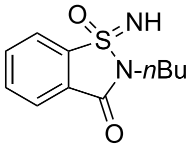

10

The intermediate sulfonimidate was prepared according to standard procedure C starting from 2-(methoxycarbonyl)benzenediazonium tetrafluoroborate (125 mg, 0.50 mmol, 1.0 equiv), *N*-tritylsulfinylamine (169 mg, 0.56 mmol, 1.1 equiv), HOBt hydrate (100 mg, 0.65 mmol, 1.3 equiv) and *N*-methylpiperidine (67 µL, 0.56 mmol, 1.1

equiv). The resulting crude product (brown oil) was taken up in non-dry MeCN (1.7 mL), and *n*BuNH$_2$ (99 μL, 1.00 mmol, 2.0 equiv) and Et$_3$N (70 μL, 0.50 mmol, 1.0 equiv) were added. The brown-red solution was stirred under argon for 24 h, then the solvent was removed under reduced pressure. The residue was taken up in DCM (1 mL), and 1 drop of water and 10 drops of TFA were added. The yellow solution was stirred at room temperature for 20 min, then the solvent was again removed under reduced pressure, and the product was purified by FCC (silica, pentane/EtOAc 1:1). Brown oil (57 mg, 0.24 mmol, 48%).

^{1}H NMR (600 MHz, CDCl$_3$): δ = 8.00 (dt, *J* = 7.4, 0.9 Hz, 1H), 7.90 (dt, *J* = 7.6, 0.9 Hz, 1H), 7.83 – 7.79 (m, 1H), 7.76 (td, *J* = 7.5, 1.1 Hz, 1H), 3.78 – 3.63 (m, 2H), 3.11 (s, 1H), 1.87 – 1.74 (m, 2H), 1.43 (h, *J* = 7.4 Hz, 2H), 0.96 (t, *J* = 7.4 Hz, 3H) ppm. **^{13}C{^{1}H} NMR** (151 MHz, CDCl$_3$): δ = 159.7, 140.9, 134.6, 134.0, 127.6, 125.0, 121.1, 38.5, 30.9, 20.3, 13.7 ppm. **MS** (ESI): m/z (%): 499 (15) [2M+Na]$^+$, 339 (58), 261 (100) [M+Na]$^+$, 239 (51) [M+H]$^+$, 183 (41) [M-C$_4$H$_8$]$^+$. **HRMS** (ESI): [M+Na]$^+$ = [C$_{11}$H$_{14}$O$_2$N$_2$NaS]$^+$, calcd. 261.0668, found 261.0665. **IR** (ATR): $\tilde{\nu}$ = 3264, 2959, 2935, 2871, 2326, 2116, 1709, 1594, 1460, 1334, 1254, 1339, 1109, 986, 935, 789, 749, 677 cm^{-1}. **DC**: R$_f$ (SiO$_2$, Pentan/EtOAc 1:1) = 0.24.

5-(2-Ethoxy-5-nitrophenyl)-1-methyl-3-propyl-1,6-dihydro-7*H*-pyrazolo[4,3-*d*]pyrimidin-7-one (14)

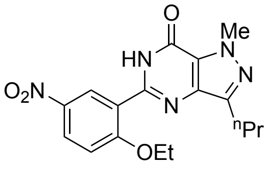

14

5-(2-Ethoxyphenyl)-1-methyl-3-propyl-1,6-dihydro-7H-pyrazolo[4,3-d]pyrimidin-7-one (1017 mg, 3.26 mmol, 1.0 equiv) was dissolved in conc. H$_2$SO$_4$ (5 mL, 0.65 M) at RT. The solution was cooled to 0 °C (ice bath), and potassium nitrate (362 mg, 3.58 mmol, 1.1 equiv) was added portion-wise. The solution was stirred at 0 °C for 1 h, then poured onto ice (100 g), and extracted with DCM (3 x 30 mL). The combined organic fractions were washed with sat. aq. sodium carbonate solution (30 mL), dried over MgSO$_4$, and the solvent was evaporated under reduced pressure. The obtained product (faint yellowish powder, 1111 mg, 3.11 mmol, 95%) was of sufficient purity and directly used in the next step.

^{1}H NMR (600 MHz, CDCl$_3$): δ = 10.80 (s, 1H), 9.29 (d, *J* = 2.9 Hz, 1H), 8.32 (dd, *J* = 9.2, 3.0 Hz, 1H), 7.13 (d, *J* = 9.2 Hz, 1H), 4.41 (q, *J* = 7.0 Hz, 2H), 4.26 (s, 3H), 2.98 – 2.90 (m, 2H), 1.86 (h, *J* = 7.4 Hz, 2H), 1.65 (t, *J* = 7.0 Hz, 3H), 1.04 (t, *J* = 7.4 Hz, 3H) ppm. **^{13}C{^{1}H} NMR** (151 MHz, CDCl$_3$): δ = 160.7, 153.7, 147.3, 146.1, 142.3, 138.4, 127.4, 124.6, 121.4, 113.1, 66.7, 38.4, 27.8, 22.5, 14.6, 14.2 ppm. **MS** (ESI): m/z (%): 380 (100) [M+Na]$^+$. **HRMS** (ESI): [M+Na]$^+$ = [$C_{17}H_{19}O_4N_5Na$]$^+$, calcd. 380.1329, found 380.1322. **IR** (ATR): $\tilde{\nu}$ = 3324, 3120, 2954, 2653, 2473, 2324, 2158, 2042, 1943, 1687, 1600, 1560, 1520, 1478, 1408, 1344, 1265, 1235, 1151, 1072, 1026, 928, 839, 786, 743, 677 cm^{-1}. **Melting point:** 208.5 – 210.4 °C (degrad.).

5-(5-Amino-2-ethoxyphenyl)-1-methyl-3-propyl-1,6-dihydro-7*H*-pyrazolo[4,3 *d*]pyrimidin-7-one (15)

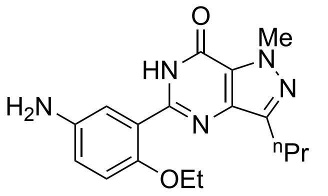

15

A 50 mL-Schlenk flask was charged with 5-(2-ethoxy-5-nitrophenyl)-1-methyl-3-propyl-1,6-dihydro-7H-pyrazolo[4,3-d]pyrimidin-7-one (500 mg, 1.40 mmol, 1.0 equiv) and Pd/C 5 w% (25 mg, 5 w%). The flask was closed with a rubber septum and purged with argon (3 x). Ethanol (10 mL) and THF (10 mL) were added with a syringe, and the suspension was stirred for 10 min at RT. A balloon with hydrogen was attached with a syringe, and the argon was removed by a short application of vacuum. The suspension was stirred under hydrogen for 24 h at rt, then filtered over celite® (solid residues were washed with EtOAc), and the yellow solution was concentrated under reduced pressure. The crude product was used without further purification. Yellowish solid, 447 mg (1.37 mmol, 98%).

^{1}H NMR (600 MHz, CDCl$_3$): δ = 11.32 (s, 1H), 7.81 (d, *J* = 3.0 Hz, 1H), 6.87 (d, *J* = 8.7 Hz, 1H), 6.79 (dd, *J* = 8.7, 3.0 Hz, 1H), 4.26 (s, 3H), 4.18 (q, *J* = 7.0 Hz, 2H), 3.64 (s_{br}, 2H), 2.94 – 2.89 (m, 2H), 1.86 (h, *J* = 7.4 Hz, 2H), 1.54 (t, *J* = 7.0 Hz, 3H), 1.03 (t, *J* = 7.4 Hz, 3H) ppm. **^{13}C{^{1}H} NMR** (151 MHz, CDCl$_3$): δ = 154.1, 149.9, 148.6, 146.7, 141.1, 138.8, 124.6, 120.9, 119.5, 116.7, 115.3, 66.2, 38.3, 27.9, 22.6, 15.0, 14.2 ppm. **MS** (ESI): m/z (%): 694 (12), 432 (12), 350 (41), 328 (100) [M+H]$^+$, 299 (41).

5-[2-Ethoxy-5-(4-methyl-*N*-tritylpiperazine-1-sulfonimidoyl)phenyl]-1-methyl-3-propyl-1,6-dihydro-7*H*-pyrazolo[4,3-*d*]pyrimidin-7-one (16)

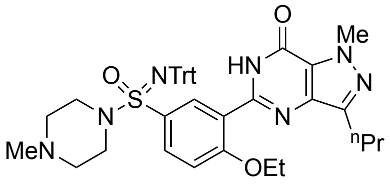

16

4-Ethoxy-3-(1-methyl-7-oxo-3-propyl-6,7-dihydro-1H-pyrazolo[4,3-d]pyrimidin-5-yl)-benzenediazonium tetrafluoroborate (213 mg, 0.50 mmol, 1.0 equiv) was subjected to the standard procedure C to yield crude 1*H*-benzo[*d*][1,2,3]triazol-1-yl 4-ethoxy-3-(1-methyl-7-oxo-3-propyl-6,7-dihydro-1*H*-pyrazolo[4,3-*d*]pyrimidin-5-yl)-*N*-trityl-benzenesulfonimidate (red gel). That compound was dissolved in neat *N*-methylpiperazine (1 mL) and stirred for 18 h at RT. Evaporation of the volatiles under reduced pressure and purification by FCC (SiO_2, DCM/EtOH 95:5) furnished 5-(2-ethoxy-5-(4-methyl-*N*-tritylpiperazine-1-sulfonimidoyl)phenyl)-1-methyl-3-propyl-1,6-dihydro-7*H*-pyrazolo[4,3-*d*]pyrimidin-7-one (205 mg, 0.29 mmol, 57%) as yellowish foam.

^{1}H NMR (600 MHz, $CDCl_3$): δ = 10.92 (s, 1H), 8.97 (d, *J* = 2.5 Hz, 1H), 8.00 (dd, *J* = 8.7, 2.5 Hz, 1H), 7.59 – 7.54 (m, 6H), 7.26 – 7.23 (m, 6H), 7.20 – 7.16 (m, 3H), 7.11 (d, *J* = 8.8 Hz, 1H), 4.35 (q, *J* = 7.0 Hz, 2H), 4.28 (s, 3H), 2.91 (t, *J* = 7.6 Hz, 2H), 2.79 (s_{br}, 4H), 2.09 (s, 3H), 2.00 (s_{br}, 2H), 1.95 (s_{br}, 2H), 1.82 (h, *J* = 7.4 Hz, 2H), 1.64 (t, *J* = 7.0 Hz, 3H), 0.92 (t, *J* = 7.4 Hz, 3H) ppm. **^{13}C{^{1}H} NMR** (151 MHz, $CDCl_3$): δ = 158.5, 153.9, 148.0, 147.1, 147.0, 138.7, 132.2, 131.3, 130.6, 129.3, 127.5, 126.4, 124.6, 120.5, 112.8, 72.2, 66.1, 53.9, 46.1, 45.7, 38.4, 27.8, 22.3, 14.8, 14.1 ppm. **MS** (ESI): m/z (%) = 1431 (60) [2M+H], 764 (16), 434 (24), 243 (100) $[Trt]^+$. **HRMS** (ESI): $[M+H]^+$ = $[C_{46}H_{40}O_3N_7]^+$, calcd. 738.3187, found. 738.3192. **IR** (ATR): $\tilde{\nu}$ = 3315, 3058, 2935, 2846, 2799, 2322, 2163, 2049, 1989, 1956, 1916, 1694, 1587, 1487, 1449, 1391, 1276, 1241, 1170, 1143, 1081, 1029, 927, 814, 750, 700, 658 cm^{-1}. **TLC:** R_f (SiO_2, DCM/EtOH 95:5) = 0.20.

5-[2-Ethoxy-5-(4-methylpiperazine-1-sulfonimidoyl)phenyl]-1-methyl-3-propyl-1,6-dihydro-7*H*-pyrazolo[4,3-*d*]pyrimidin-7-one (13)

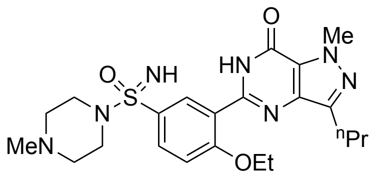

13

Acidolysis of **16** in wet DCM (1 mL DCM + 3 drops of water) with TFA (40 drops) at room temperature deprotected the trityl group within 20 minutes. The solution was diluted by DCM (20 mL) and washed with sat. aq. Na_2CO_3 solution (3 x 5 mL) and dried over Na_2SO_4. Removal of the solvent under reduced pressure afforded the crude product, which was purified by FCC (silica, DCM/EtOH/Et_3N 94:5:1). Off-white solid [126 mg, 0.27 mmol, 93% (53% over two steps)].

Notes:

- The reaction does not proceed under the standard conditions for the conversion of *N*-Trt-*O*-Bt-sulfonimidates to *N*-Trt-sulfonimidamides (1.2 equiv secondary amine).
- The basic washing in the last step is crucial to remove trifluoroacetic acid, which would otherwise be dragged through the column by the basic eluent, resulting in the contamination of the final product with triethylammonium trifluoroacetate.

^{1}H NMR (600 MHz, $CDCl_3$): δ = 10.82 (s, 1H), 8.88 (d, *J* = 2.5 Hz, 1H), 7.95 (dd, *J* = 8.7, 2.4 Hz, 1H), 7.11 (d, *J* = 8.8 Hz, 1H), 4.35 (q, *J* = 7.0 Hz, 2H), 4.26 (s, 3H), 3.17 – 3.03 (m, 4H), 2.91 (t, *J* = 7.6 Hz, 2H), 2.60 (s, 1H), 2.52 – 2.43 (m, 4H), 2.25 (s, 3H), 1.85 (h, *J* = 7.4 Hz, 2H), 1.62 (t, *J* = 7.0 Hz, 3H), 1.01 (t, *J* = 7.4 Hz, 3H) ppm. **^{13}C{^{1}H} NMR** (151 MHz, $CDCl_3$): δ = 159.2, 153.8, 147.1, 146.7, 138.6, 132.3, 131.5, 129.0, 124.6, 121.0, 112.9, 66.1, 54.6, 47.1, 45.8, 38.4, 27.9, 22.4, 14.7, 14.2 ppm. **MS** (ESI): m/z (%) = 474 $[M+H]^+$ (100). **HRMS** (ESI): $[M+H]^+$ = $[C_{22}H_{32}O_3N_7S]^+$, calcd. 474.2282, found 474.2271. **IR** (ATR): $\tilde{\nu}$ = 3377, 3213, 2927, 2848, 2799, 2495, 2292, 2169, 2022, 1917, 1683, 1584, 1457, 1390, 1285, 1136, 1033, 992, 941, 812, 705 cm^{-1}. **Melting point:** 172.3 - 176.7 (degrad.). **TLC:** R_f (SiO_2, DCM/EtOH/Et_3N 94:5:1) = 0.23.

5. References

[1] T. Q. Davies, A. Hall, M. C. Willis, *Angew. Chem. Int. Ed.* **2017**, *56*, 14937-14941; *Angew. Chem.* **2017**, *129*, 15133-15137.

6. NMR spectra

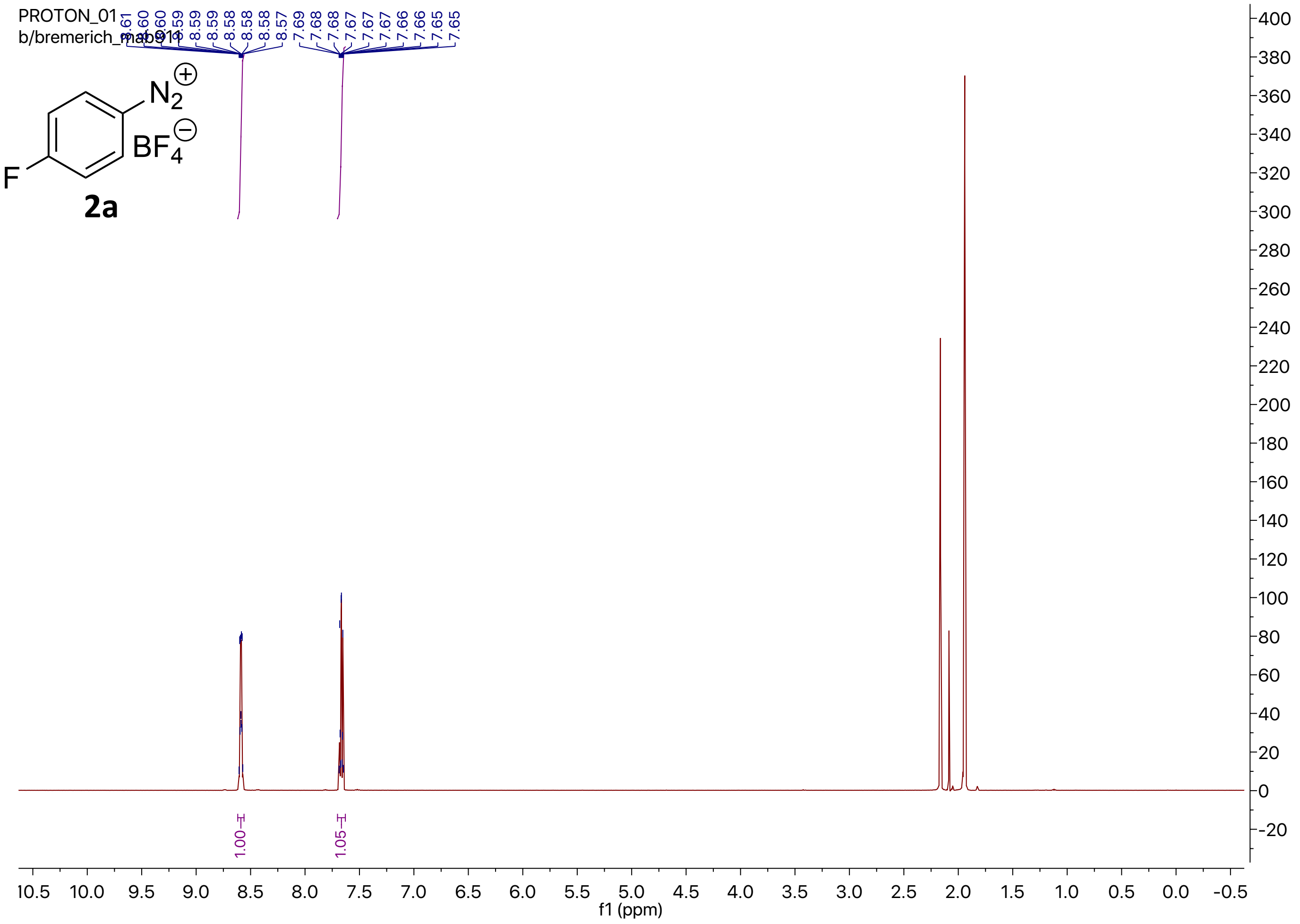

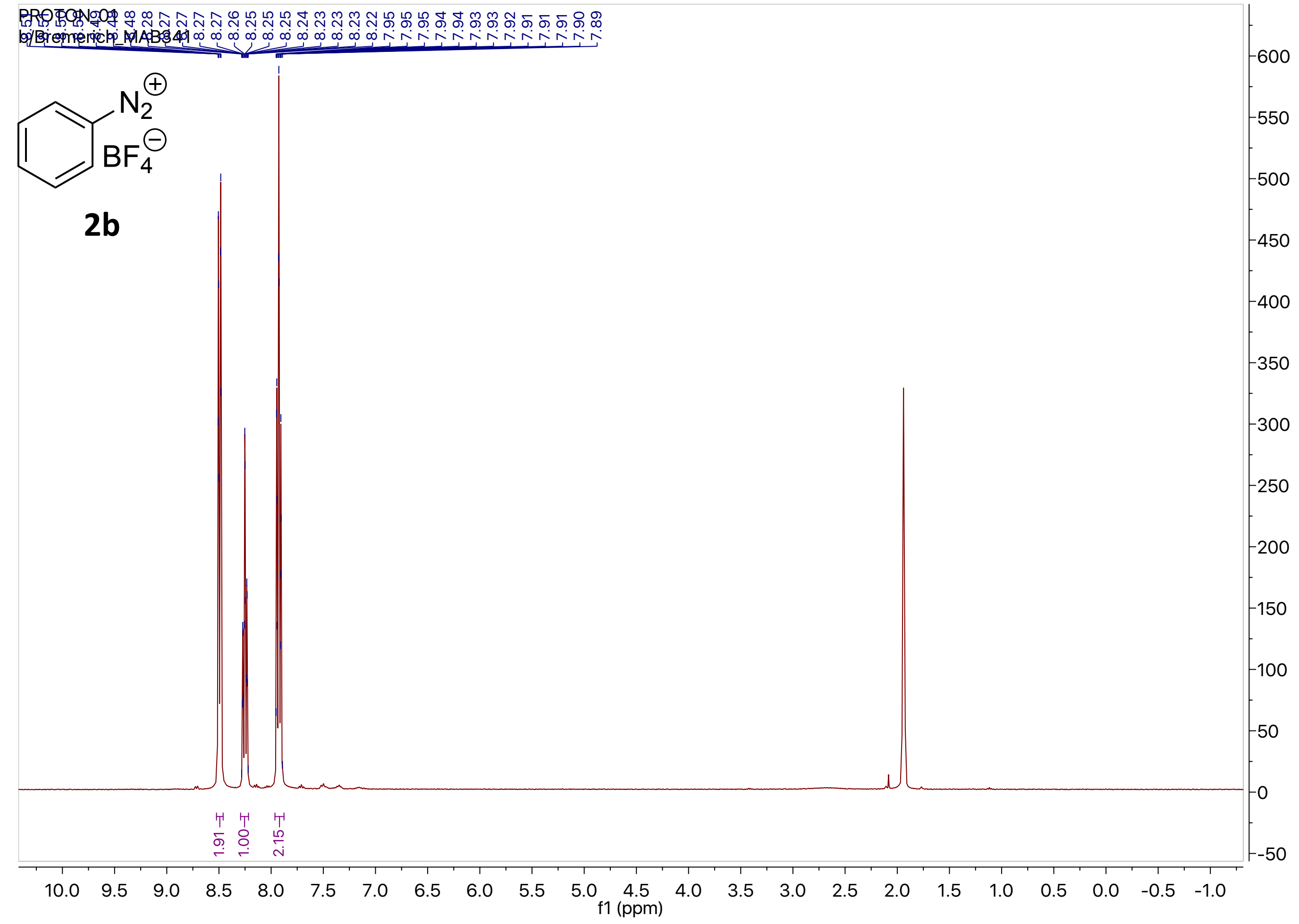

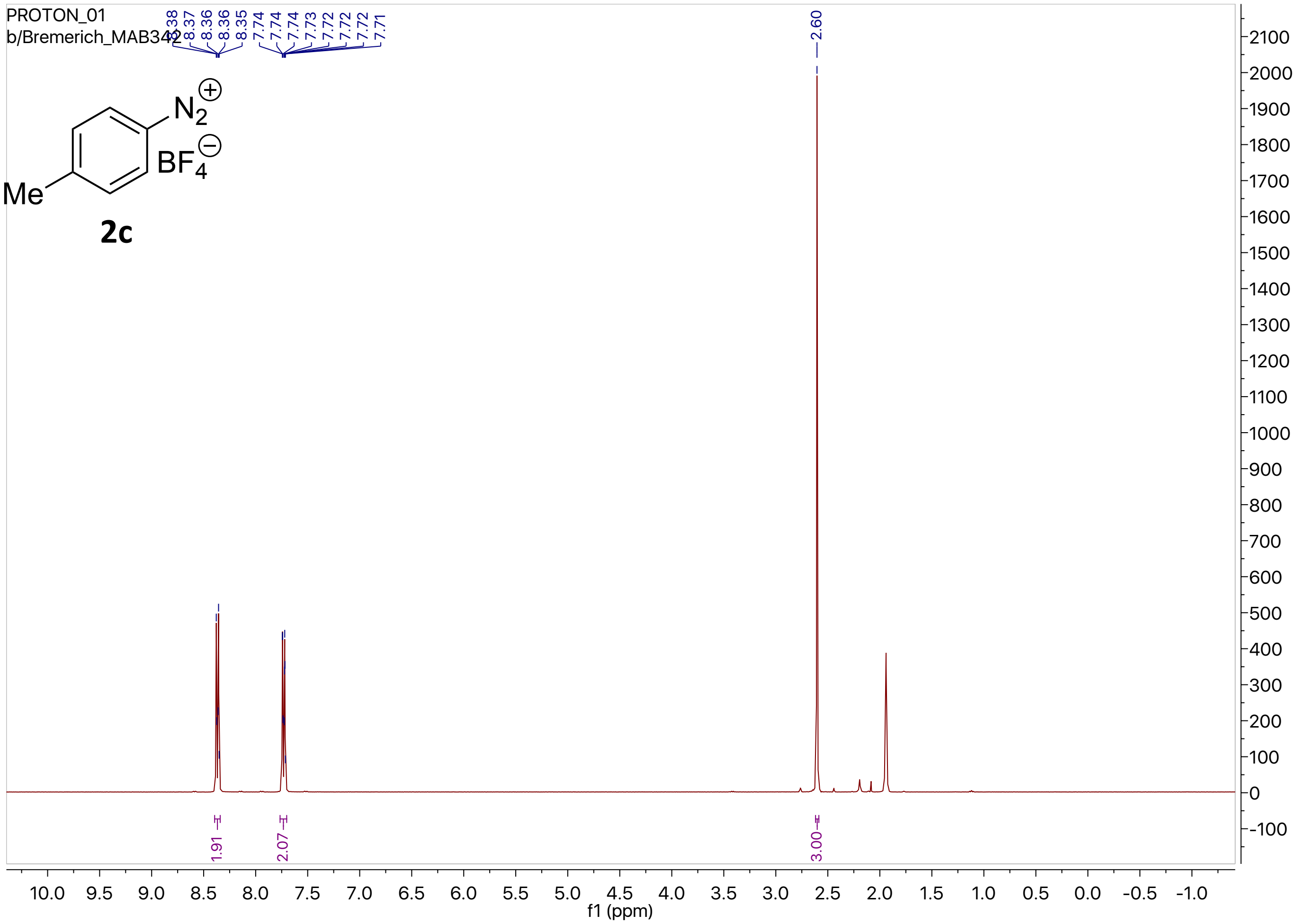
PROTON_01
b/Bremerich_MAB342
2c
f1 (ppm)

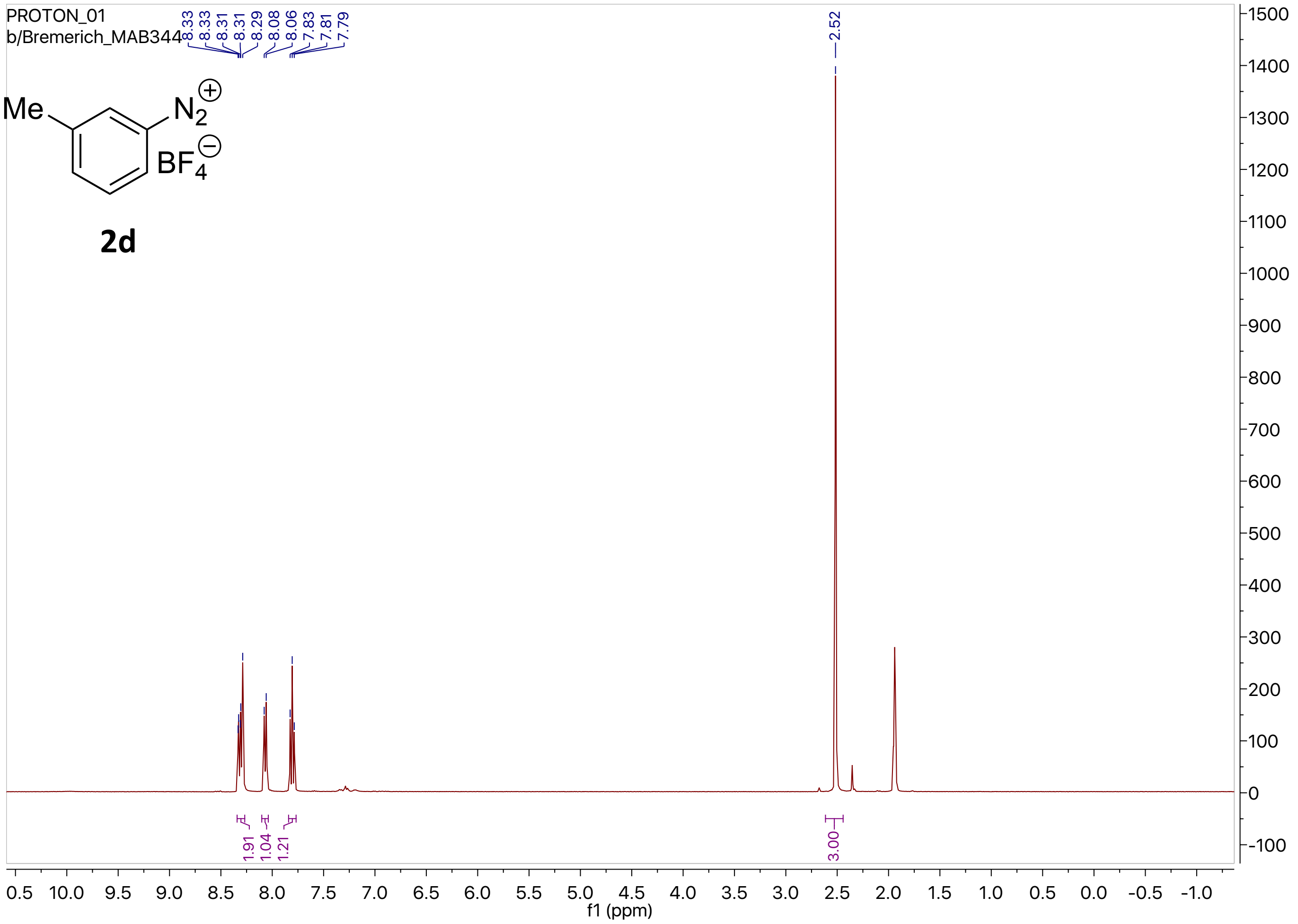
PROTON_01
b/Bremerich_MAB344
2d
f1 (ppm)

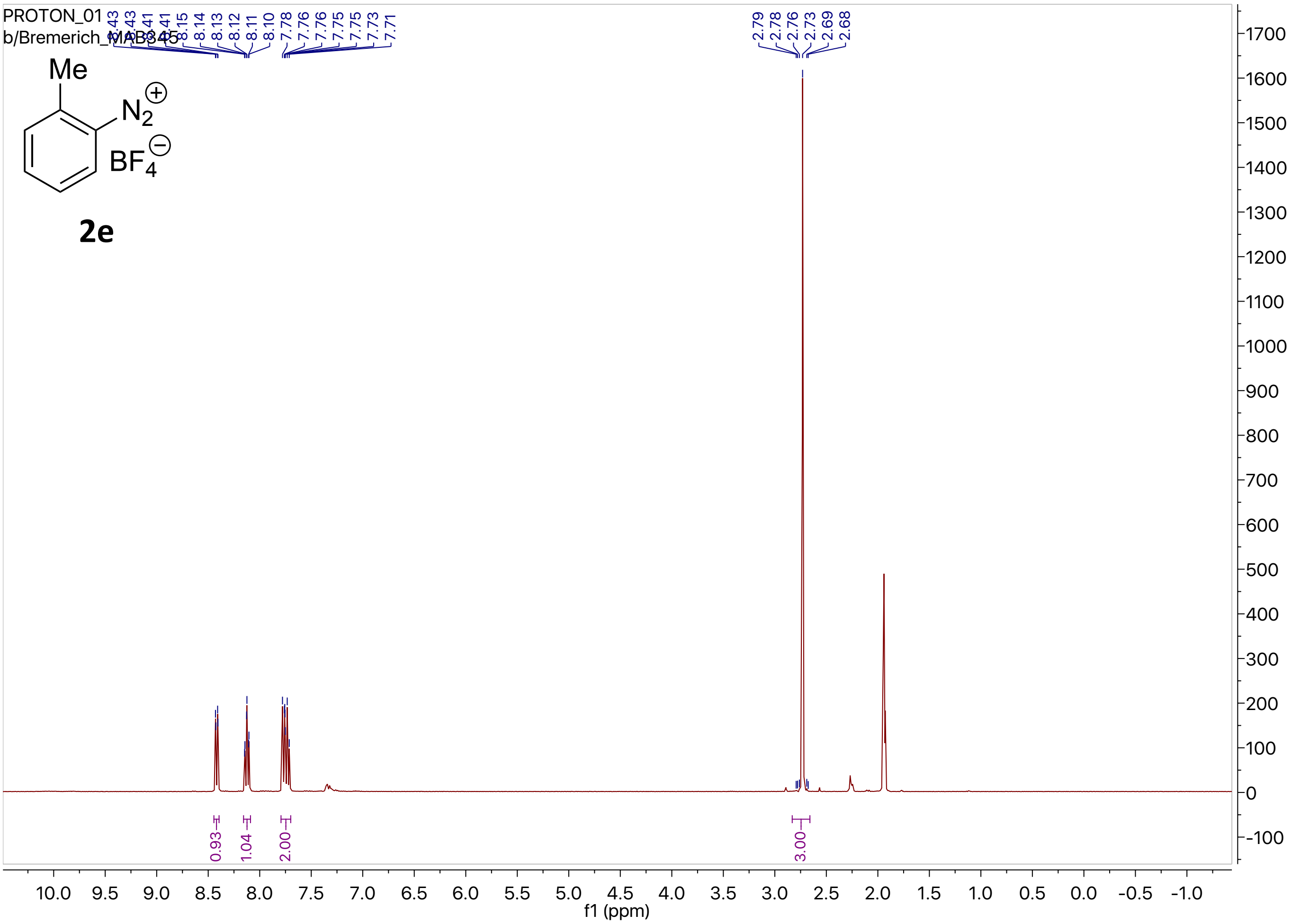
PROTON_01
b/Bremerich_MAB345
8.43 8.43 8.41 8.41 8.15 8.14 8.13 8.12 8.11 8.10 7.78 7.76 7.76 7.75 7.75 7.73 7.71
2.79 2.78 2.76 2.73 2.69 2.68
Me
N2
BF4
2e
0.93 1.04 2.00 3.00
f1 (ppm)

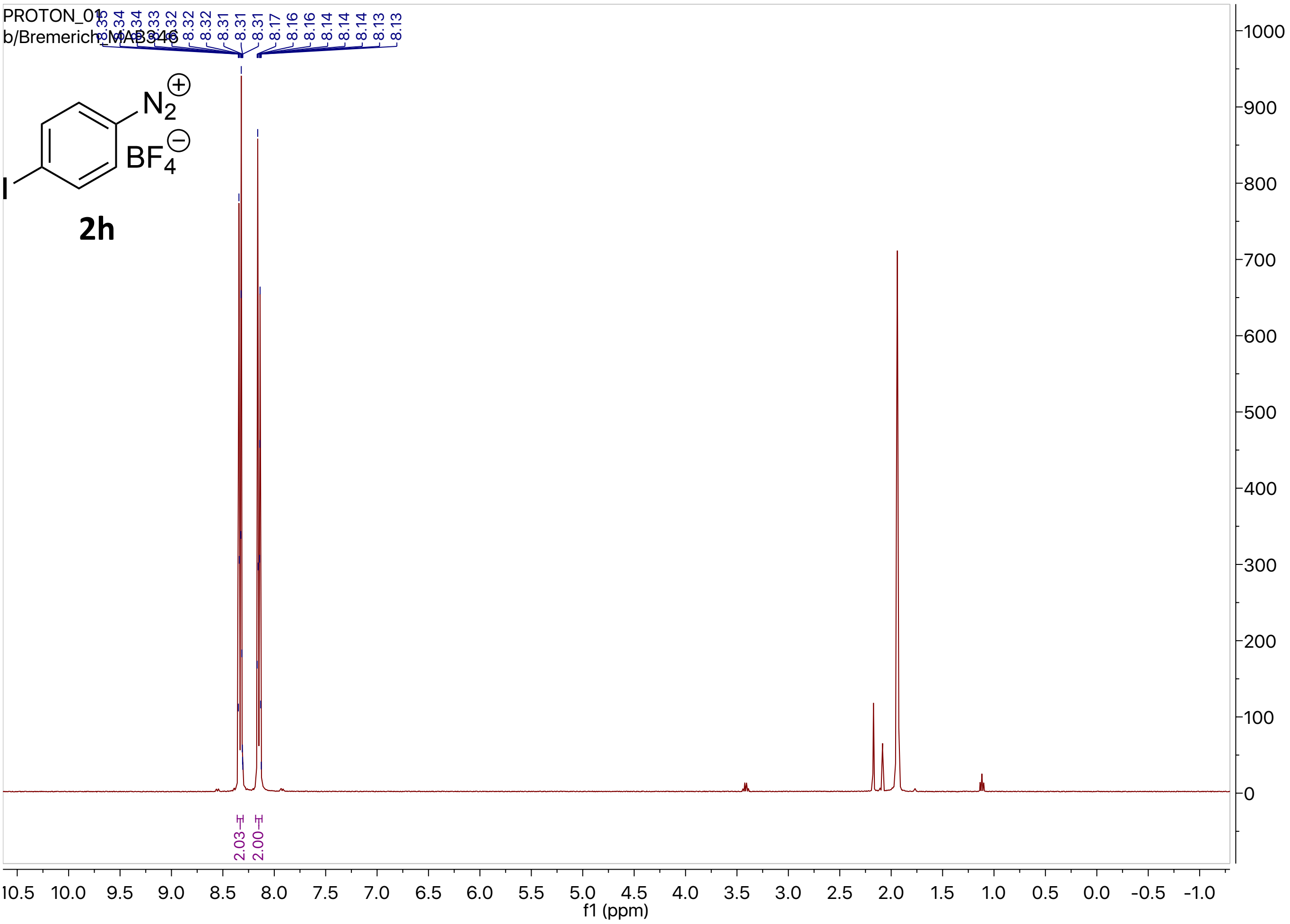
PROTON_01
b/Bremerich_MAB346
8.34 8.34 8.33 8.32 8.32 8.31 8.31 8.31 8.17 8.16 8.16 8.14 8.14 8.13 8.13
N2
BF4
I
2h
2.03 2.00
f1 (ppm)

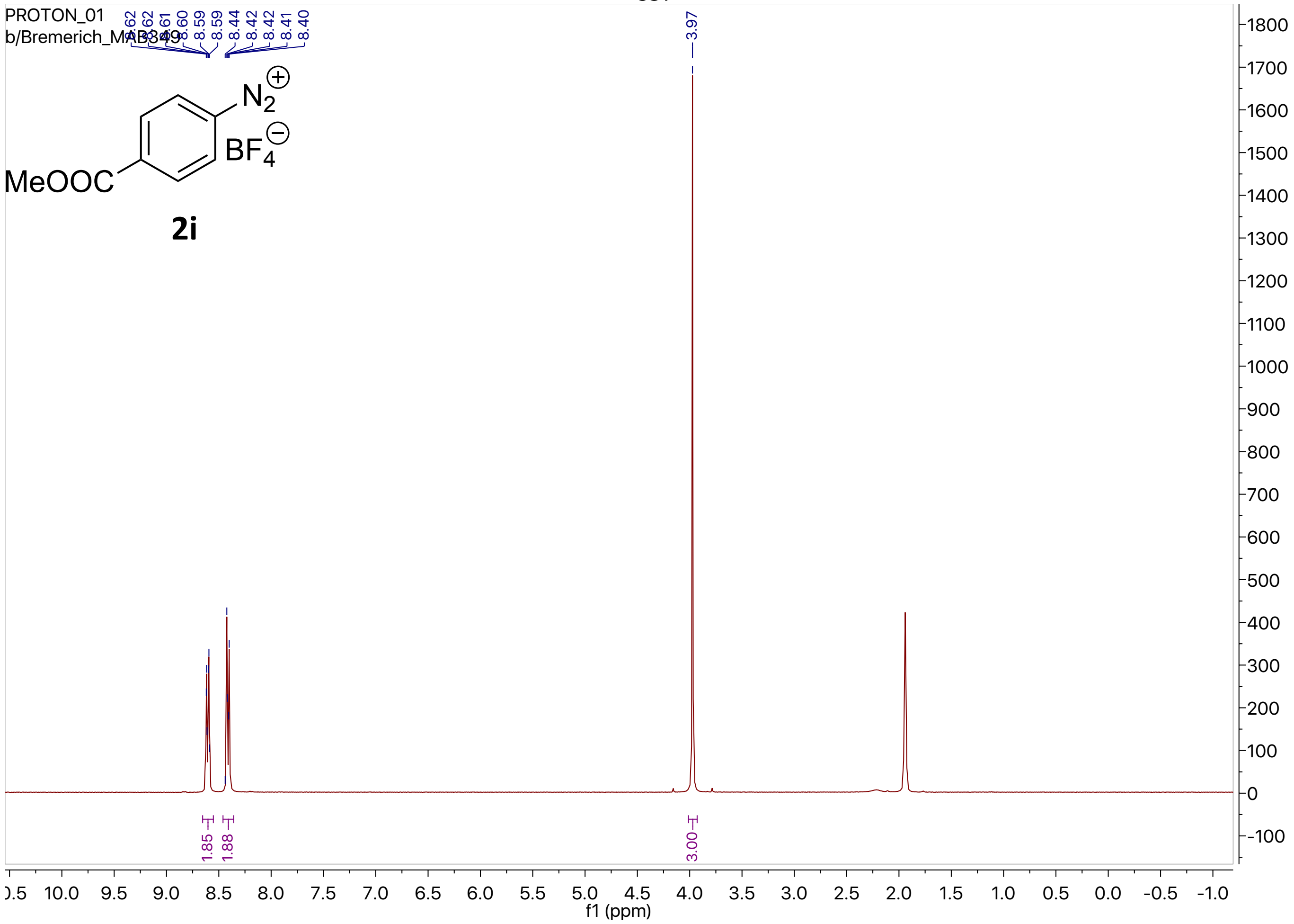
PROTON_01
8.62 8.62 8.61 8.60 8.59 8.59 8.44 8.42 8.42 8.41 8.40
3.97
MeOOC
$N_2^{\oplus}$
$BF_4^{\ominus}$
2i
1.85
1.88
3.00
f1 (ppm)

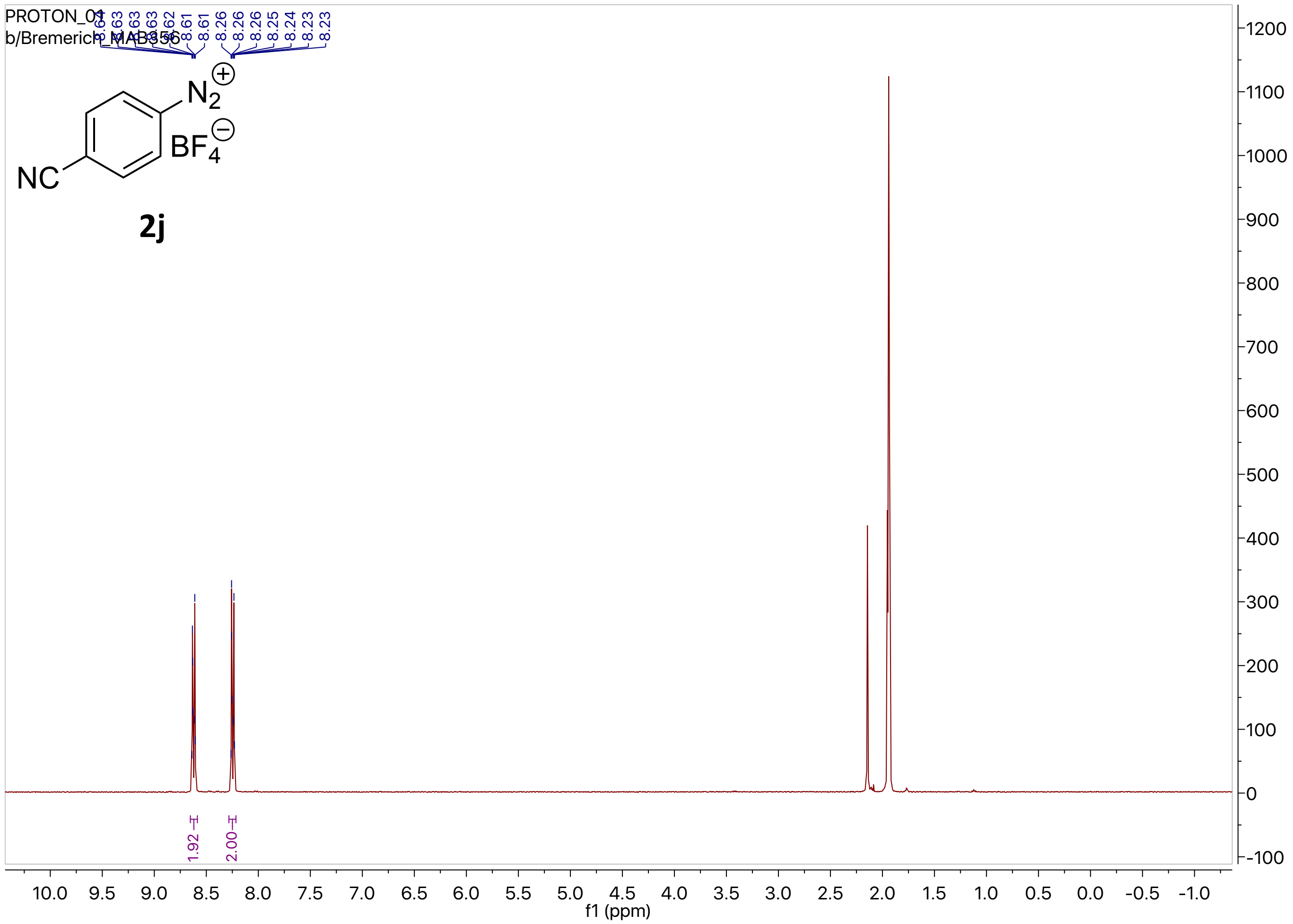
PROTON_01
8.63 8.63 8.62 8.61 8.26 8.26 8.25 8.24 8.23 8.23
NC
$N_2^{\oplus}$
$BF_4^{\ominus}$
2j
1.92
2.00
f1 (ppm)

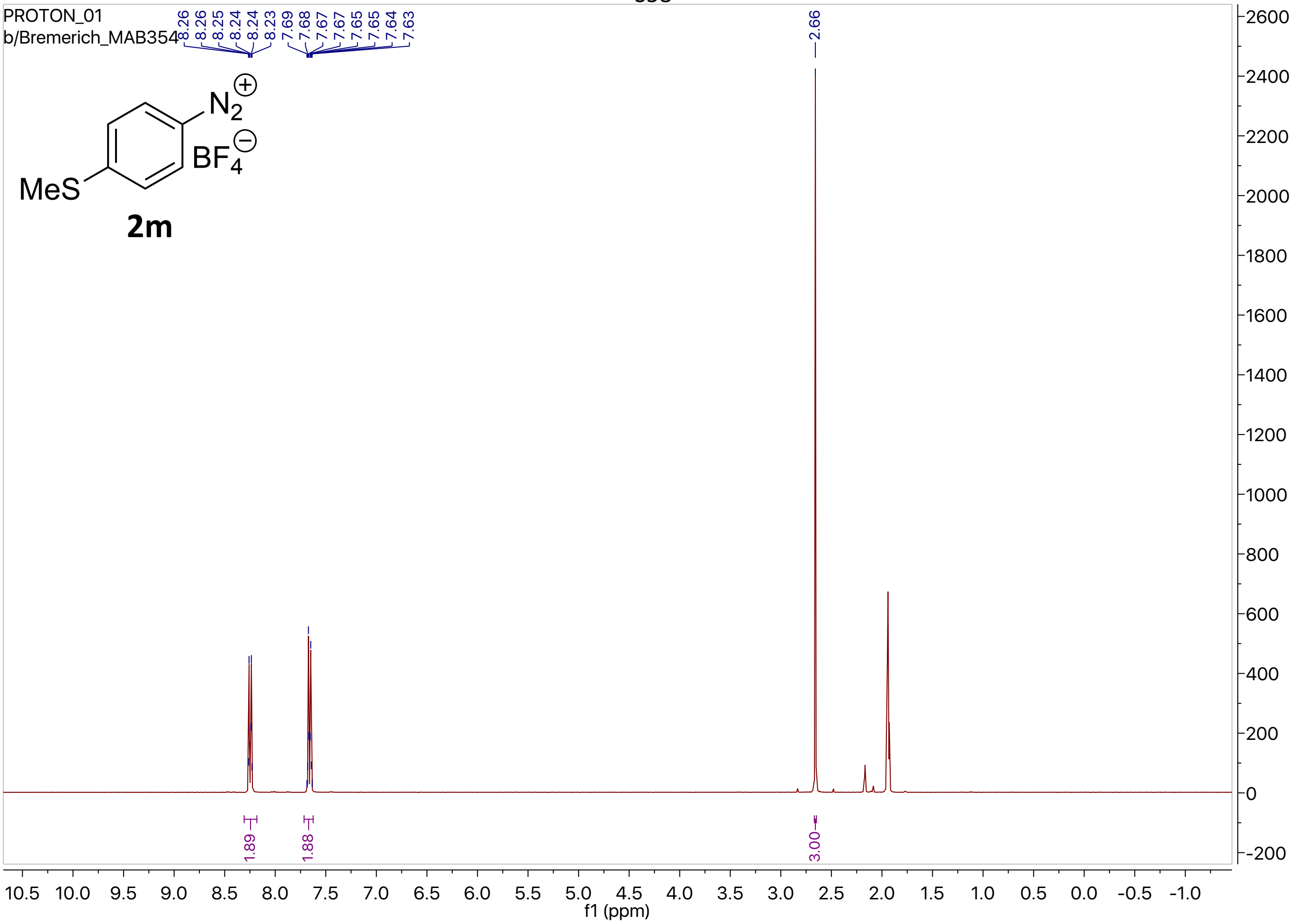
PROTON_01
b/Bremerich_MAB354
8.26 8.26 8.25 8.24 8.24 8.23 7.69 7.68 7.67 7.67 7.65 7.65 7.64 7.63
2.66
MeS
$N_2^{\oplus}$
$BF_4^{\ominus}$
2m
1.89
1.88
3.00
f1 (ppm)

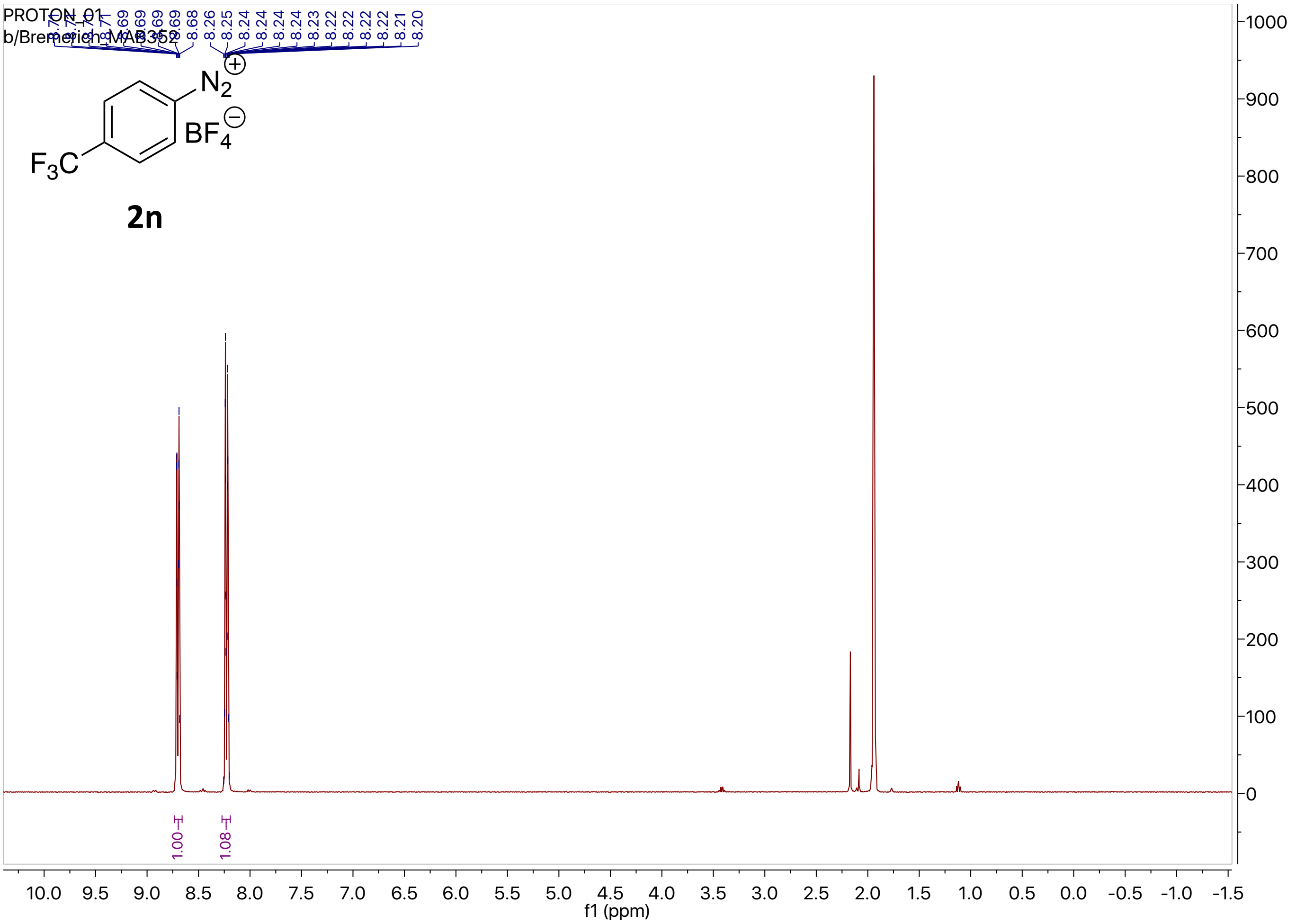
PROTON_01
b/Bremerich_MAB352
8.69 8.69 8.69 8.68 8.26 8.25 8.24 8.24 8.24 8.24 8.23 8.22 8.22 8.22 8.22 8.21 8.20
F_3C
$N_2^{\oplus}$
$BF_4^{\ominus}$
2n
1.00
1.08
f1 (ppm)

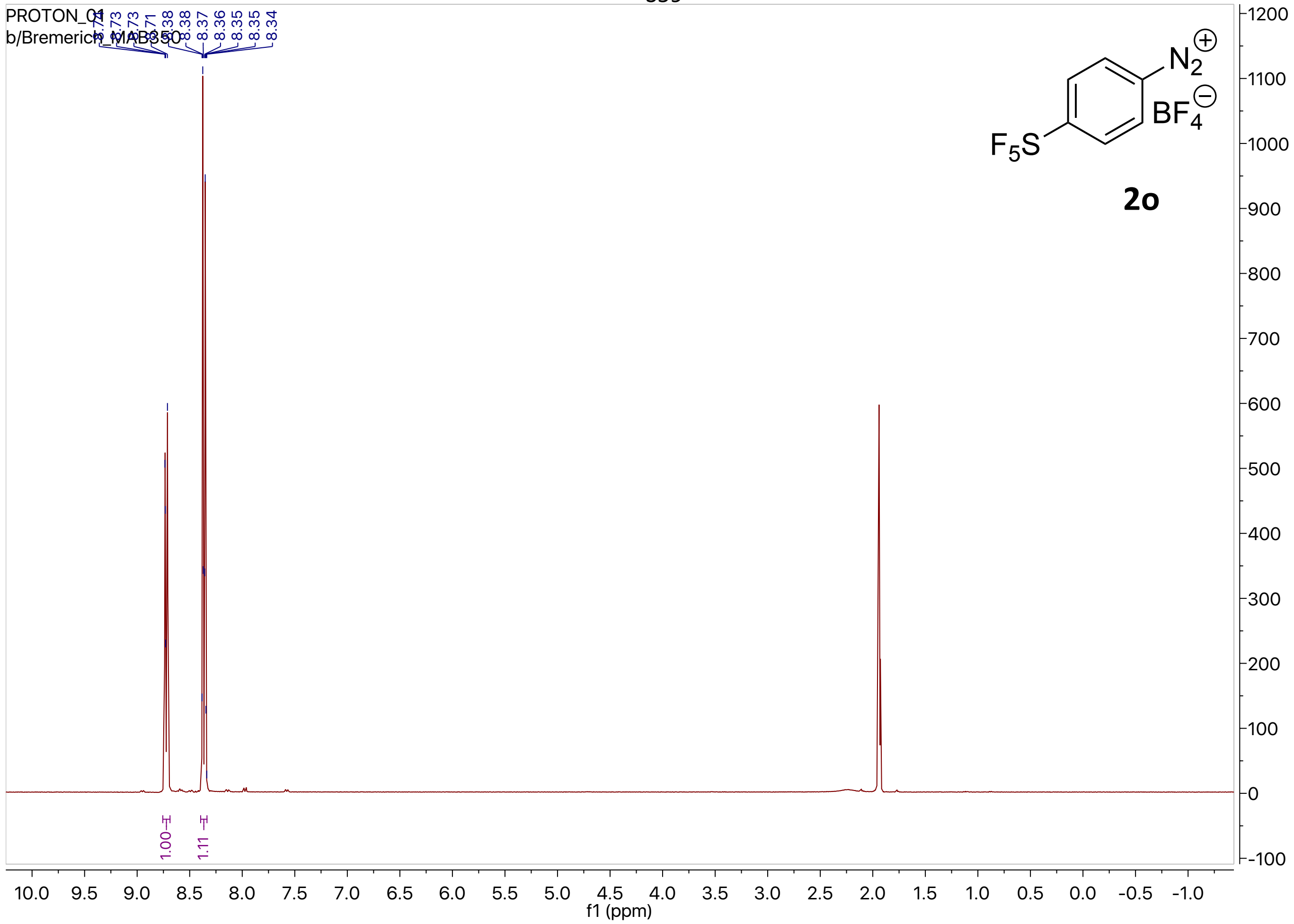
PROTON_01
b/Bremerich_MAB350
8.38
8.38
8.37
8.36
8.35
8.35
8.34
F_5S
$N_2^{\oplus}$
$BF_4^{\ominus}$
2o
1.00
1.11
f1 (ppm)

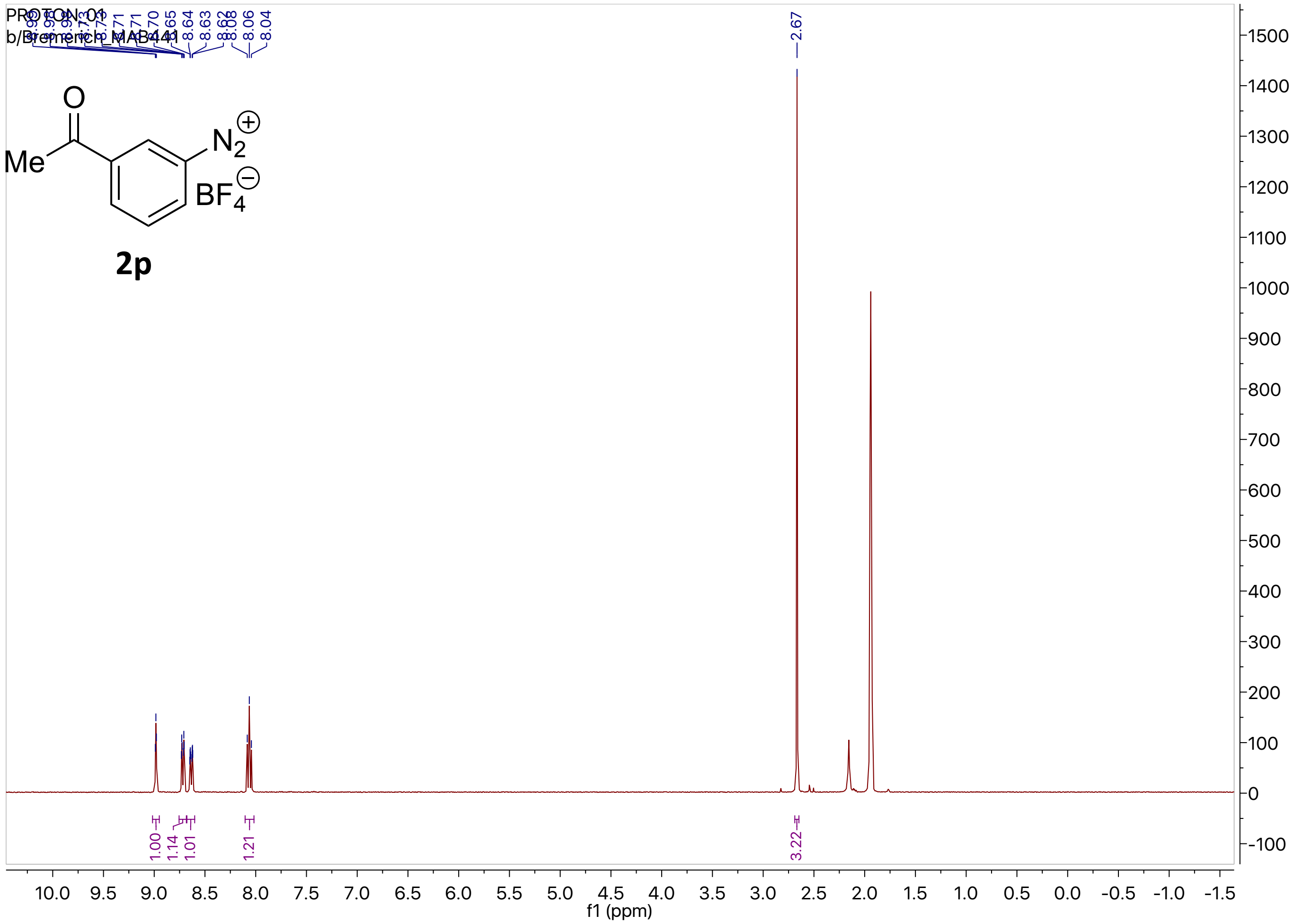
PROTON_01
b/Bremerich_MAB441
8.71
8.70
8.65
8.64
8.63
8.62
8.06
8.04
2.67
O
Me
$N_2^{\oplus}$
$BF_4^{\ominus}$
2p
1.00
1.14
1.01
1.21
3.22
f1 (ppm)

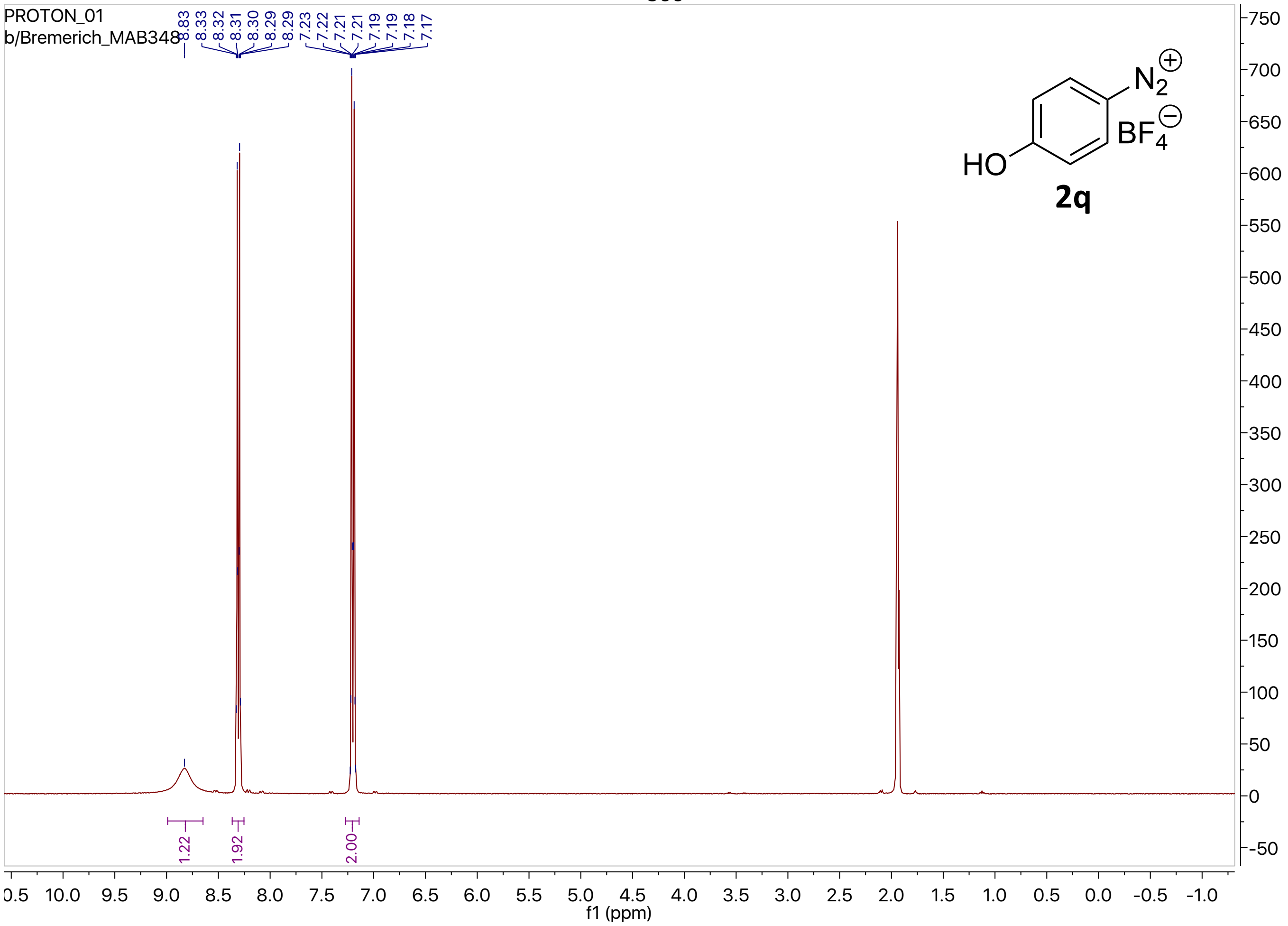
PROTON_01
b/Bremerich_MAB348
8.83
8.33
8.32
8.31
8.30
8.29
8.29
7.23
7.22
7.21
7.21
7.19
7.19
7.18
7.17
HO
$N_2^{\oplus}$
$BF_4^{\ominus}$
2q
1.22
1.92
2.00
f1 (ppm)

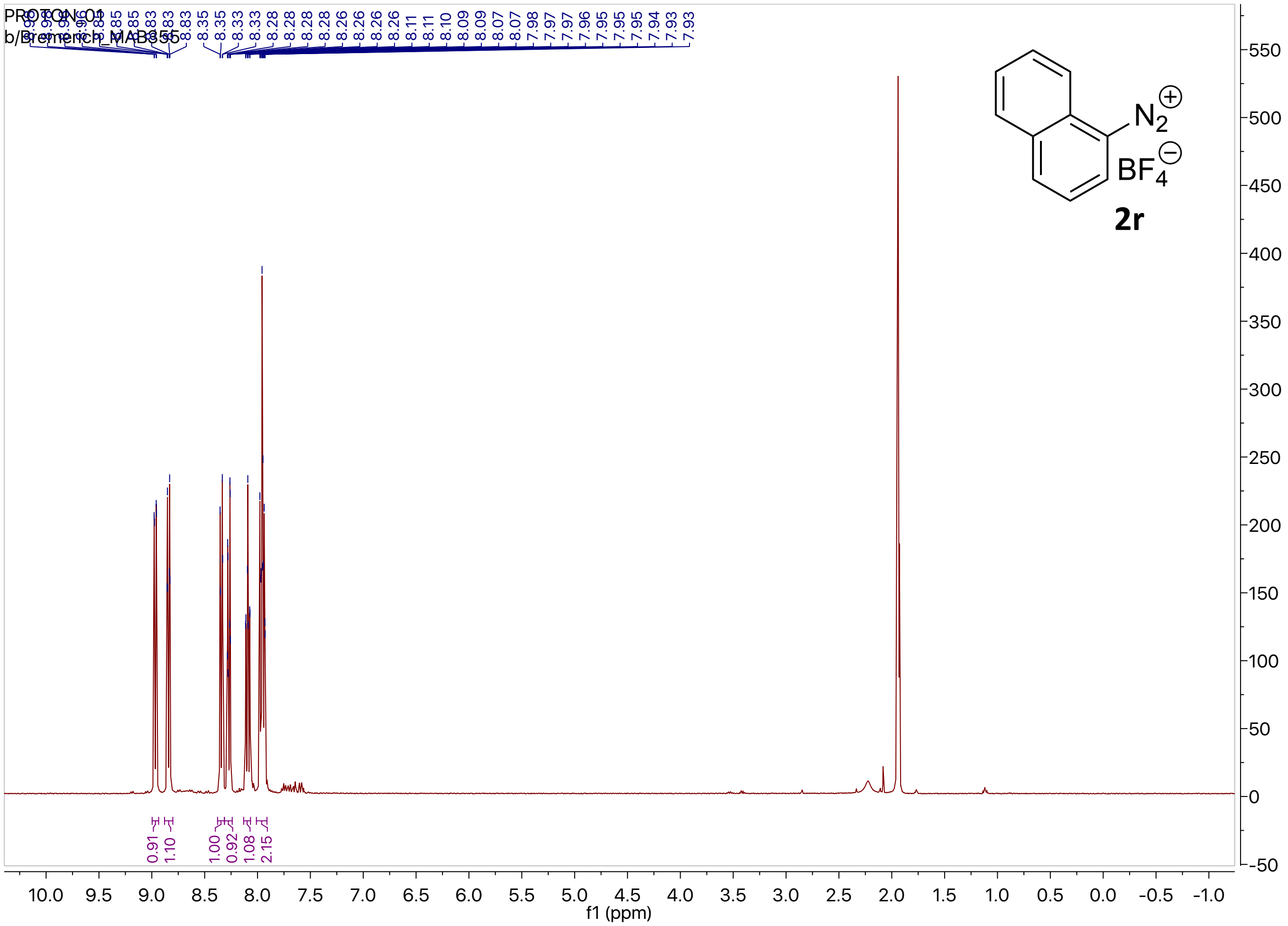
PROTON_01
b/Bremerich_MAB355
8.85
8.85
8.83
8.83
8.35
8.35
8.33
8.33
8.28
8.28
8.28
8.26
8.26
8.26
8.11
8.11
8.10
8.09
8.07
8.07
7.98
7.97
7.96
7.95
7.95
7.94
7.93
7.93
$N_2^{\oplus}$
$BF_4^{\ominus}$
2r
0.91
1.10
1.00
0.92
1.08
2.15
f1 (ppm)

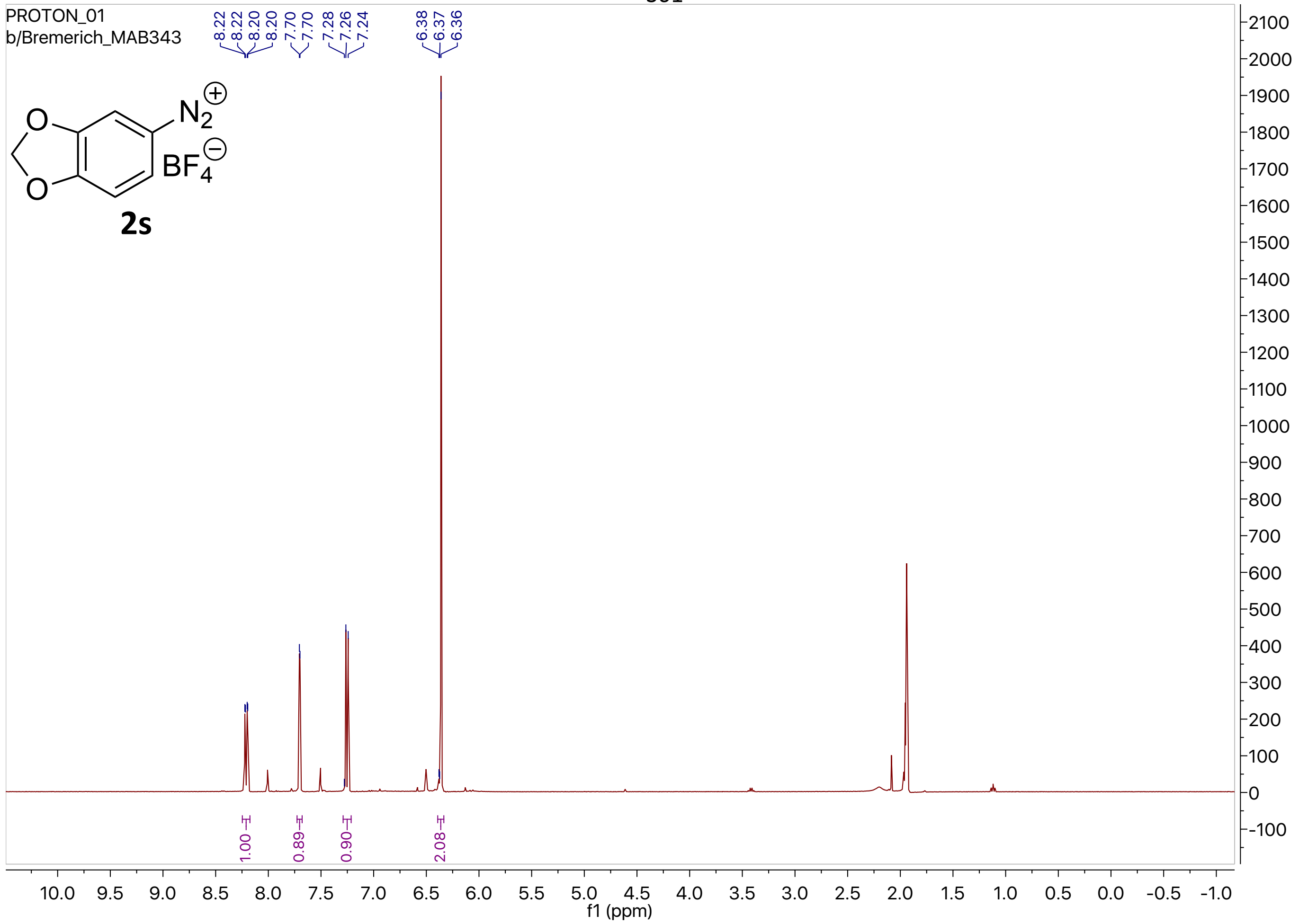
PROTON_01
b/Bremerich_MAB343
8.22
8.22
8.20
8.20
7.70
7.70
7.28
7.26
7.24
6.38
6.37
6.36
2s
1.00
0.89
0.90
2.08
f1 (ppm)

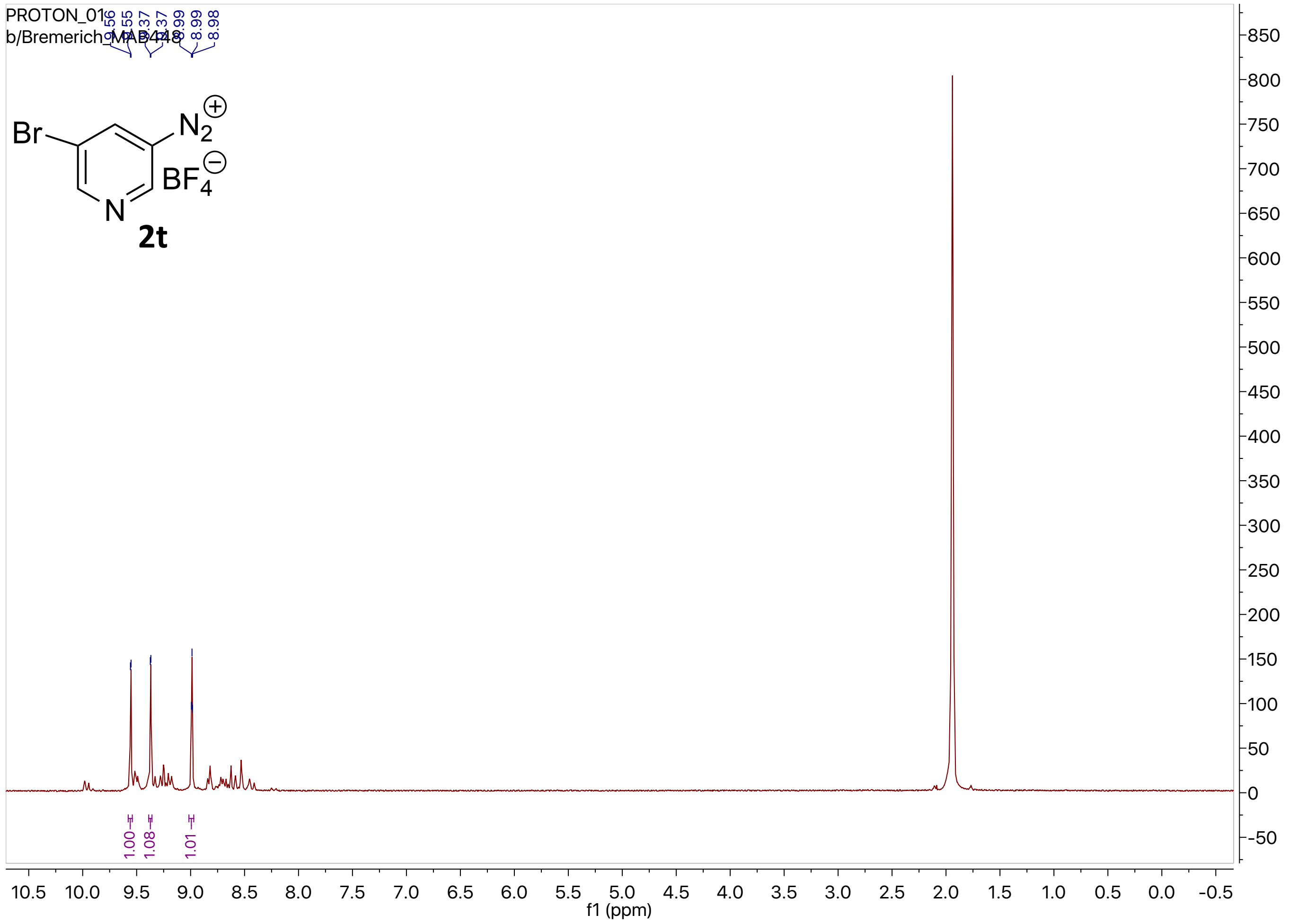
PROTON_01
b/Bremerich_MAB448
9.55
9.37
9.37
8.99
8.99
8.98
2t
1.00
1.08
1.01
f1 (ppm)

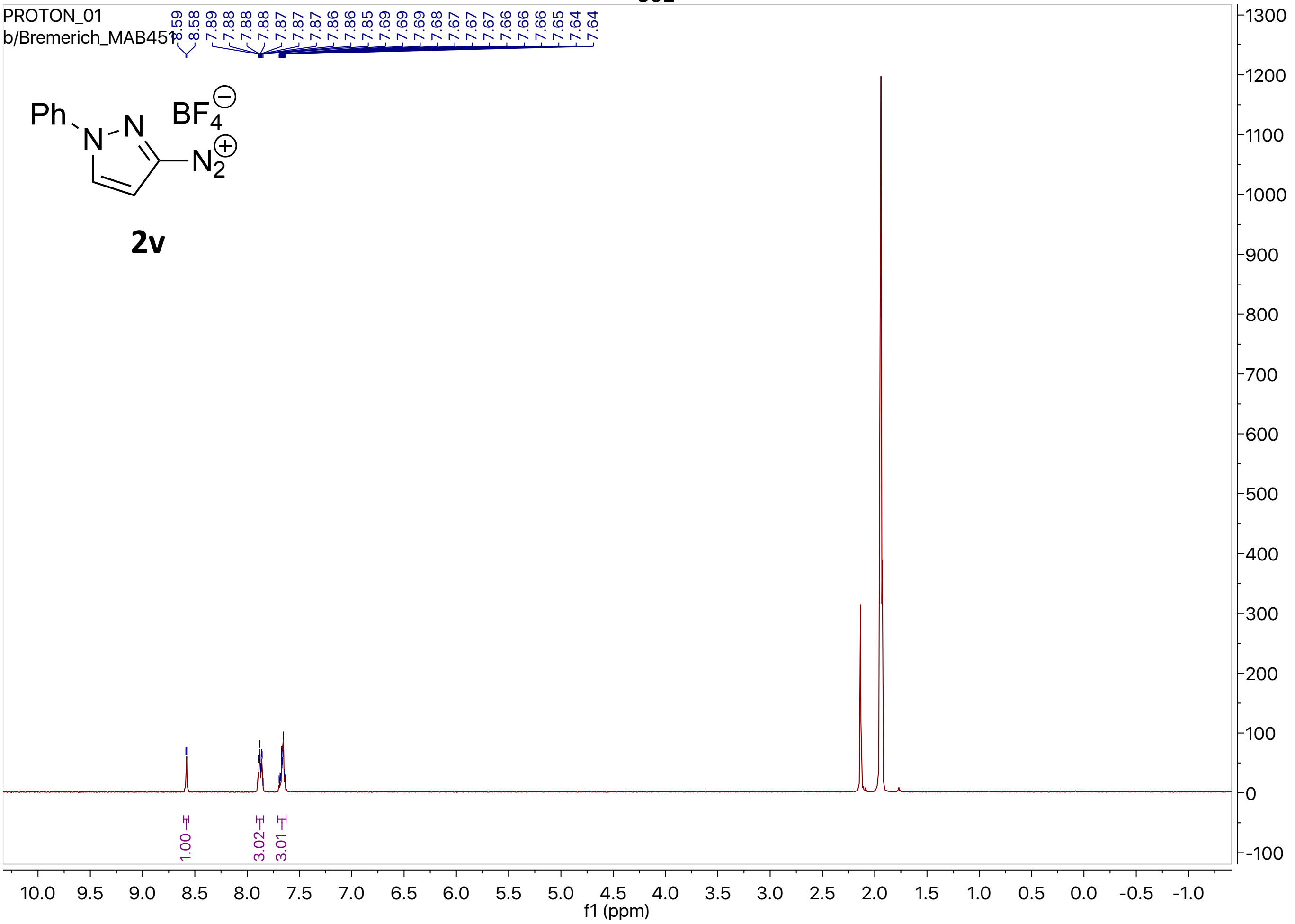
PROTON_01
b/Bremerich_MAB451
8.59
8.58
7.89
7.88
7.88
7.88
7.87
7.87
7.86
7.86
7.85
7.69
7.69
7.69
7.68
7.67
7.67
7.67
7.66
7.66
7.66
7.65
7.64
7.64
Ph
N
N
BF4
N2
2v
1.00
3.02
3.01
f1 (ppm)

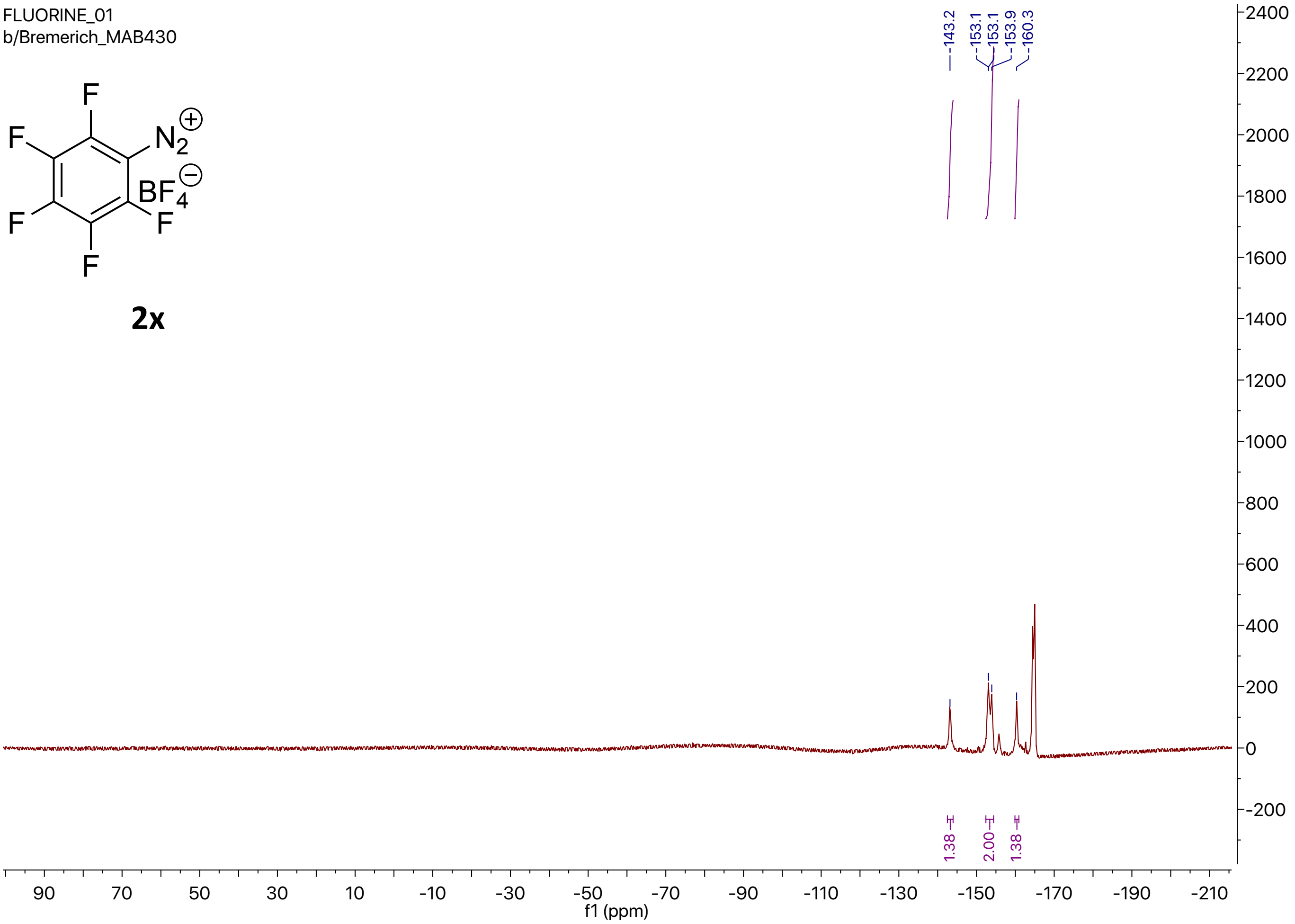
FLUORINE_01
b/Bremerich_MAB430
-143.2
-153.1
-153.1
-153.9
-160.3
F
N2
BF4
2x
1.38
2.00
1.38
f1 (ppm)

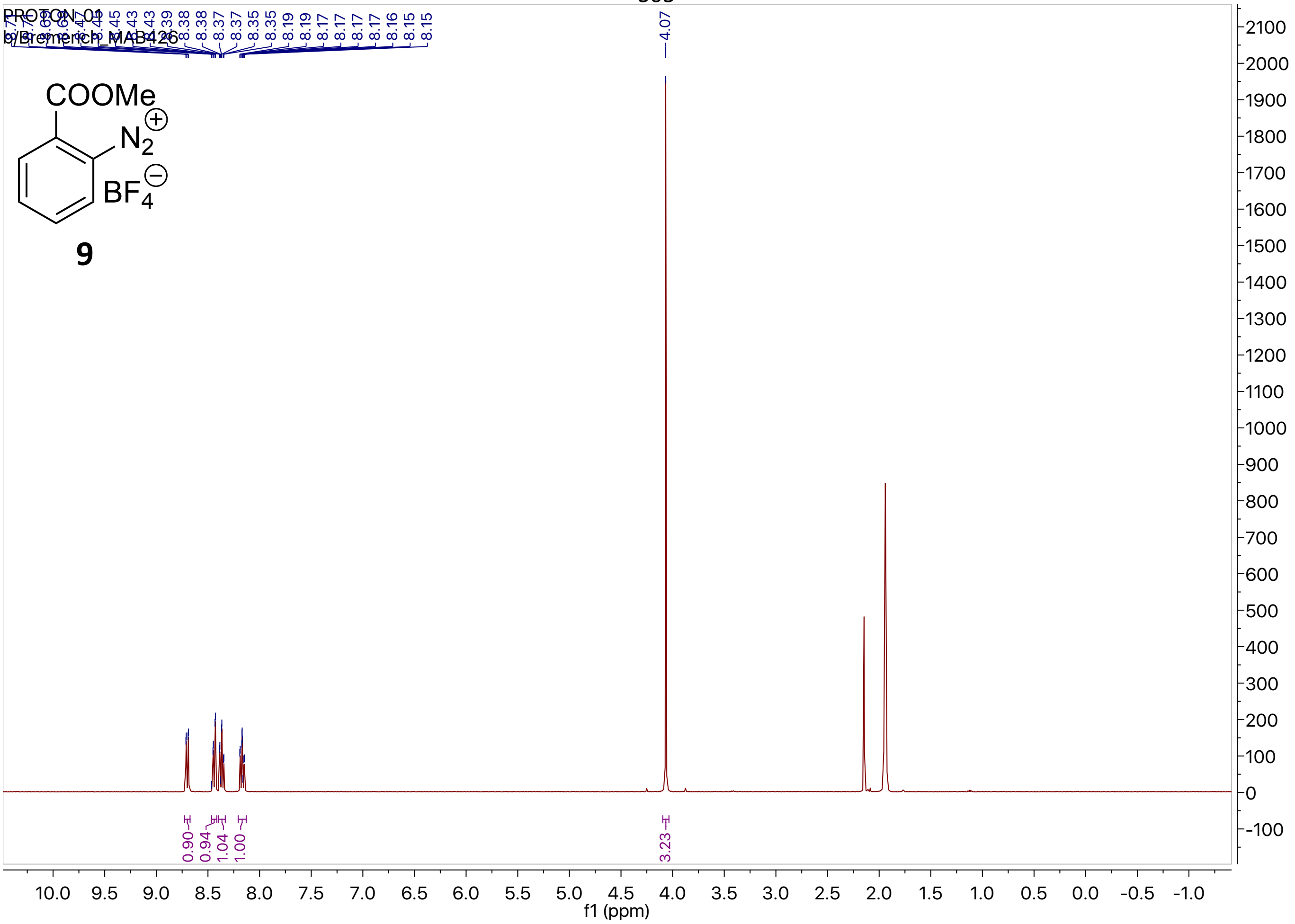

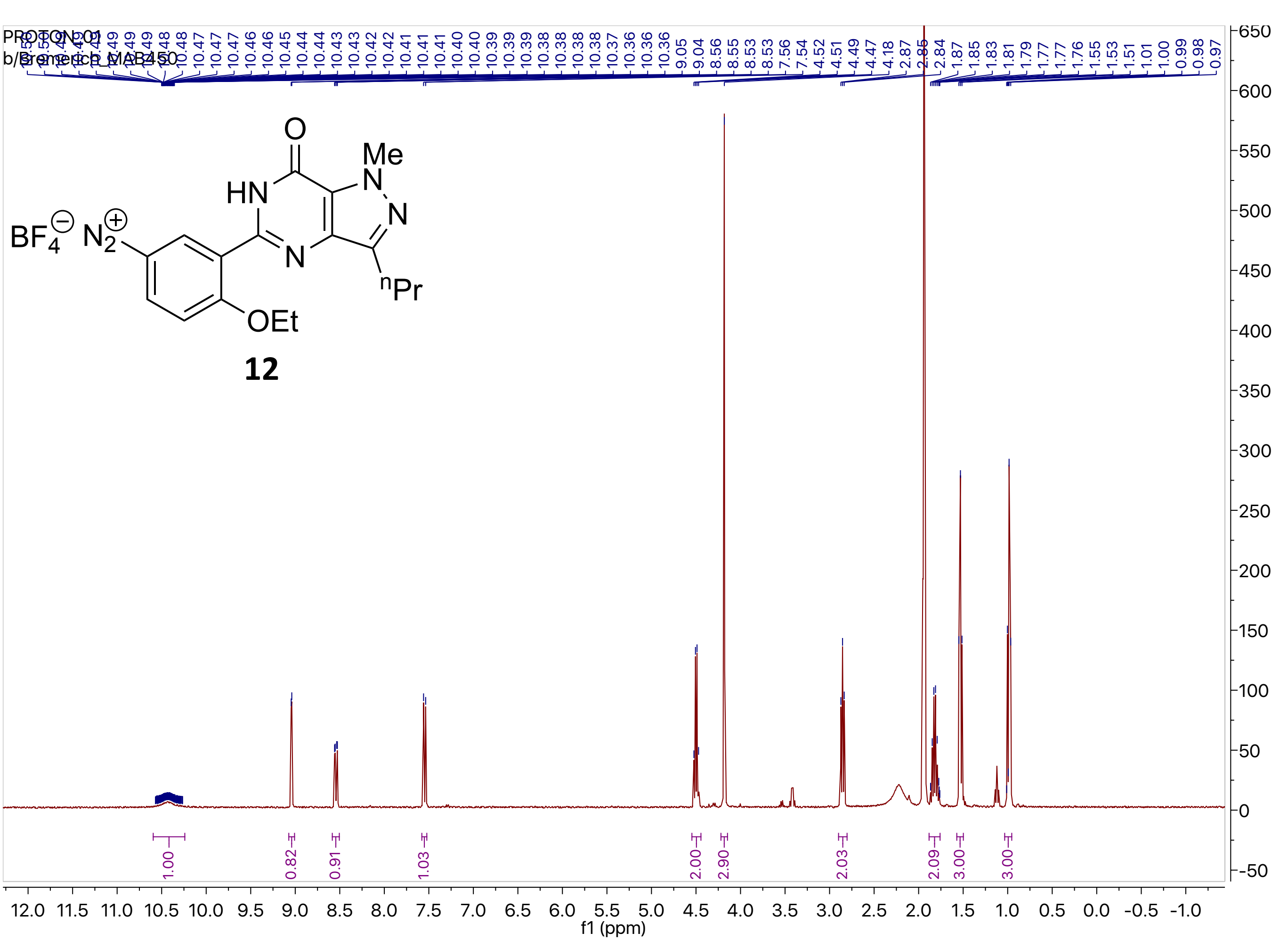

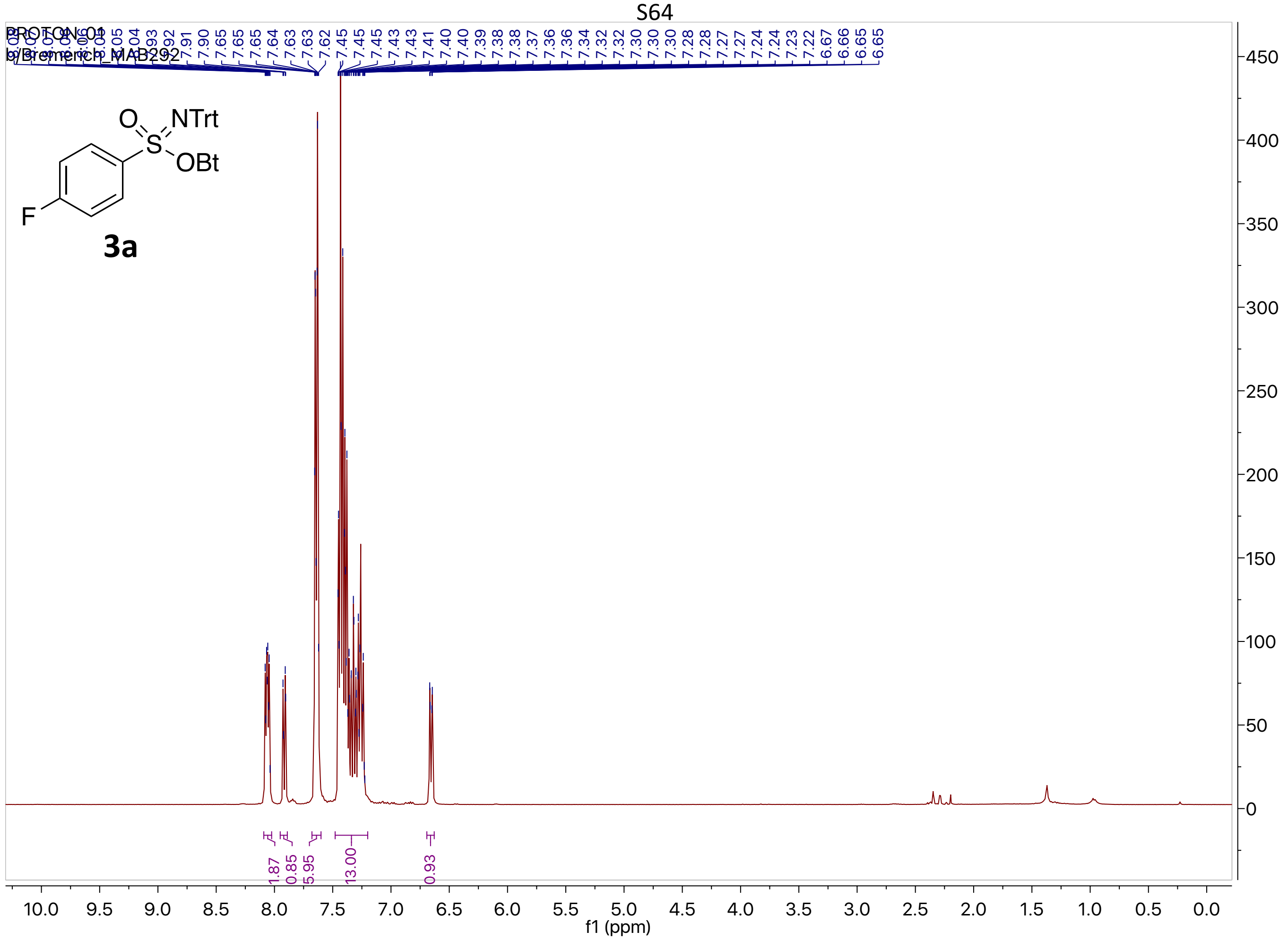
PROTON_01
b/Bremerich_MAB292
O
NTrt
S
OBt
F
3a
1.87
0.85
5.95
13.00
0.93
f1 (ppm)

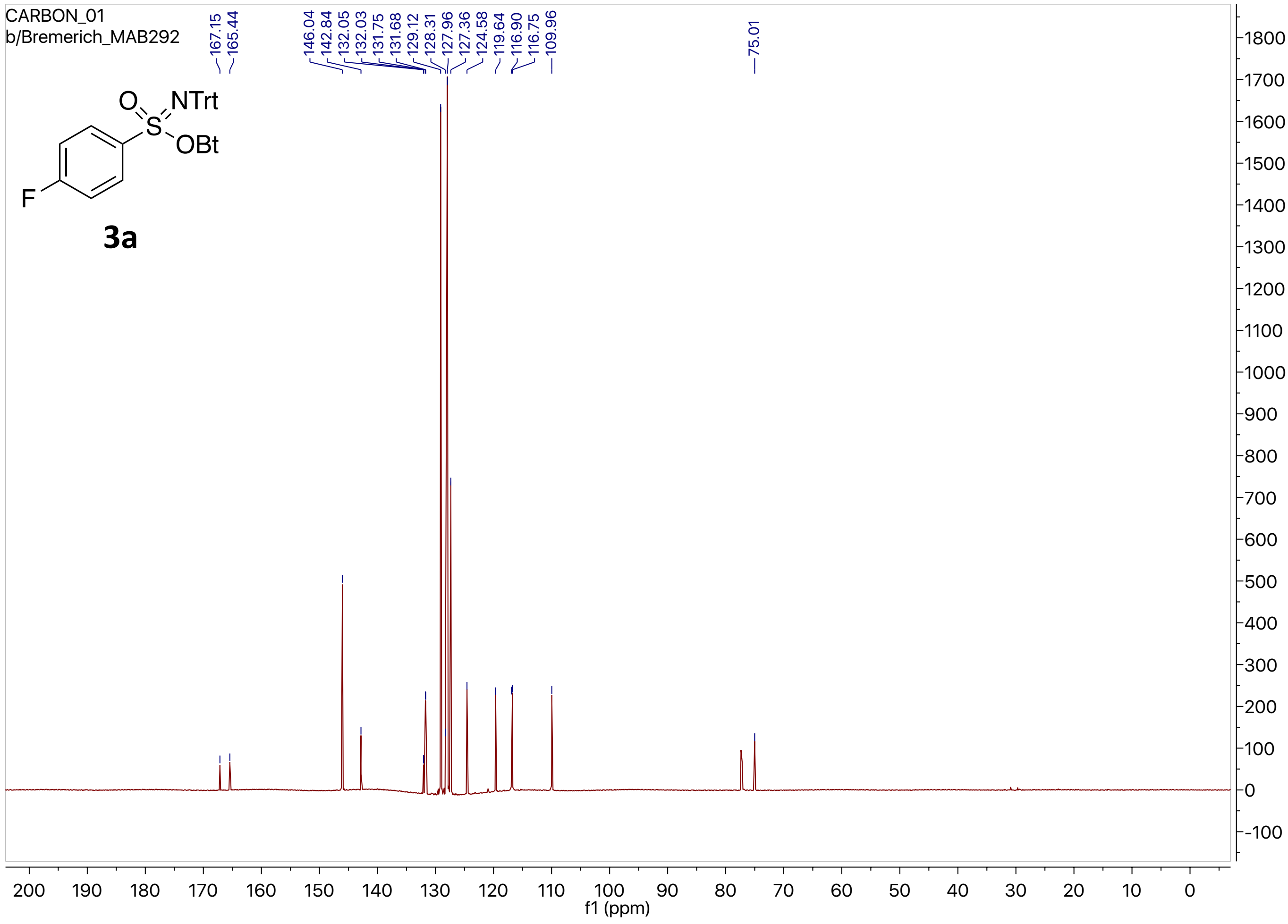
CARBON_01
b/Bremerich_MAB292
167.15
165.44
146.04
142.84
132.05
132.03
131.75
131.68
129.12
128.31
127.96
127.36
124.58
119.64
116.90
116.75
109.96
75.01
O
NTrt
S
OBt
F
3a
f1 (ppm)

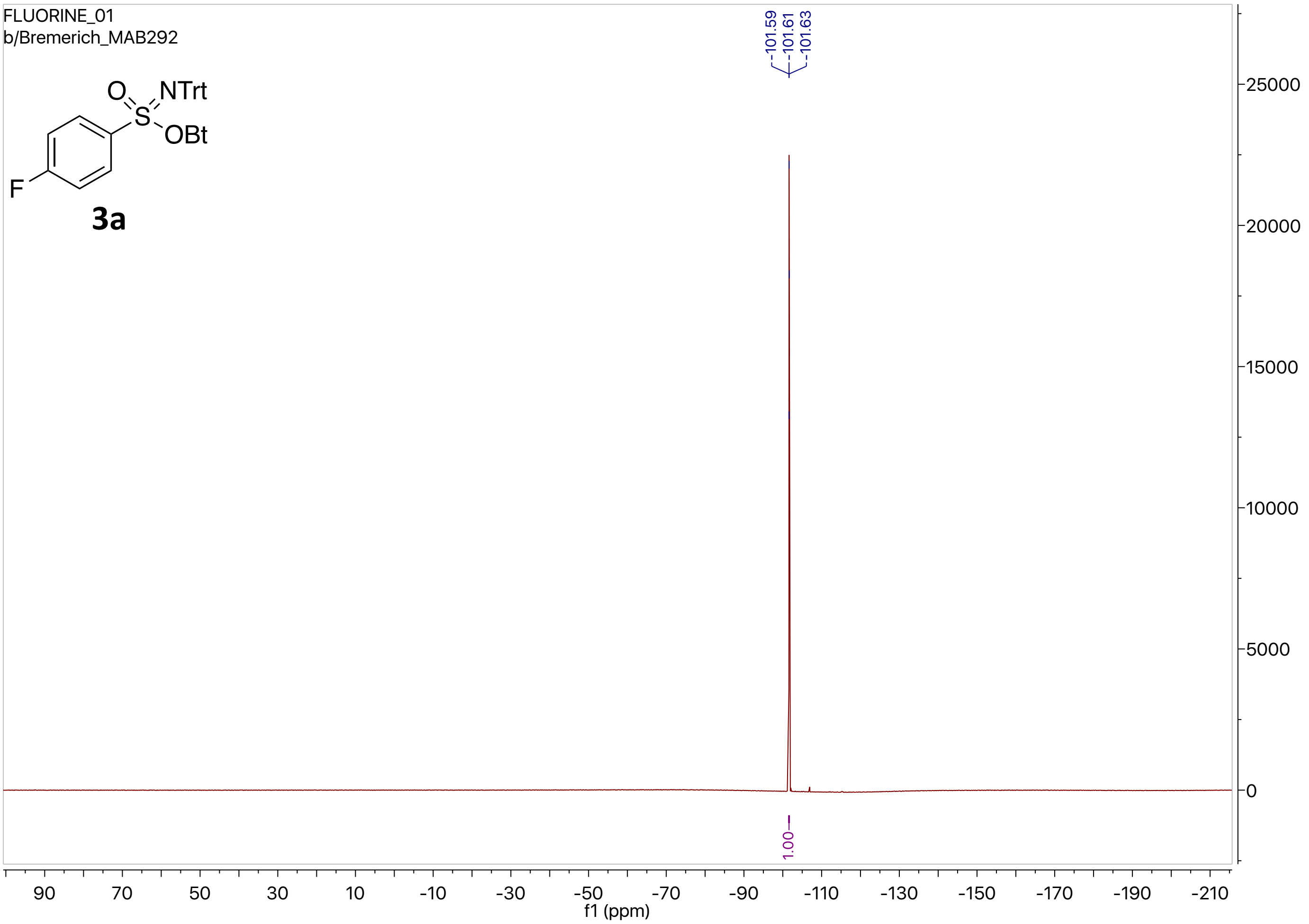
FLUORINE_01
b/Bremerich_MAB292
NTrt
O
S
OBt
F
-101.59
-101.61
-101.63
25000
20000
15000
10000
5000
0
1.00
90
70
50
30
10
-10
-30
-50
-70
-90
-110
-130
-150
-170
-190
-210
f1 (ppm)

3a

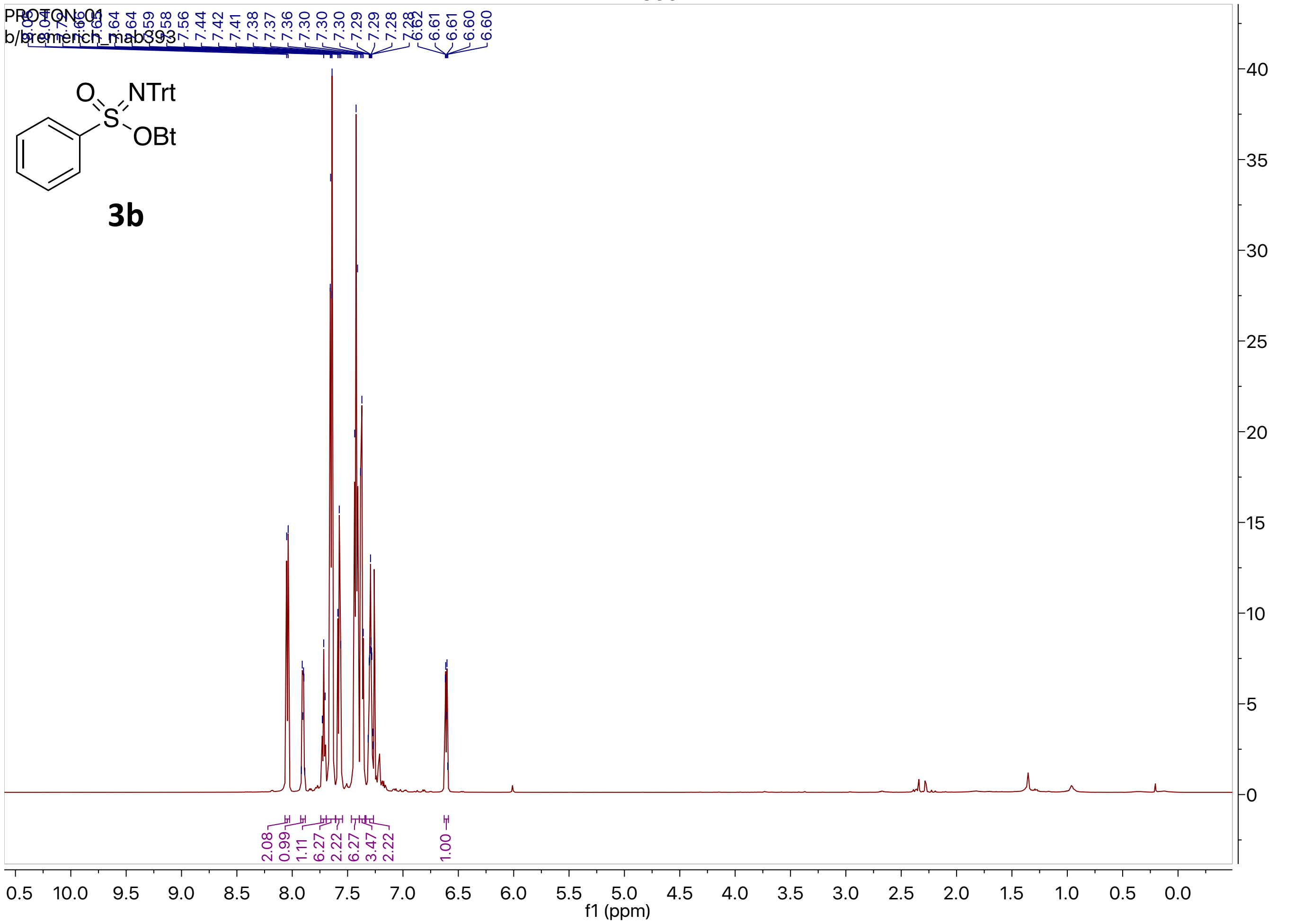
PROTON_01
b/bremerich_mab393
NTrt
OBt
3b
f1 (ppm)

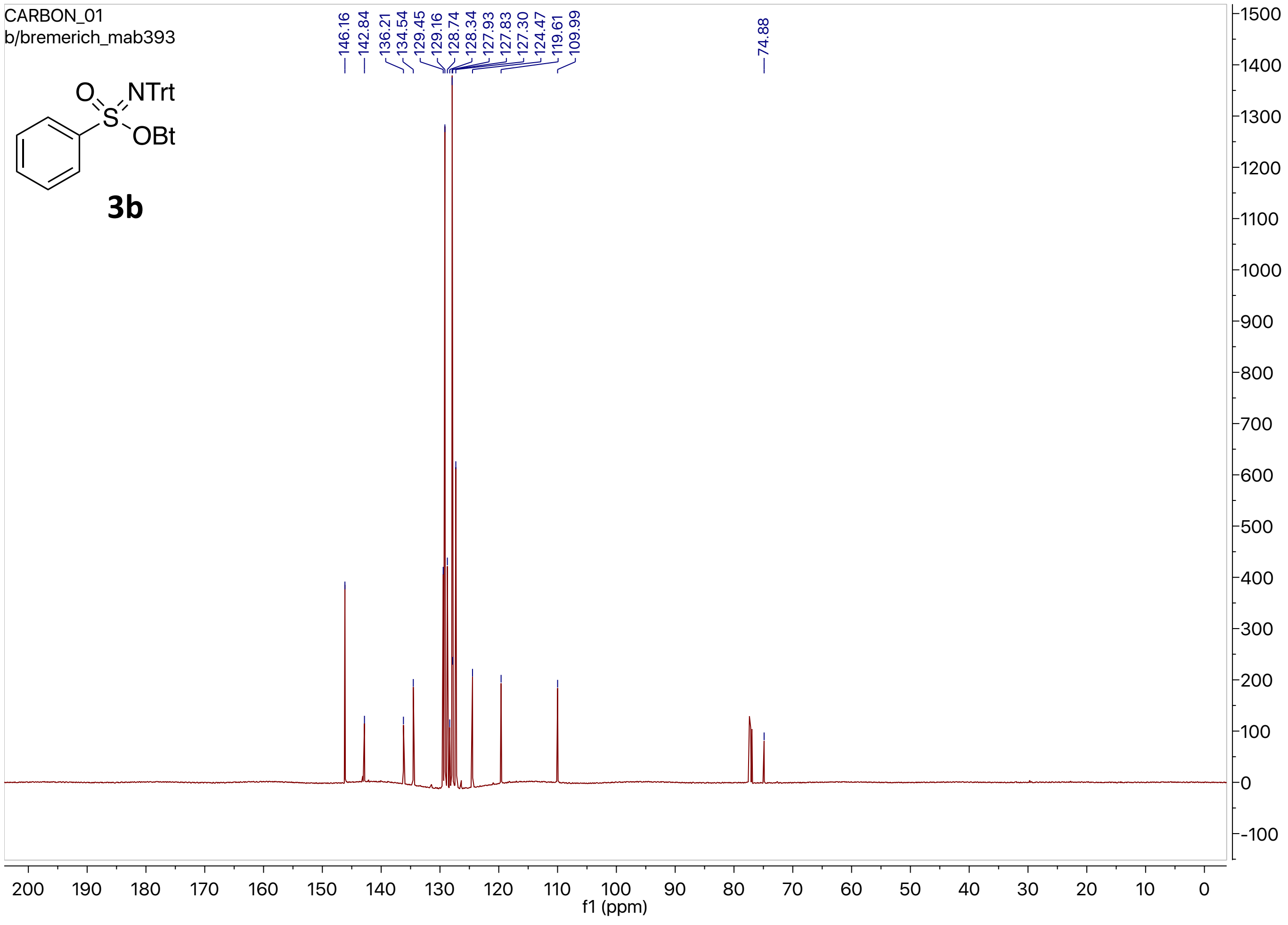
CARBON_01
b/bremerich_mab393
146.16
142.84
136.21
134.54
129.45
129.16
128.74
128.34
127.93
127.83
127.30
124.47
119.61
109.99
74.88
NTrt
OBt
3b
f1 (ppm)

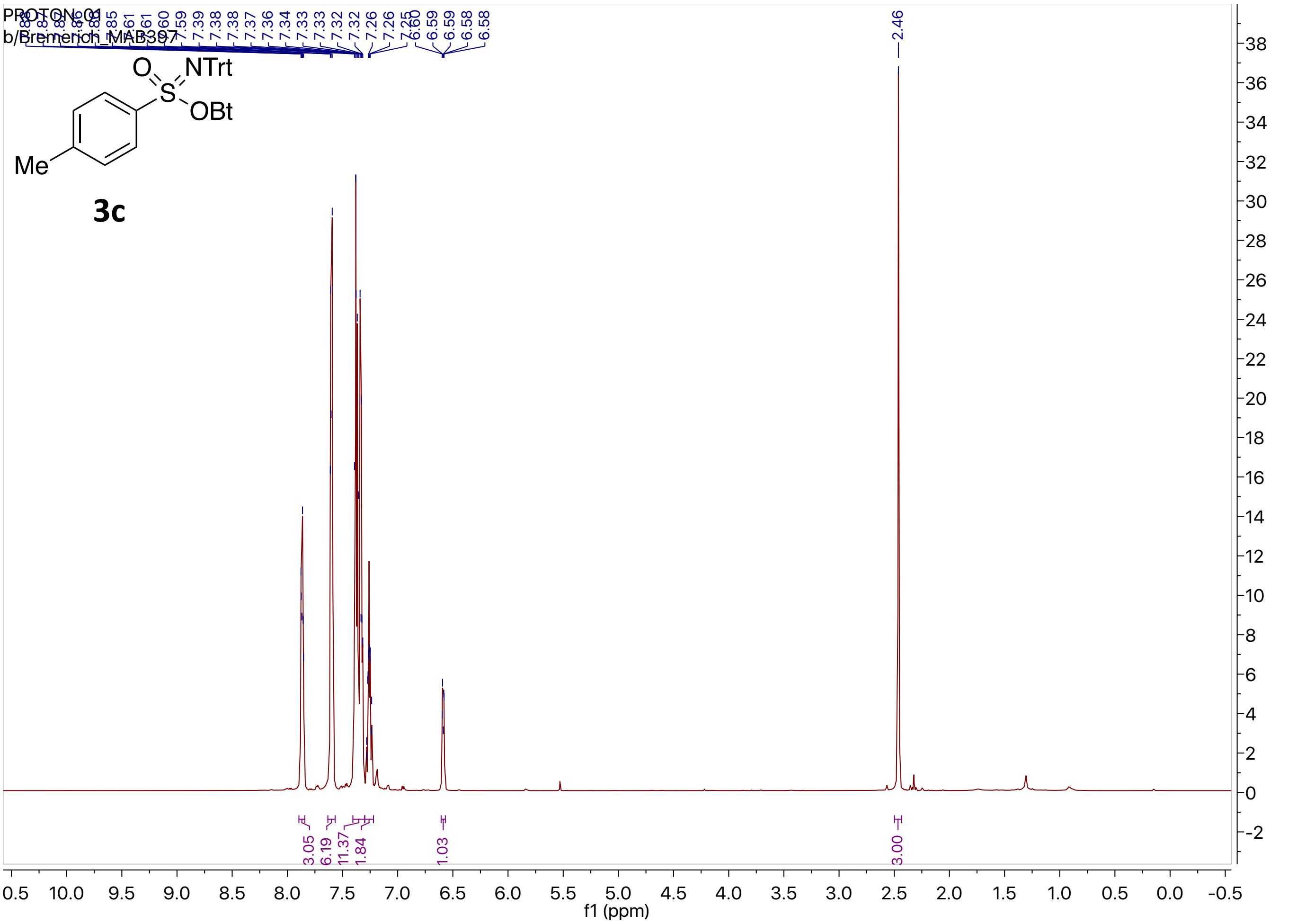
PROTON_01
b/Bremerich_MAB397
O
NTrt
S
OBt
Me
3c
7.61
7.61
7.60
7.59
7.39
7.38
7.38
7.37
7.36
7.34
7.33
7.33
7.32
7.32
7.26
7.26
7.25
6.60
6.59
6.59
6.58
6.58
2.46
3.05
6.19
11.37
1.84
1.03
3.00
f1 (ppm)

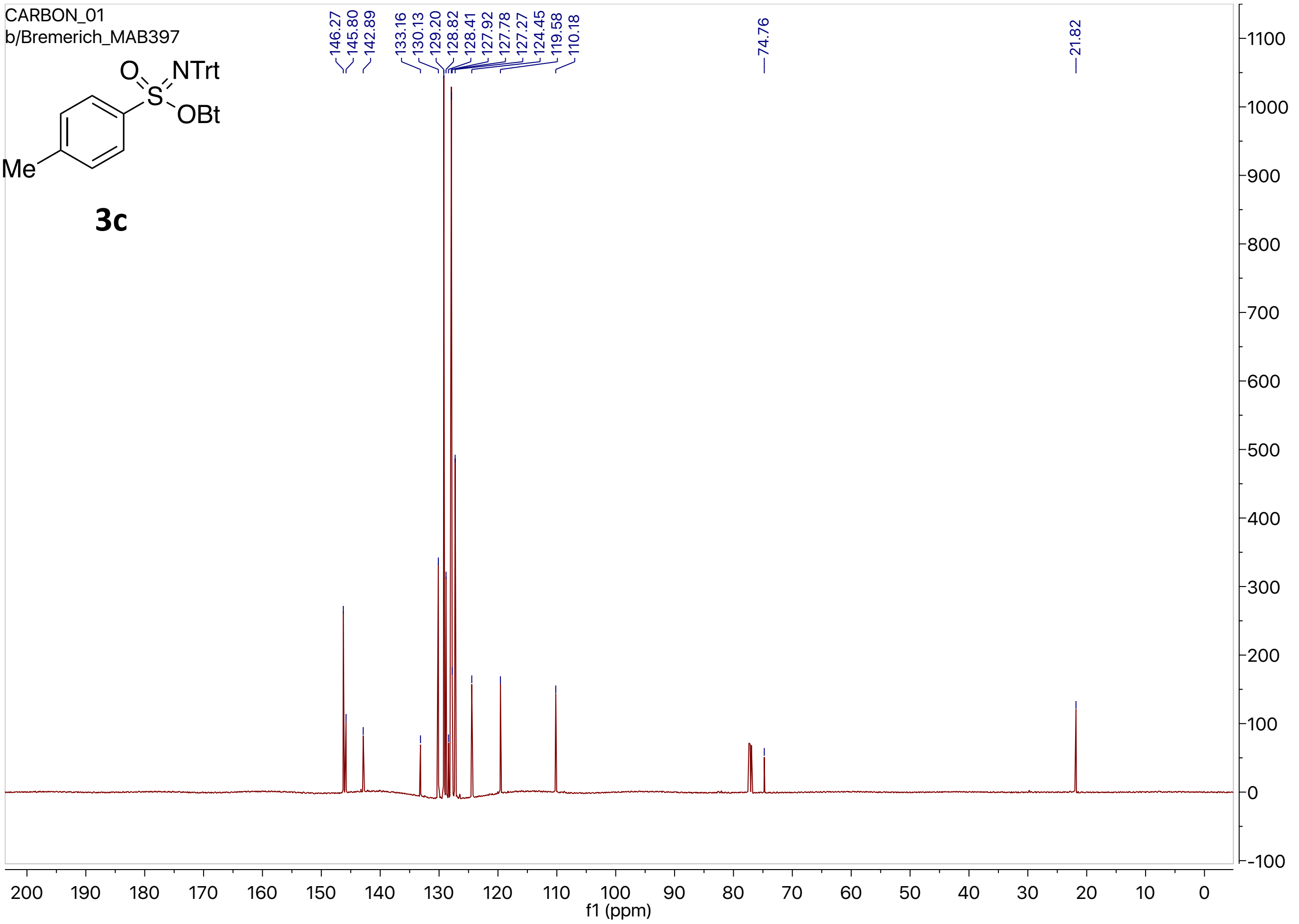
CARBON_01
b/Bremerich_MAB397
O
NTrt
S
OBt
Me
3c
146.27
145.80
142.89
133.16
130.13
129.20
128.82
128.41
127.92
127.78
127.27
124.45
119.58
110.18
74.76
21.82
f1 (ppm)

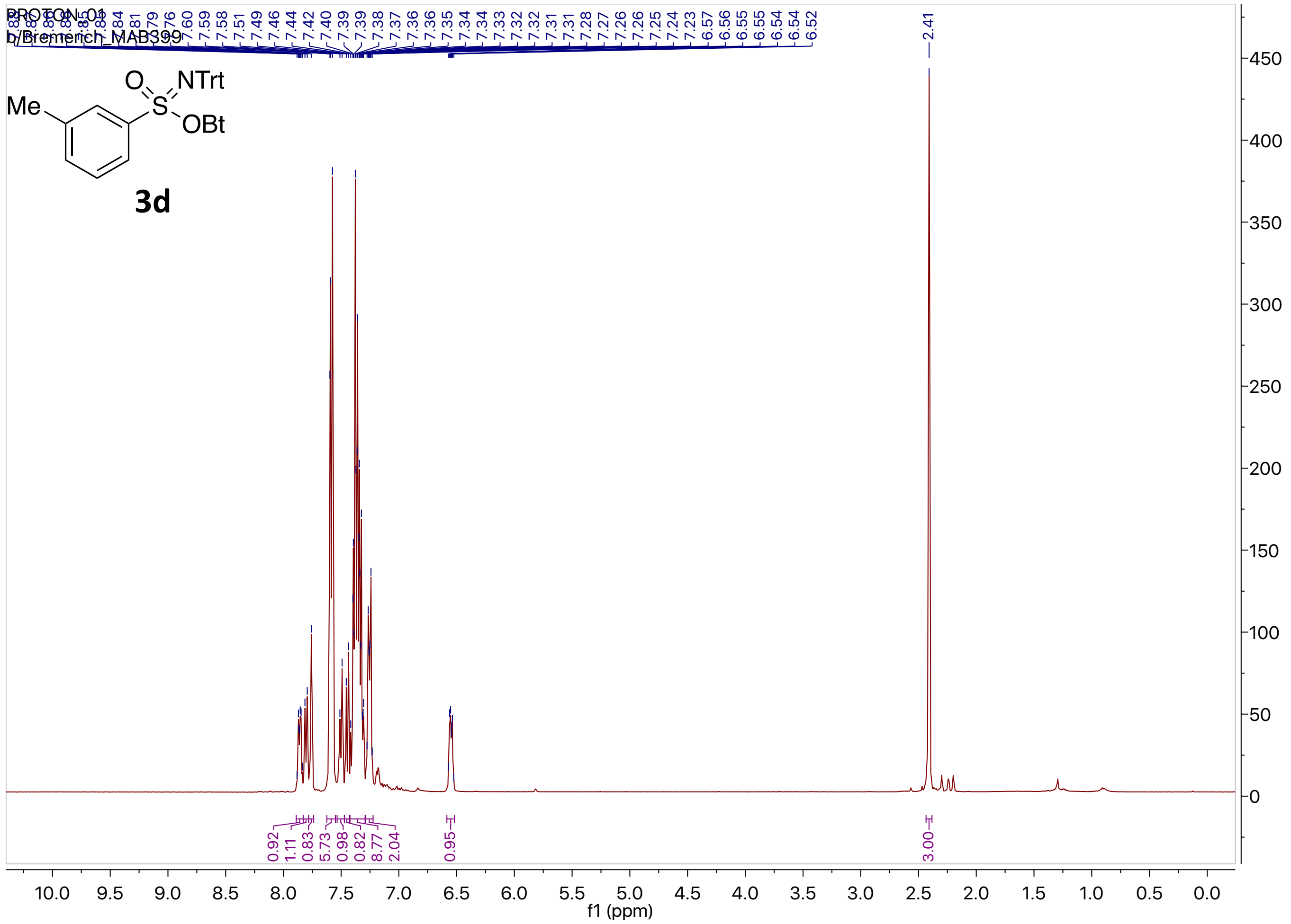
PROTON_01
b/Bremerich_MAB399
7.84
7.81
7.79
7.76
7.60
7.59
7.58
7.51
7.49
7.46
7.44
7.42
7.40
7.39
7.39
7.38
7.37
7.36
7.36
7.35
7.34
7.34
7.33
7.32
7.32
7.31
7.28
7.27
7.26
7.26
7.25
7.24
7.23
6.57
6.56
6.55
6.55
6.54
6.54
6.52
2.41
O
NTrt
Me
S
OBt
3d
0.92
1.11
0.83
5.73
0.98
0.82
8.77
2.04
0.95
3.00
f1 (ppm)

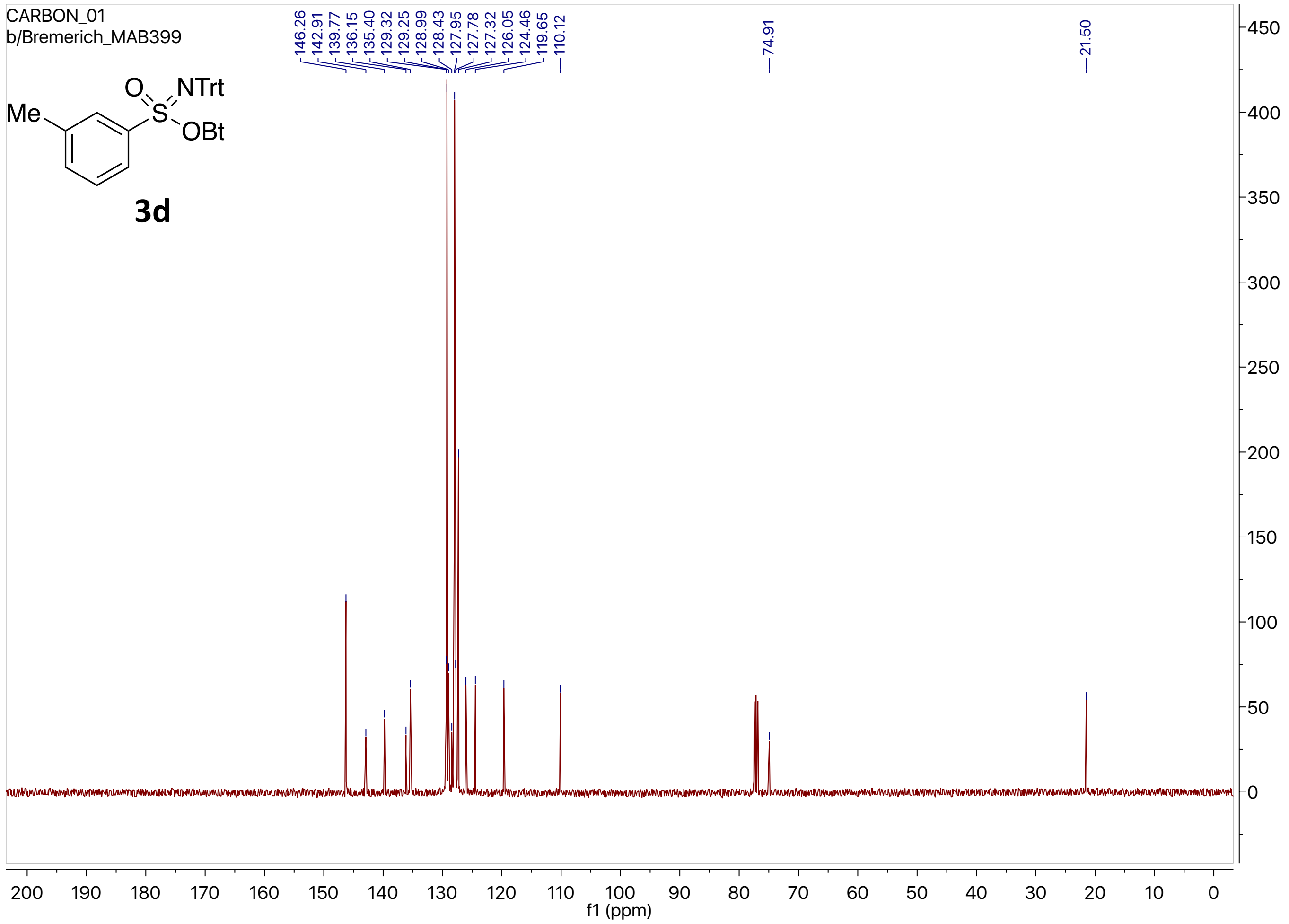
CARBON_01
b/Bremerich_MAB399
146.26
142.91
139.77
136.15
135.40
129.32
129.25
128.99
128.43
127.95
127.78
127.32
126.05
124.46
119.65
110.12
74.91
21.50
O
NTrt
Me
S
OBt
3d
f1 (ppm)

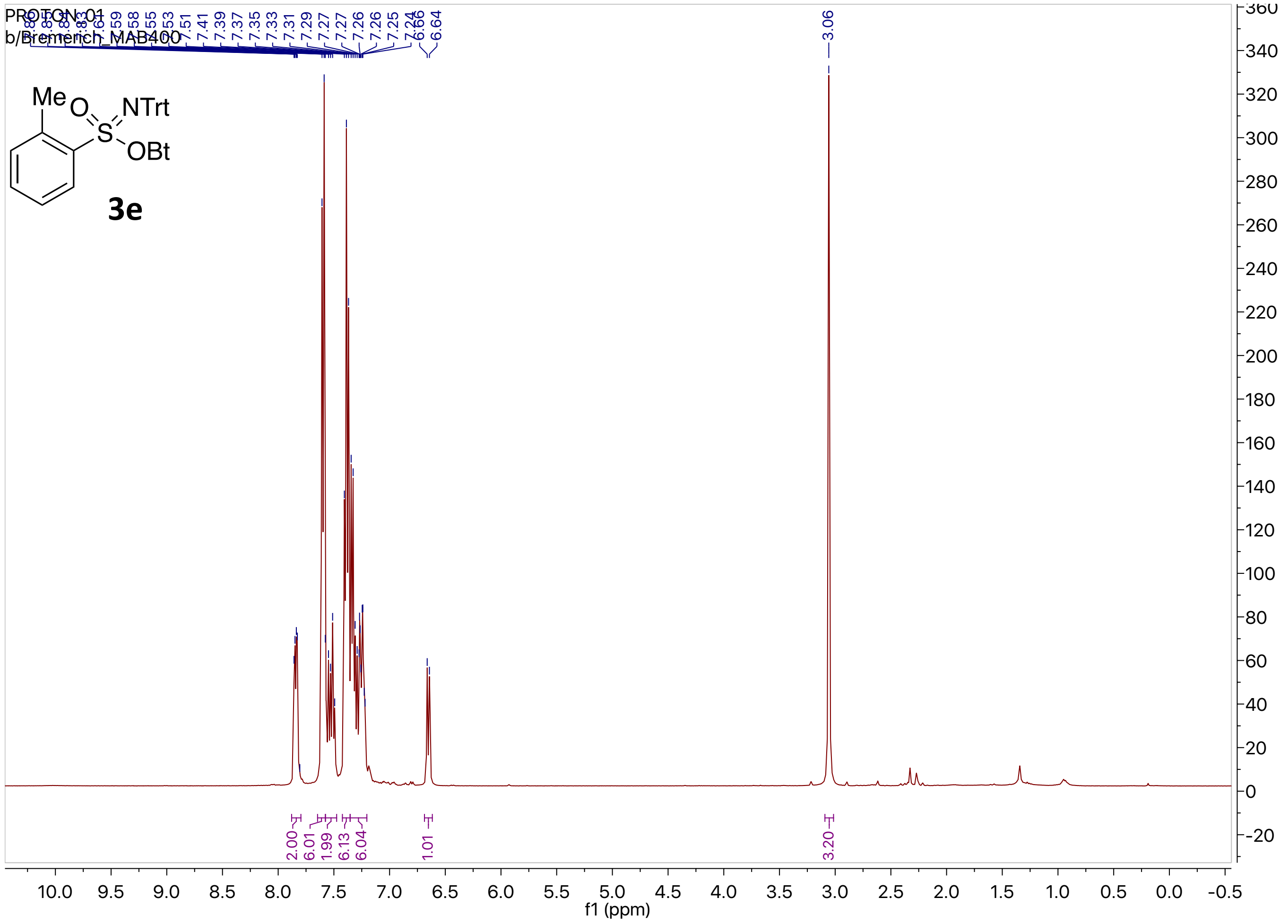
PROTON_01
b/Bremerich_MAB400
7.59 7.58 7.55 7.53 7.51 7.41 7.39 7.37 7.35 7.33 7.31 7.29 7.27 7.27 7.26 7.26 7.25 7.24 6.66 6.64
3.06
Me O NTrt S OBt
3e
2.00 6.01 1.99 6.13 6.04 1.01 3.20
f1 (ppm)

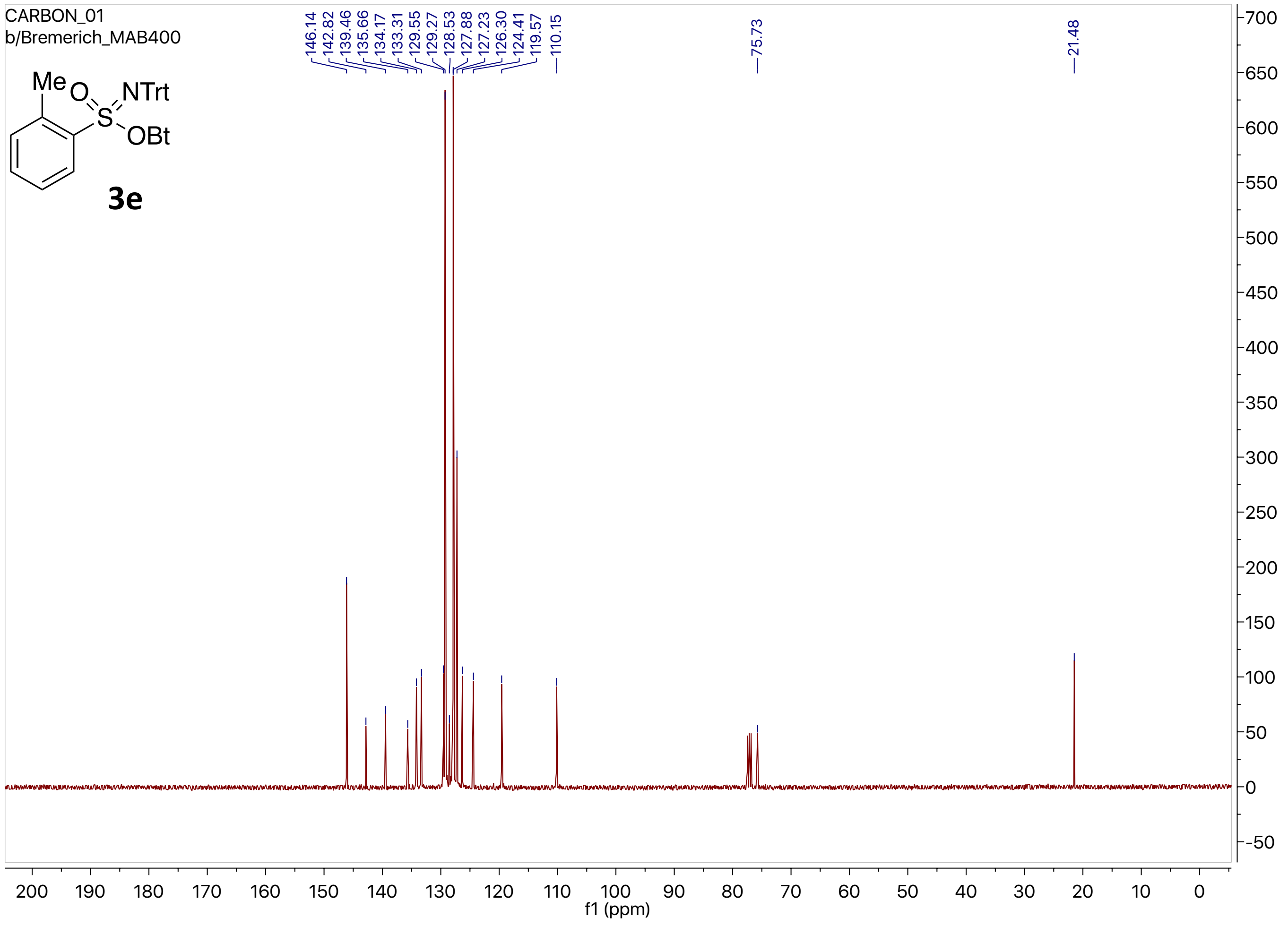
CARBON_01
b/Bremerich_MAB400
146.14 142.82 139.46 135.66 134.17 133.31 129.55 129.27 128.53 127.88 127.23 126.30 124.41 119.57 110.15
75.73
21.48
Me O NTrt S OBt
3e
f1 (ppm)

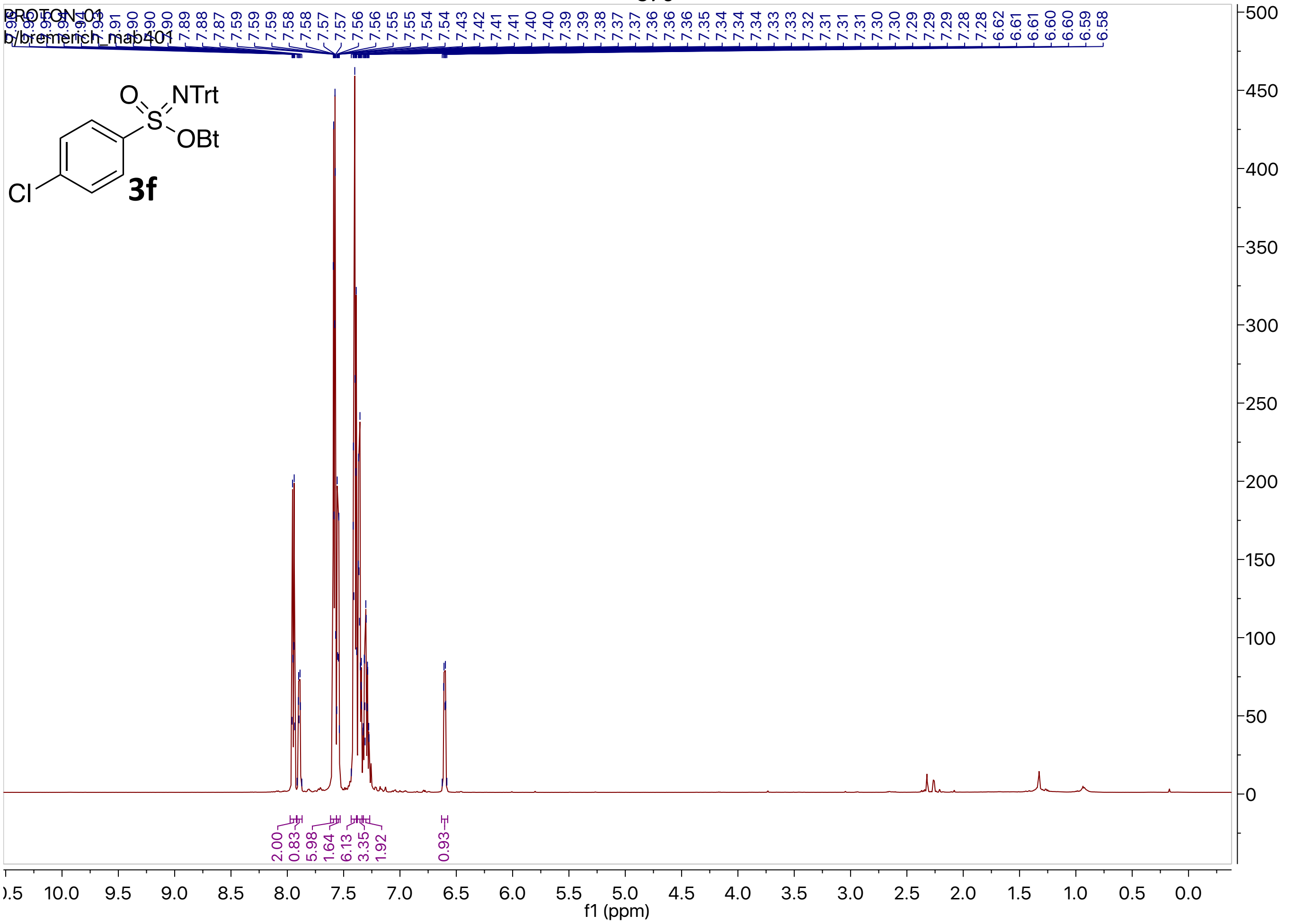
PROTON_01
b/bremerich_mab401
7.91
7.90
7.90
7.90
7.89
7.88
7.87
7.59
7.59
7.59
7.58
7.58
7.57
7.57
7.56
7.56
7.55
7.55
7.54
7.54
7.43
7.42
7.41
7.41
7.40
7.40
7.39
7.39
7.38
7.37
7.37
7.36
7.36
7.36
7.35
7.34
7.34
7.34
7.33
7.33
7.32
7.31
7.31
7.31
7.30
7.29
7.29
7.29
7.28
7.28
6.62
6.61
6.61
6.60
6.60
6.59
6.58
O
S
NTrt
OBt
Cl
3f
2.00
0.83
5.98
1.64
6.13
3.35
1.92
0.93
f1 (ppm)

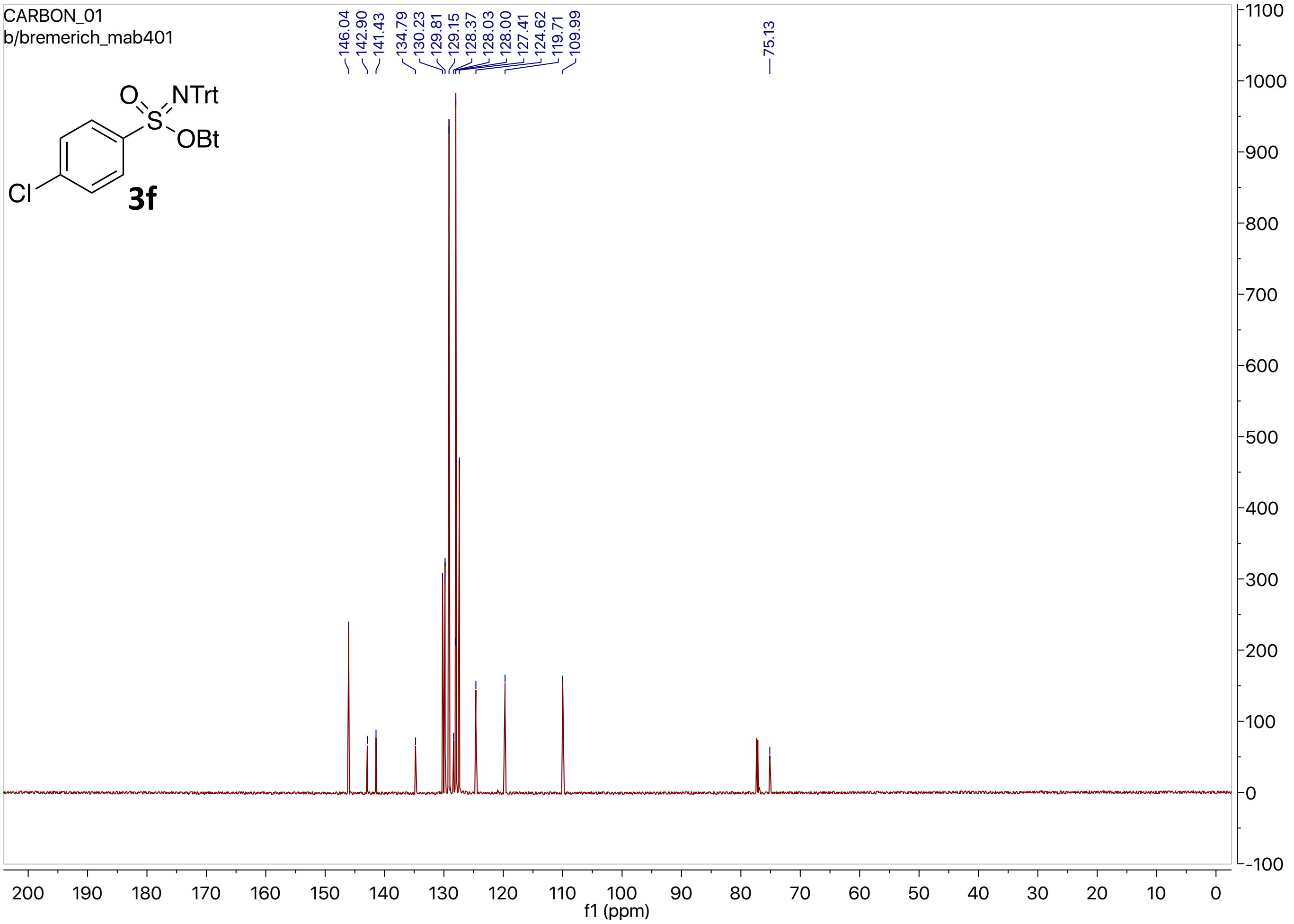
CARBON_01
b/bremerich_mab401
146.04
142.90
141.43
134.79
130.23
129.81
129.15
128.37
128.03
128.00
127.41
124.62
119.71
109.99
75.13
O
S
NTrt
OBt
Cl
3f
f1 (ppm)

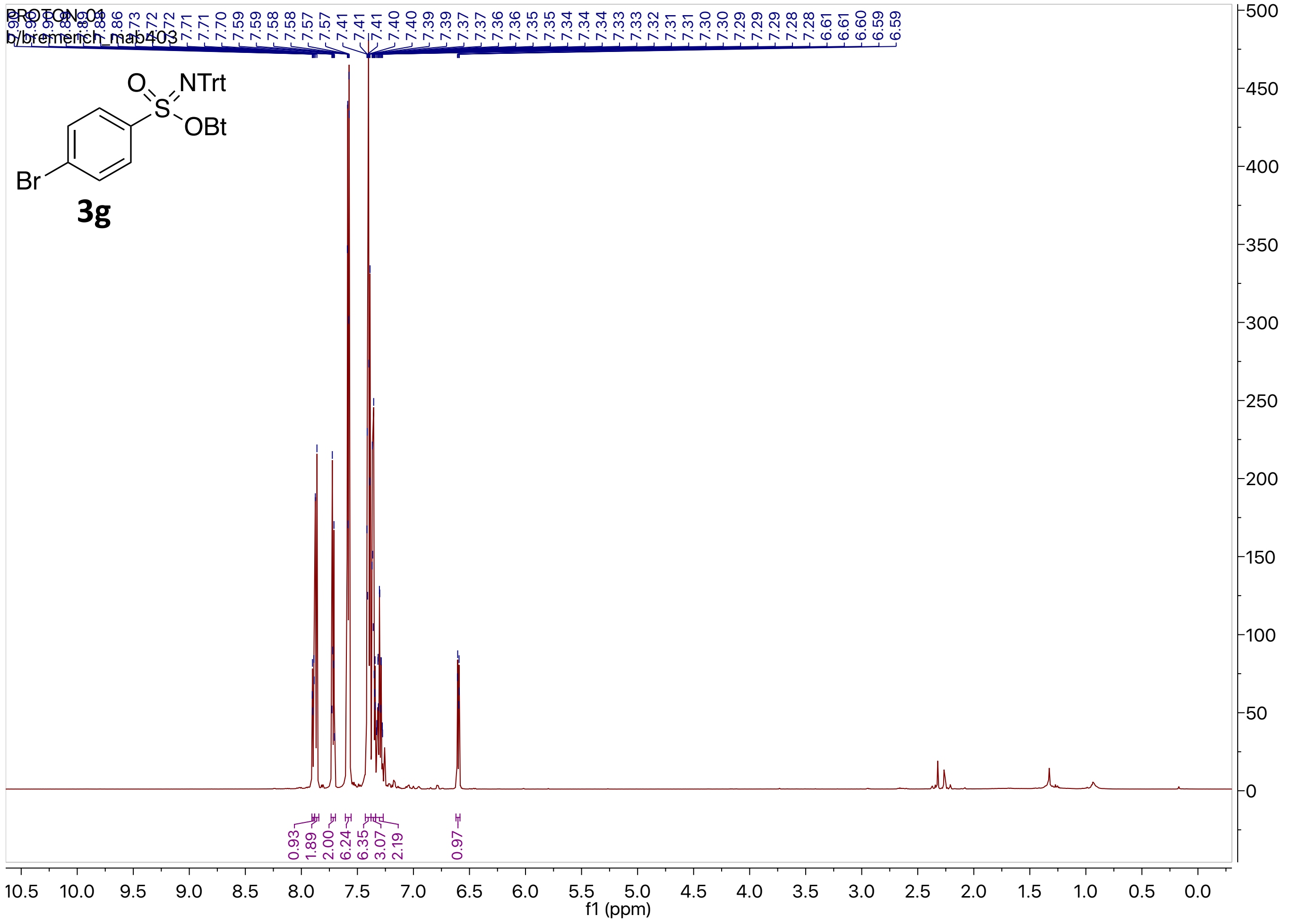
PROTON_01
b/bremerich_mab403
3g
NTrt
OBt
Br
f1 (ppm)

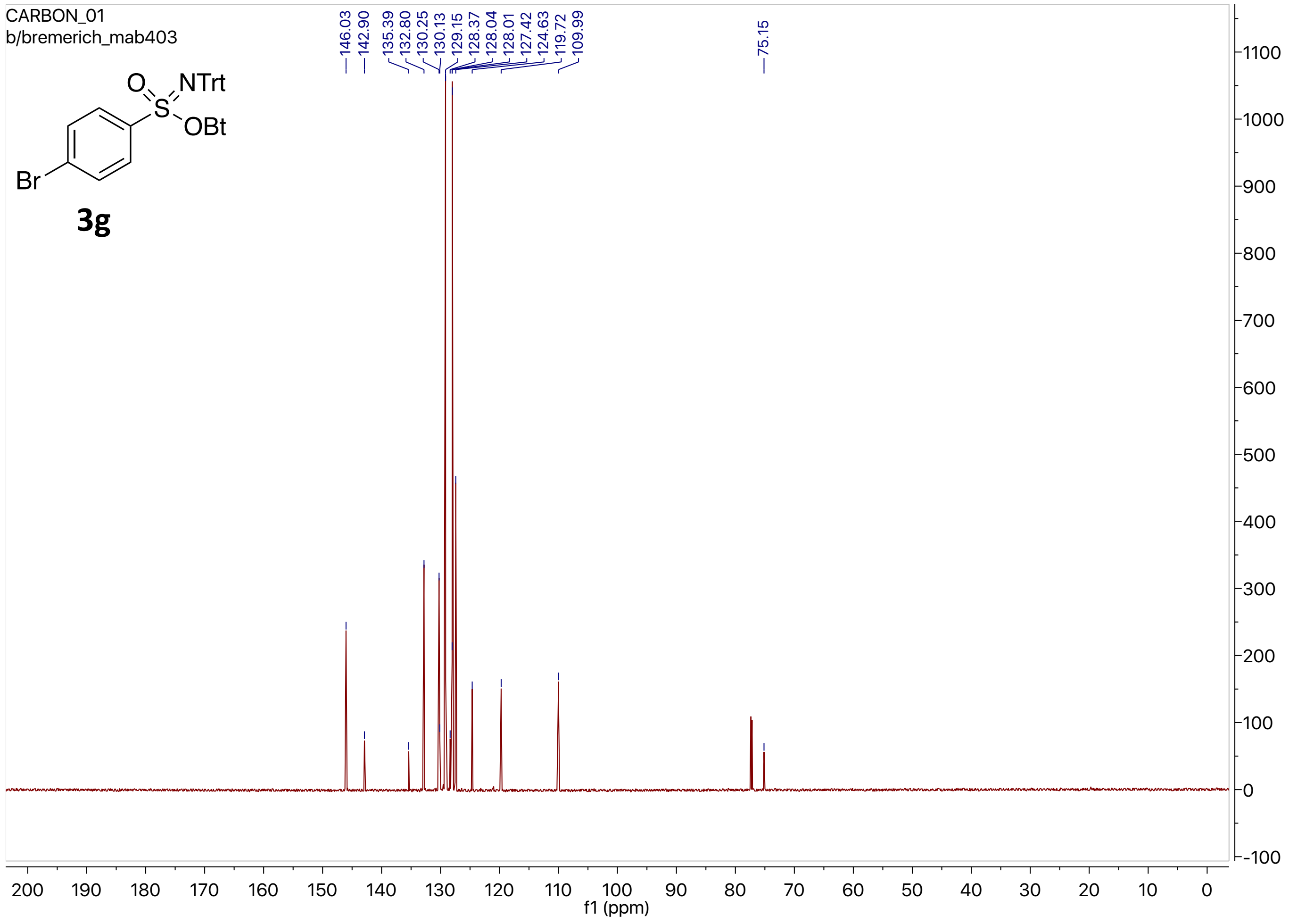
CARBON_01
b/bremerich_mab403
146.03
142.90
135.39
132.80
130.25
130.13
129.15
128.37
128.04
128.01
127.42
124.63
119.72
109.99
75.15
3g
NTrt
OBt
Br
f1 (ppm)

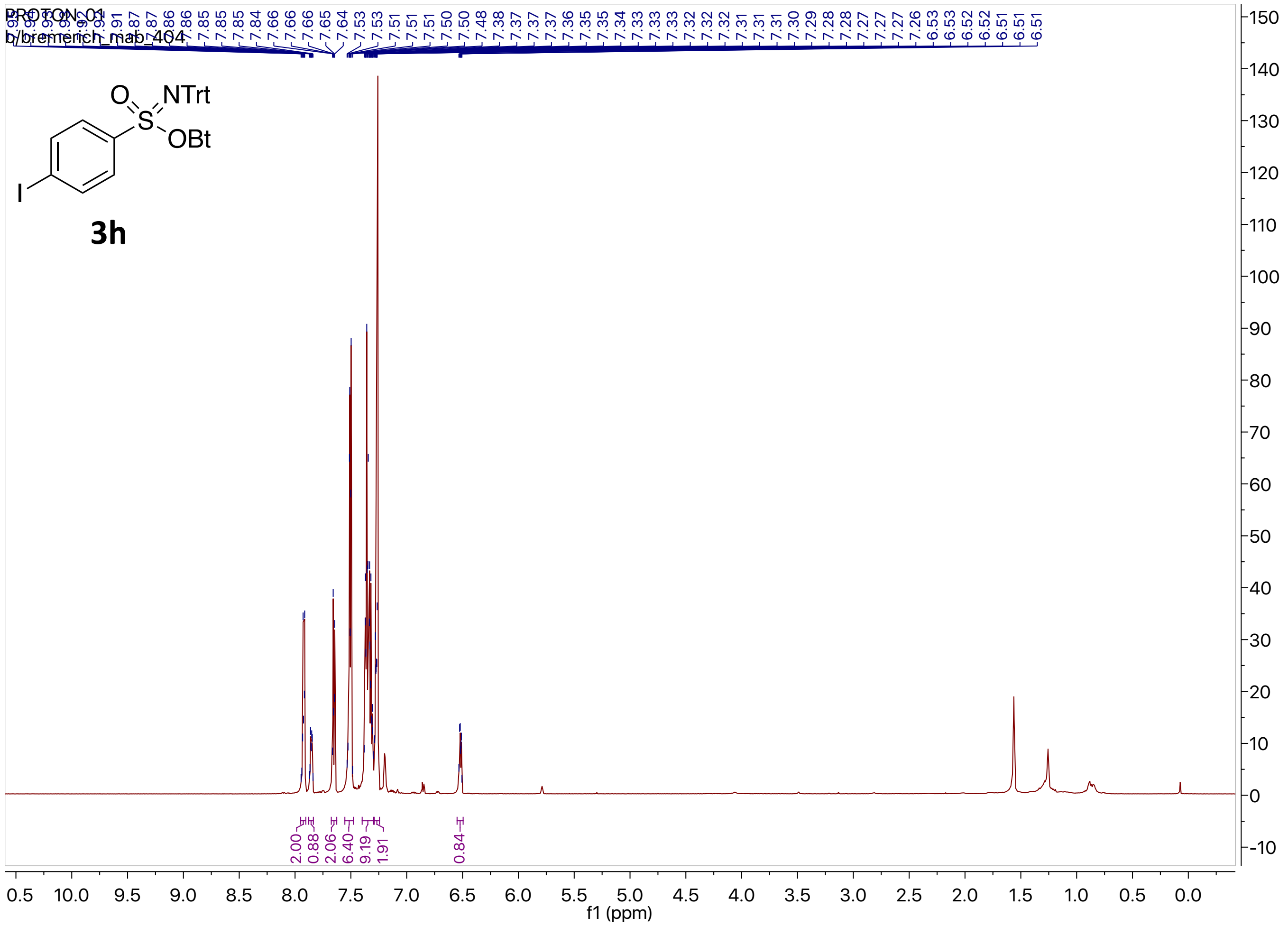

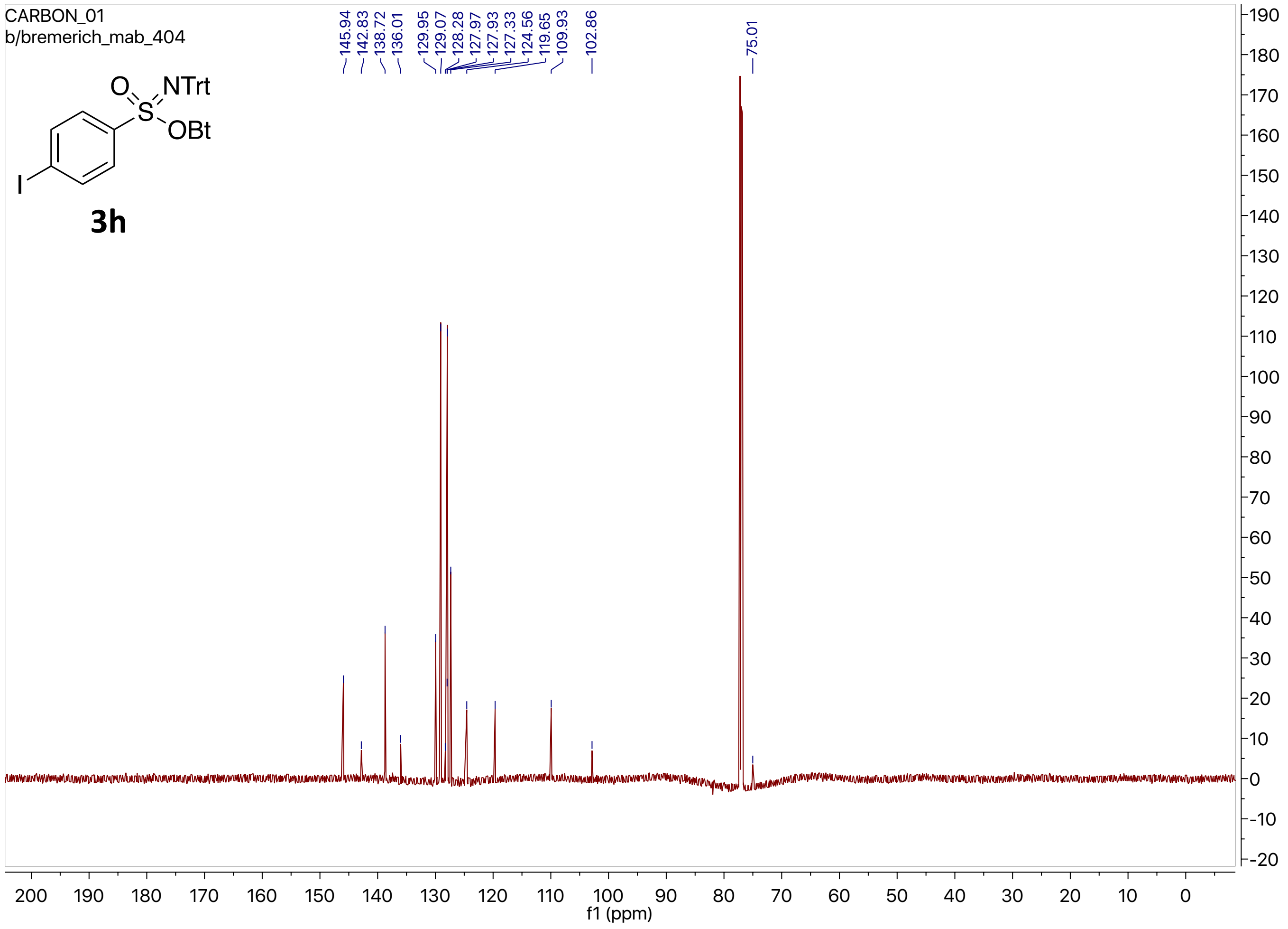

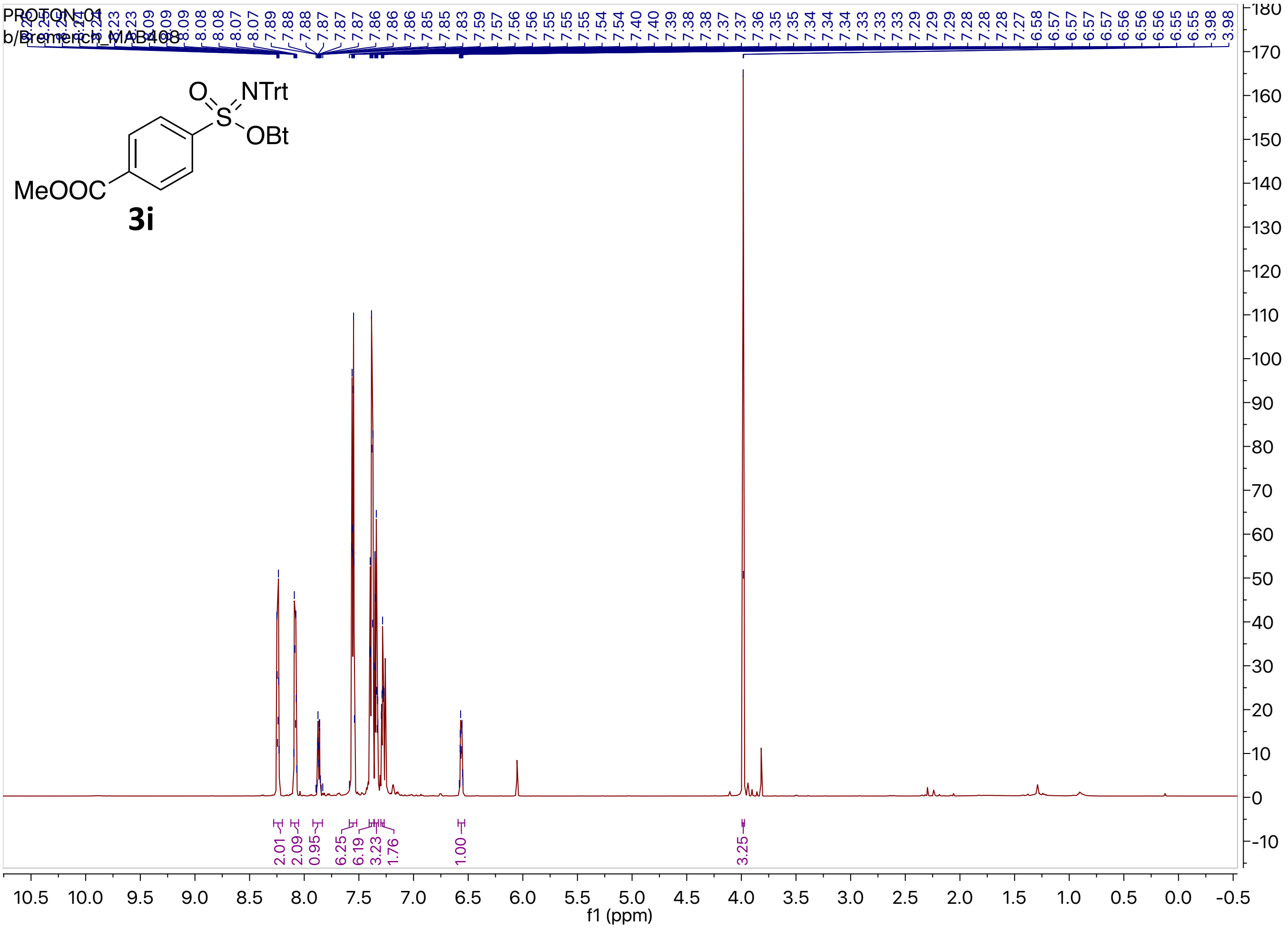
PROTON_01
b/Bremerich_MAB408
8.23
8.09
8.09
8.08
8.07
7.89
7.88
7.88
7.87
7.87
7.87
7.86
7.86
7.85
7.85
7.83
7.59
7.57
7.56
7.56
7.55
7.55
7.55
7.54
7.54
7.40
7.39
7.38
7.38
7.37
7.37
7.36
7.35
7.34
7.34
7.33
7.33
7.33
7.29
7.29
7.29
7.28
7.28
7.28
7.27
6.58
6.57
6.57
6.57
6.56
6.56
6.56
6.55
6.55
3.98
3.98
NTrt
OBt
MeOOC
3i
2.01
2.09
0.95
6.25
6.19
3.23
1.76
1.00
3.25
f1 (ppm)

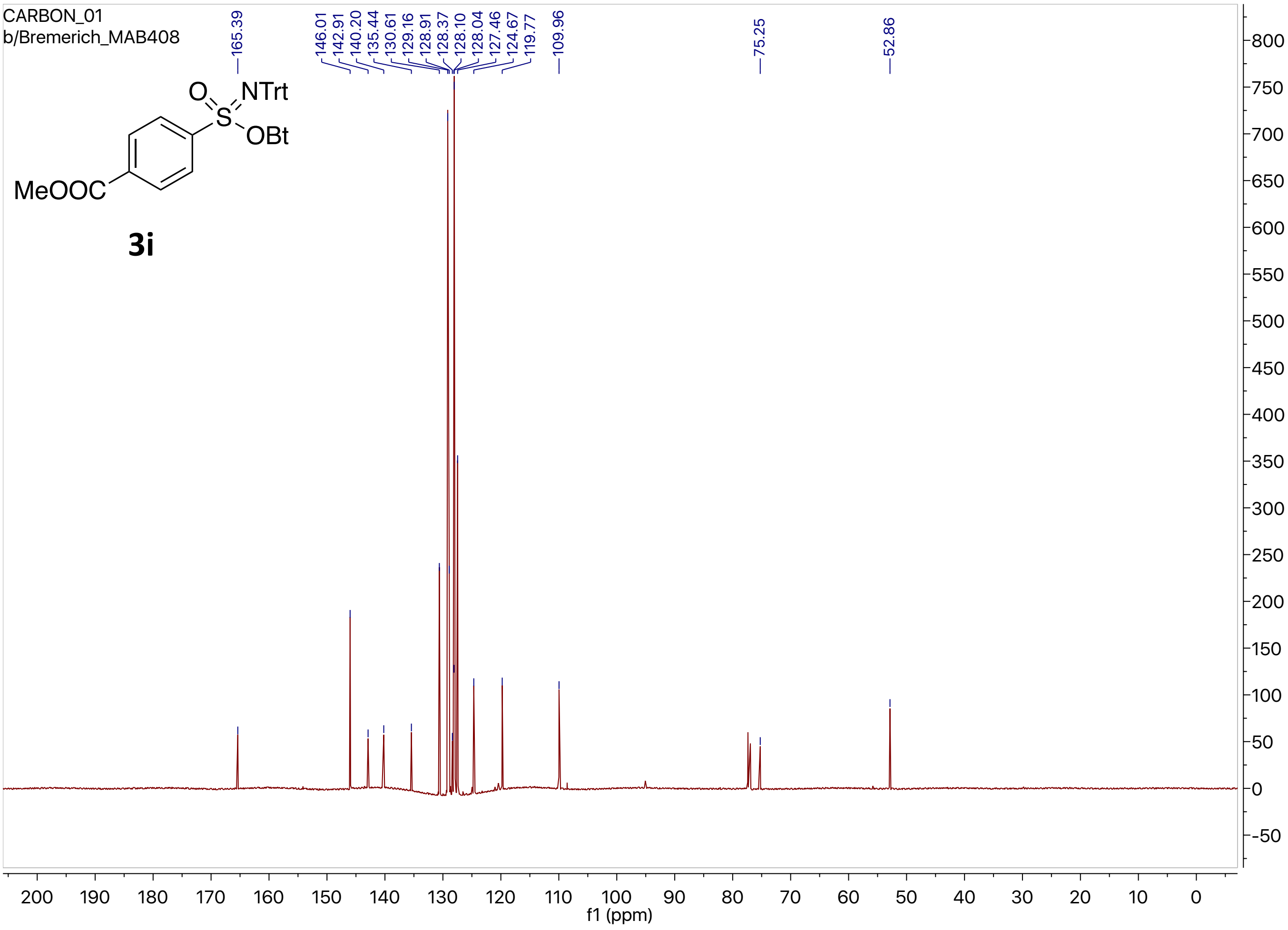
CARBON_01
b/Bremerich_MAB408
165.39
146.01
142.91
140.20
135.44
130.61
129.16
128.91
128.37
128.10
128.04
127.46
124.67
119.77
109.96
75.25
52.86
NTrt
OBt
MeOOC
3i
f1 (ppm)

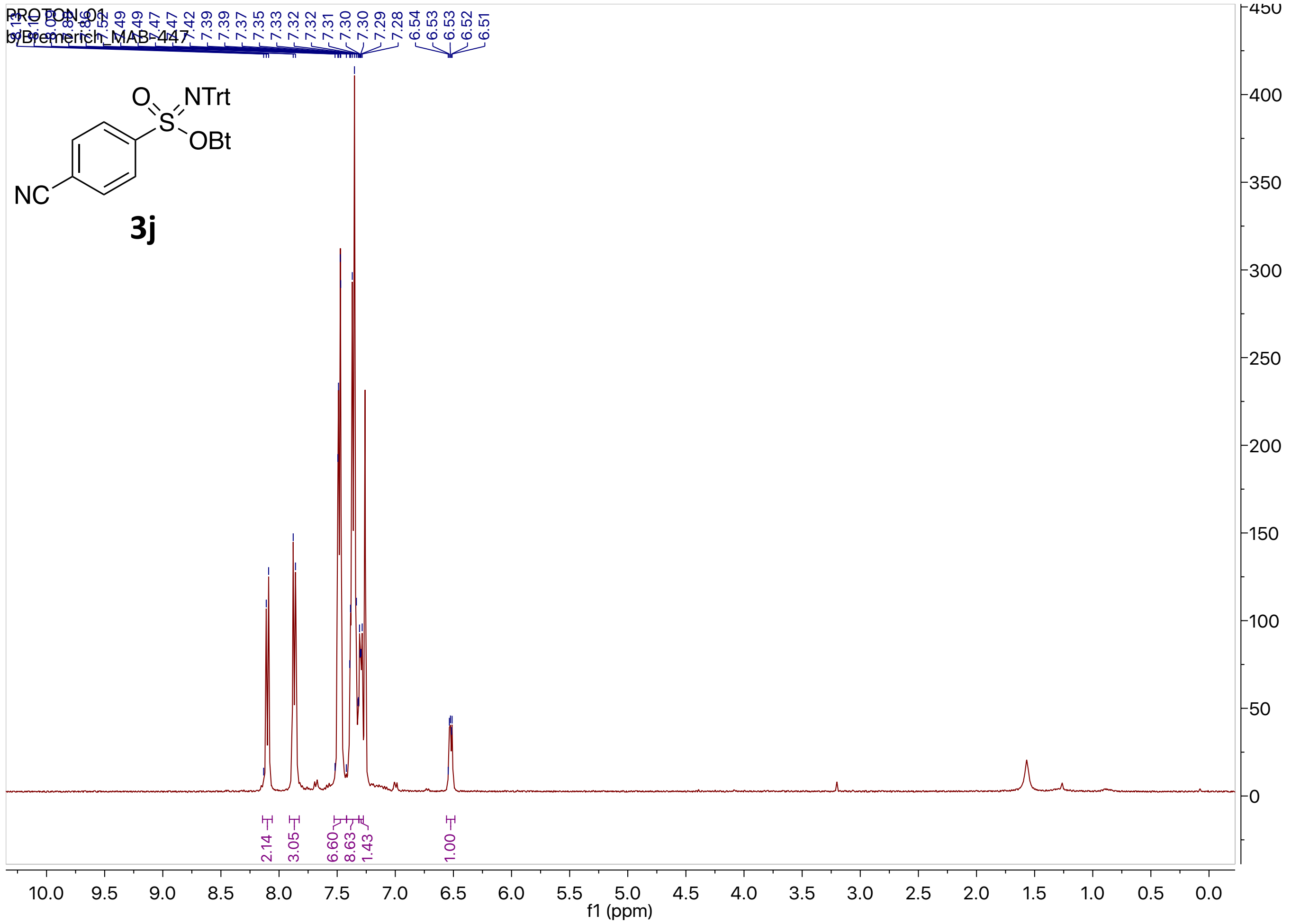
PROTON_01
b/Bremerich_MAB-447
7.49
7.49
7.47
7.47
7.42
7.39
7.39
7.37
7.35
7.33
7.32
7.32
7.31
7.30
7.30
7.29
7.28
6.54
6.53
6.53
6.52
6.51
O
NTrt
S
OBt
NC
3j
2.14
3.05
6.60
8.63
1.43
1.00
f1 (ppm)

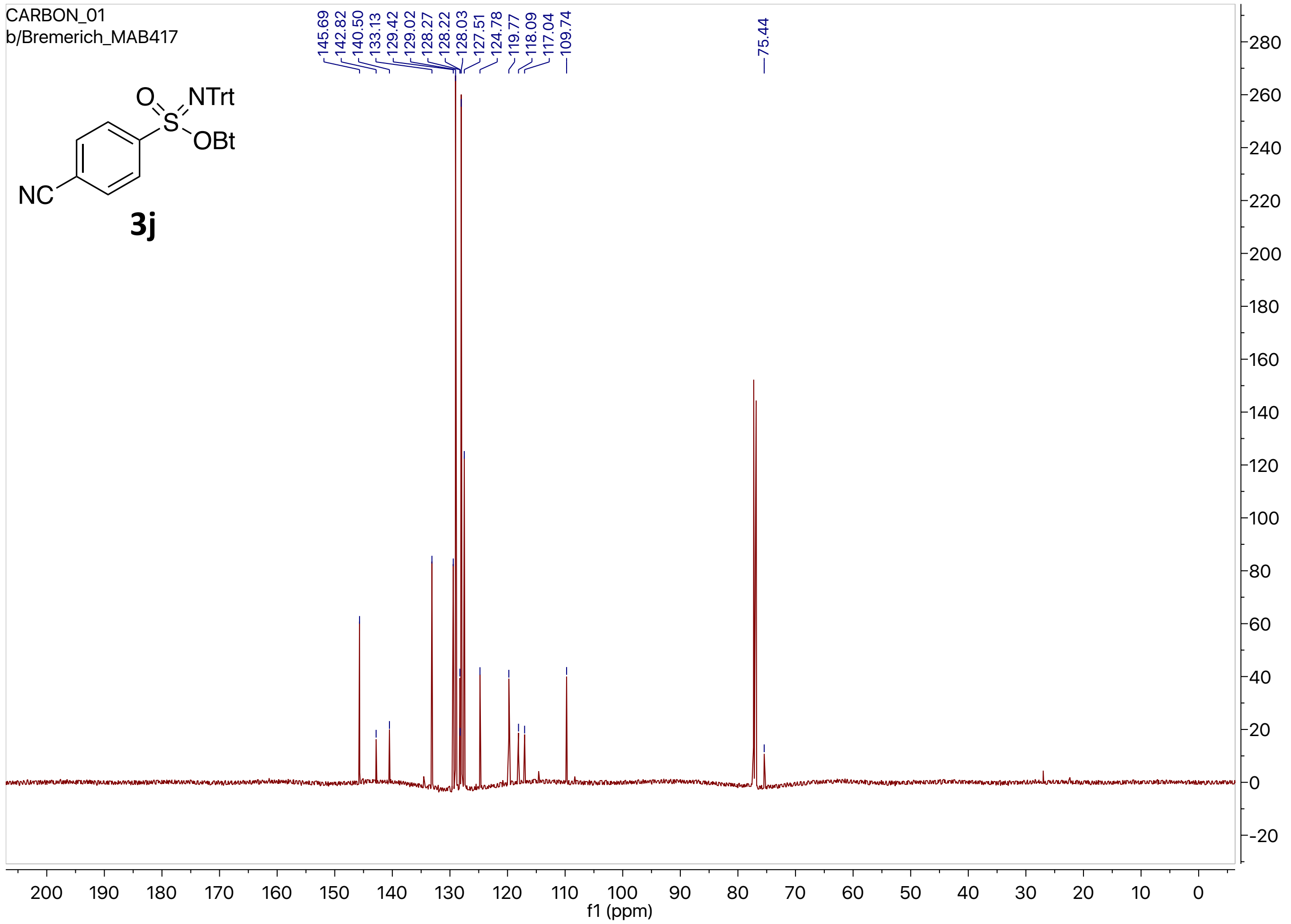
CARBON_01
b/Bremerich_MAB417
145.69
142.82
140.50
133.13
129.42
129.02
128.27
128.22
128.03
127.51
124.78
119.77
118.09
117.04
109.74
75.44
O
NTrt
S
OBt
NC
3j
f1 (ppm)

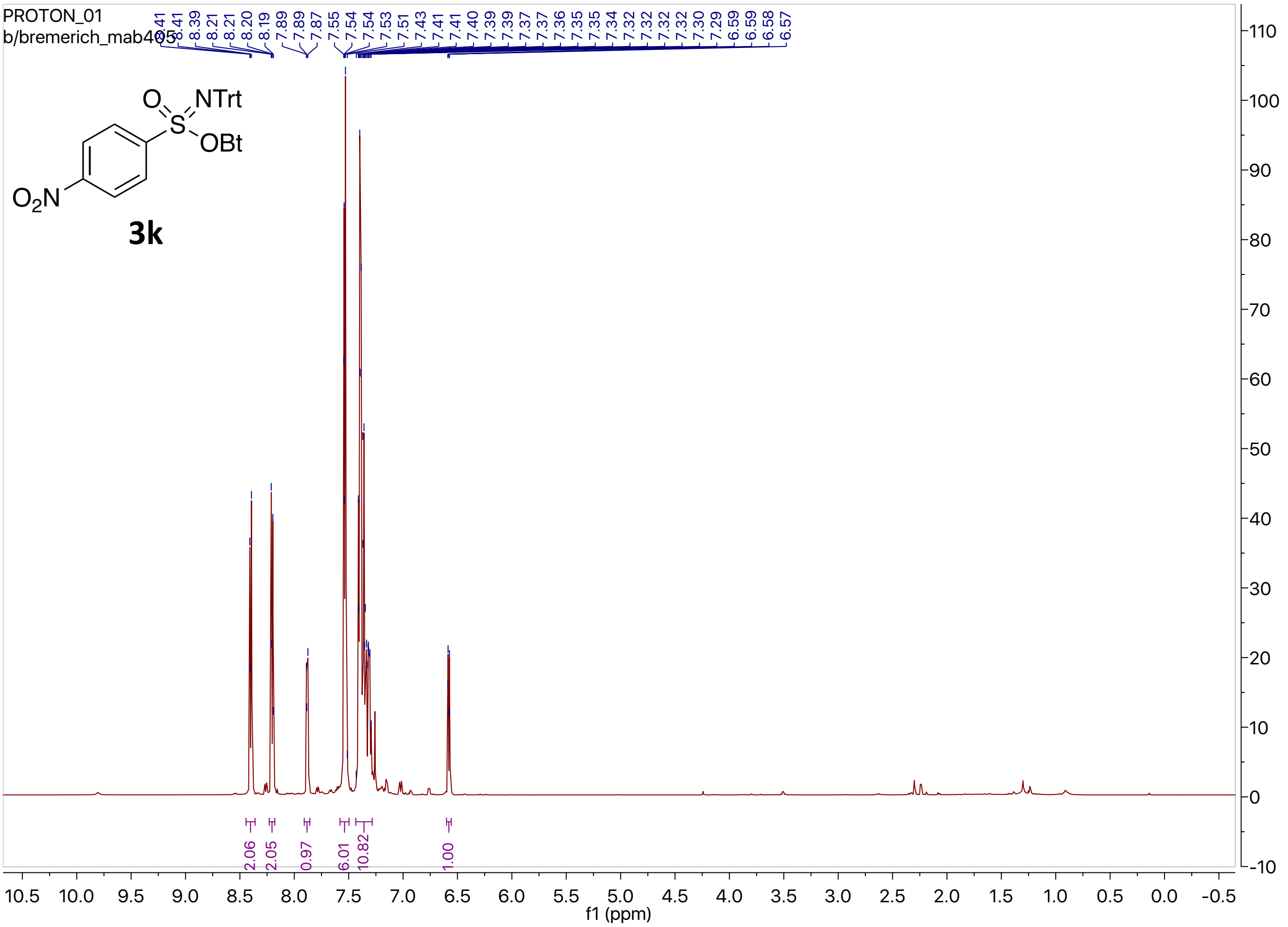
PROTON_01
b/bremerich_mab405
3k
f1 (ppm)

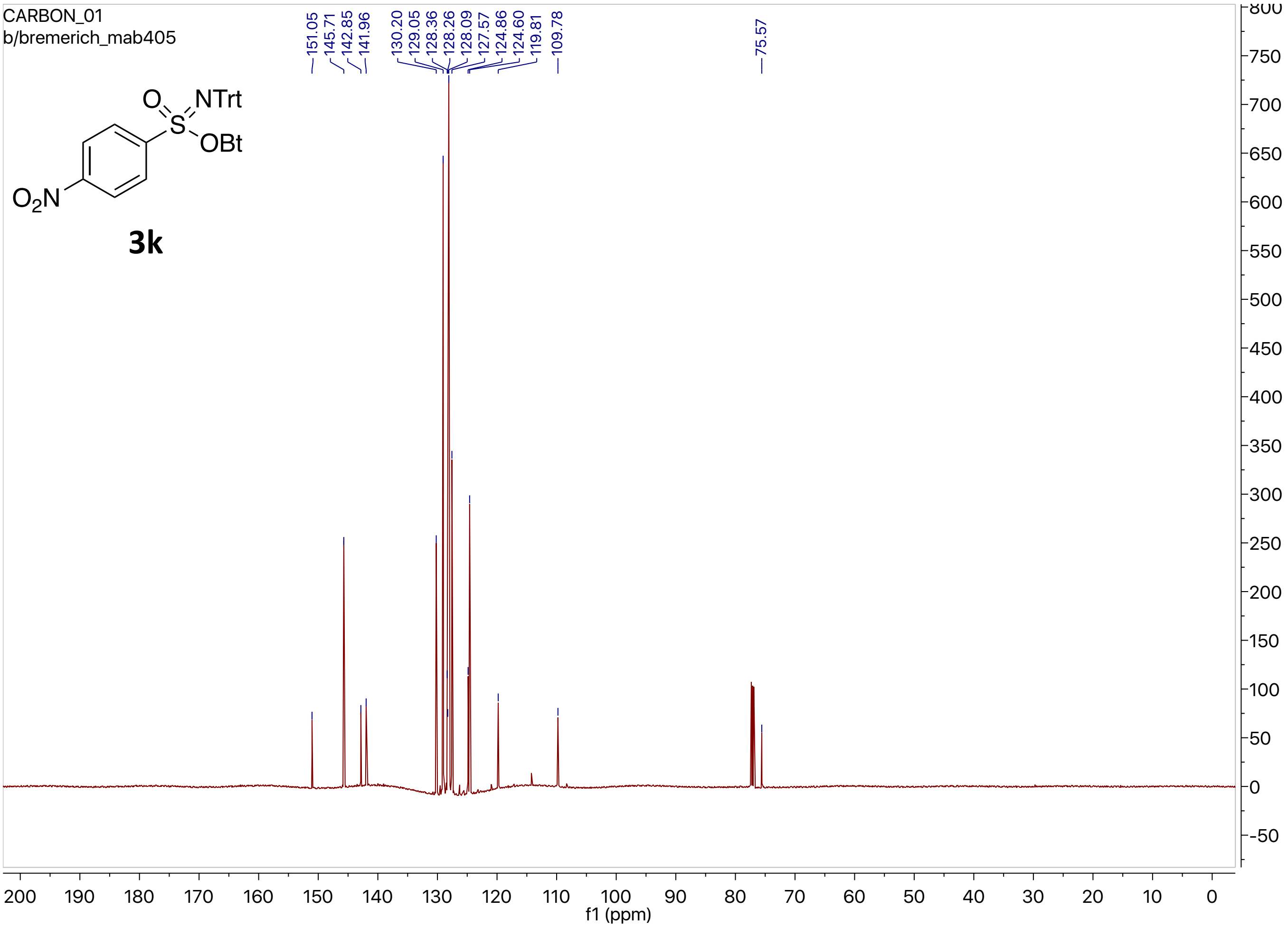
CARBON_01
b/bremerich_mab405
3k
f1 (ppm)

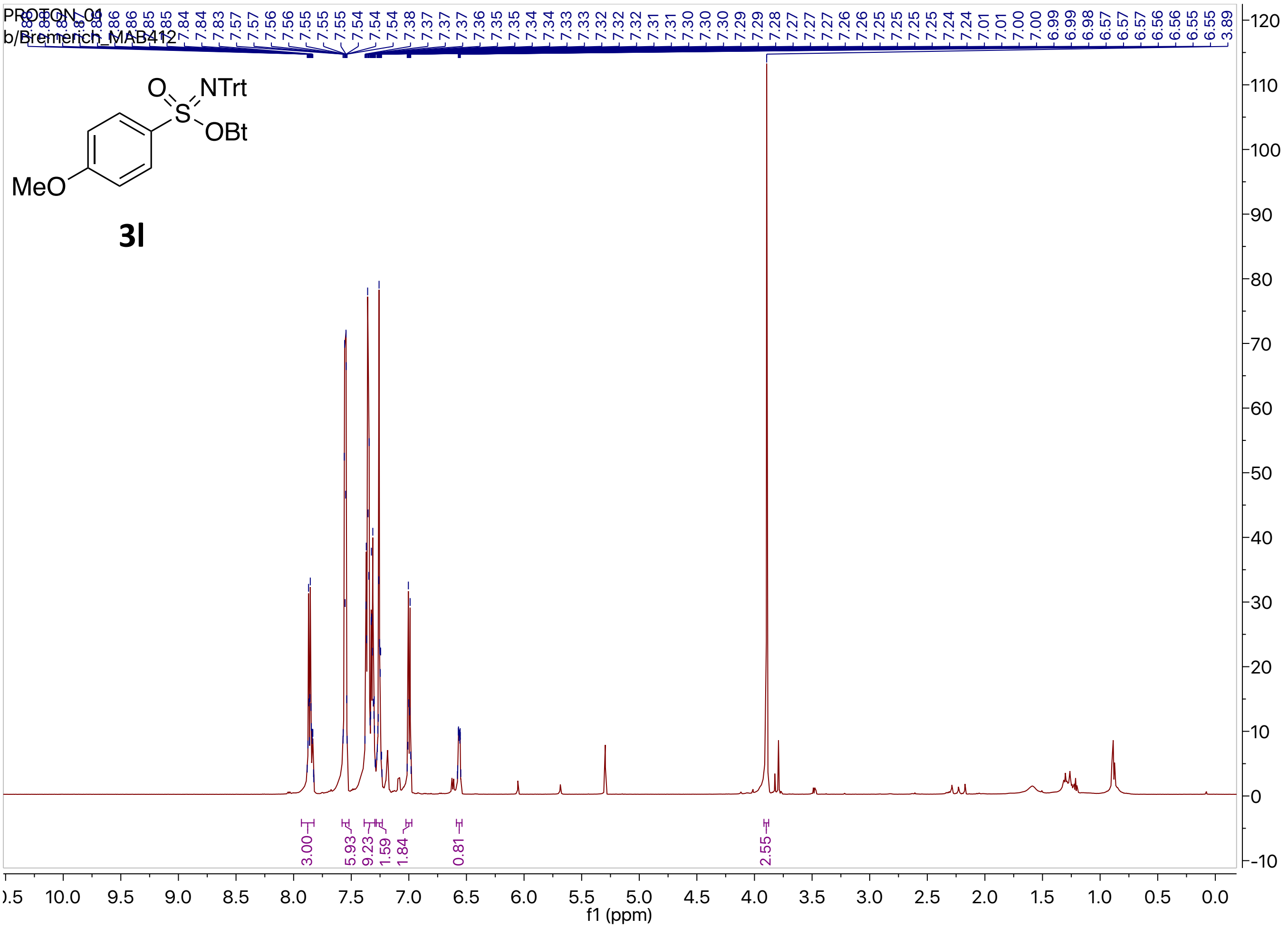
PROTON_01
b/Bremerich_MAB412
7.86 7.86 7.85 7.85 7.84 7.84 7.83 7.57 7.57 7.56 7.56 7.55 7.55 7.55 7.54 7.54 7.54 7.54 7.38 7.37 7.37 7.37 7.36 7.35 7.35 7.34 7.34 7.33 7.33 7.32 7.32 7.32 7.31 7.31 7.30 7.30 7.30 7.29 7.29 7.28 7.27 7.27 7.27 7.26 7.25 7.25 7.25 7.25 7.24 7.24 7.01 7.01 7.00 7.00 6.99 6.99 6.98 6.57 6.57 6.56 6.56 6.55 6.55 3.89
NTrt
OBt
MeO
3.00 5.93 9.23 1.59 1.84 0.81 2.55
f1 (ppm)

3l

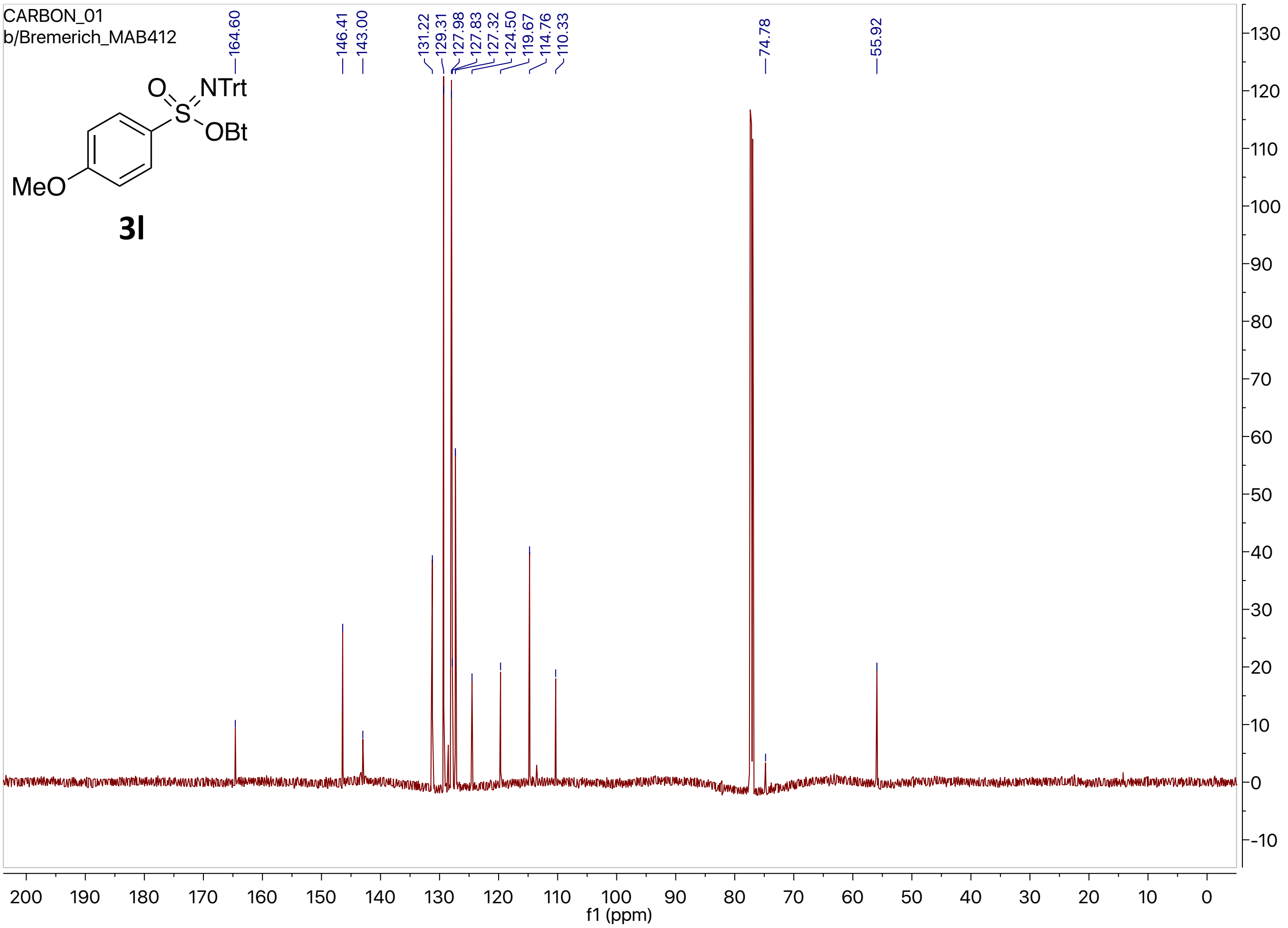
CARBON_01
b/Bremerich_MAB412
164.60
146.41
143.00
131.22
129.31
127.98
127.83
127.32
124.50
119.67
114.76
110.33
74.78
55.92
NTrt
OBt
MeO
f1 (ppm)

3l

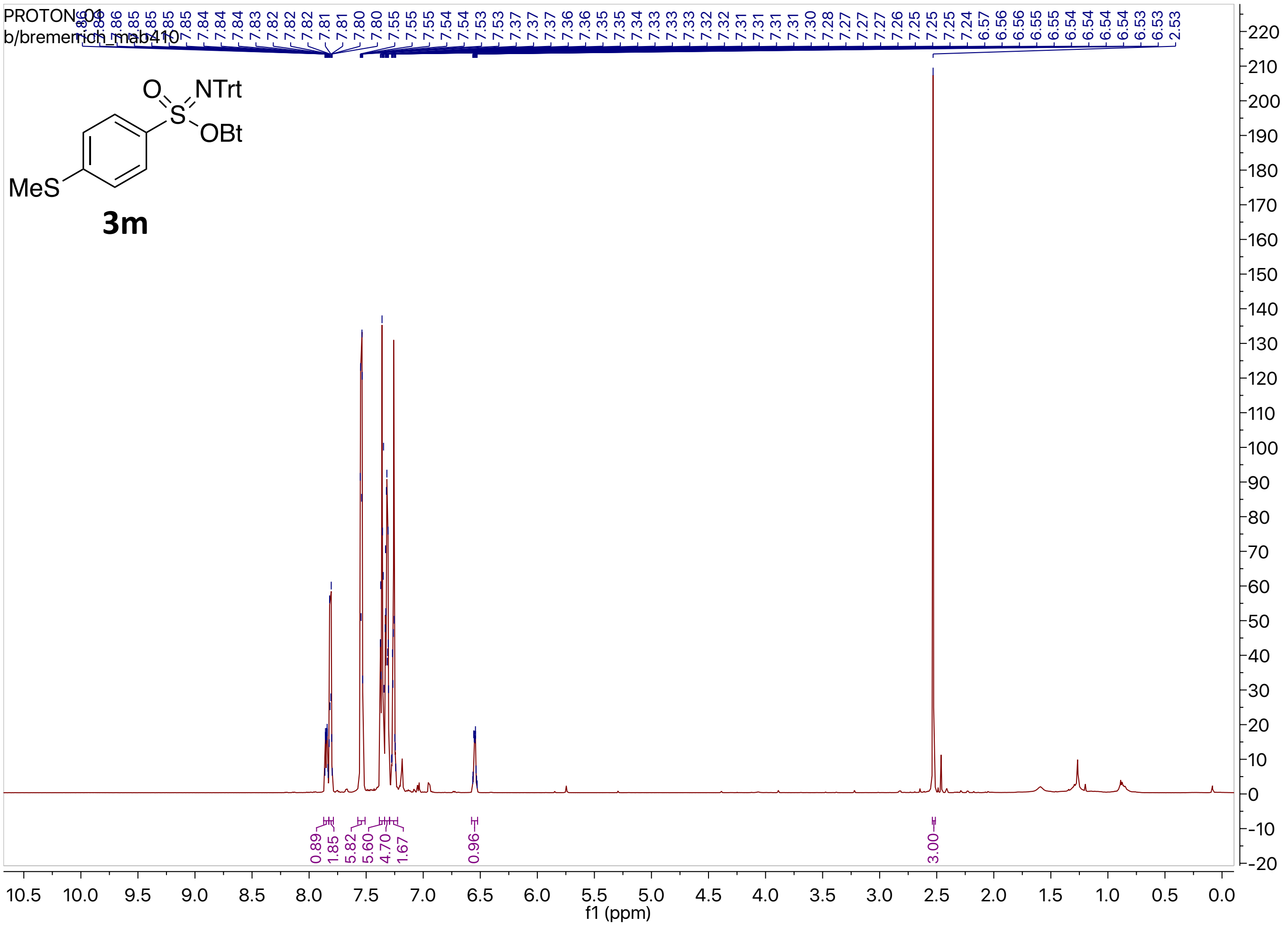

PROTON_01
b/bremerrich_mab410
NTrt
OBt
MeS
3m
f1 (ppm)

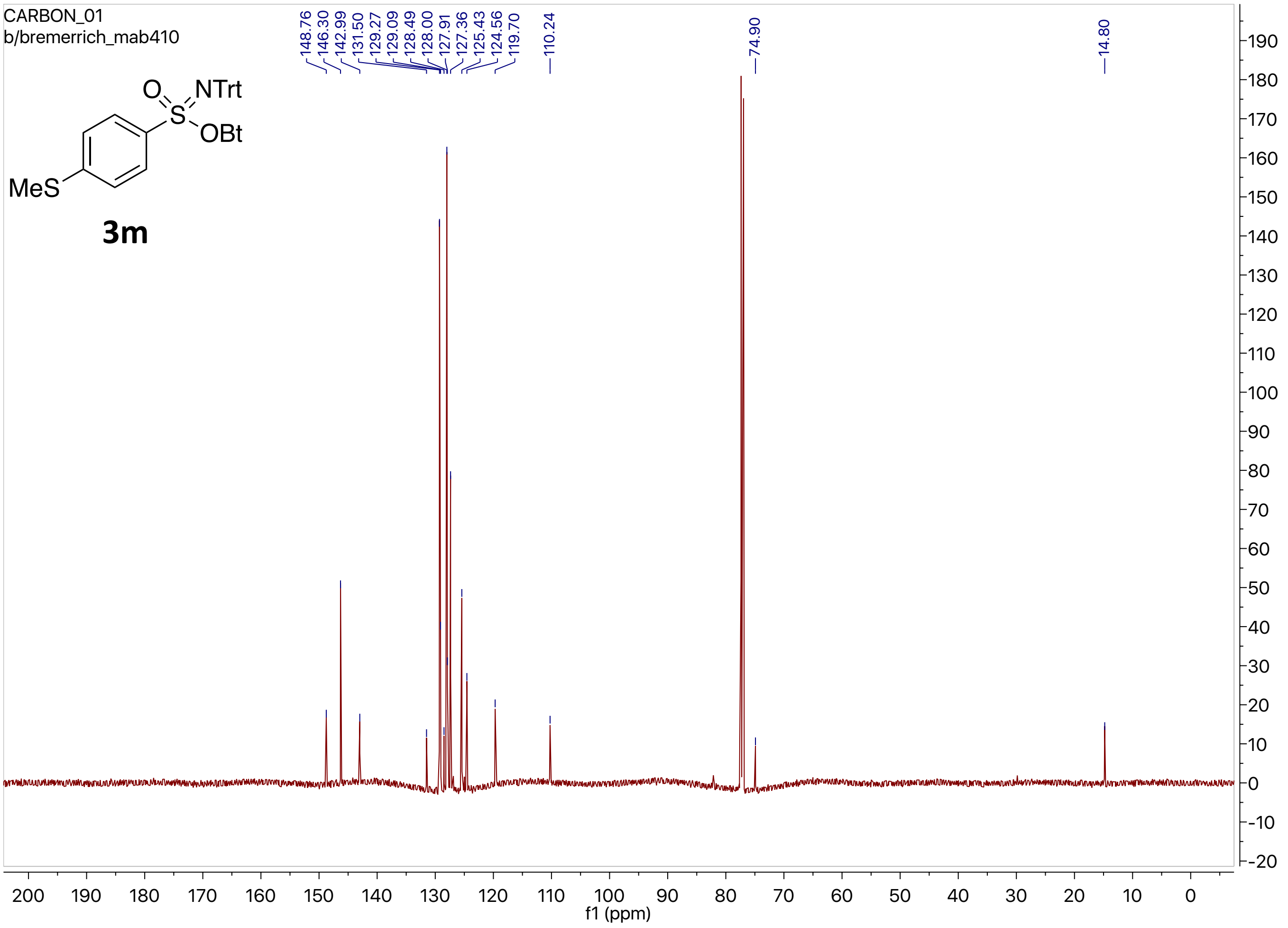

CARBON_01
b/bremerrich_mab410
NTrt
OBt
MeS
3m
148.76
146.30
142.99
131.50
129.27
129.09
128.49
128.00
127.91
127.36
125.43
124.56
119.70
110.24
74.90
14.80
f1 (ppm)

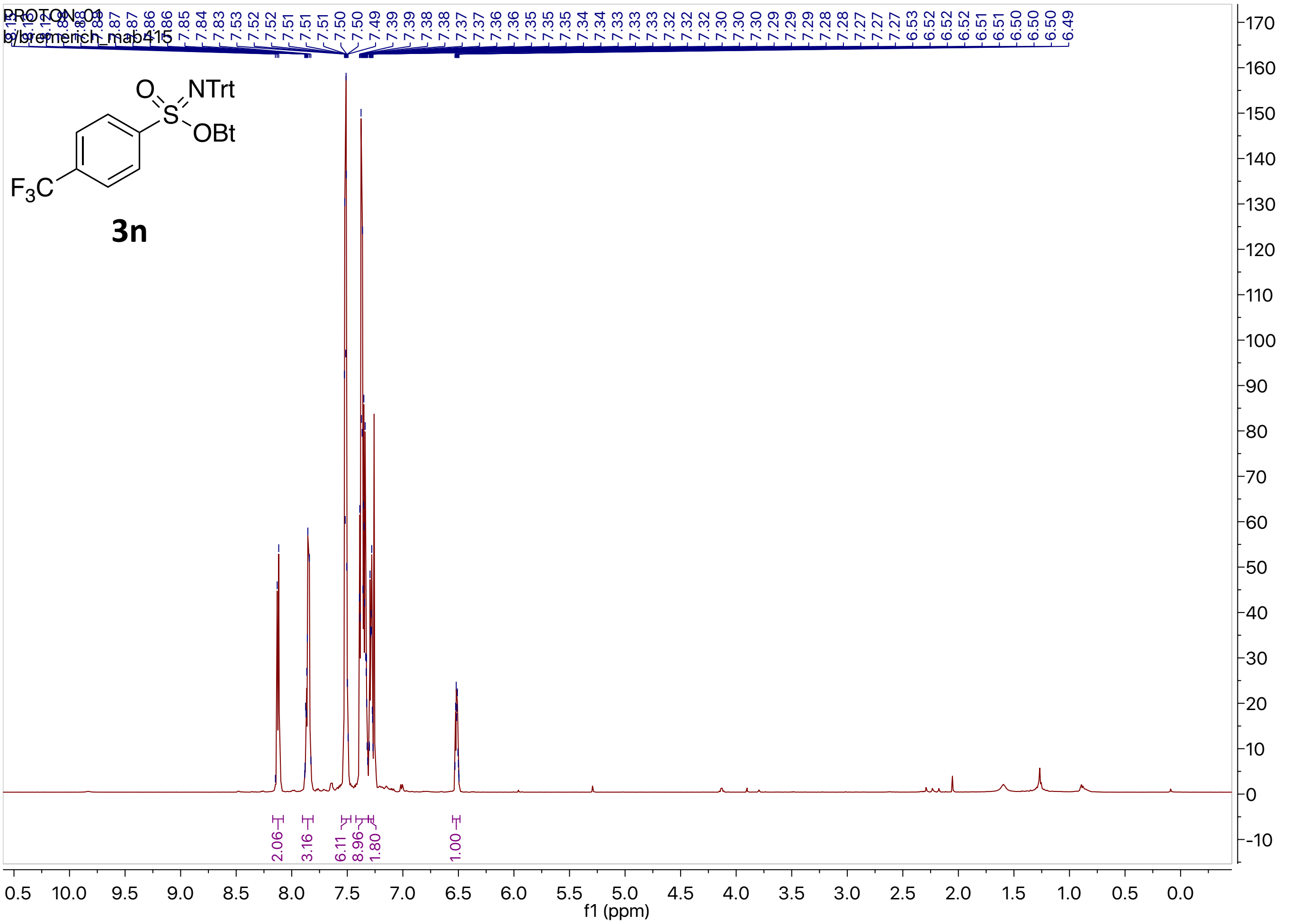
PROTON_01
b/bremerich_mab415
7.87 7.87 7.86 7.86 7.85 7.84 7.83 7.53 7.52 7.52 7.51 7.51 7.51 7.50 7.50 7.49 7.39 7.39 7.38 7.38 7.37 7.37 7.36 7.36 7.35 7.35 7.35 7.34 7.34 7.33 7.33 7.33 7.32 7.32 7.32 7.30 7.30 7.29 7.29 7.29 7.28 7.28 7.27 7.27 7.27 6.53 6.52 6.52 6.51 6.51 6.50 6.50 6.50 6.49
NTrt
OBt
F3C
3n
2.06 3.16 6.11 8.96 1.80 1.00
f1 (ppm)

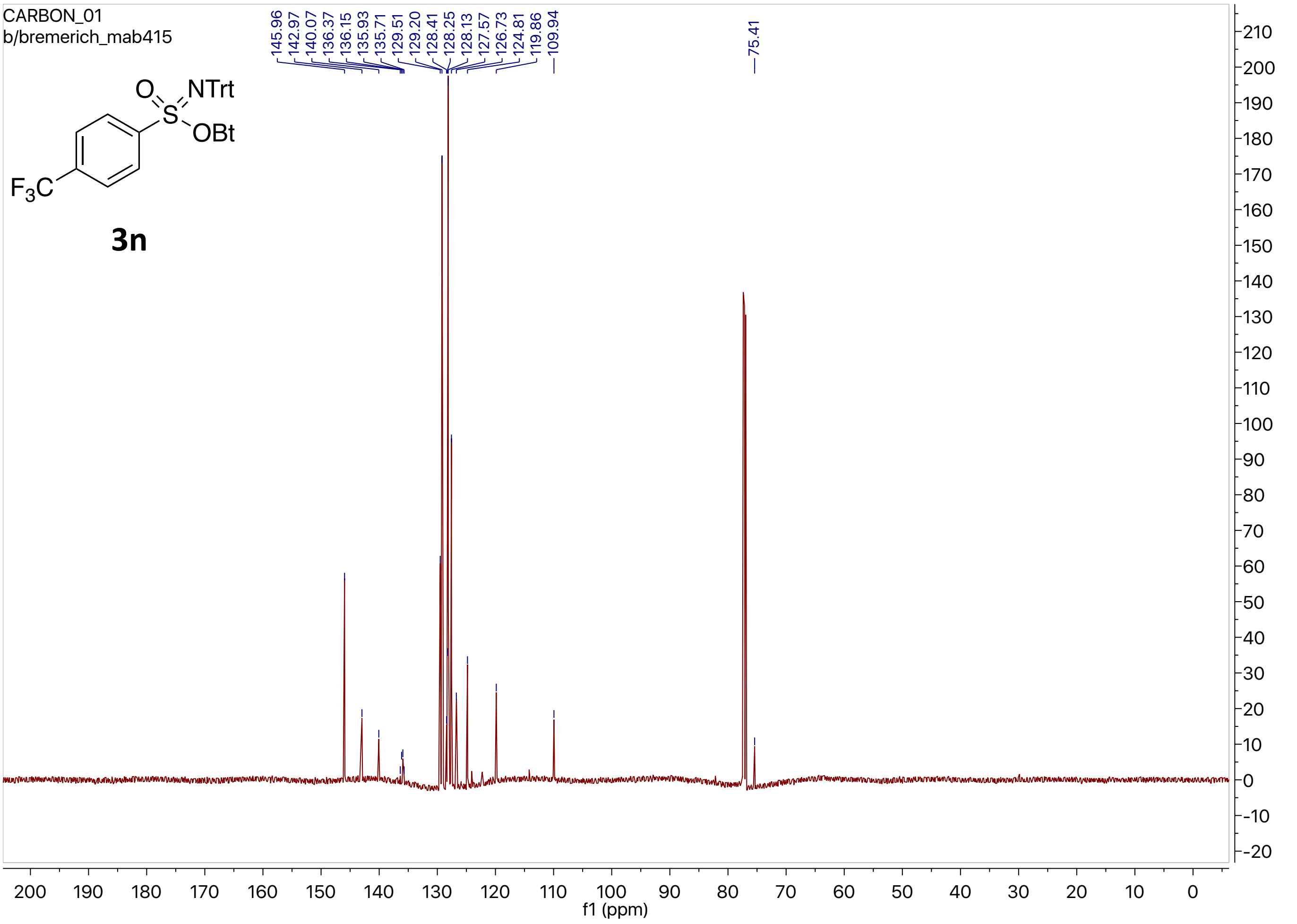
CARBON_01
b/bremerich_mab415
145.96 142.97 140.07 136.37 136.15 135.93 135.71 129.51 129.20 128.41 128.25 128.13 127.57 126.73 124.81 119.86 109.94
75.41
NTrt
OBt
F3C
3n
f1 (ppm)

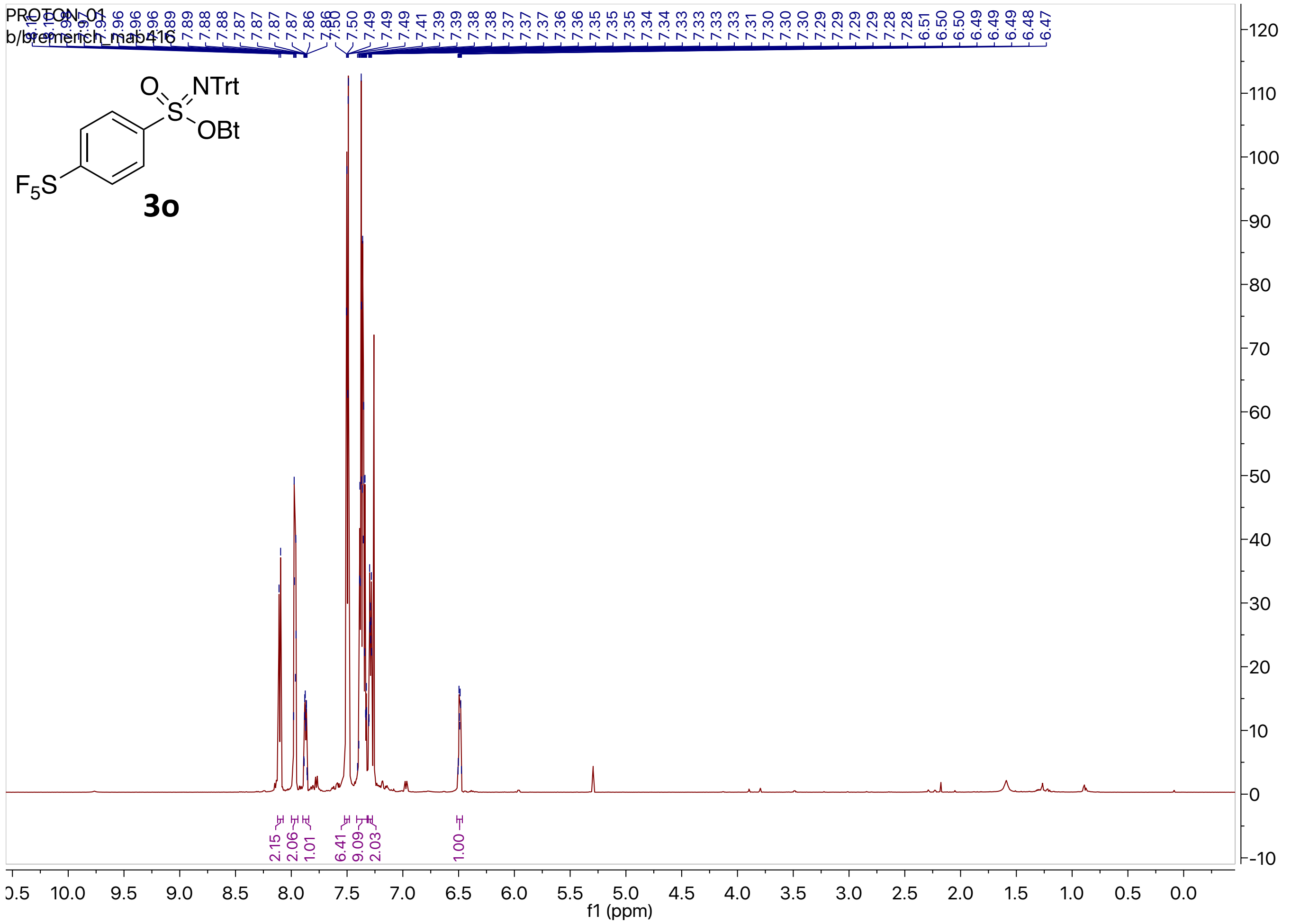
PROTON_01
b/bremerich_mab416
7.96 7.96 7.96 7.89 7.88 7.88 7.87 7.87 7.87 7.86 7.86 7.50 7.50 7.49 7.49 7.49 7.41 7.39 7.39 7.38 7.38 7.37 7.37 7.36 7.35 7.35 7.35 7.34 7.33 7.33 7.33 7.33 7.31 7.30 7.30 7.29 7.29 7.29 7.28 7.28 6.51 6.50 6.49 6.49 6.49 6.48 6.47
O
NTrt
S
OBt
F5S
3o
2.15
2.06
1.01
6.41
9.09
2.03
1.00
f1 (ppm)

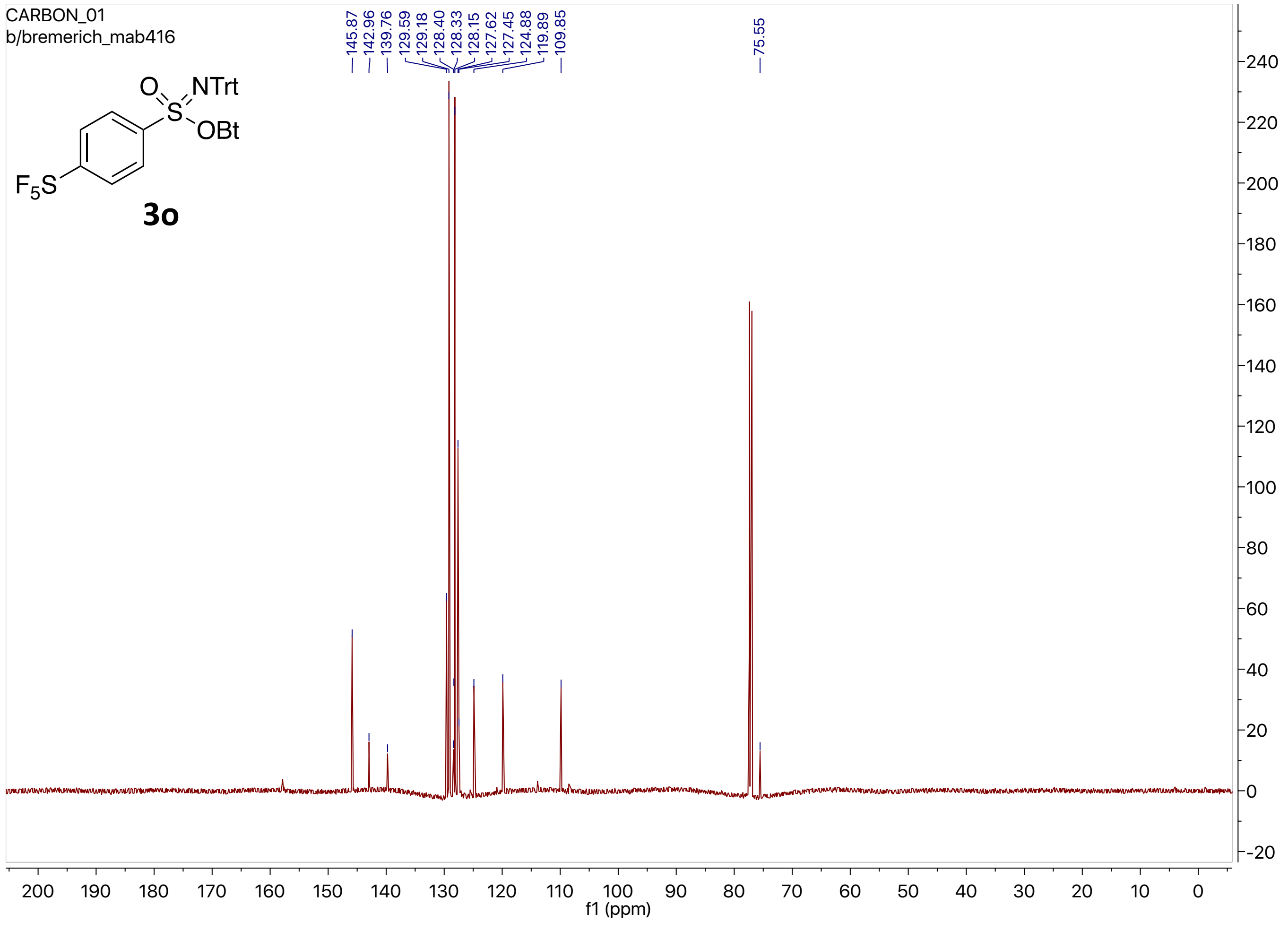
CARBON_01
b/bremerich_mab416
145.87 142.96 139.76 129.59 129.18 128.40 128.33 128.15 127.62 127.45 124.88 119.89 109.85
75.55
O
NTrt
S
OBt
F5S
3o
f1 (ppm)

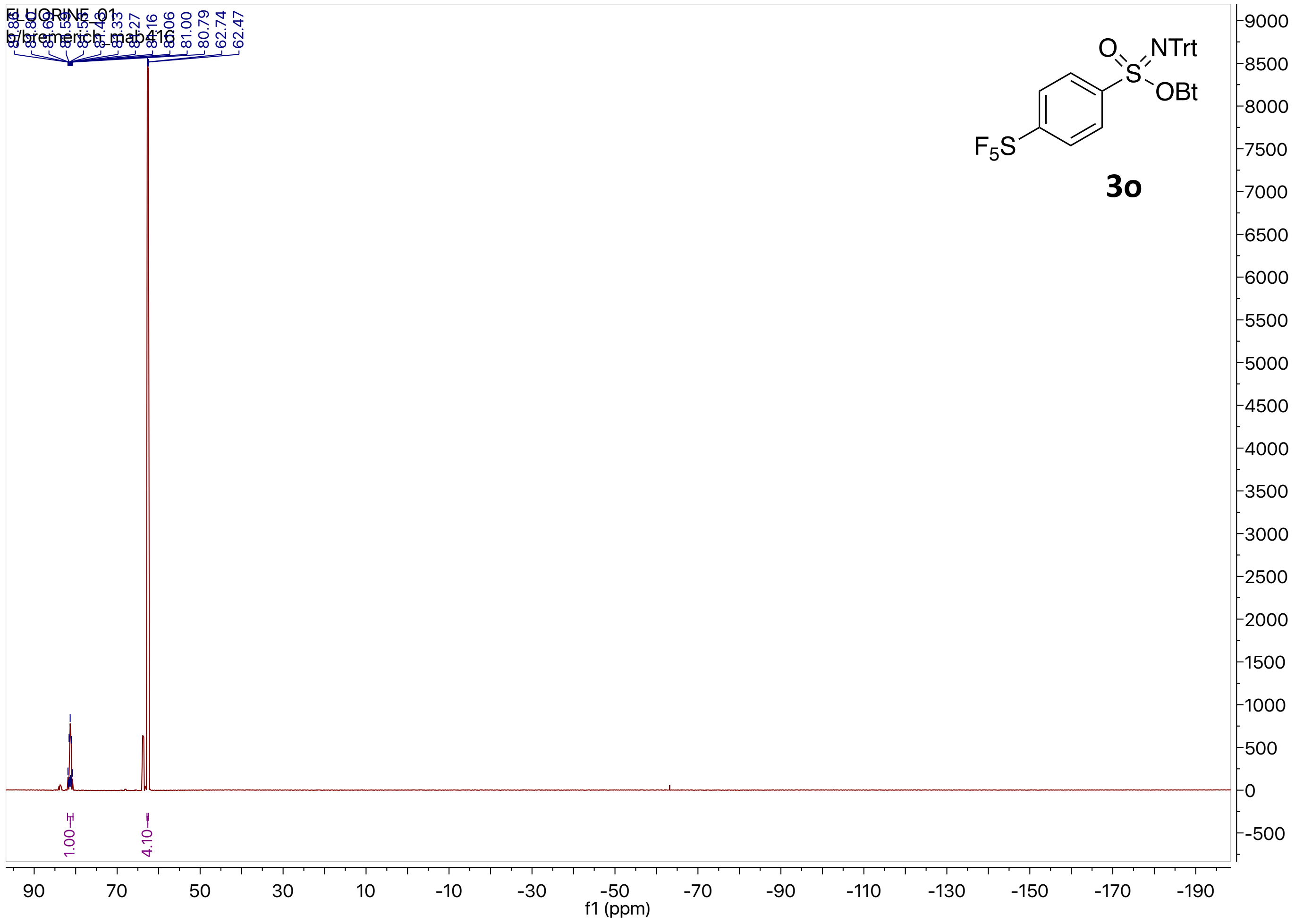
FLUORINE_01
b/bremerich: mab416
81.00
80.79
62.74
62.47
1.00
4.10
f1 (ppm)
NTrt
OBt
F5S
3o

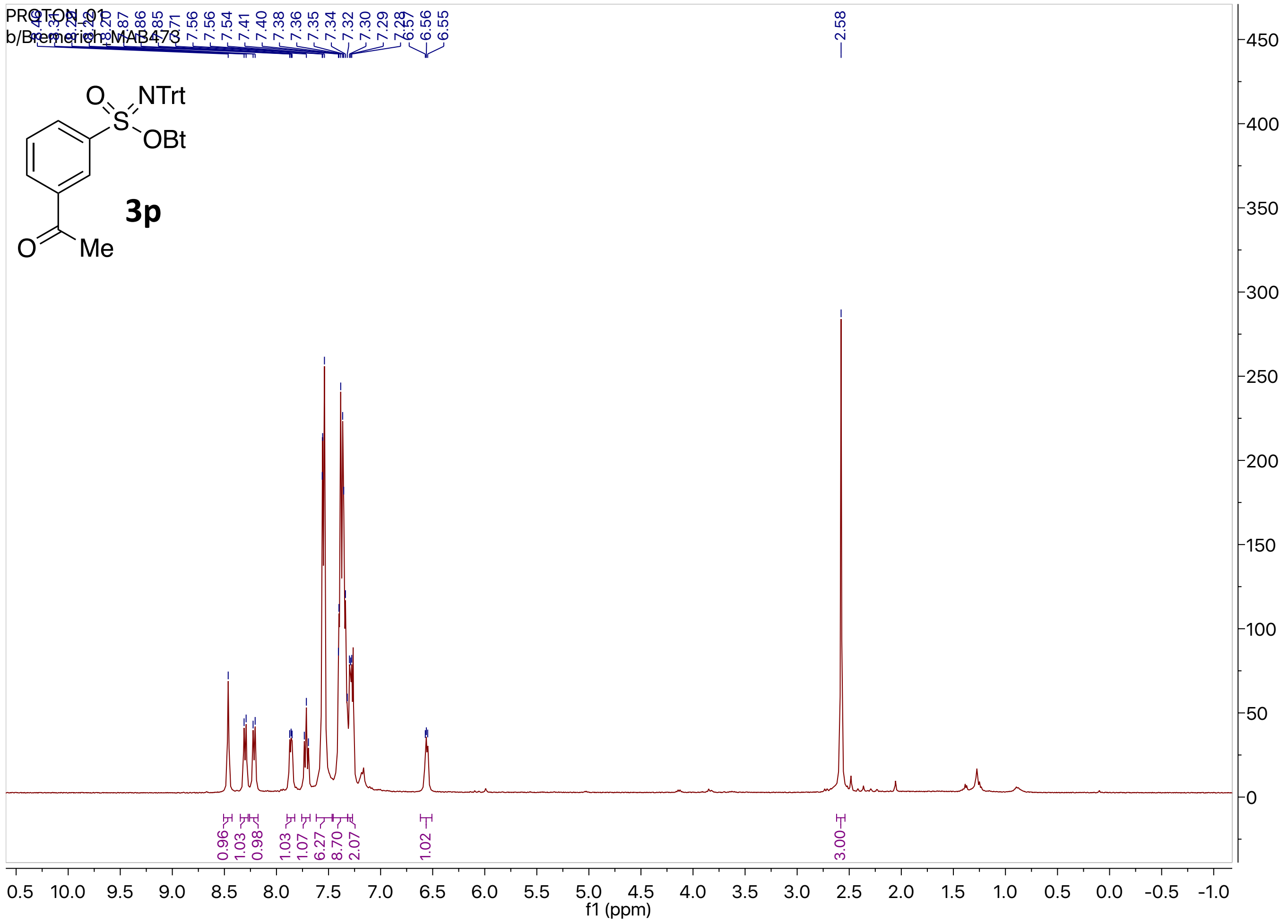
PROTON_01
b/Bremerich_MAB473
7.87
7.86
7.85
7.71
7.56
7.56
7.54
7.41
7.40
7.38
7.36
7.35
7.34
7.32
7.30
7.29
7.28
6.57
6.56
6.55
2.58
NTrt
OBt
O
S
O
Me
3p
0.96
1.03
0.98
1.03
1.07
6.27
8.70
2.07
1.02
3.00
f1 (ppm)

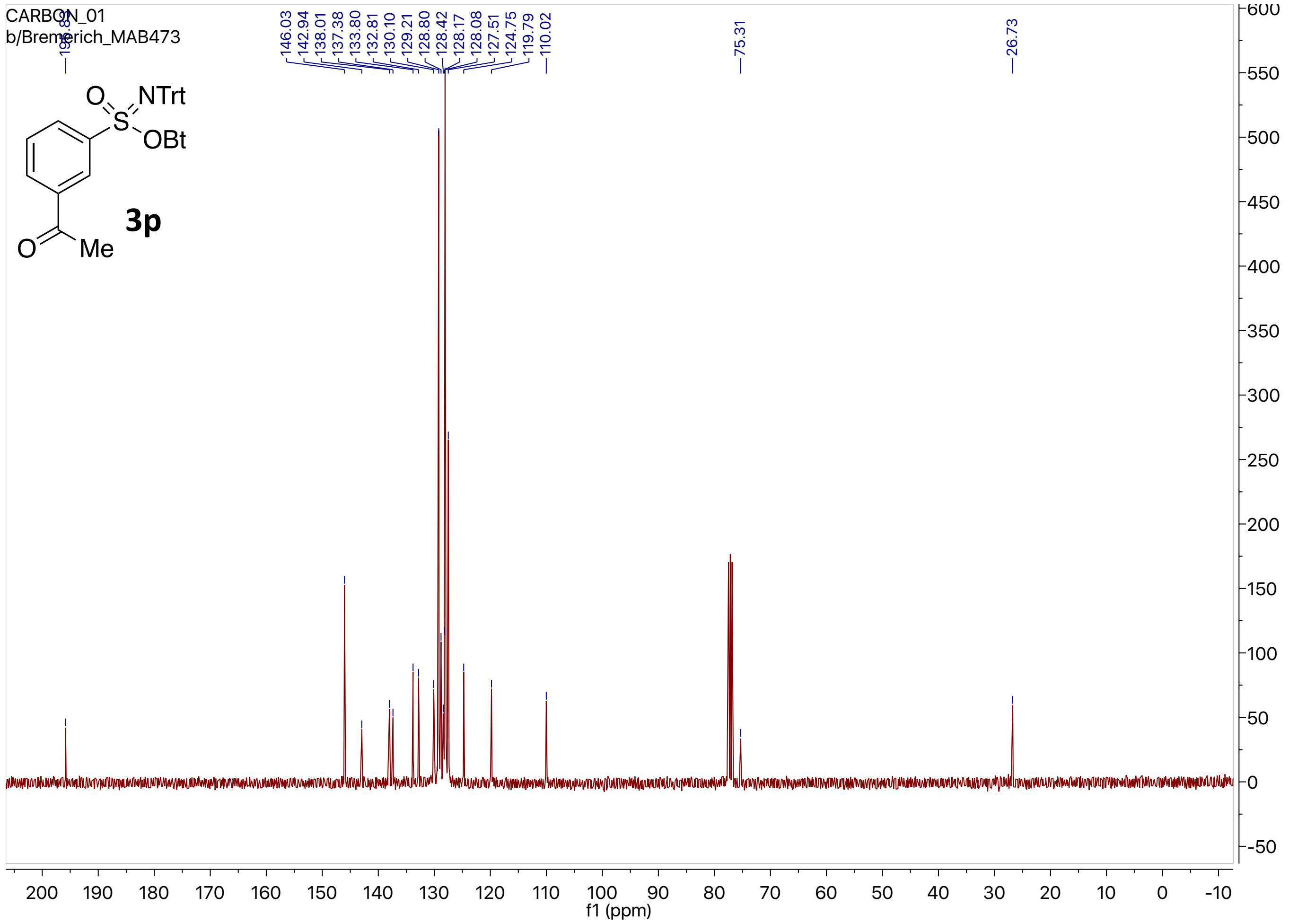
CARBON_01
b/Bremerich_MAB473
146.03
142.94
138.01
137.38
133.80
132.81
130.10
129.21
128.80
128.42
128.17
128.08
127.51
124.75
119.79
110.02
75.31
26.73
NTrt
OBt
O
S
O
Me
3p
f1 (ppm)

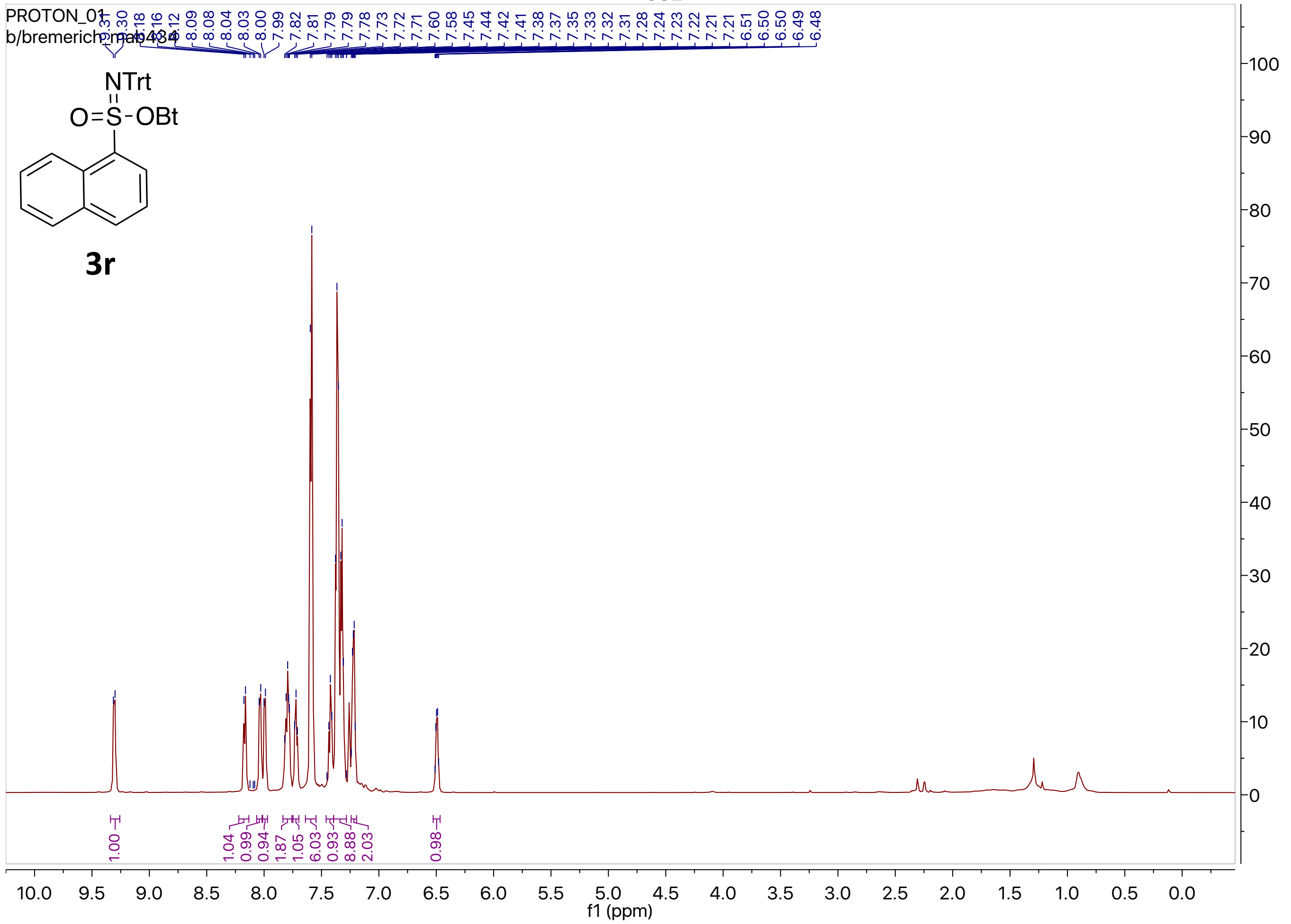
PROTON_01
b/bremerich_mab434
NTrt
O=S-OBt
f1 (ppm)

3r

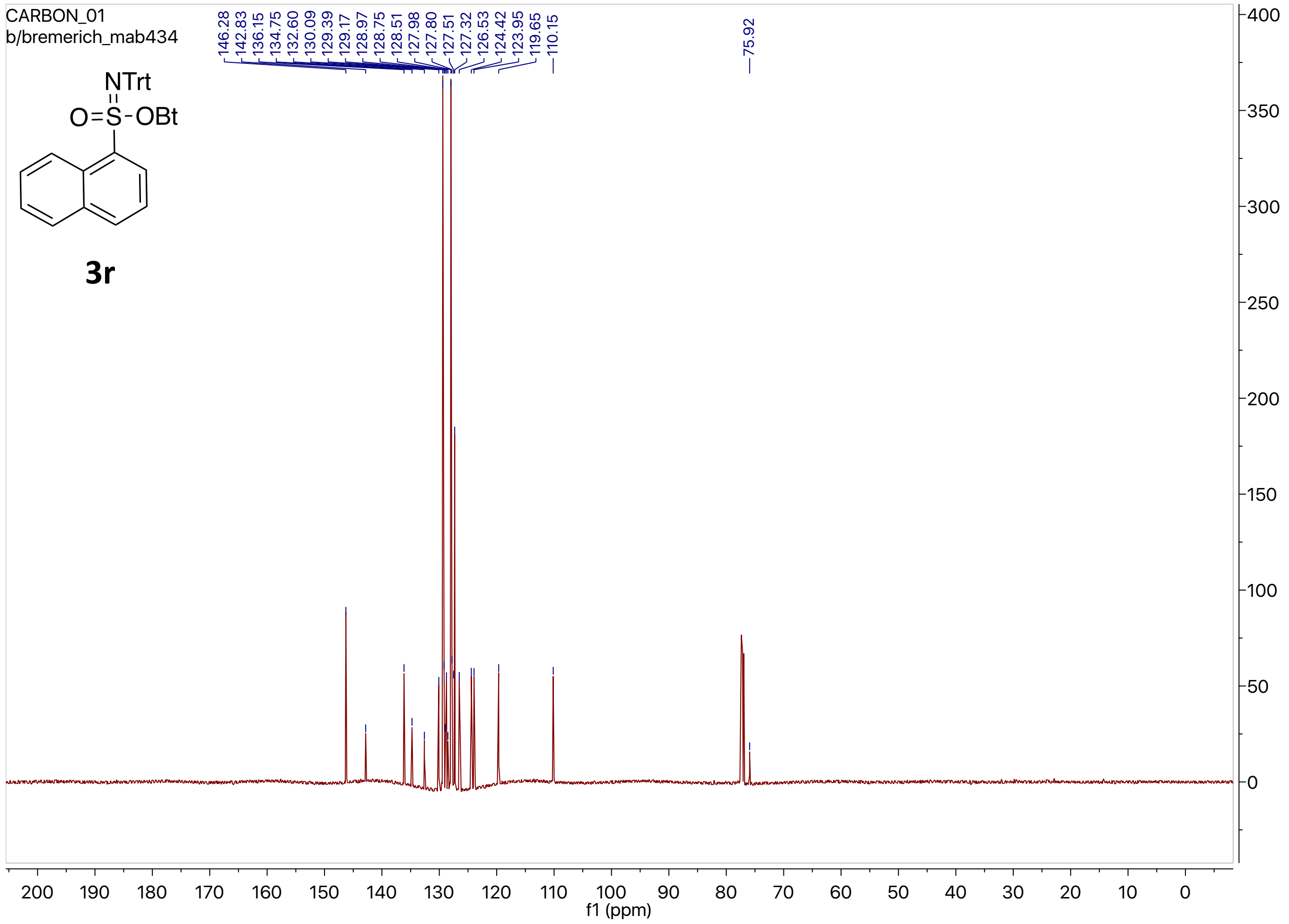
CARBON_01
b/bremerich_mab434
NTrt
O=S-OBt
146.28
142.83
136.15
134.75
132.60
130.09
129.39
129.17
128.97
128.75
128.51
127.98
127.80
127.51
127.32
126.53
124.42
123.95
119.65
110.15
75.92
f1 (ppm)

3r

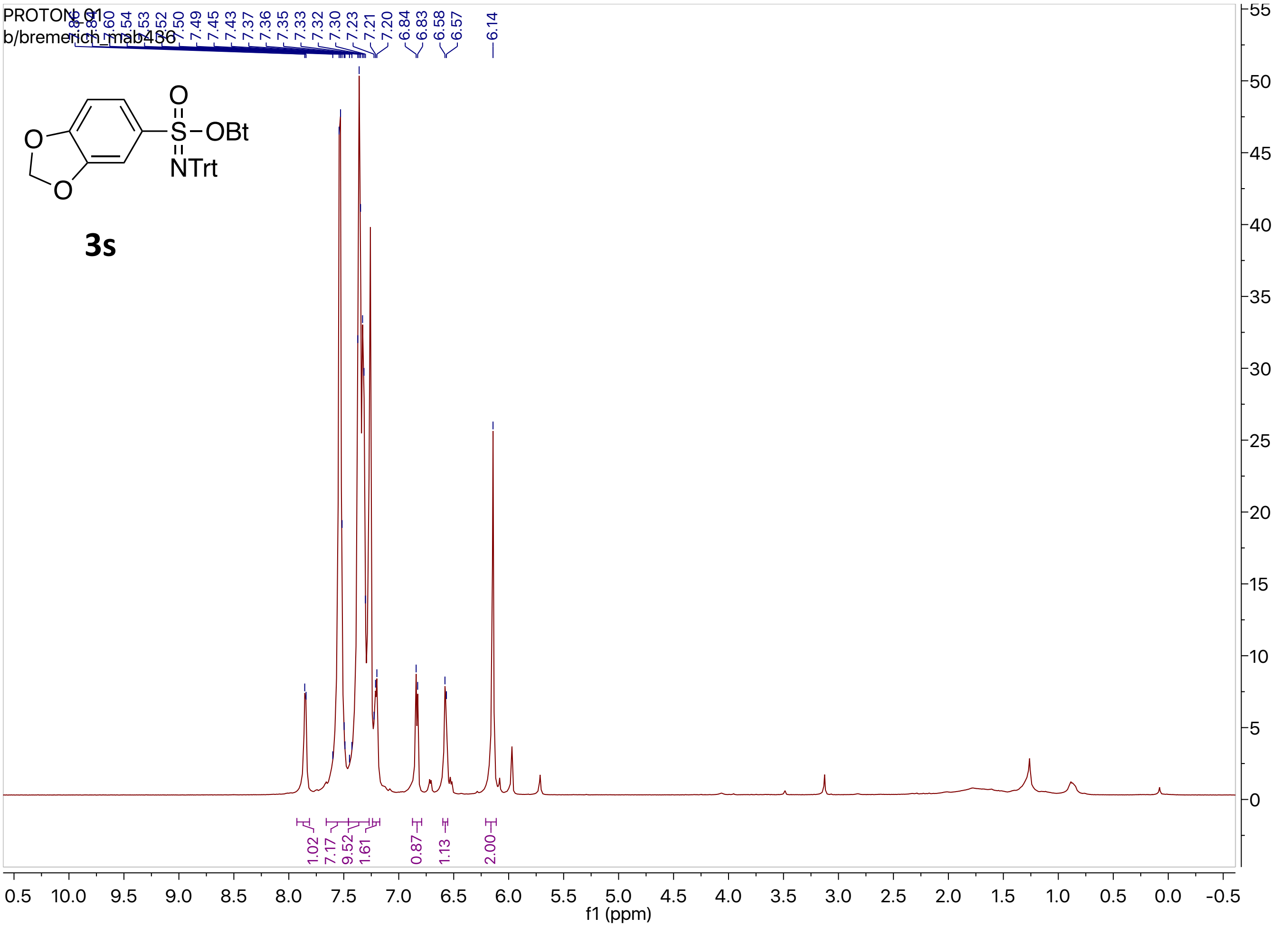
PROTON_01
b/bremerich_mab436
7.60
7.54
7.53
7.52
7.50
7.49
7.45
7.43
7.37
7.36
7.35
7.33
7.32
7.30
7.23
7.21
7.20
6.84
6.83
6.58
6.57
6.14
O
S
OBt
NTrt
O
O
3s
1.02
7.17
9.52
1.61
0.87
1.13
2.00
f1 (ppm)

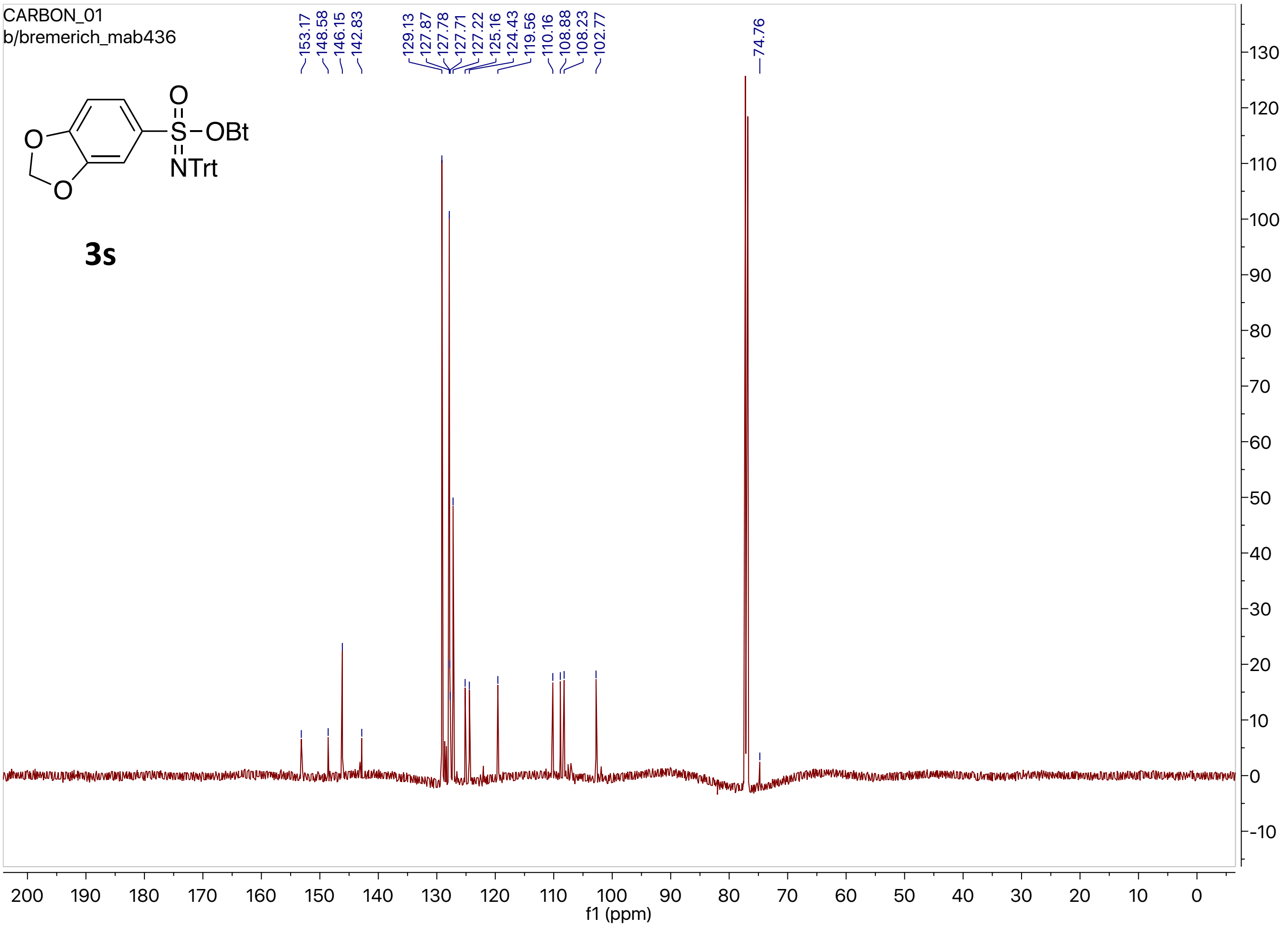
CARBON_01
b/bremerich_mab436
153.17
148.58
146.15
142.83
129.13
127.87
127.78
127.71
127.22
125.16
124.43
119.56
110.16
108.88
108.23
102.77
74.76
O
S
OBt
NTrt
O
O
3s
f1 (ppm)

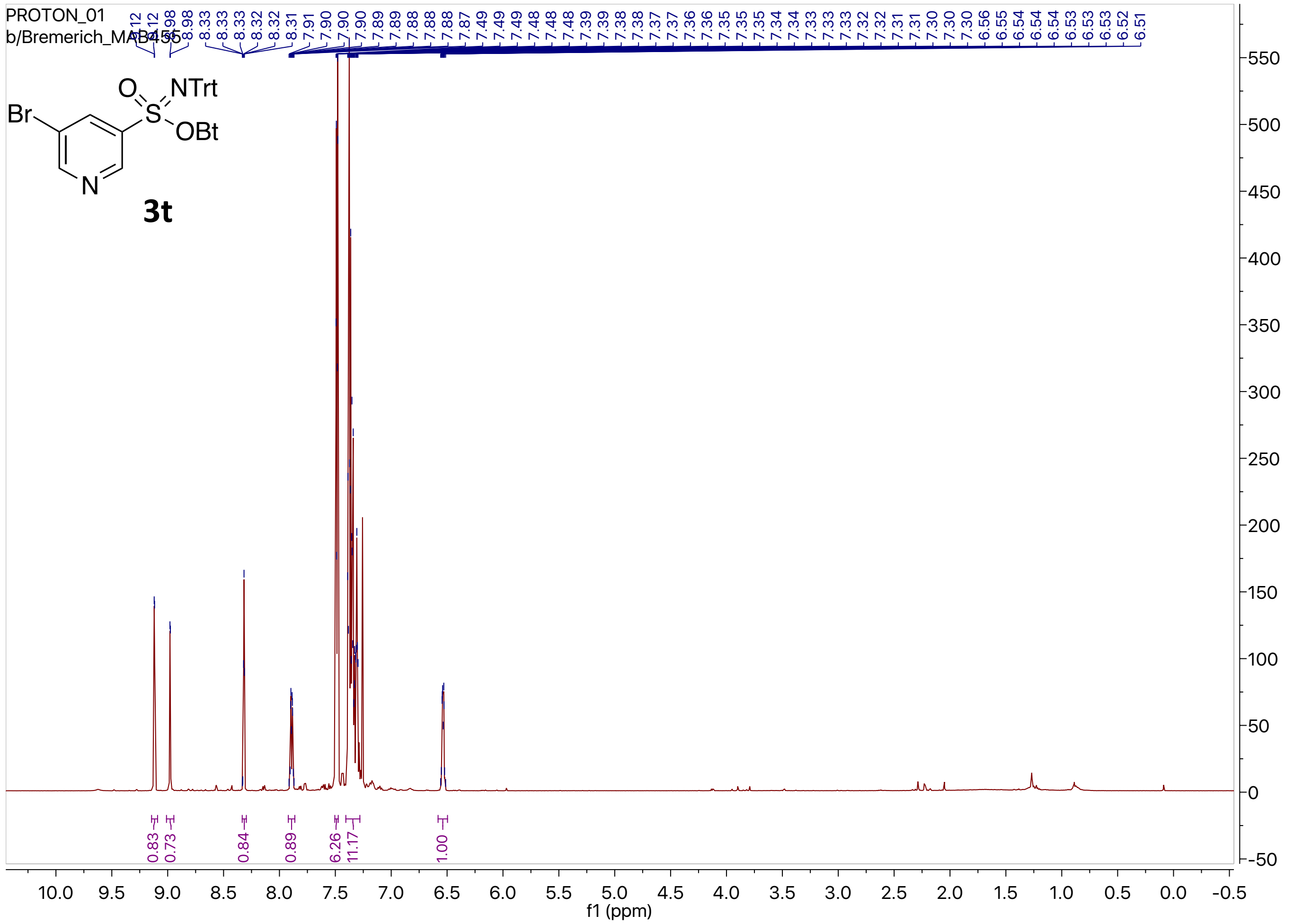
PROTON_01
b/Bremerich_MAB455
9.12 9.12 8.98 8.98 8.33 8.33 8.32 8.32 8.31 7.91 7.90 7.90 7.90 7.89 7.89 7.88 7.88 7.88 7.87 7.49 7.49 7.49 7.48 7.48 7.48 7.39 7.39 7.38 7.38 7.37 7.37 7.36 7.36 7.35 7.35 7.35 7.34 7.34 7.33 7.33 7.33 7.32 7.32 7.31 7.31 7.30 7.30 6.56 6.55 6.54 6.54 6.53 6.53 6.52 6.51
Br
NTrt
OBt
N
3t
0.83 0.73 0.84 0.89 6.26 11.17 1.00
f1 (ppm)

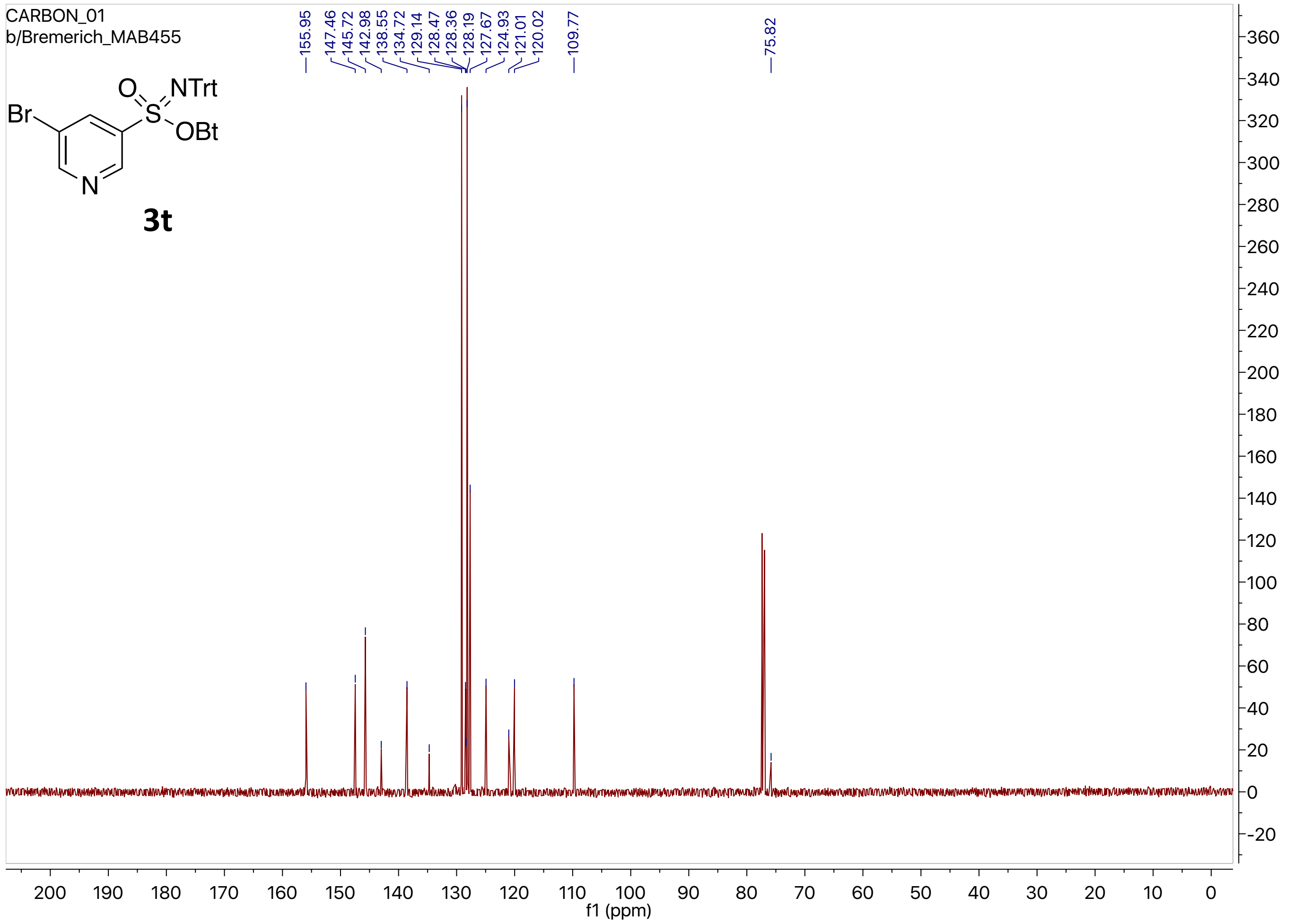
CARBON_01
b/Bremerich_MAB455
155.95 147.46 145.72 142.98 138.55 134.72 129.14 128.47 128.36 128.19 127.67 124.93 121.01 120.02 109.77 75.82
Br
NTrt
OBt
N
3t
f1 (ppm)

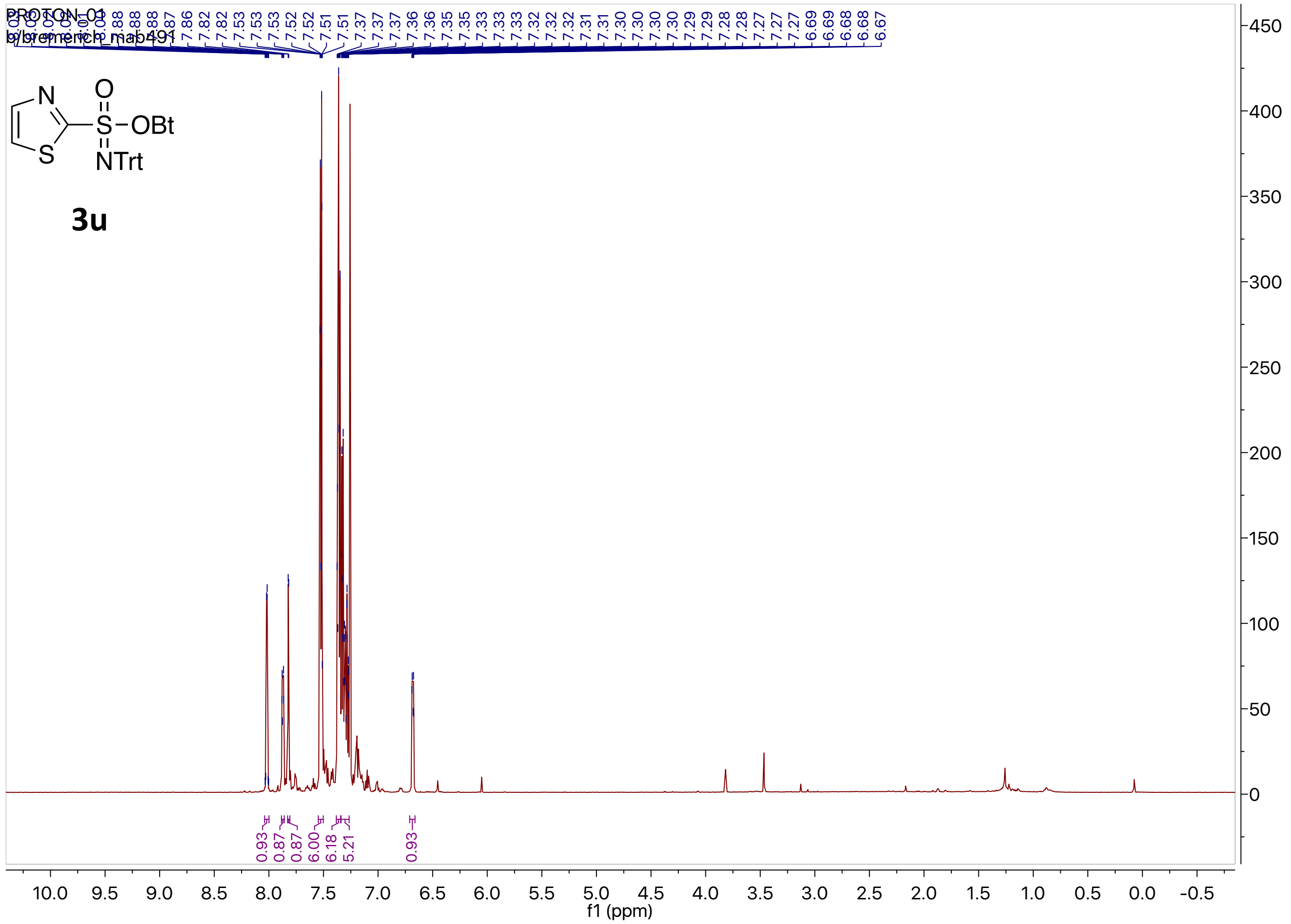
PROTON_01
b/bremerich_mab491
7.88 7.88 7.88 7.87 7.86 7.82 7.82 7.53 7.53 7.52 7.52 7.51 7.51 7.37 7.37 7.37 7.36 7.36 7.35 7.35 7.33 7.33 7.33 7.32 7.32 7.32 7.31 7.30 7.30 7.30 7.30 7.29 7.29 7.28 7.28 7.27 7.27 7.27 6.69 6.69 6.68 6.67
N
S
O
S
OBt
NTrt
3u
0.93 0.87 0.87 6.00 6.18 5.21 0.93
f1 (ppm)

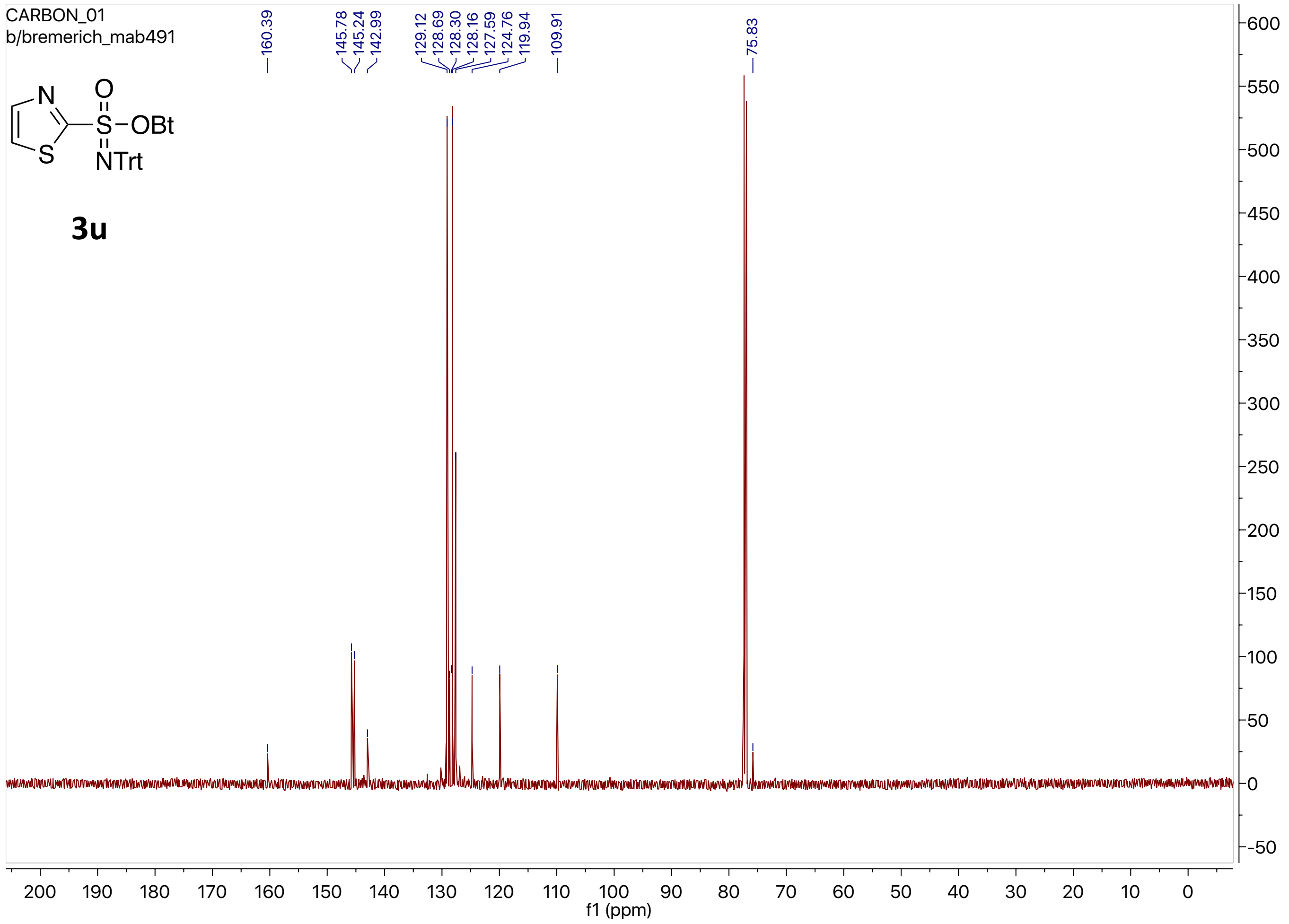
CARBON_01
b/bremerich_mab491
160.39
145.78 145.24 142.99
129.12 128.69 128.30 128.16 127.59 124.76 119.94
109.91
75.83
N
S
O
S
OBt
NTrt
3u
f1 (ppm)

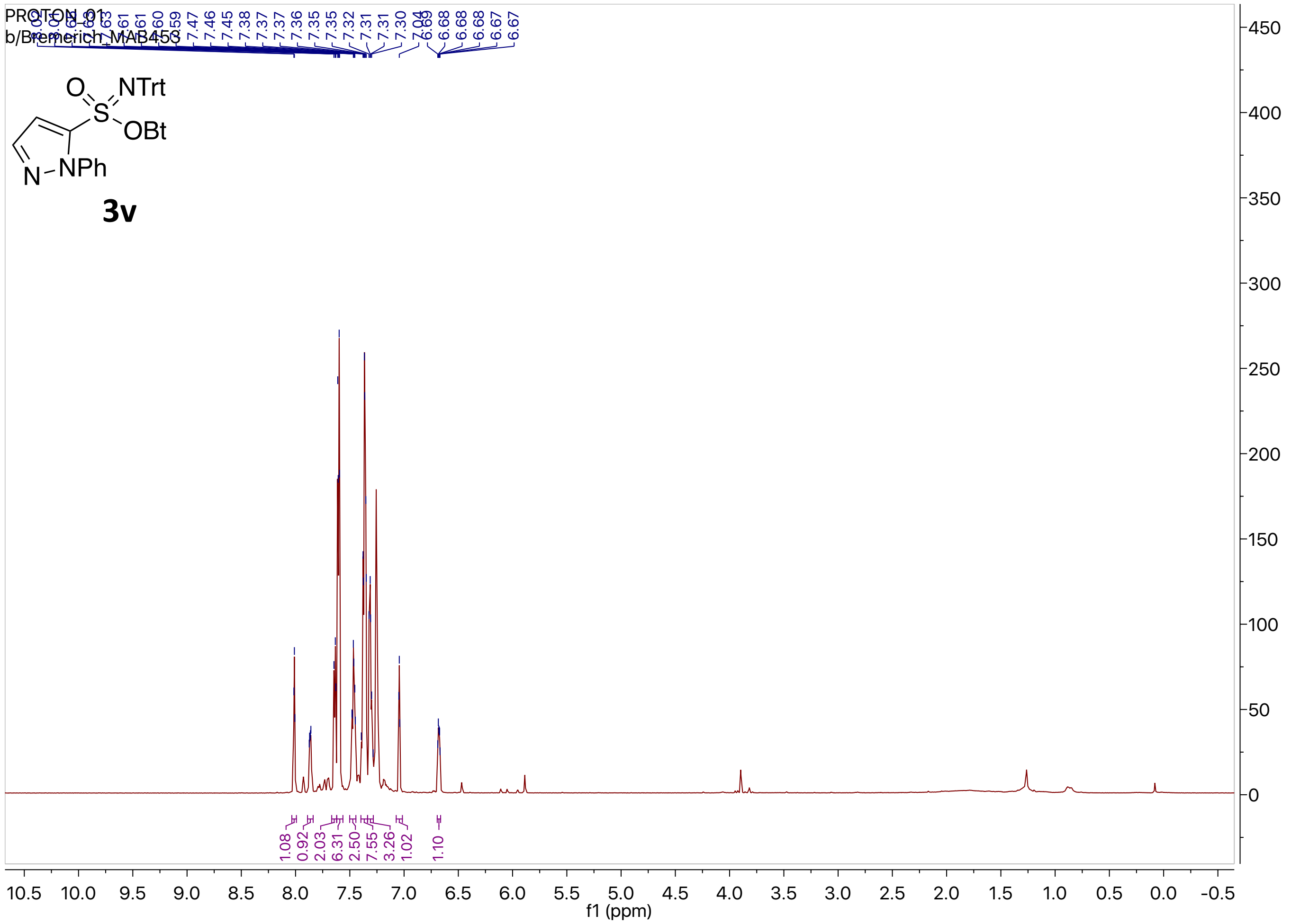

PROTON_01
b/Bremerich_MAB453
NTrt
OBt
NPh
3v
f1 (ppm)

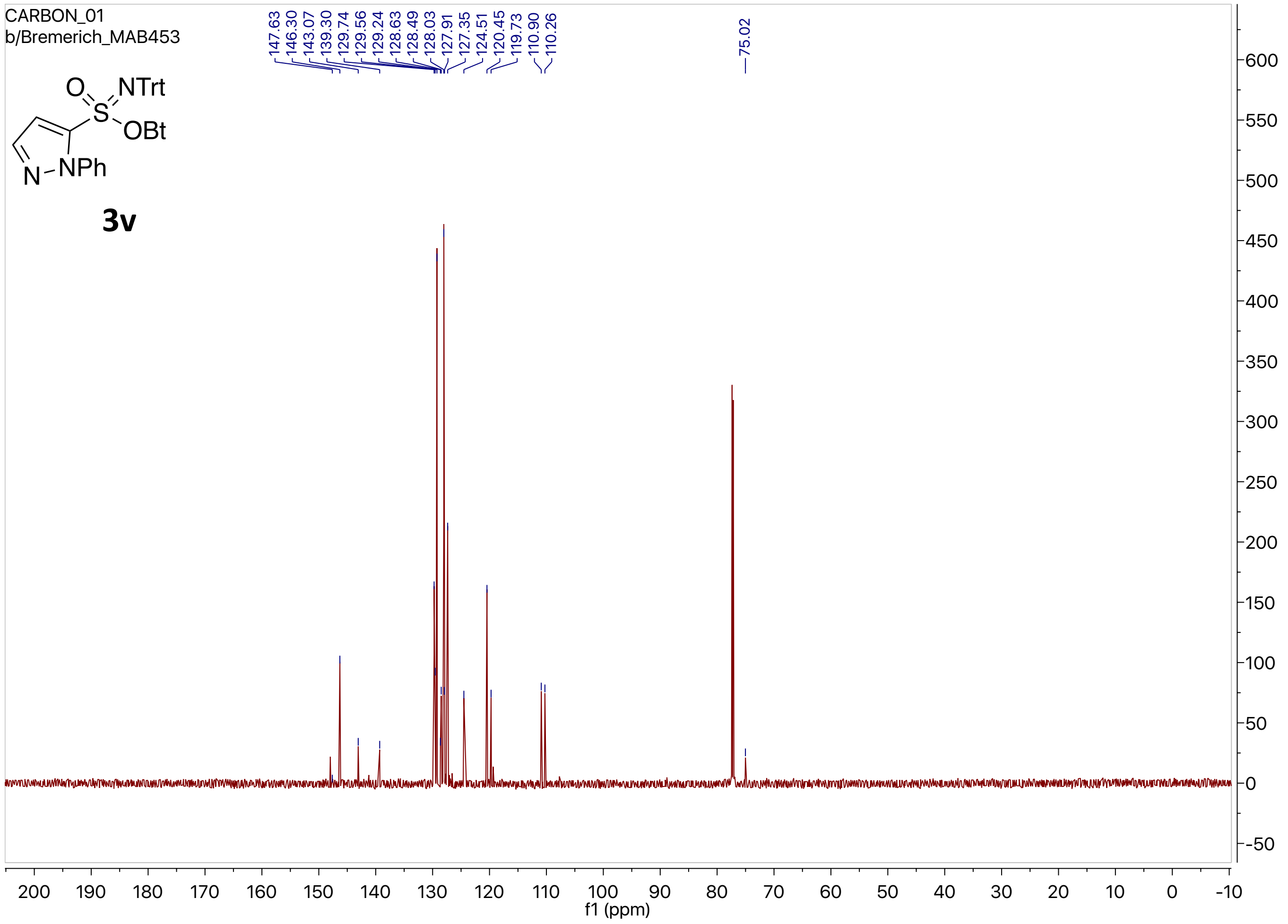

CARBON_01
b/Bremerich_MAB453
147.63
146.30
143.07
139.30
129.74
129.56
129.24
128.63
128.49
128.03
127.91
127.35
124.51
120.45
119.73
110.90
110.26
75.02
NTrt
OBt
NPh
3v
f1 (ppm)

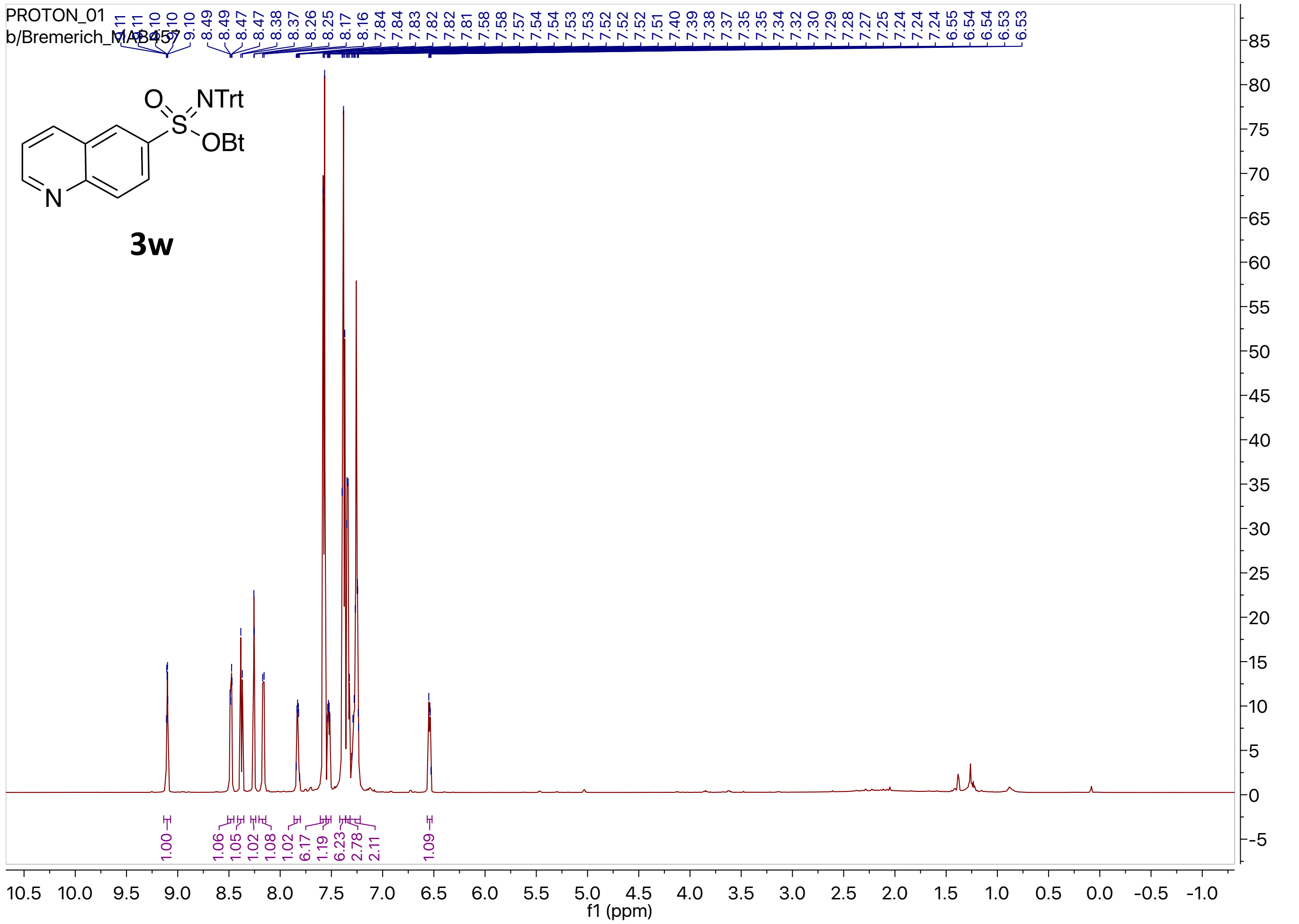
PROTON_01
b/Bremerich_MAB457
3w
9.11 9.11 9.10 9.10 9.10 8.49 8.49 8.47 8.47 8.38 8.37 8.26 8.25 8.17 8.16 7.84 7.84 7.83 7.82 7.82 7.81 7.58 7.58 7.57 7.54 7.54 7.53 7.53 7.52 7.52 7.52 7.51 7.40 7.39 7.38 7.37 7.35 7.35 7.34 7.32 7.30 7.29 7.28 7.27 7.25 7.24 7.24 7.24 6.55 6.54 6.54 6.53 6.53
1.00 1.06 1.05 1.02 1.08 1.02 6.17 1.19 6.23 2.78 2.11 1.09
f1 (ppm)

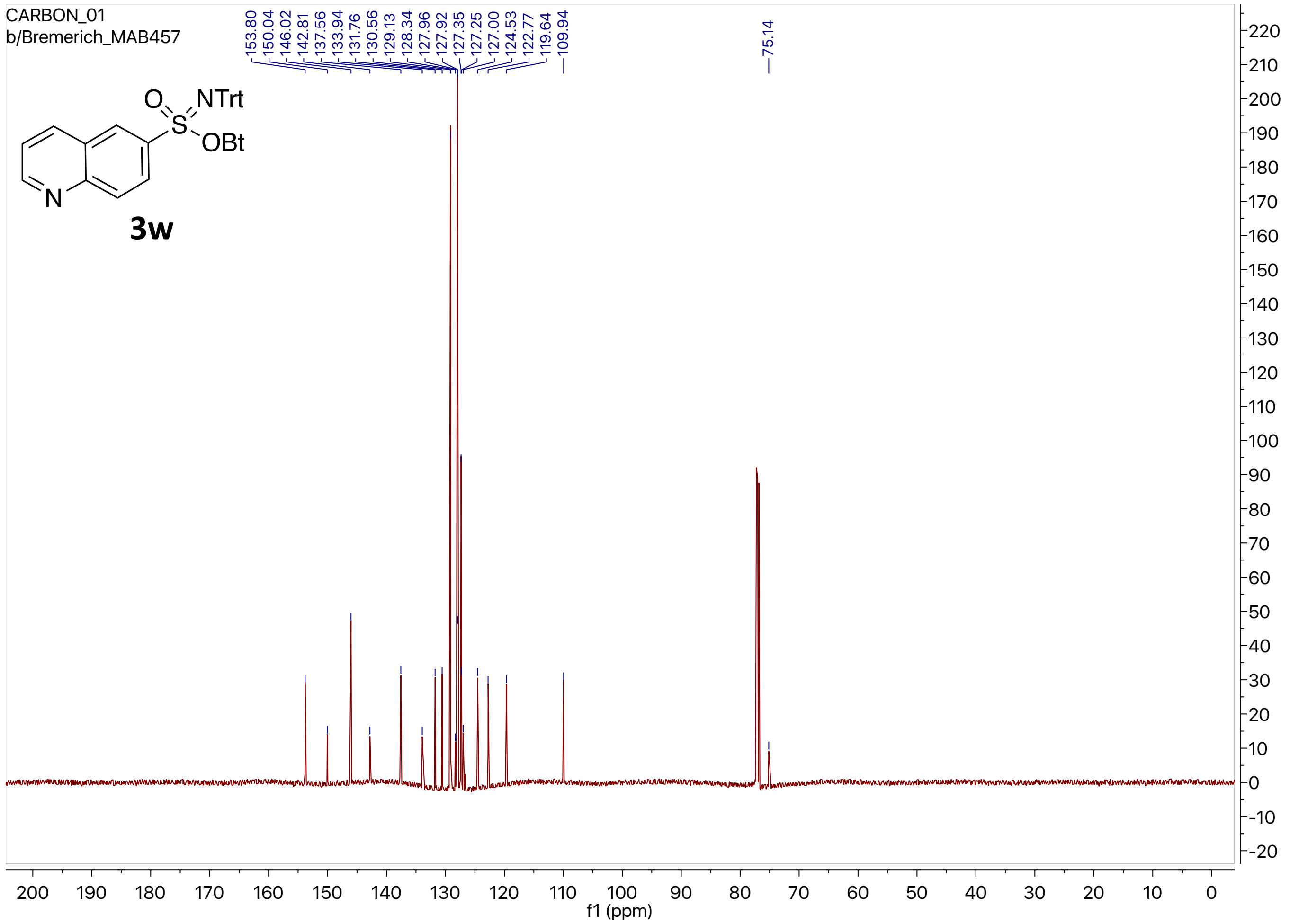
CARBON_01
b/Bremerich_MAB457
3w
153.80 150.04 146.02 142.81 137.56 133.94 131.76 130.56 129.13 128.34 127.96 127.92 127.35 127.25 127.00 124.53 122.77 119.64 109.94
75.14
f1 (ppm)

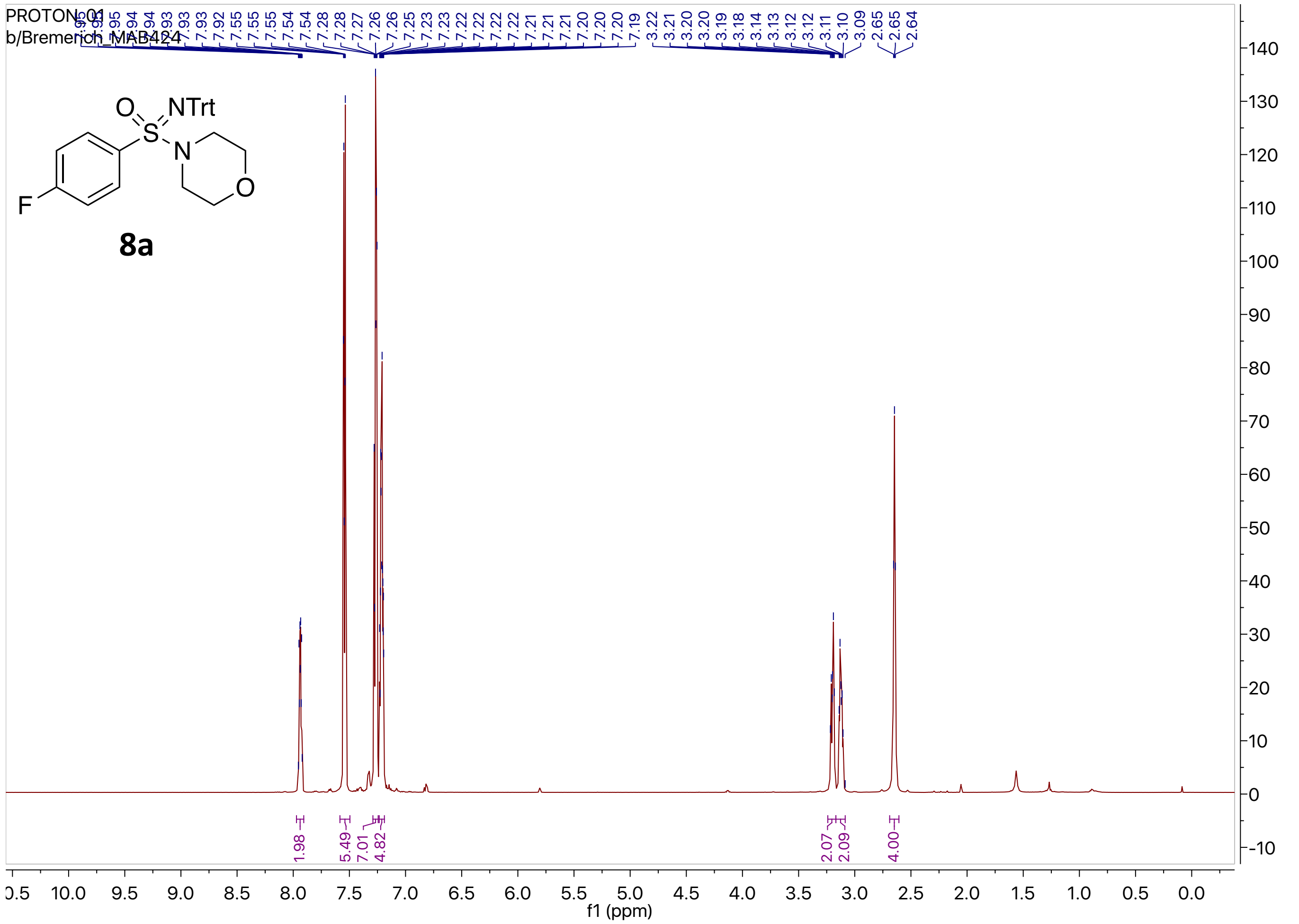
PROTON_01
b/Bremerich_MAB424
NTrt
8a
f1 (ppm)

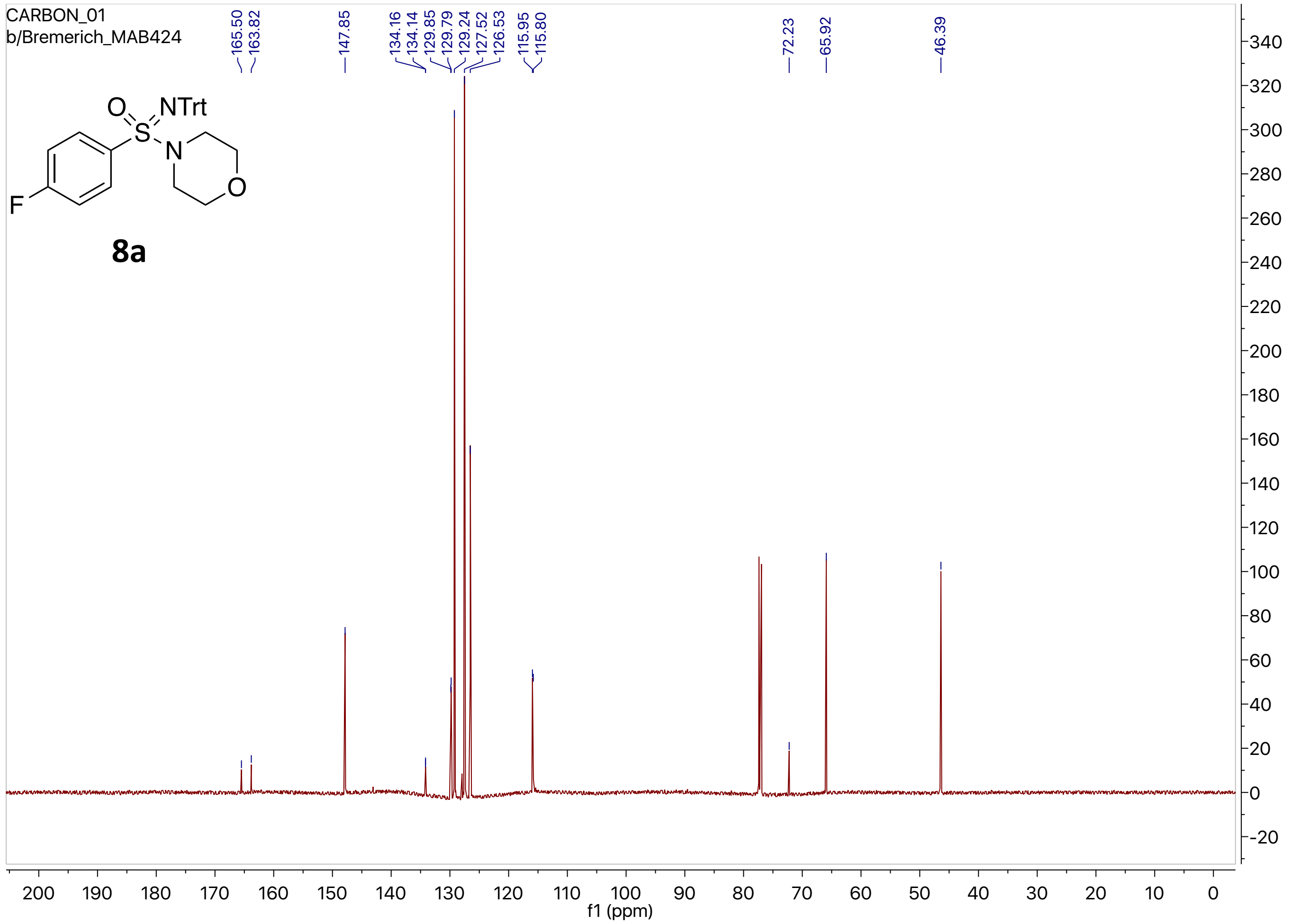
CARBON_01
b/Bremerich_MAB424
165.50
163.82
147.85
134.16
134.14
129.85
129.79
129.24
127.52
126.53
115.95
115.80
72.23
65.92
46.39
NTrt
8a
f1 (ppm)

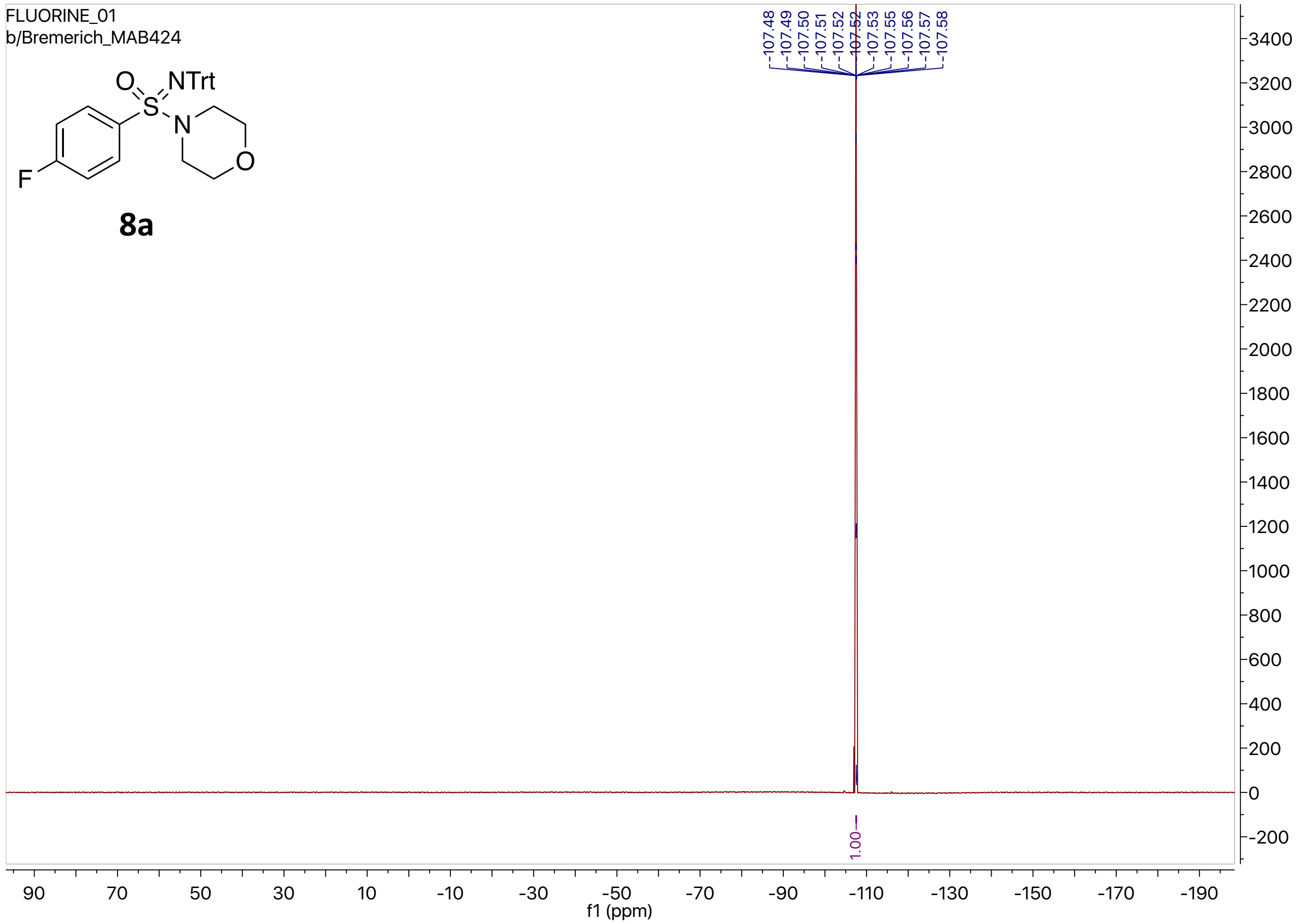
FLUORINE_01
b/Bremerich_MAB424
O
NTrt
S
N
O
F
8a
-107.48
-107.49
-107.50
-107.51
-107.52
-107.52
-107.53
-107.55
-107.56
-107.57
-107.58
1.00
90
70
50
30
10
-10
-30
-50
-70
-90
-110
-130
-150
-170
-190
f1 (ppm)
3400
3200
3000
2800
2600
2400
2200
2000
1800
1600
1400
1200
1000
800
600
400
200
0
-200

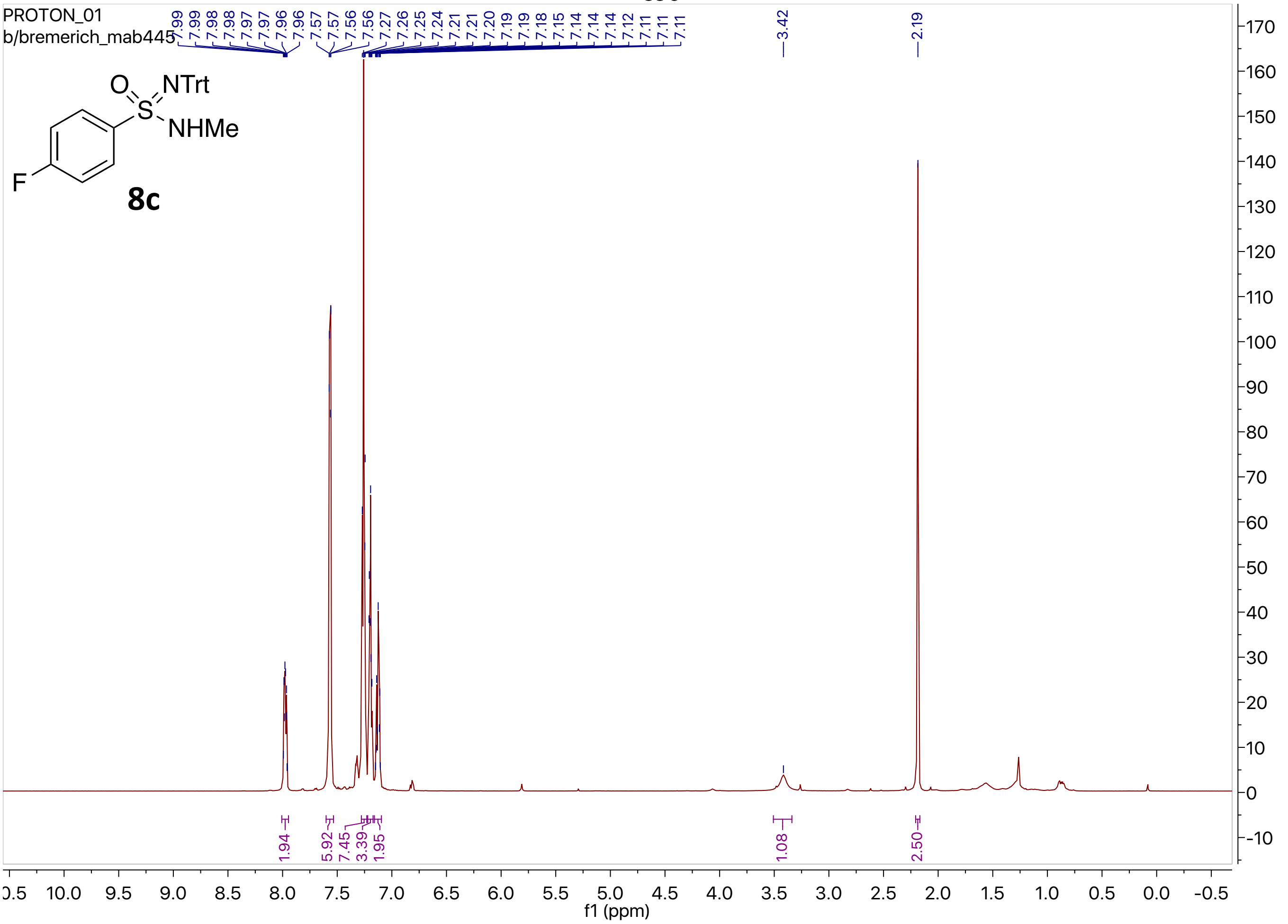
PROTON_01
b/bremerich_mab445
O NTrt
S
NHMe
F
8c
7.99 7.99 7.98 7.98 7.97 7.97 7.96 7.96 7.57 7.57 7.56 7.56 7.27 7.26 7.25 7.24 7.21 7.21 7.20 7.19 7.19 7.18 7.15 7.14 7.14 7.14 7.12 7.11 7.11 7.11
3.42
2.19
1.94
5.92
7.45
3.39
1.95
1.08
2.50
f1 (ppm)

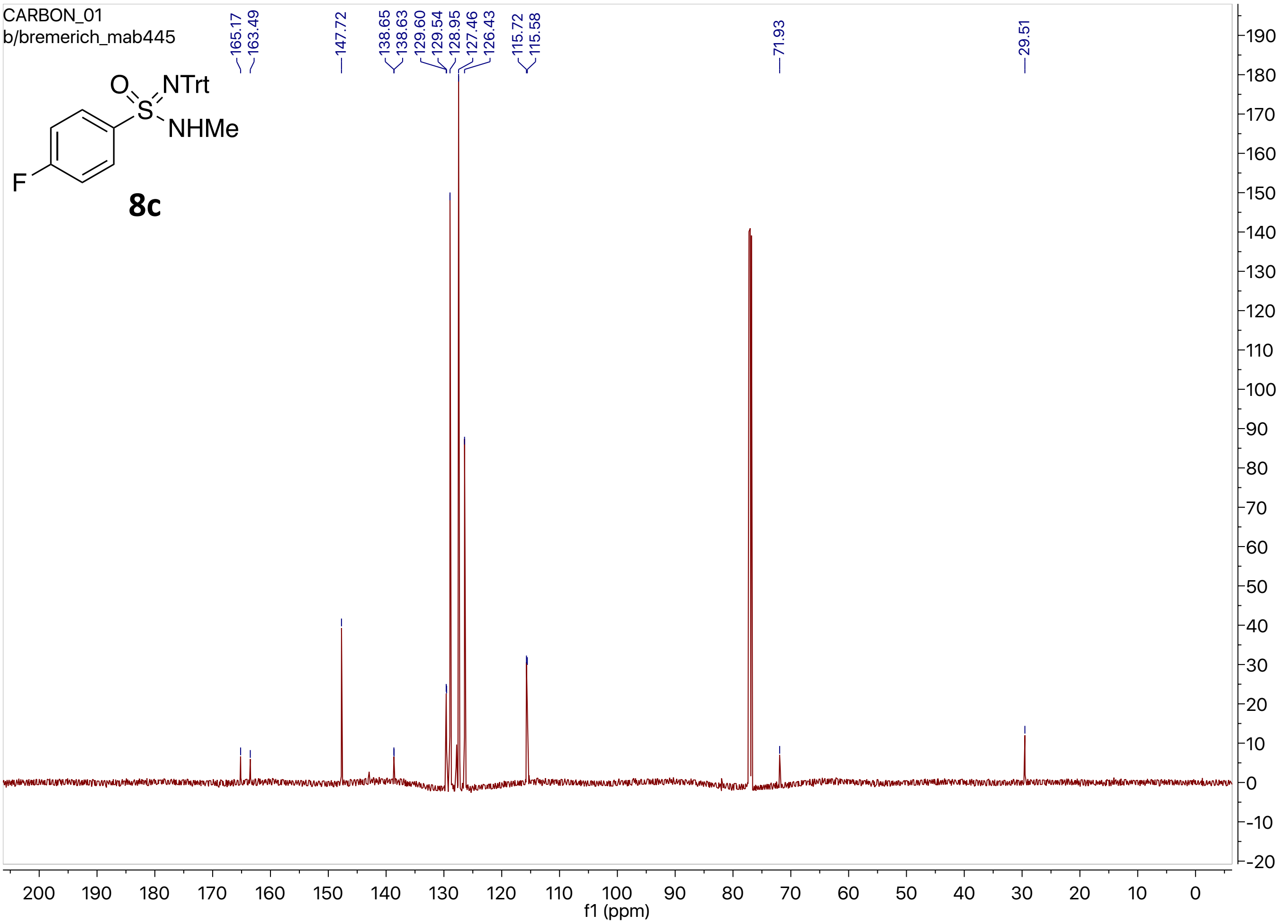
CARBON_01
b/bremerich_mab445
165.17
163.49
147.72
138.65
138.63
129.60
129.54
128.95
127.46
126.43
115.72
115.58
71.93
29.51
O NTrt
S
NHMe
F
8c
f1 (ppm)

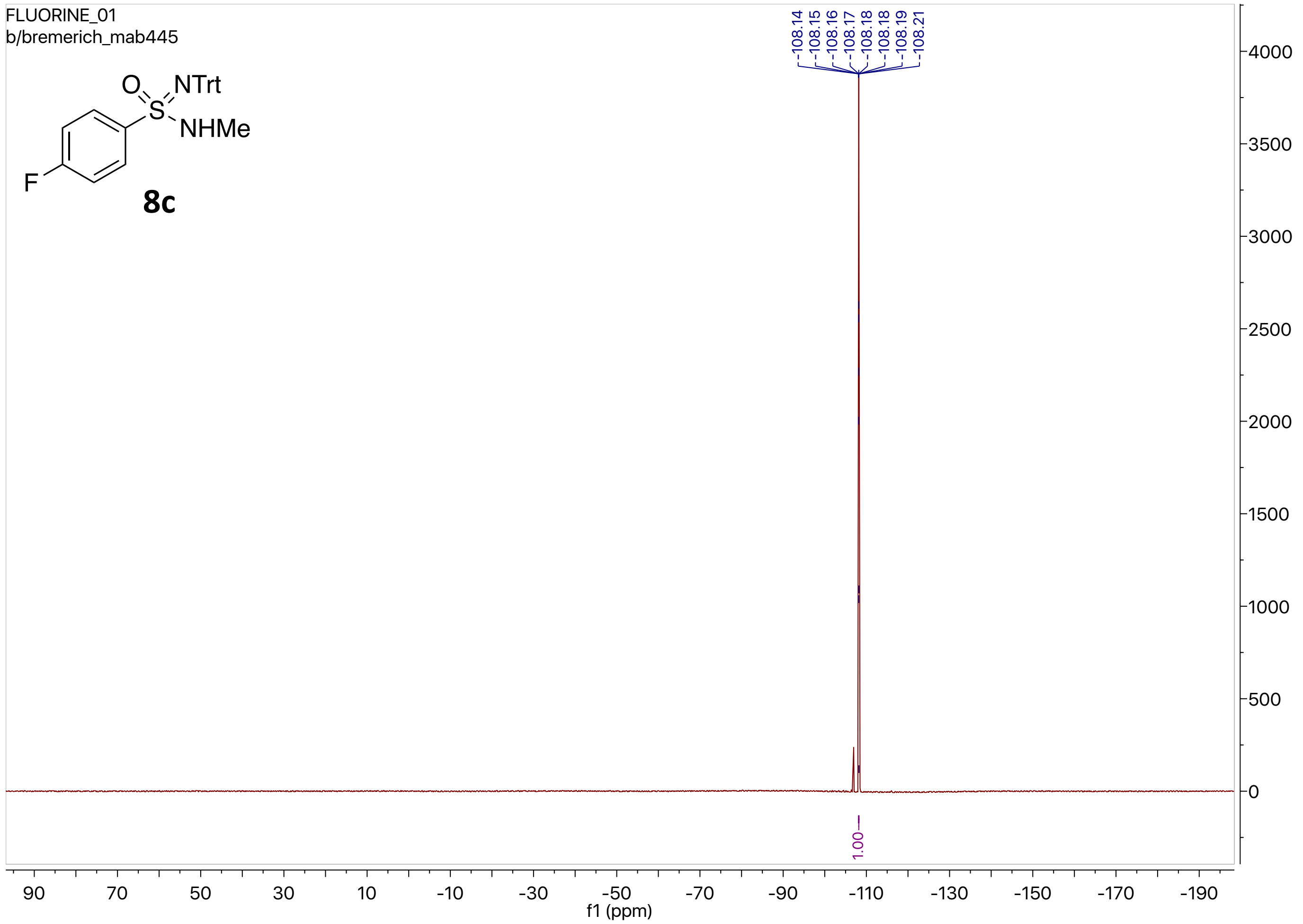
FLUORINE_01
b/bremerich_mab445
NTrt
NHMe
F
8c
-108.14
-108.15
-108.16
-108.17
-108.18
-108.18
-108.19
-108.21
1.00
4000
3500
3000
2500
2000
1500
1000
500
0
90 70 50 30 10 -10 -30 -50 -70 -90 -110 -130 -150 -170 -190
f1 (ppm)

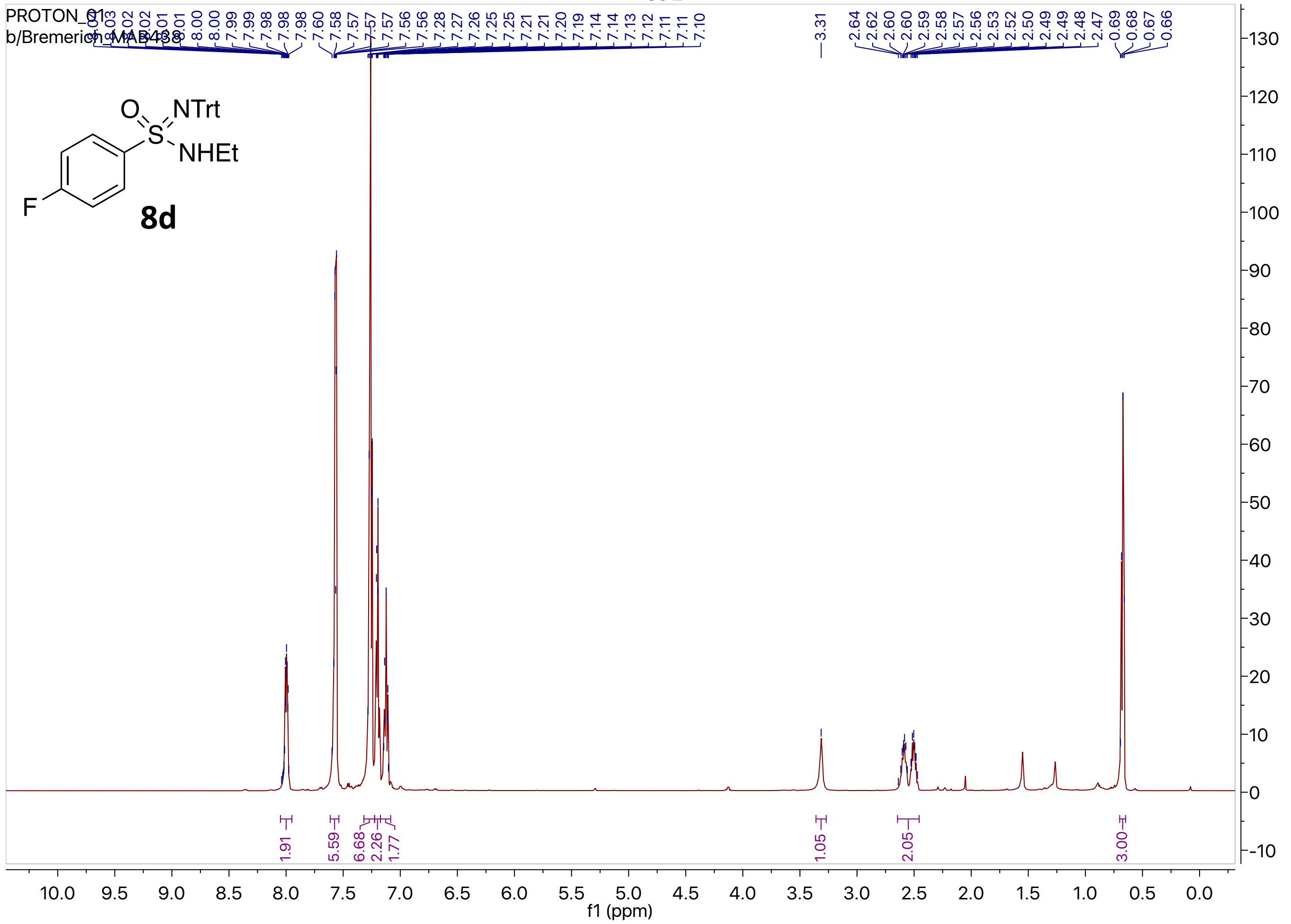
PROTON_01
b/Bremerich_MAB438
8.03 8.02 8.02 8.01 8.01 8.00 7.99 7.99 7.98 7.98 7.98 7.60 7.58 7.57 7.57 7.57 7.56 7.56 7.28 7.27 7.26 7.25 7.25 7.21 7.21 7.20 7.19 7.14 7.14 7.13 7.12 7.11 7.11 7.10
3.31
2.64 2.62 2.60 2.60 2.59 2.58 2.57 2.56 2.53 2.52 2.50 2.49 2.49 2.48 2.47 0.69 0.68 0.67 0.66
O
NTrt
S
NHEt
F
8d
1.91
5.59
6.68
2.26
1.77
1.05
2.05
3.00
f1 (ppm)

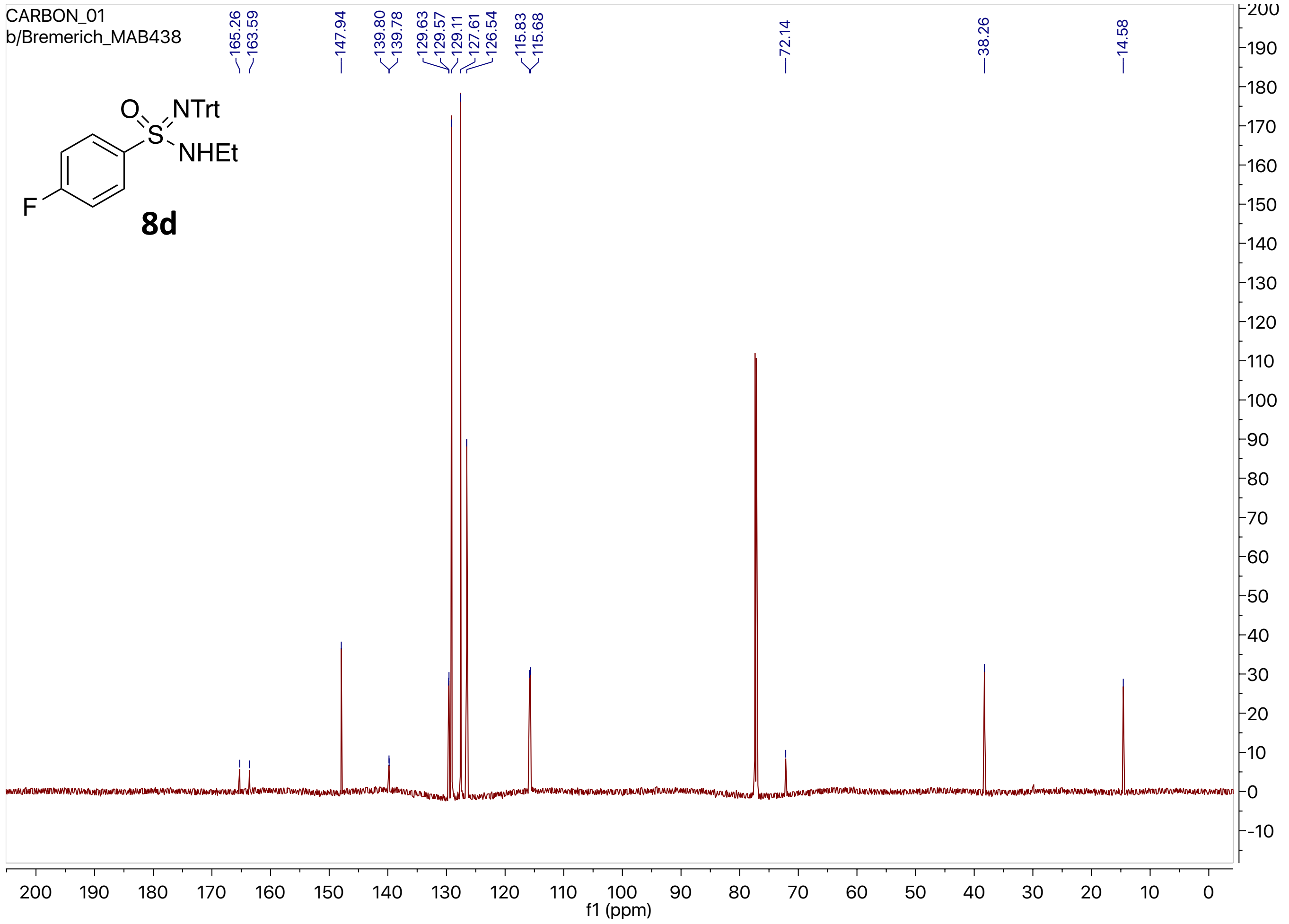
CARBON_01
b/Bremerich_MAB438
165.26 163.59
147.94
139.80 139.78
129.63 129.57 129.11 127.61 126.54
115.83 115.68
72.14
38.26
14.58
O
NTrt
S
NHEt
F
8d
f1 (ppm)

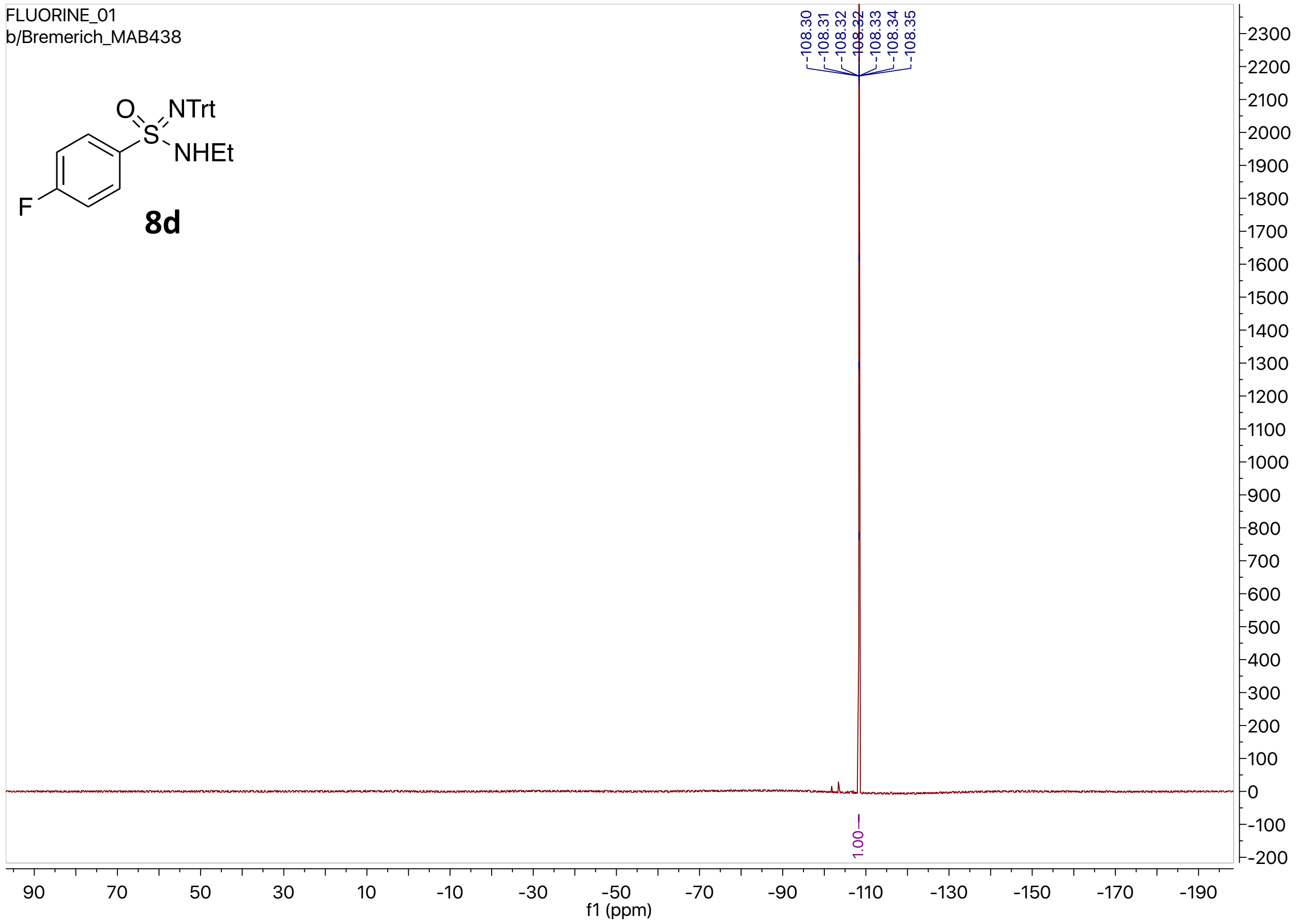
FLUORINE_01
b/Bremerich_MAB438
O
NTrt
S
NHEt
F
8d
-108.30
-108.31
-108.32
-108.32
-108.33
-108.34
-108.35
1.00
f1 (ppm)

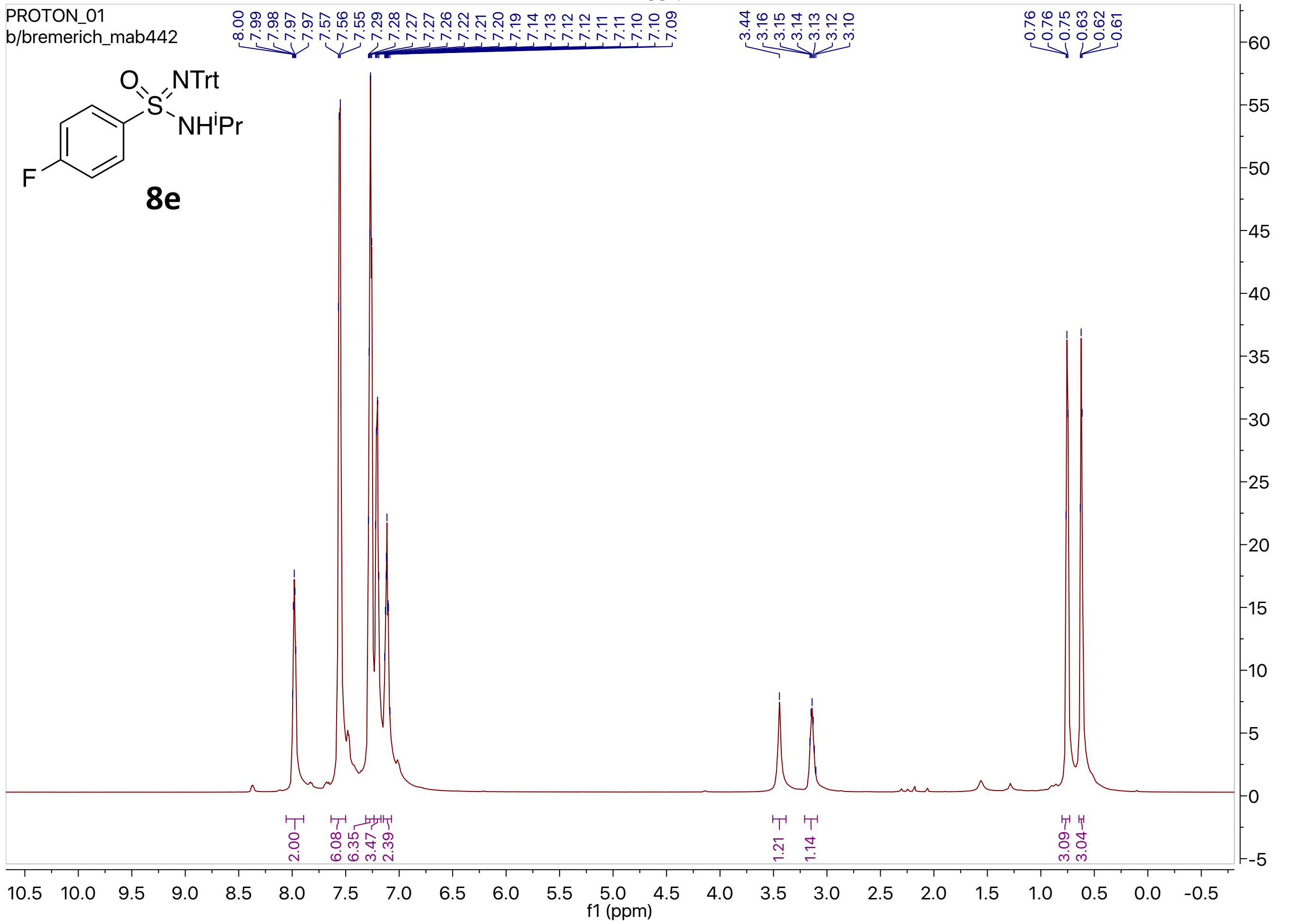
PROTON_01
b/bremerich_mab442
8.00 7.99 7.98 7.97 7.97 7.57 7.56 7.55 7.29 7.28 7.27 7.27 7.26 7.22 7.21 7.20 7.19 7.14 7.13 7.12 7.12 7.11 7.11 7.10 7.10 7.09
3.44 3.16 3.15 3.14 3.13 3.12 3.10
0.76 0.76 0.75 0.63 0.62 0.61
O
S
NTrt
NHiPr
F
8e
2.00 6.08 6.35 3.47 2.39 1.21 1.14 3.09 3.04
f1 (ppm)

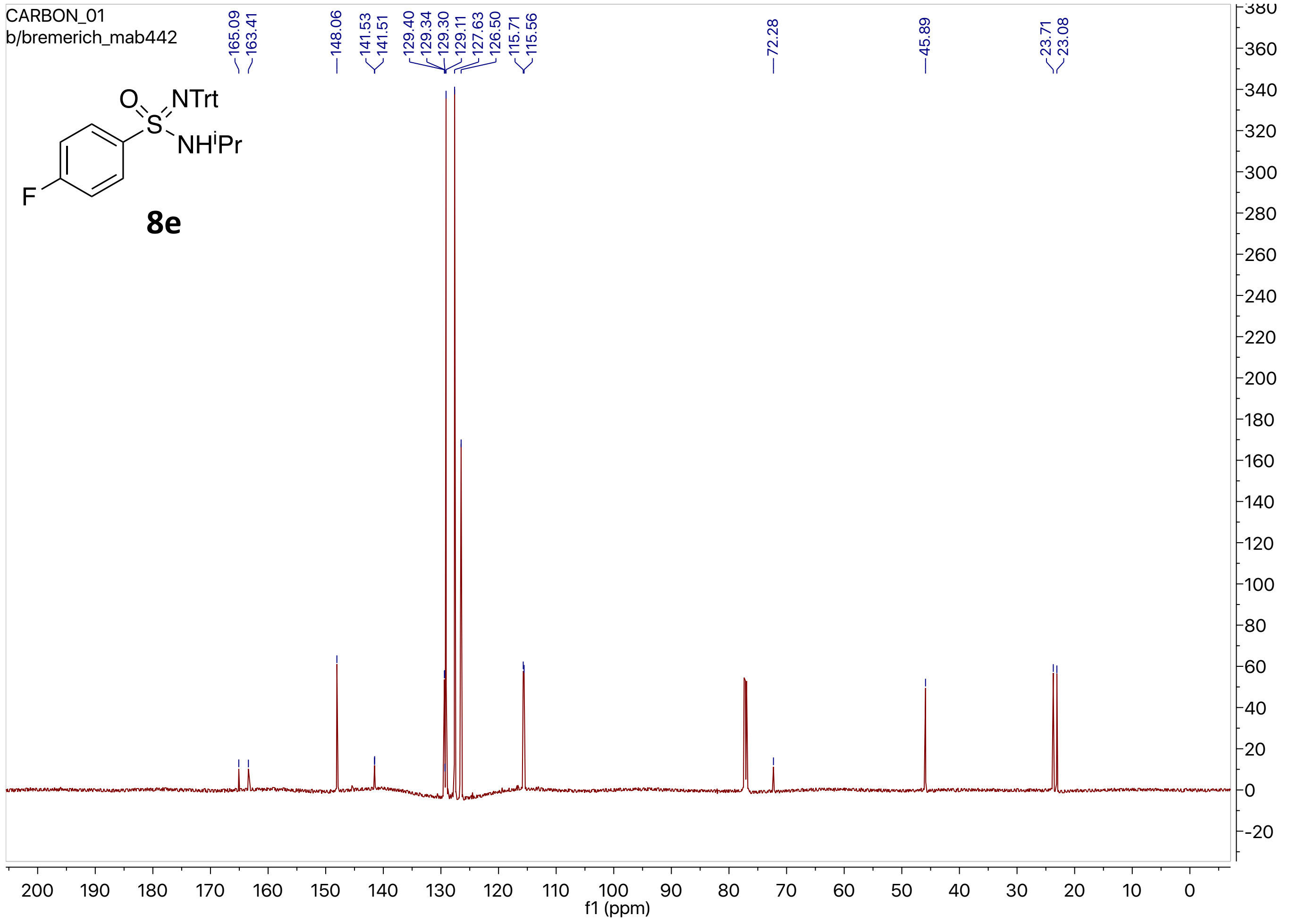
CARBON_01
b/bremerich_mab442
165.09 163.41 148.06 141.53 141.51 129.40 129.34 129.30 129.11 127.63 126.50 115.71 115.56
72.28
45.89
23.71 23.08
O
S
NTrt
NHiPr
F
8e
f1 (ppm)

FLUORINE_01
b/bremerich_mab442

8e

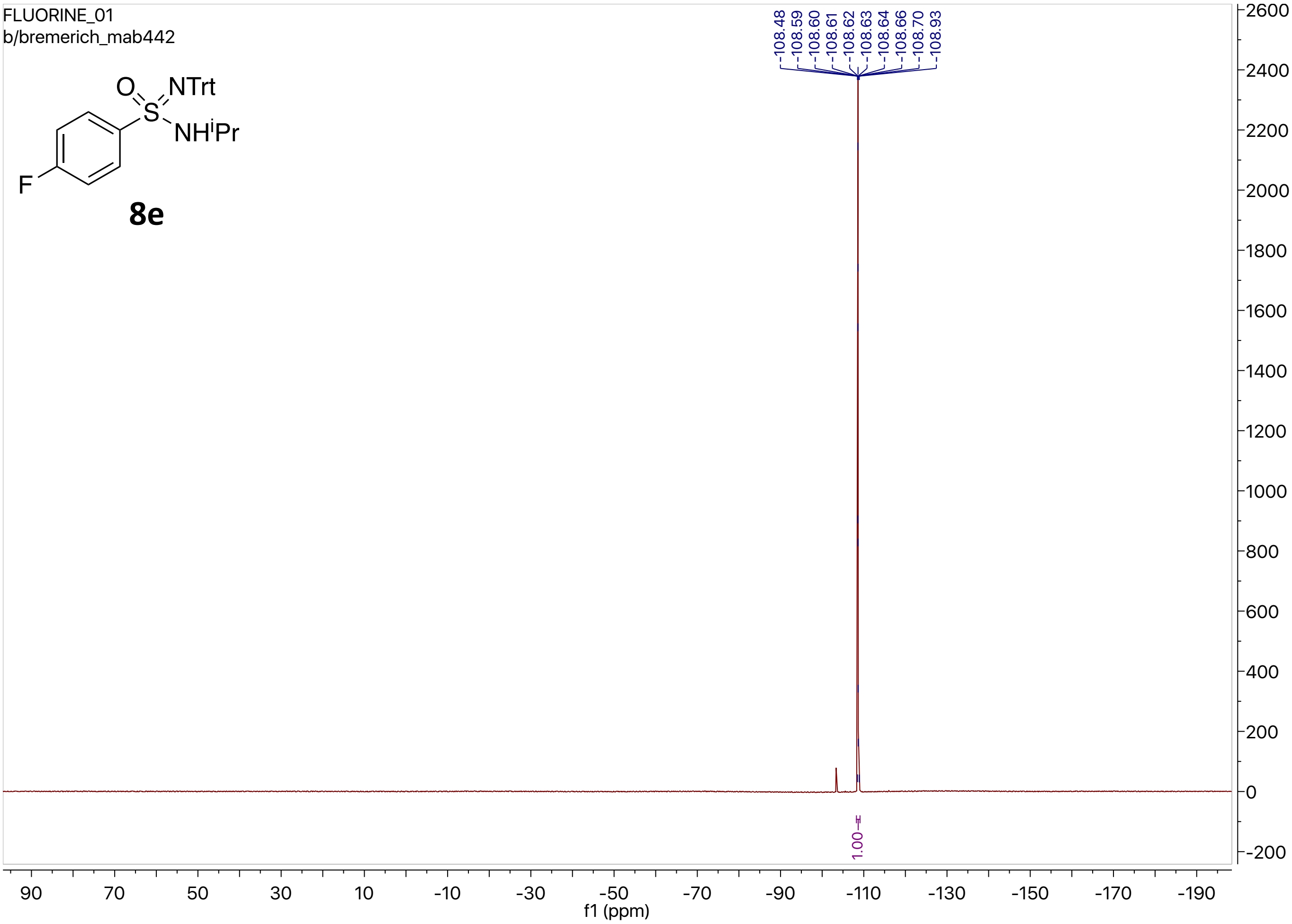

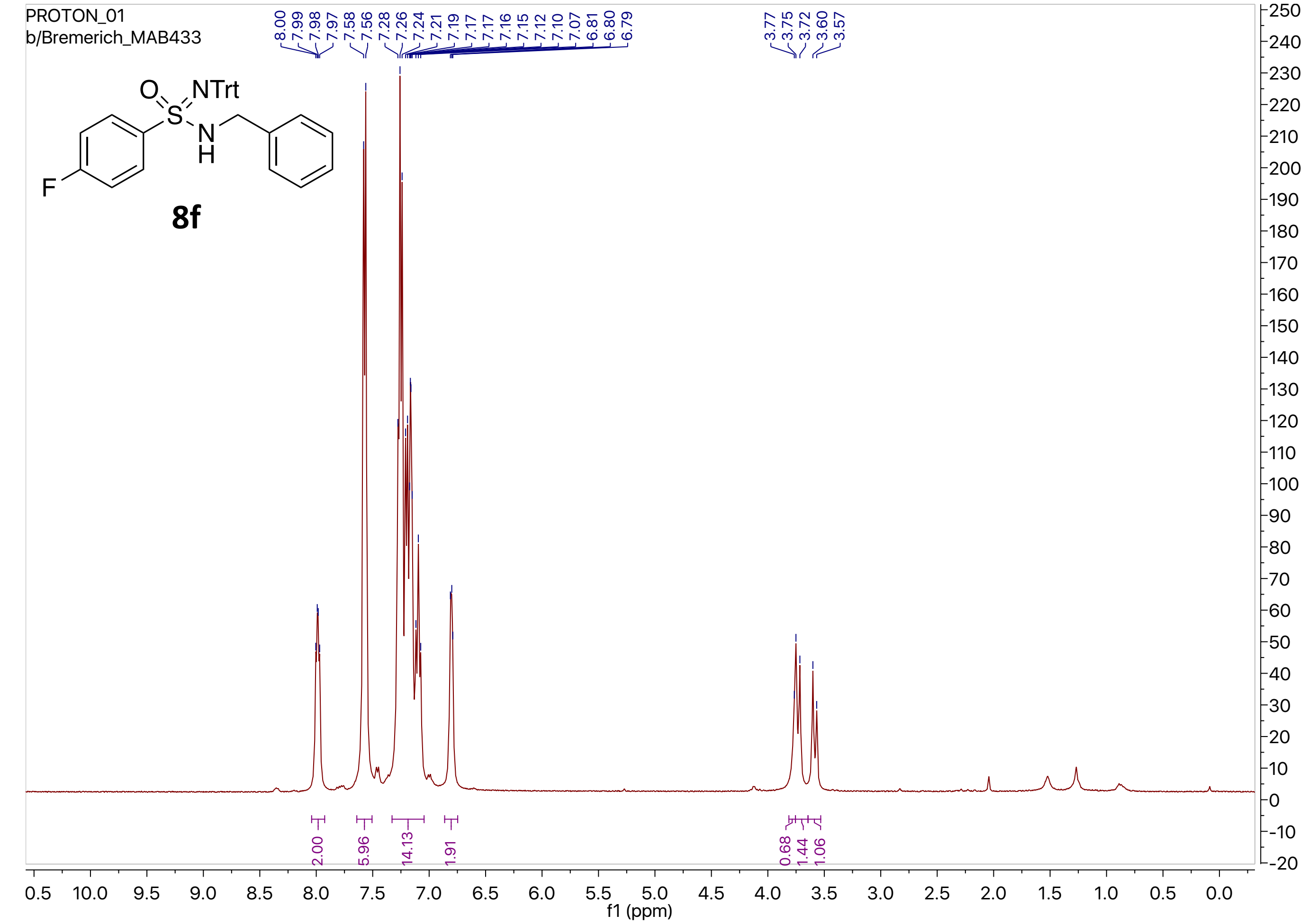
PROTON_01
b/Bremerich_MAB433
O
NTrt
S
N
H
F
8f
8.00 7.99 7.98 7.97 7.58 7.56 7.28 7.26 7.24 7.21 7.19 7.17 7.17 7.16 7.15 7.12 7.10 7.07 6.81 6.80 6.79
3.77 3.75 3.72 3.60 3.57
2.00
5.96
14.13
1.91
0.68
1.44
1.06
f1 (ppm)

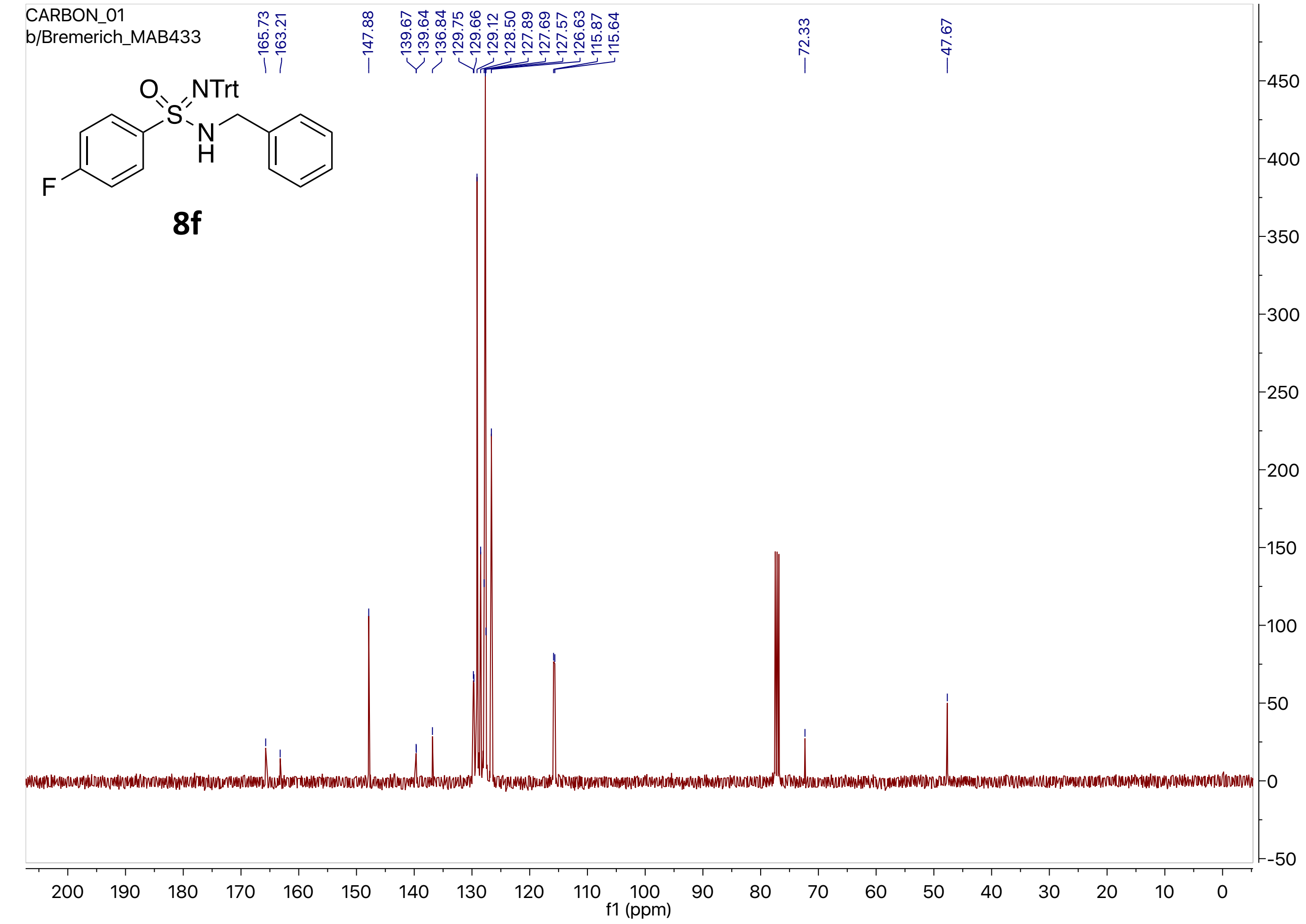
CARBON_01
b/Bremerich_MAB433
O
NTrt
S
N
H
F
8f
165.73 163.21
147.88
139.67 139.64 136.84 129.75 129.66 129.12 128.50 127.89 127.69 127.57 126.63 115.87 115.64
72.33
47.67
f1 (ppm)

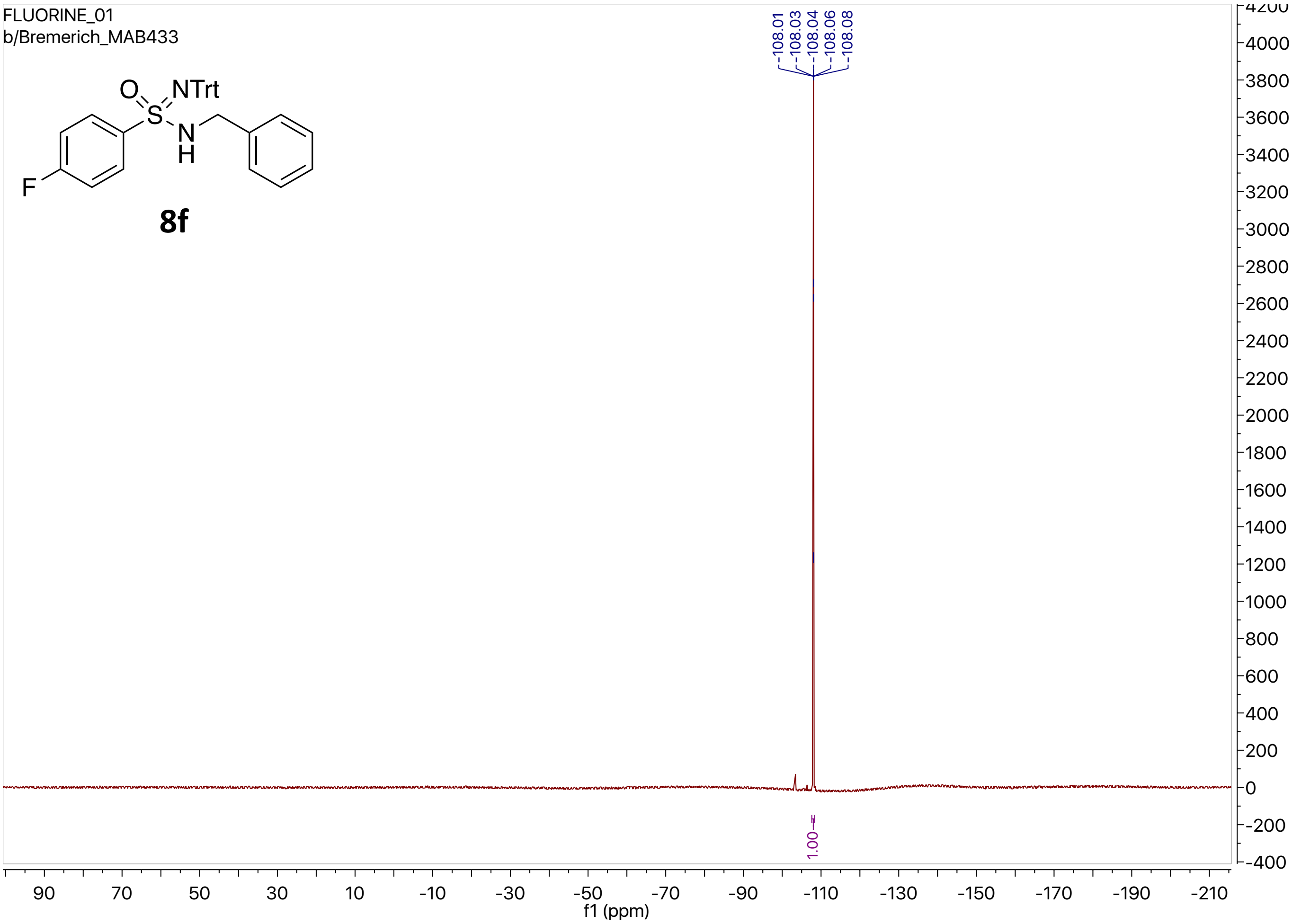
FLUORINE_01
b/Bremerich_MAB433
O
NTrt
S
N
H
F
8f
-108.01
-108.03
-108.04
-108.06
-108.08
1.00
4200
4000
3800
3600
3400
3200
3000
2800
2600
2400
2200
2000
1800
1600
1400
1200
1000
800
600
400
200
0
-200
-400
90 70 50 30 10 -10 -30 -50 -70 -90 -110 -130 -150 -170 -190 -210
f1 (ppm)

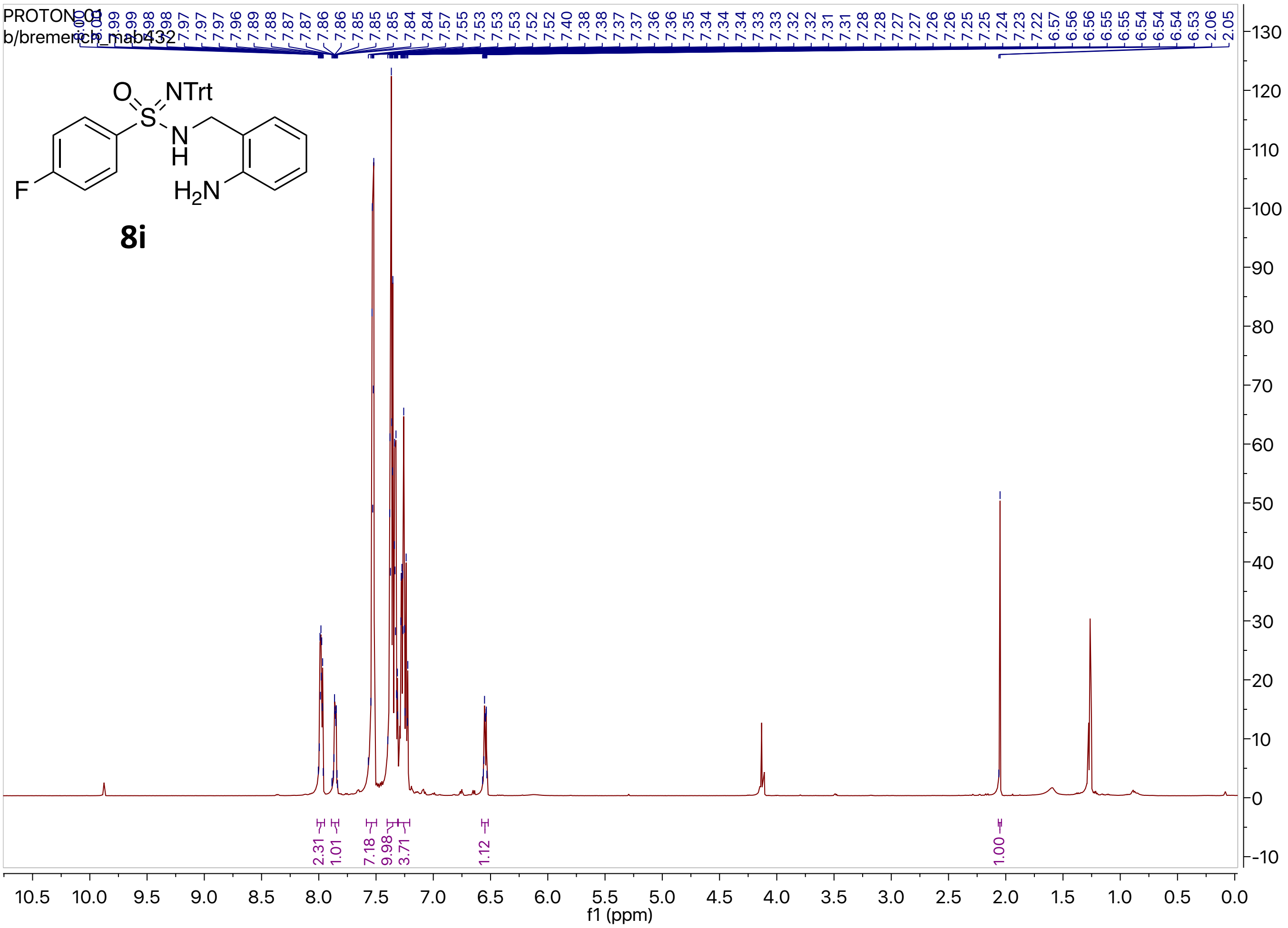
PROTON_01
b/bremerich_mab432
7.99 7.98 7.98 7.97 7.97 7.96 7.89 7.88 7.87 7.87 7.86 7.86 7.85 7.85 7.85 7.84 7.84 7.57 7.55 7.53 7.53 7.53 7.52 7.52 7.40 7.38 7.38 7.37 7.37 7.36 7.35 7.34 7.34 7.33 7.33 7.32 7.31 7.31 7.28 7.28 7.27 7.26 7.26 7.25 7.25 7.24 7.23 7.22 6.57 6.56 6.56 6.55 6.55 6.54 6.54 6.54 6.53 2.06 2.05
O
NTrt
S
N
H
F
H2N
8i
2.31 1.01 7.18 9.98 3.71 1.12 1.00
f1 (ppm)

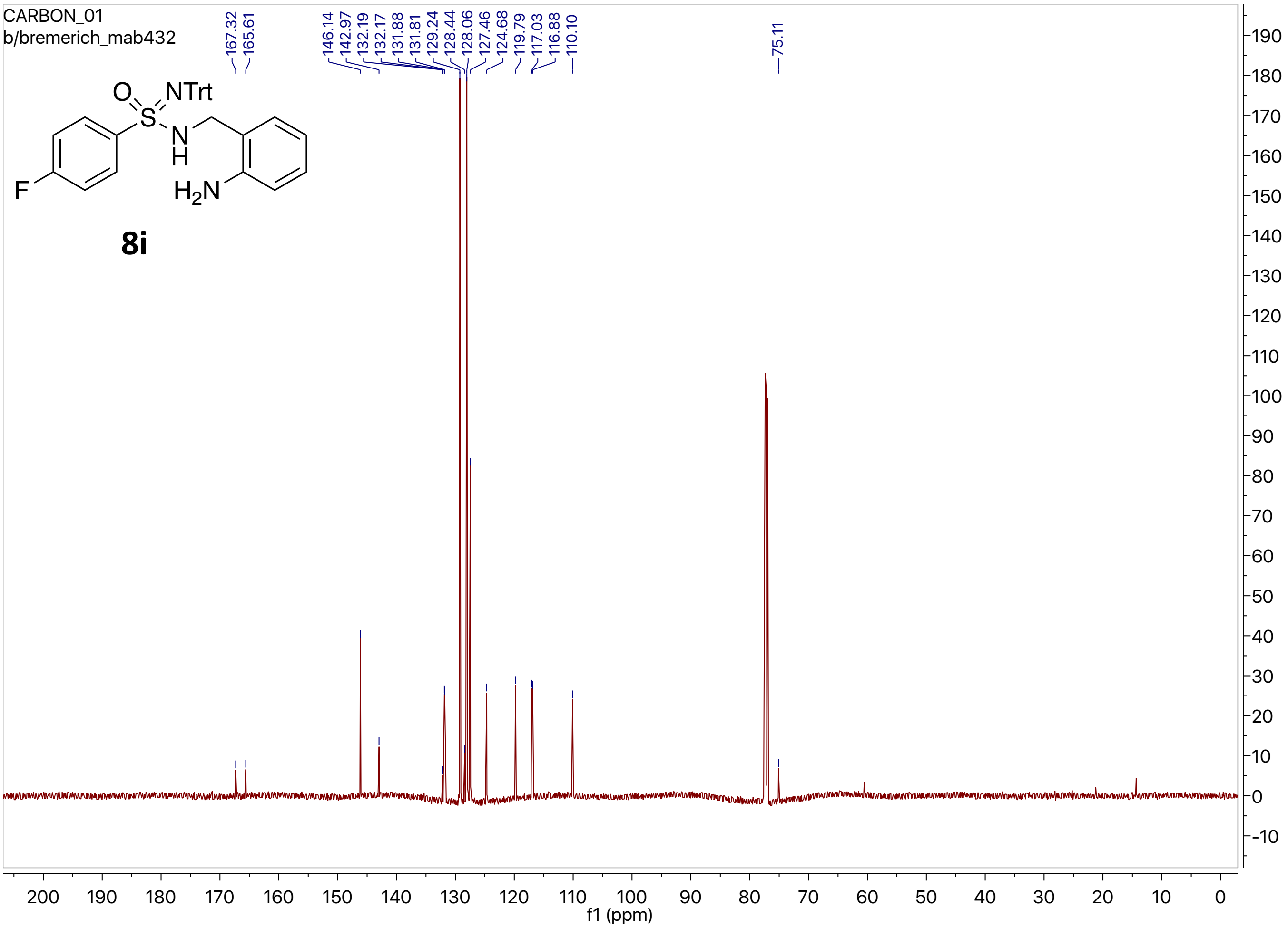
CARBON_01
b/bremerich_mab432
167.32 165.61 146.14 142.97 132.19 132.17 131.88 131.81 129.24 128.44 128.06 127.46 124.68 119.79 117.03 116.88 110.10 75.11
O
NTrt
S
N
H
F
H2N
8i
f1 (ppm)

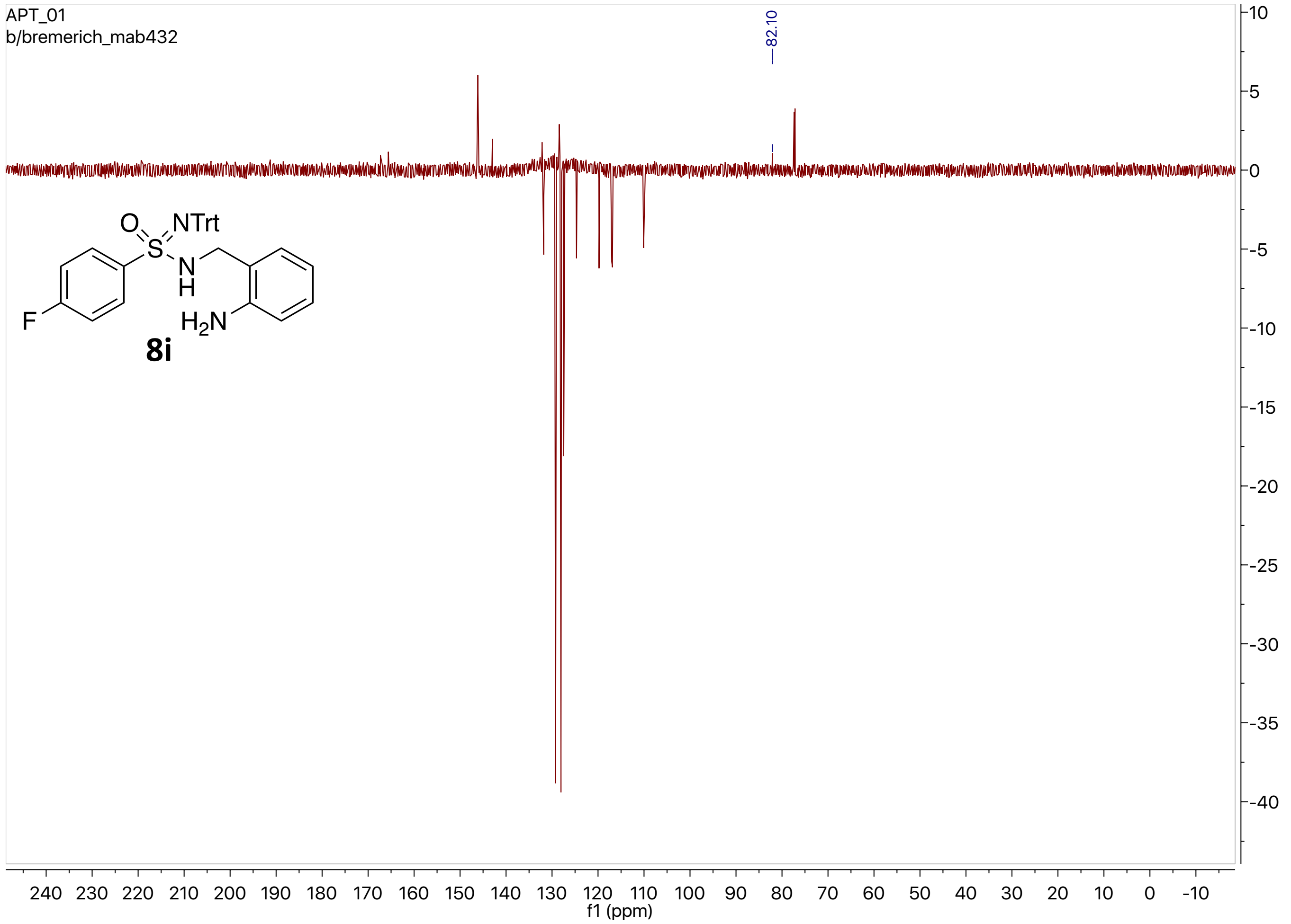
APT_01
b/bremerich_mab432
82.10
NTrt
O
S
N
H
F
H2N
8i
f1 (ppm)

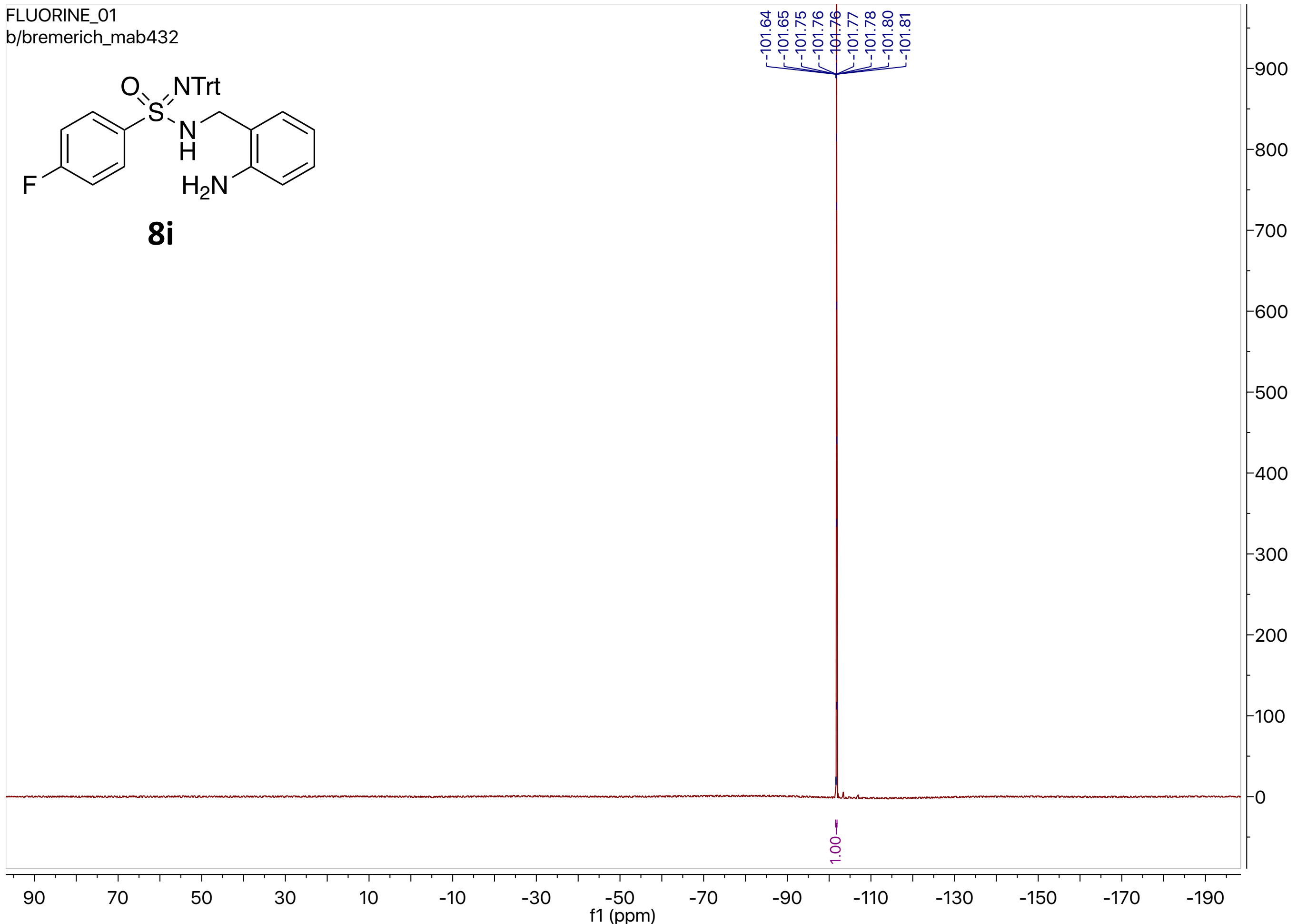
FLUORINE_01
b/bremerich_mab432
-101.64
-101.65
-101.75
-101.76
-101.76
-101.77
-101.78
-101.80
-101.81
NTrt
O
S
N
H
F
H2N
8i
1.00
f1 (ppm)

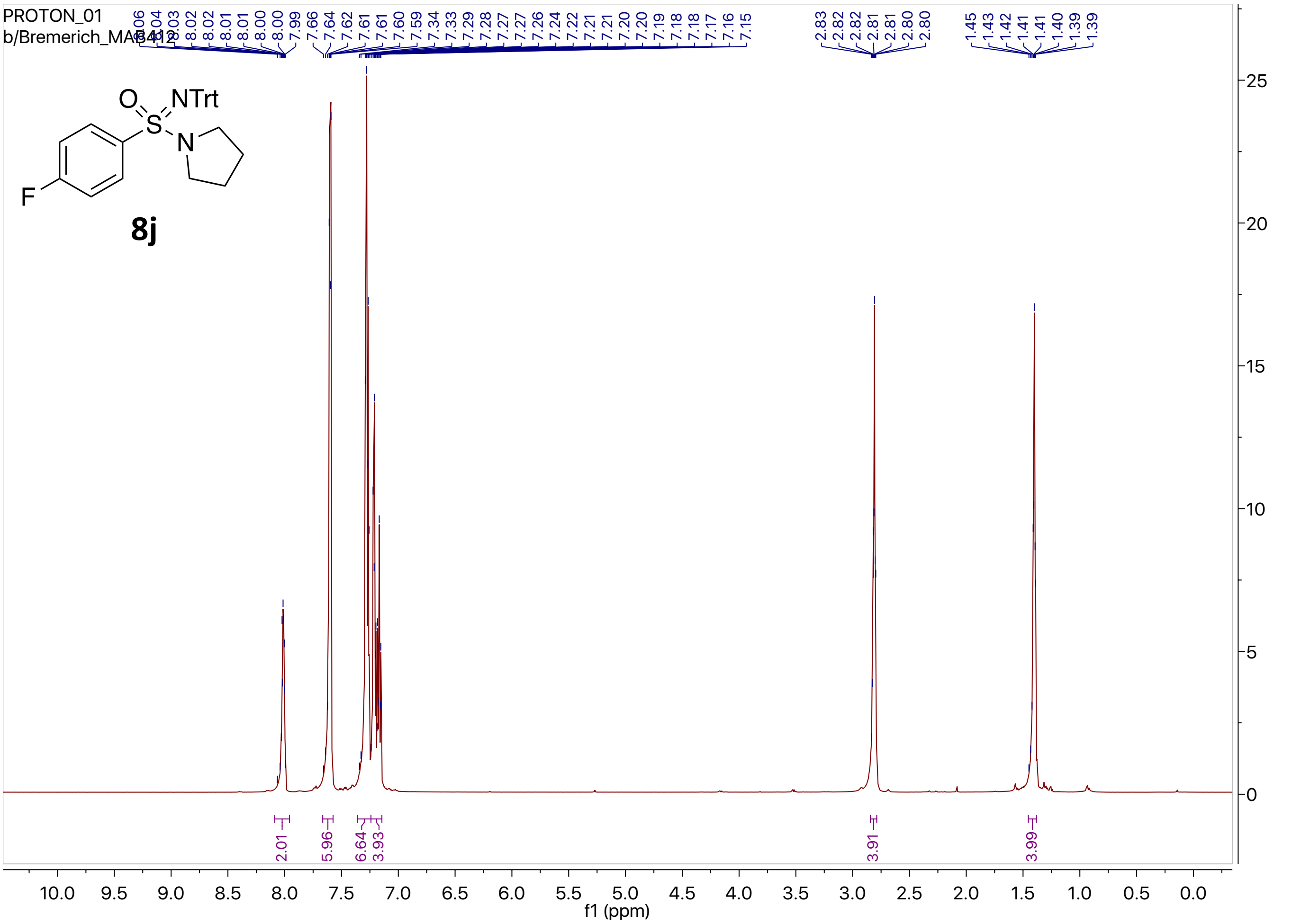
PROTON_01
b/Bremerich_MAB412
8.06 8.04 8.03 8.02 8.02 8.01 8.01 8.00 8.00 7.99 7.66 7.64 7.62 7.61 7.61 7.60 7.59 7.34 7.33 7.29 7.28 7.27 7.27 7.26 7.24 7.22 7.21 7.21 7.20 7.19 7.18 7.17 7.16 7.15
2.83 2.82 2.82 2.81 2.81 2.80 2.80
1.45 1.43 1.42 1.41 1.41 1.40 1.39 1.39
O
NTrt
S
N
F
8j
2.01
5.96
6.64
3.93
3.91
3.99
f1 (ppm)

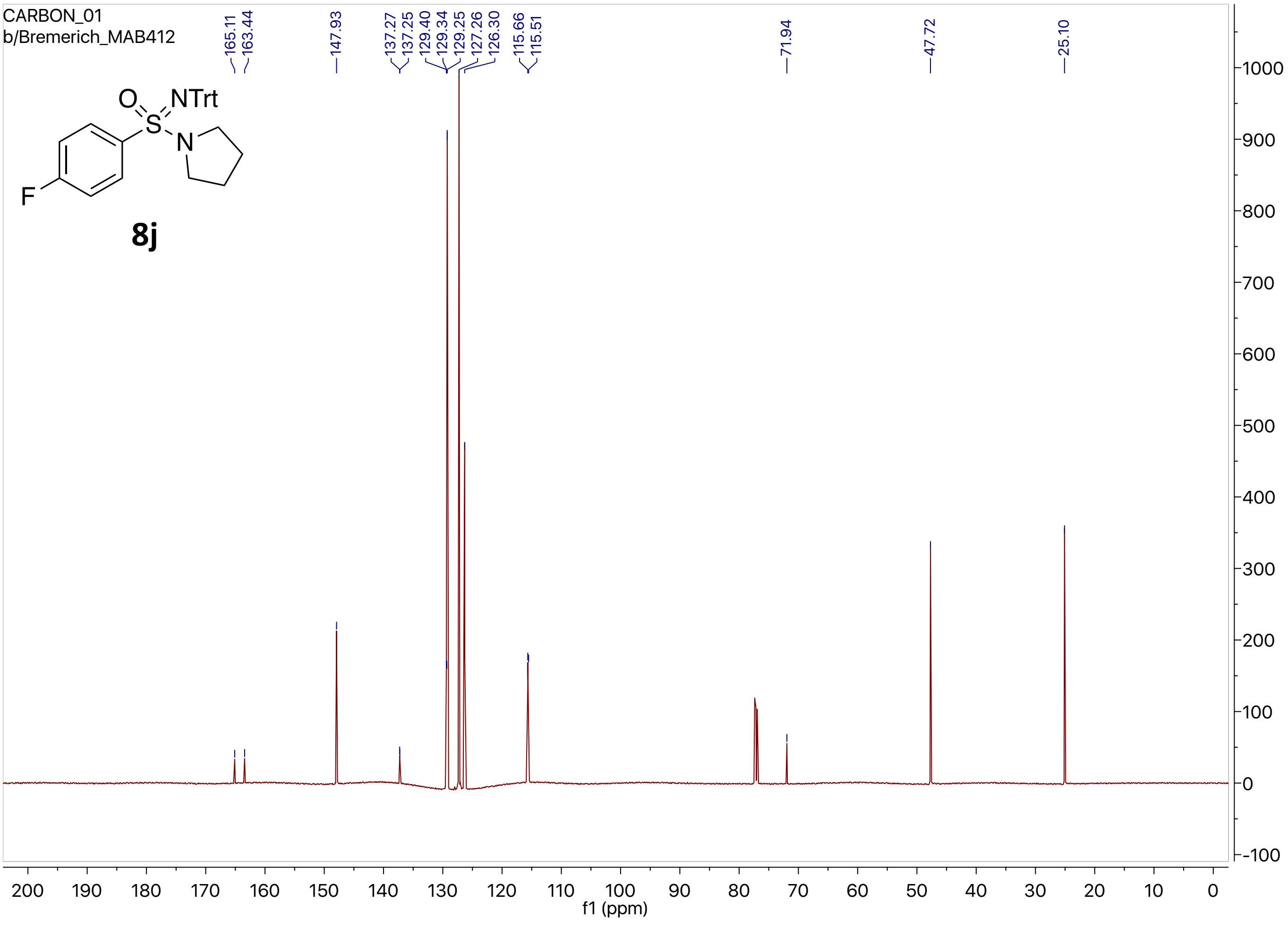
CARBON_01
b/Bremerich_MAB412
165.11 163.44
147.93
137.27 137.25 129.40 129.34 129.25 127.26 126.30
115.66 115.51
71.94
47.72
25.10
O
NTrt
S
N
F
8j
f1 (ppm)

FLUORINE_01
b/Bremerich_MAB412

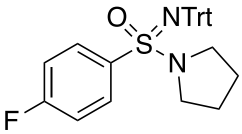

8j

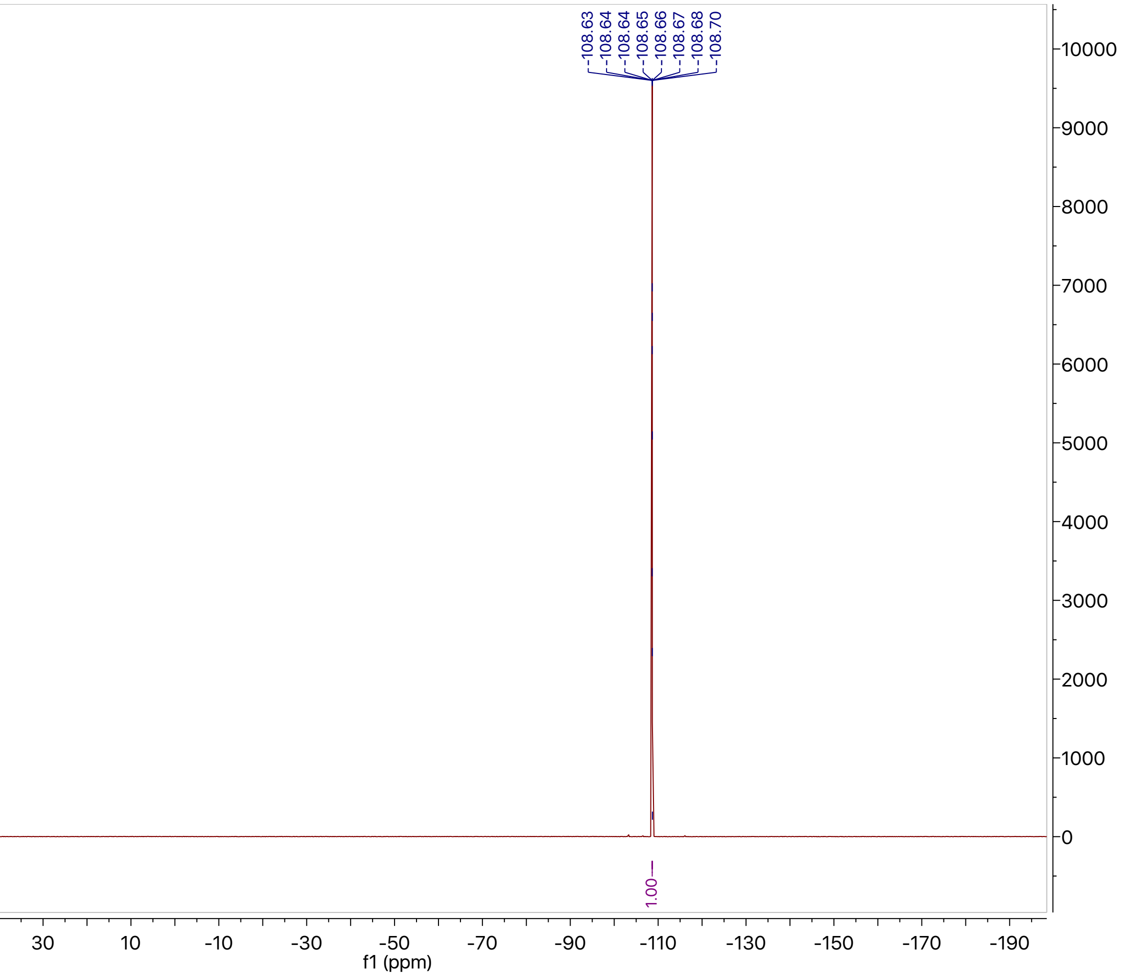

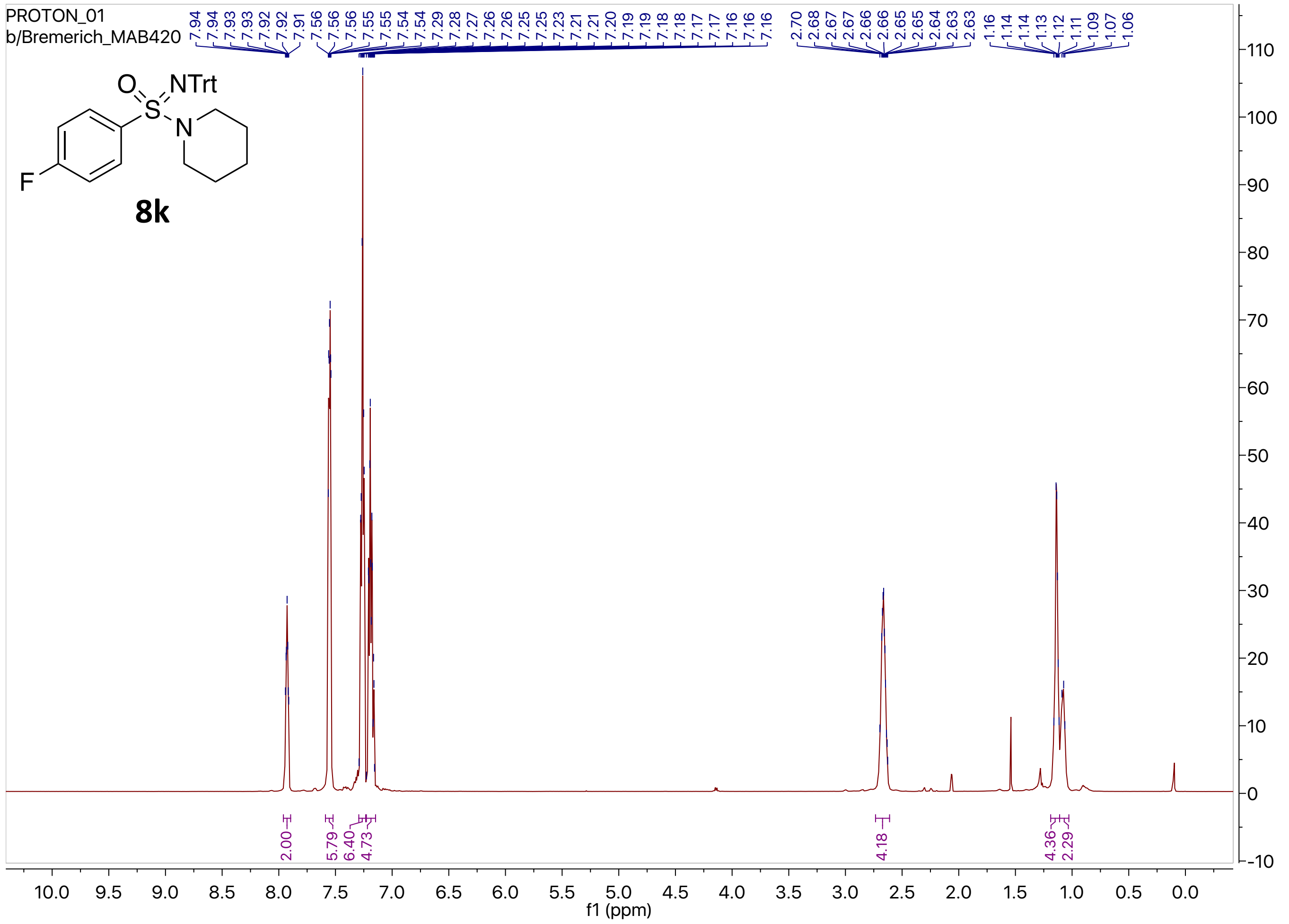

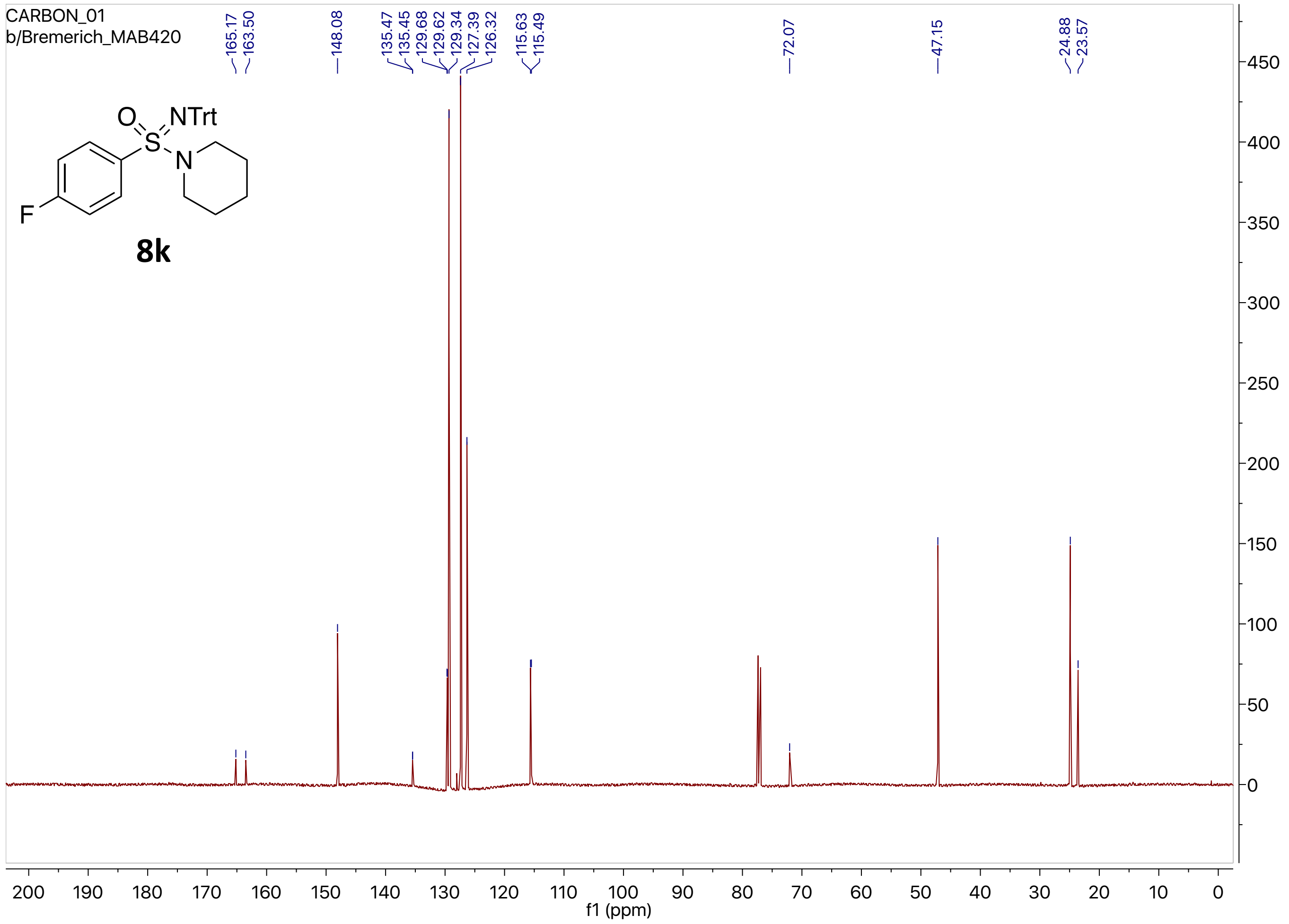

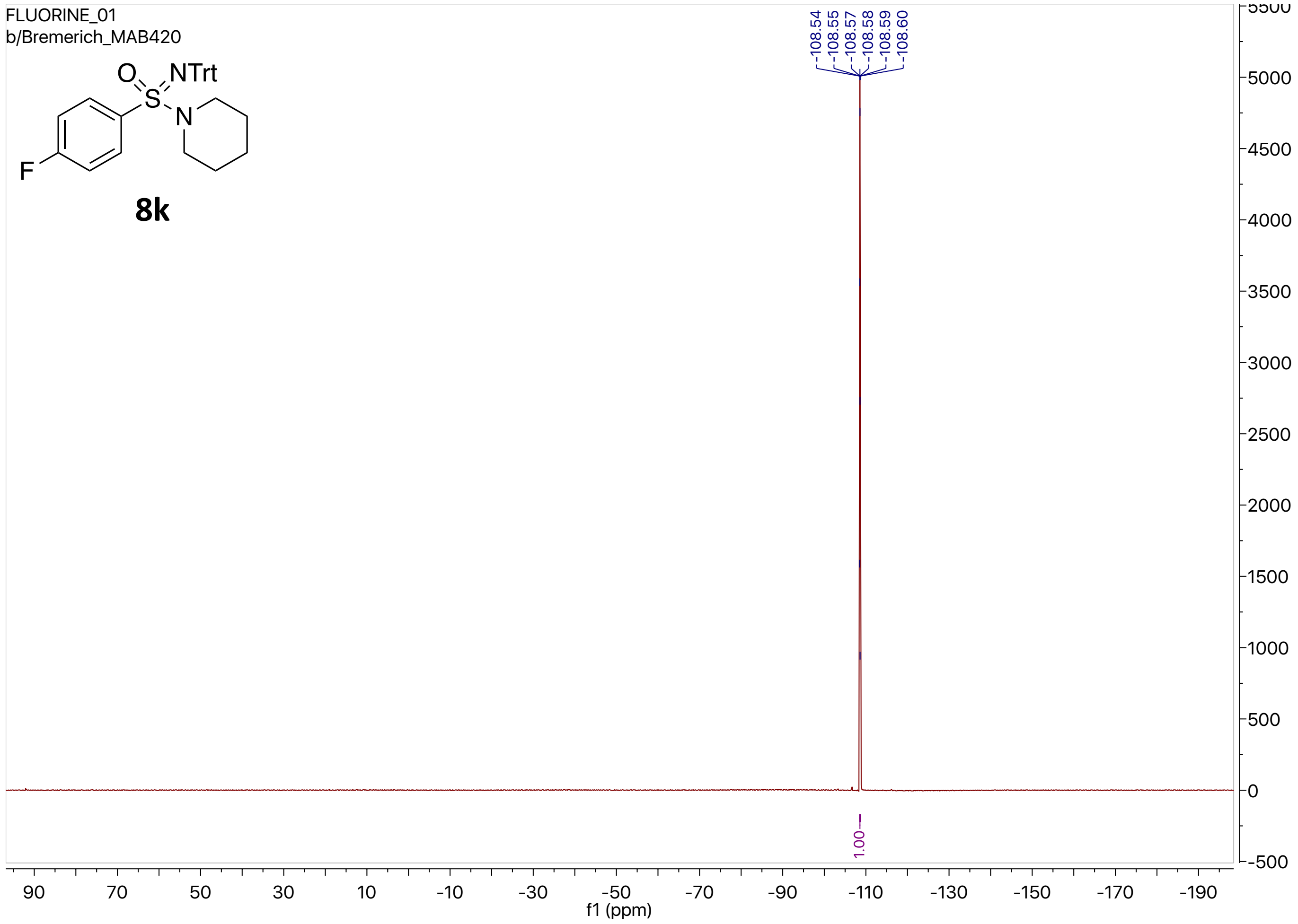
FLUORINE_01
b/Bremerich_MAB420
O
NTrt
S
N
F
8k
-108.54
-108.55
-108.57
-108.58
-108.59
-108.60
1.00
5500
5000
4500
4000
3500
3000
2500
2000
1500
1000
500
0
-500
90
70
50
30
10
-10
-30
-50
-70
-90
-110
-130
-150
-170
-190
f1 (ppm)

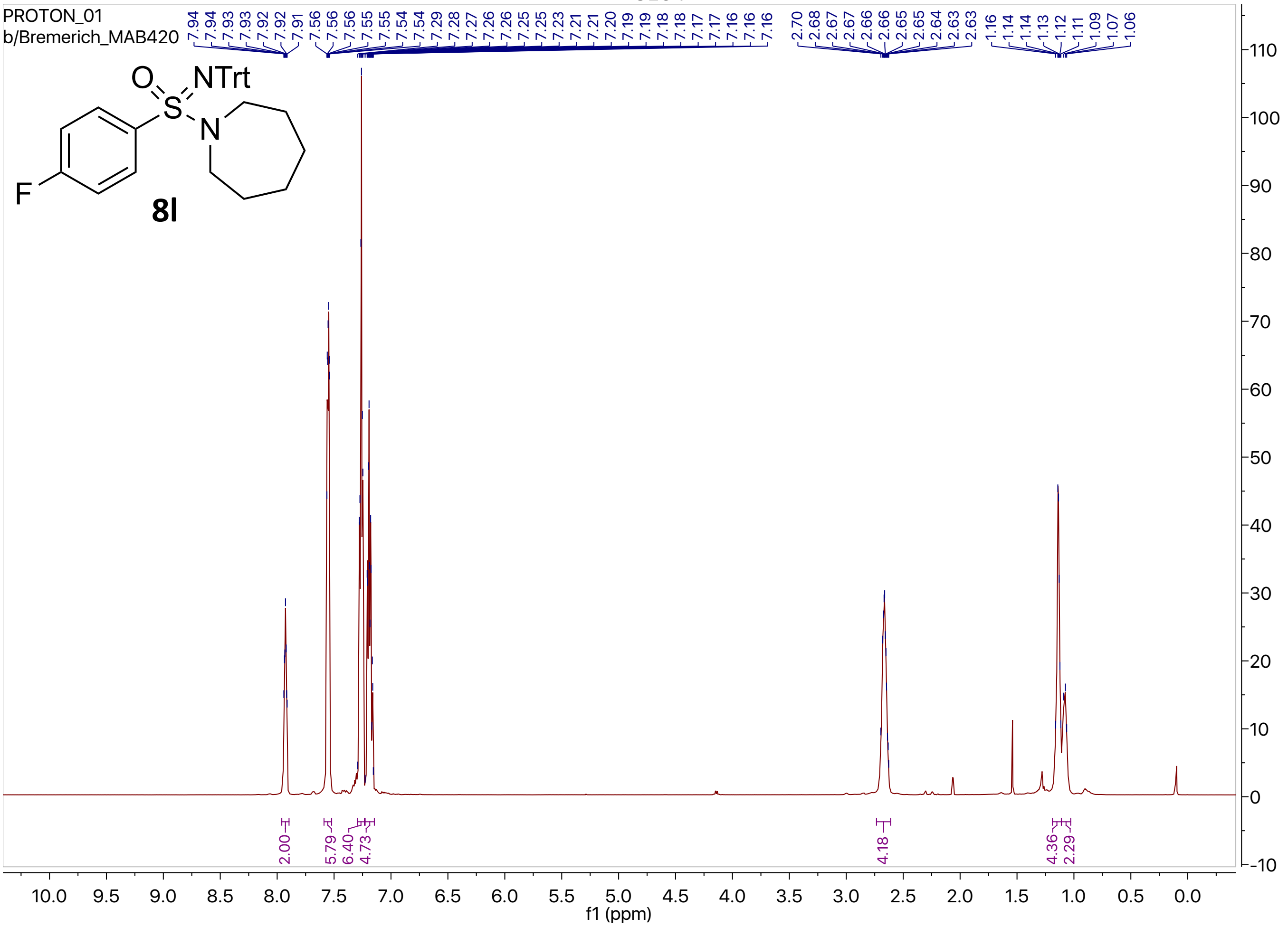
PROTON_01
b/Bremerich_MAB420
7.94 7.94 7.93 7.93 7.92 7.92 7.91 7.56 7.56 7.56 7.55 7.55 7.54 7.54 7.29 7.28 7.27 7.26 7.26 7.25 7.25 7.23 7.21 7.21 7.20 7.19 7.19 7.18 7.18 7.17 7.17 7.16 7.16 7.16
2.70 2.68 2.67 2.67 2.66 2.66 2.65 2.65 2.64 2.63 2.63
1.16 1.14 1.13 1.12 1.11 1.09 1.07 1.06
NTrt
O
S
N
F
8l
2.00 5.79 6.40 4.73 4.18 4.36 2.29
f1 (ppm)

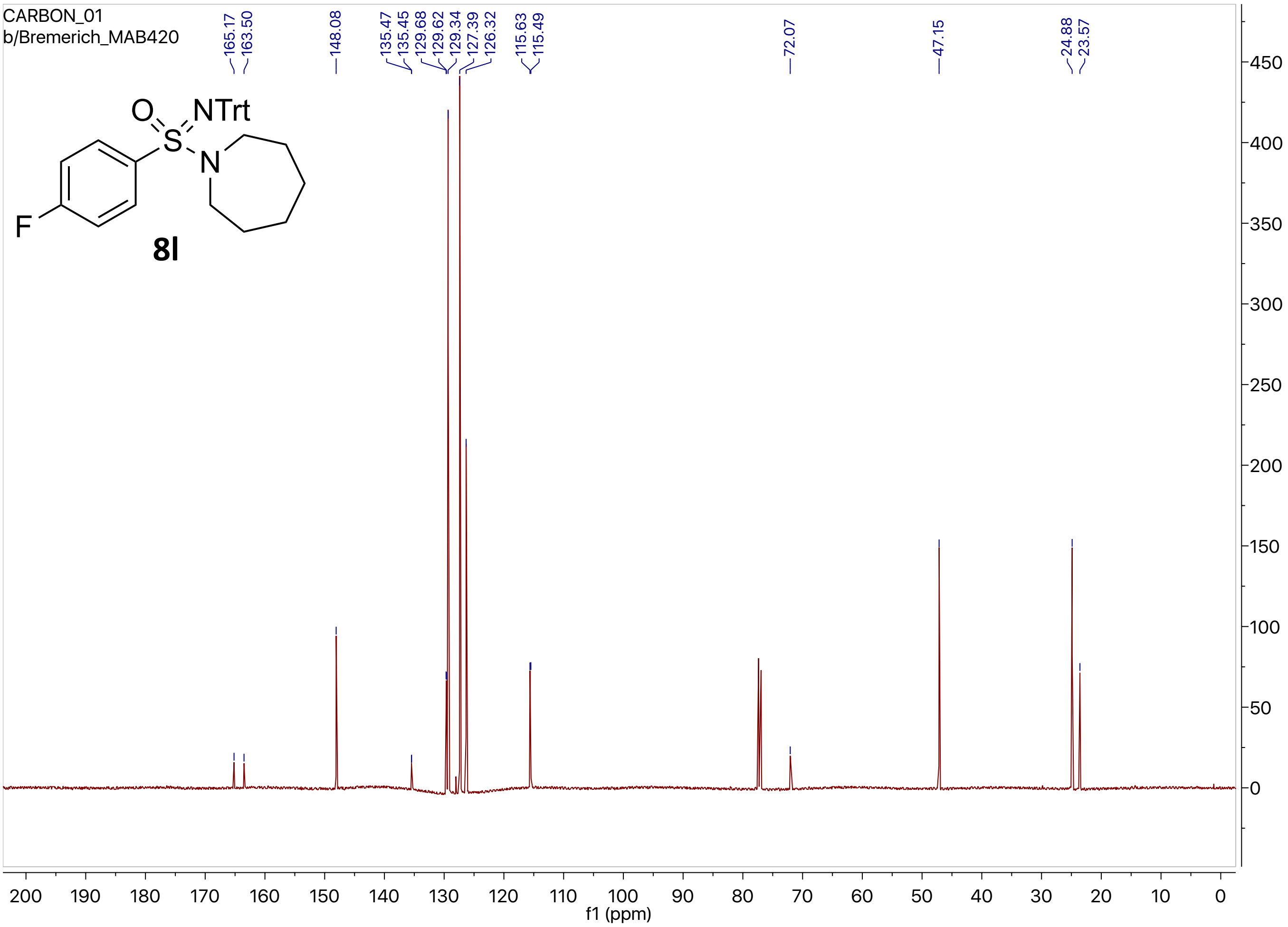
CARBON_01
b/Bremerich_MAB420
165.17 163.50 148.08 135.47 135.45 129.68 129.62 129.34 127.39 126.32 115.63 115.49 72.07 47.15 24.88 23.57
NTrt
O
S
N
F
8l
f1 (ppm)

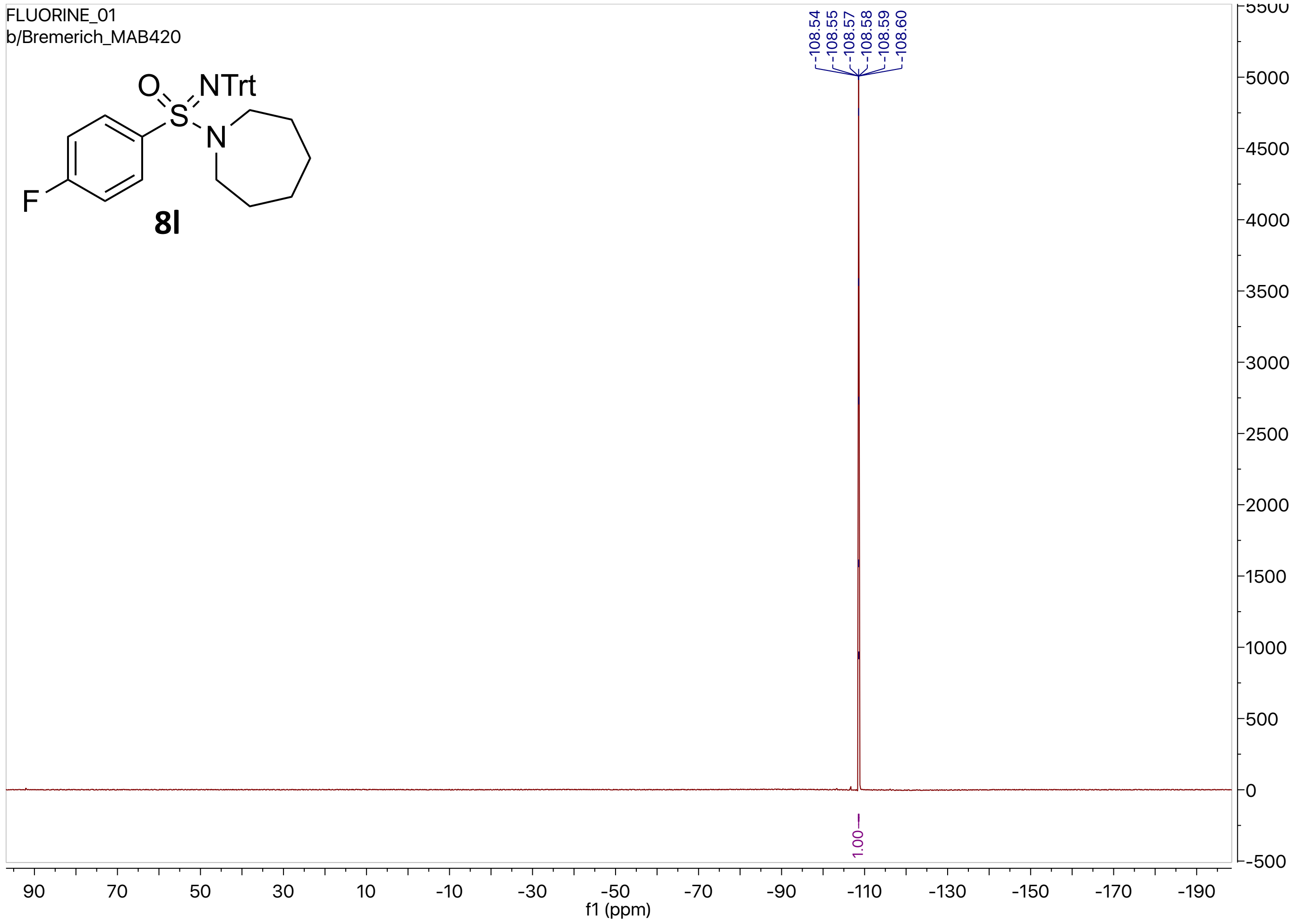
FLUORINE_01
b/Bremerich_MAB420
O
NTrt
S
N
F
8l
-108.54
-108.55
-108.57
-108.58
-108.59
-108.60
1.00
f1 (ppm)

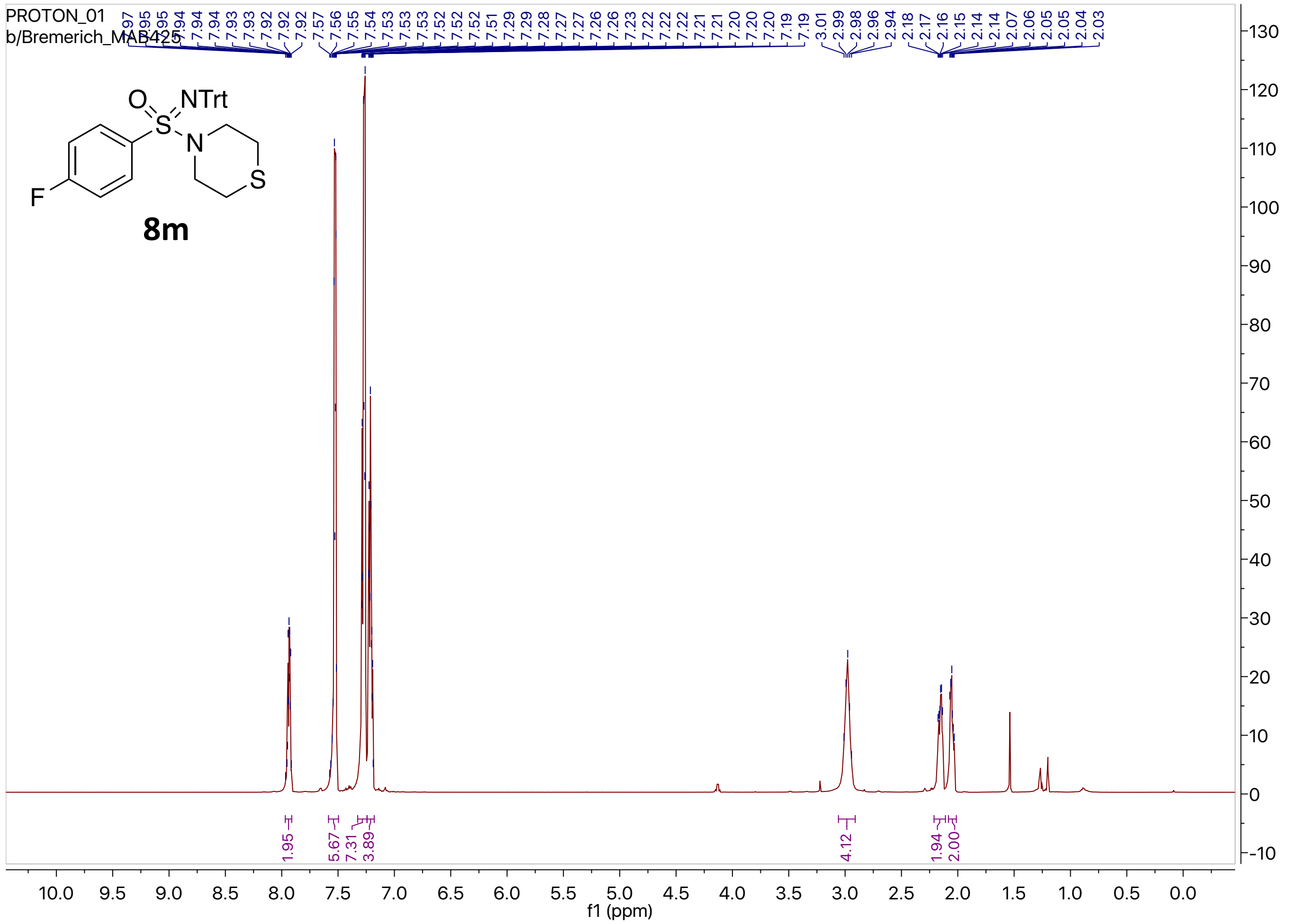
PROTON_01
b/Bremerich_MAB425
NTrt
8m
f1 (ppm)

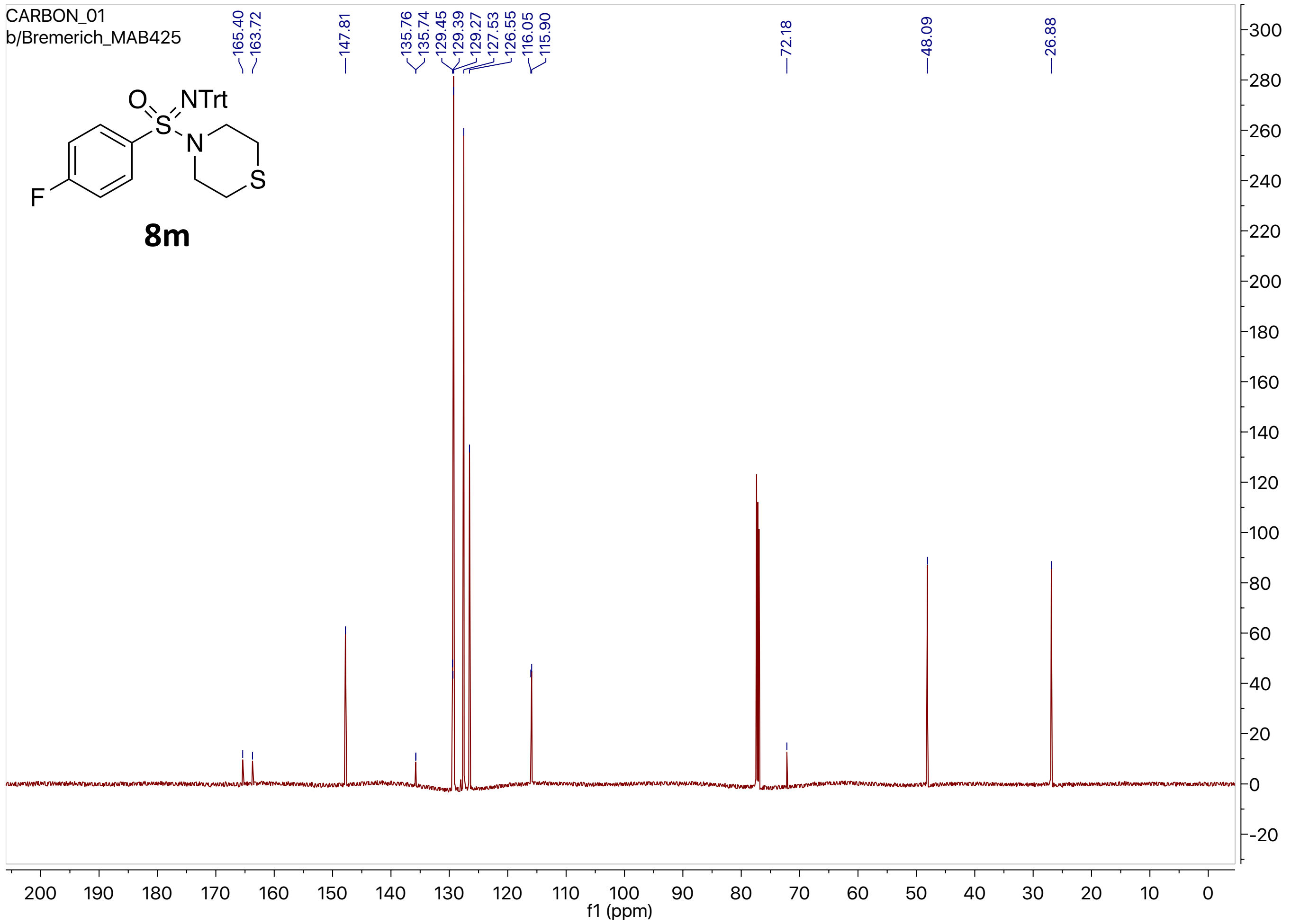
CARBON_01
b/Bremerich_MAB425
165.40
163.72
147.81
135.76
135.74
129.45
129.39
129.27
127.53
126.55
116.05
115.90
72.18
48.09
26.88
NTrt
8m
f1 (ppm)

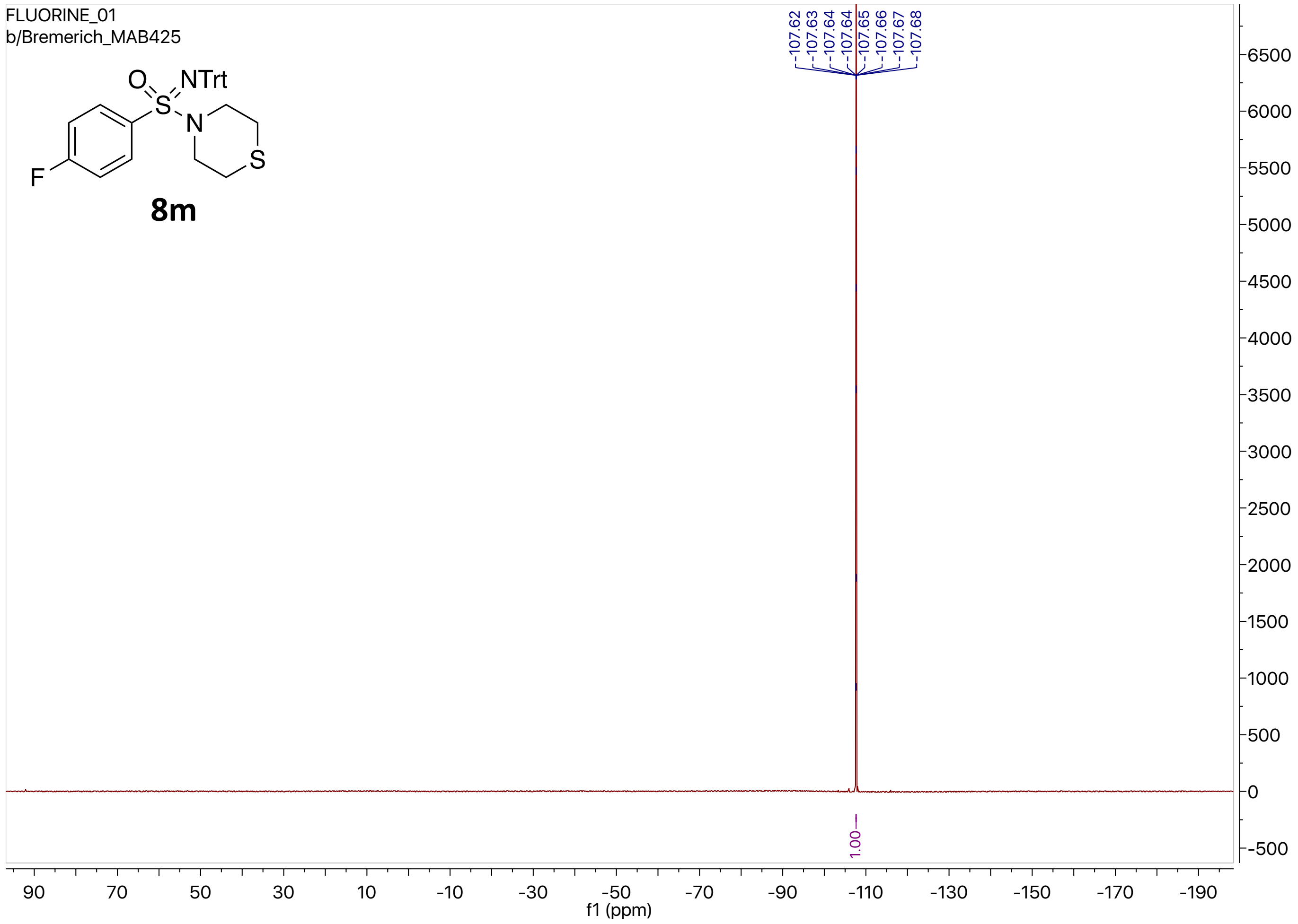
FLUORINE_01
b/Bremerich_MAB425
O
NTrt
S
N
S
F
8m
-107.62
-107.63
-107.64
-107.64
-107.65
-107.66
-107.67
-107.68
1.00
f1 (ppm)

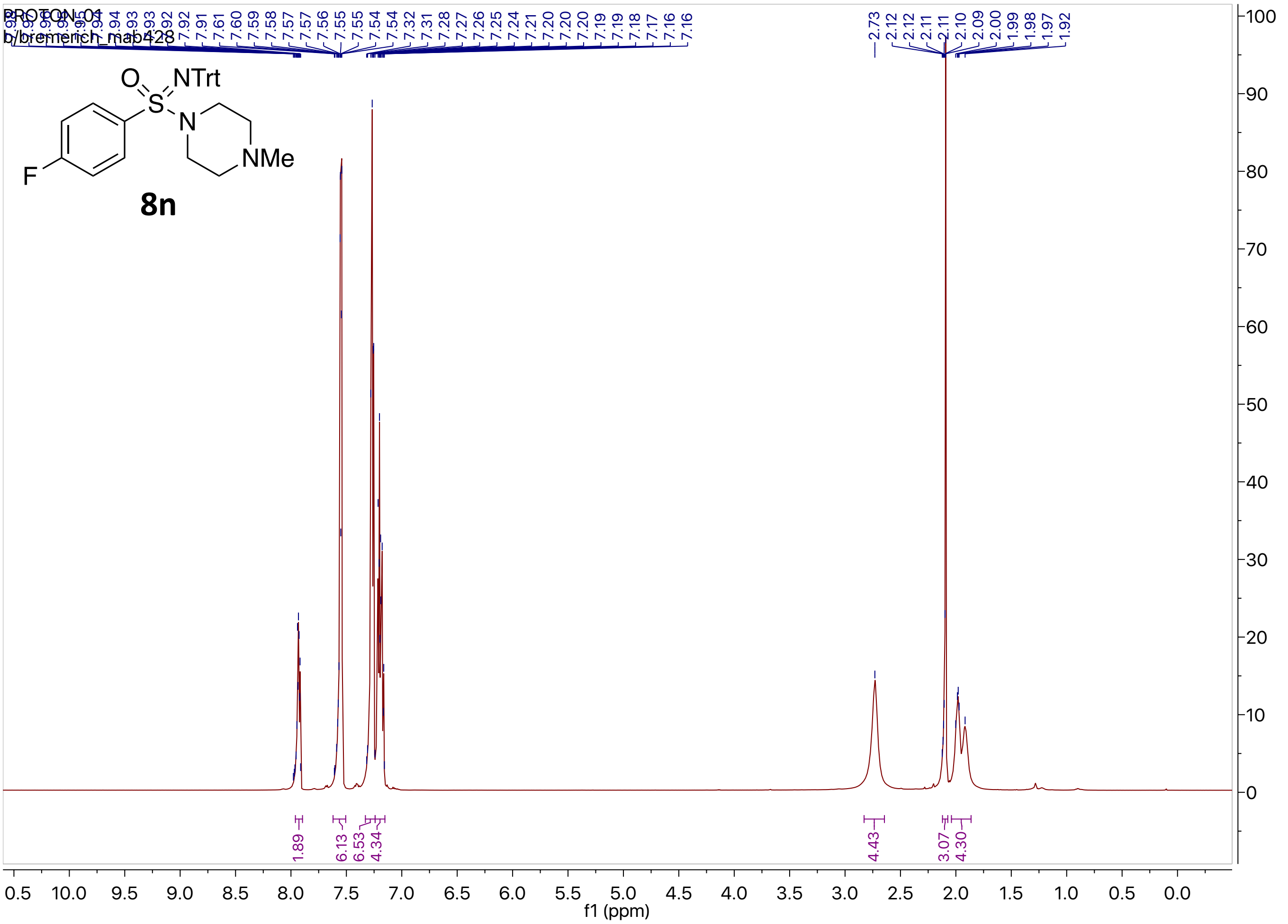
PROTON_01
b/bremerich_mab428
7.94
7.93
7.93
7.92
7.92
7.91
7.61
7.60
7.59
7.58
7.57
7.57
7.56
7.55
7.55
7.54
7.54
7.32
7.31
7.28
7.27
7.26
7.25
7.24
7.21
7.20
7.20
7.19
7.19
7.18
7.17
7.16
7.16
2.73
2.12
2.12
2.11
2.11
2.10
2.09
2.00
1.99
1.98
1.97
1.92
O
S
NTrt
N
NMe
F
8n
1.89
6.13
6.53
4.34
4.43
3.07
4.30
f1 (ppm)

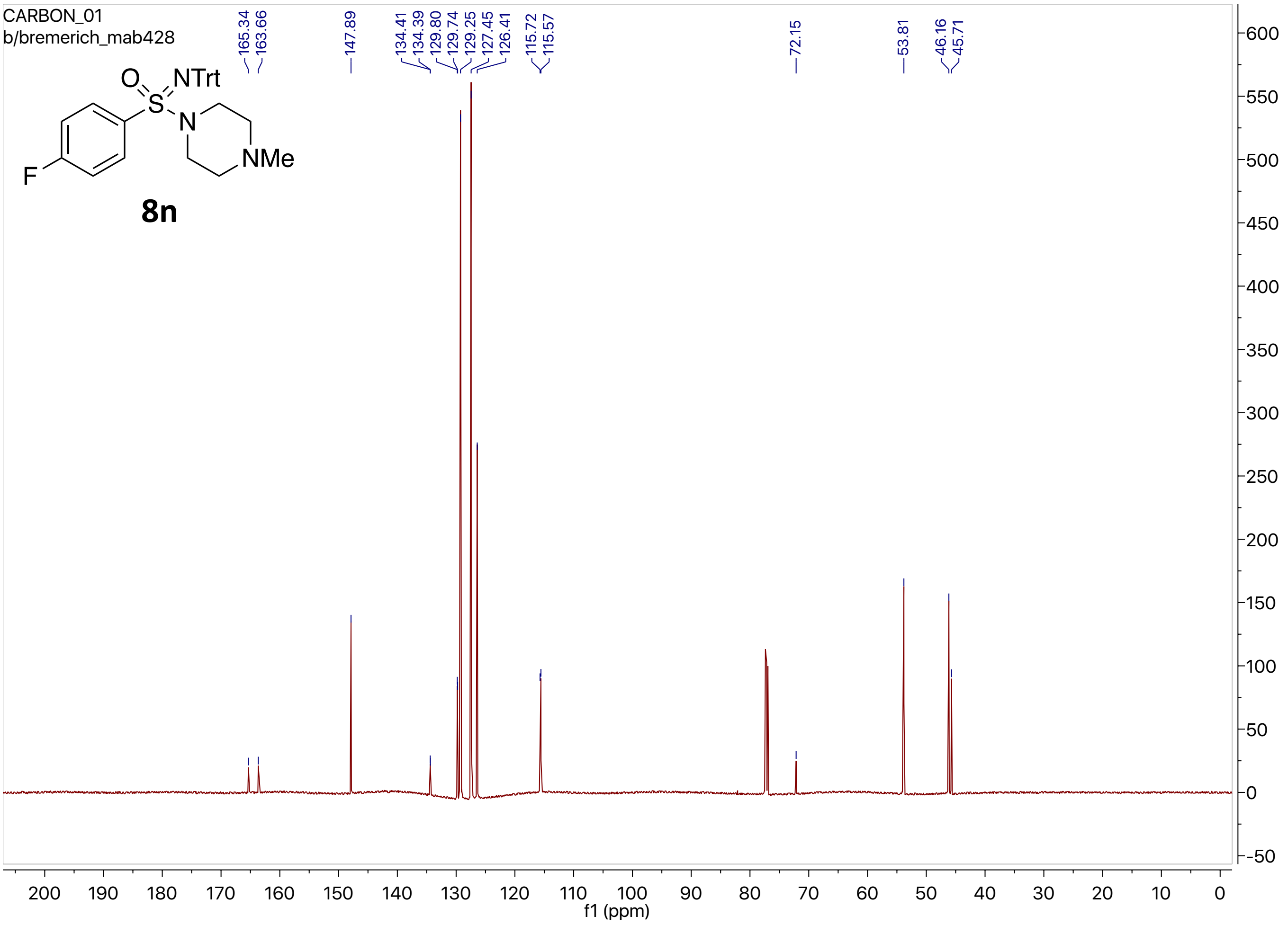
CARBON_01
b/bremerich_mab428
165.34
163.66
147.89
134.41
134.39
129.80
129.74
129.25
127.45
126.41
115.72
115.57
72.15
53.81
46.16
45.71
O
S
NTrt
N
NMe
F
8n
f1 (ppm)

FLUORINE_01
b/bremerich_mab428

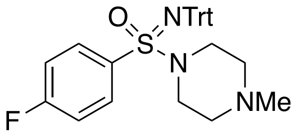

8n

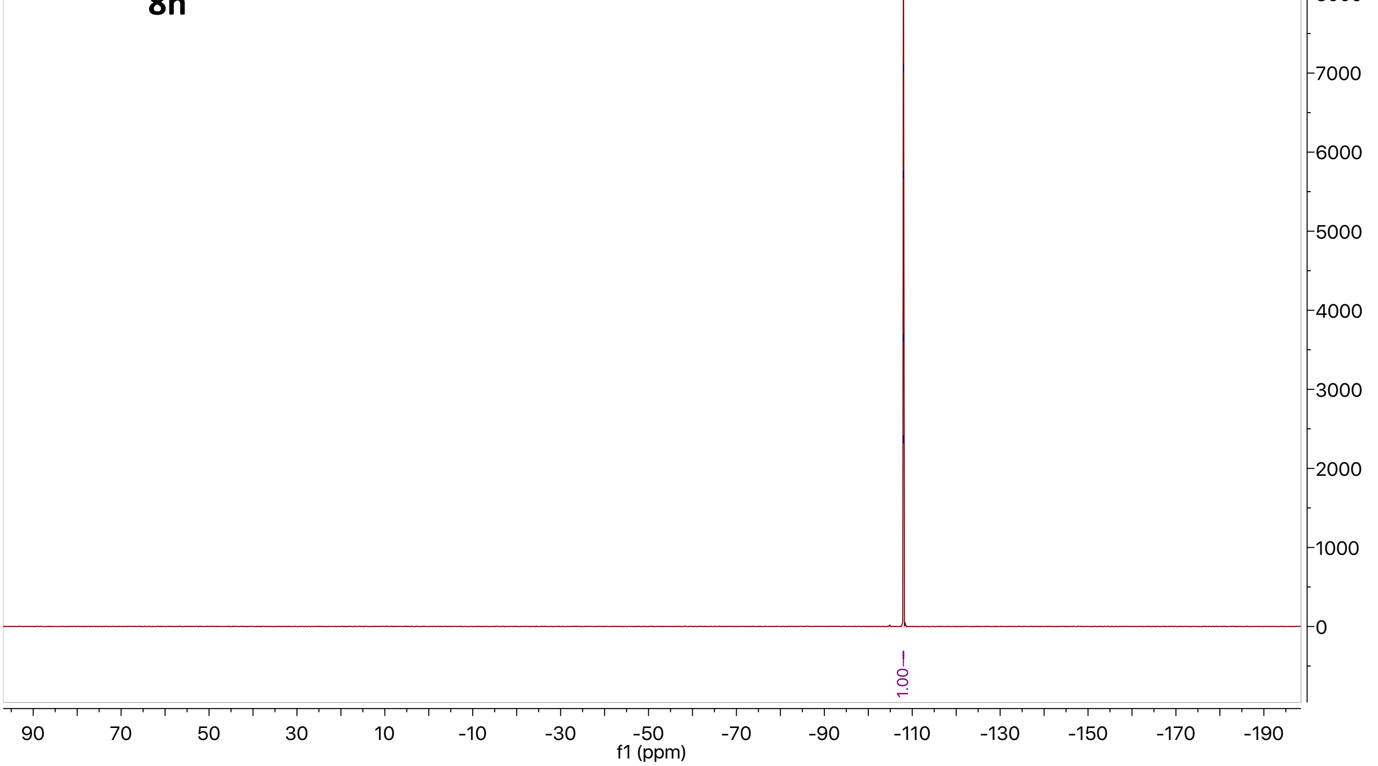

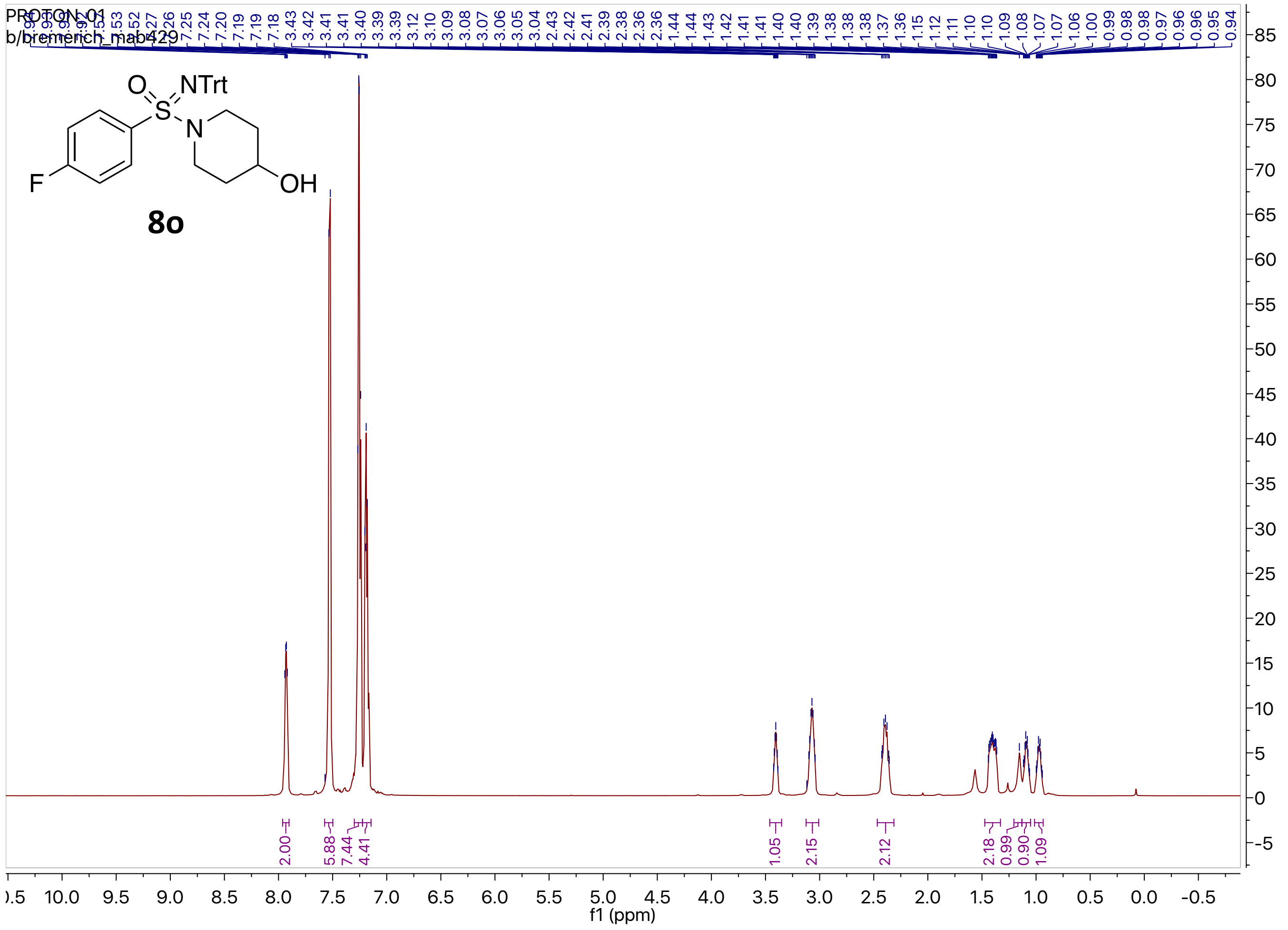

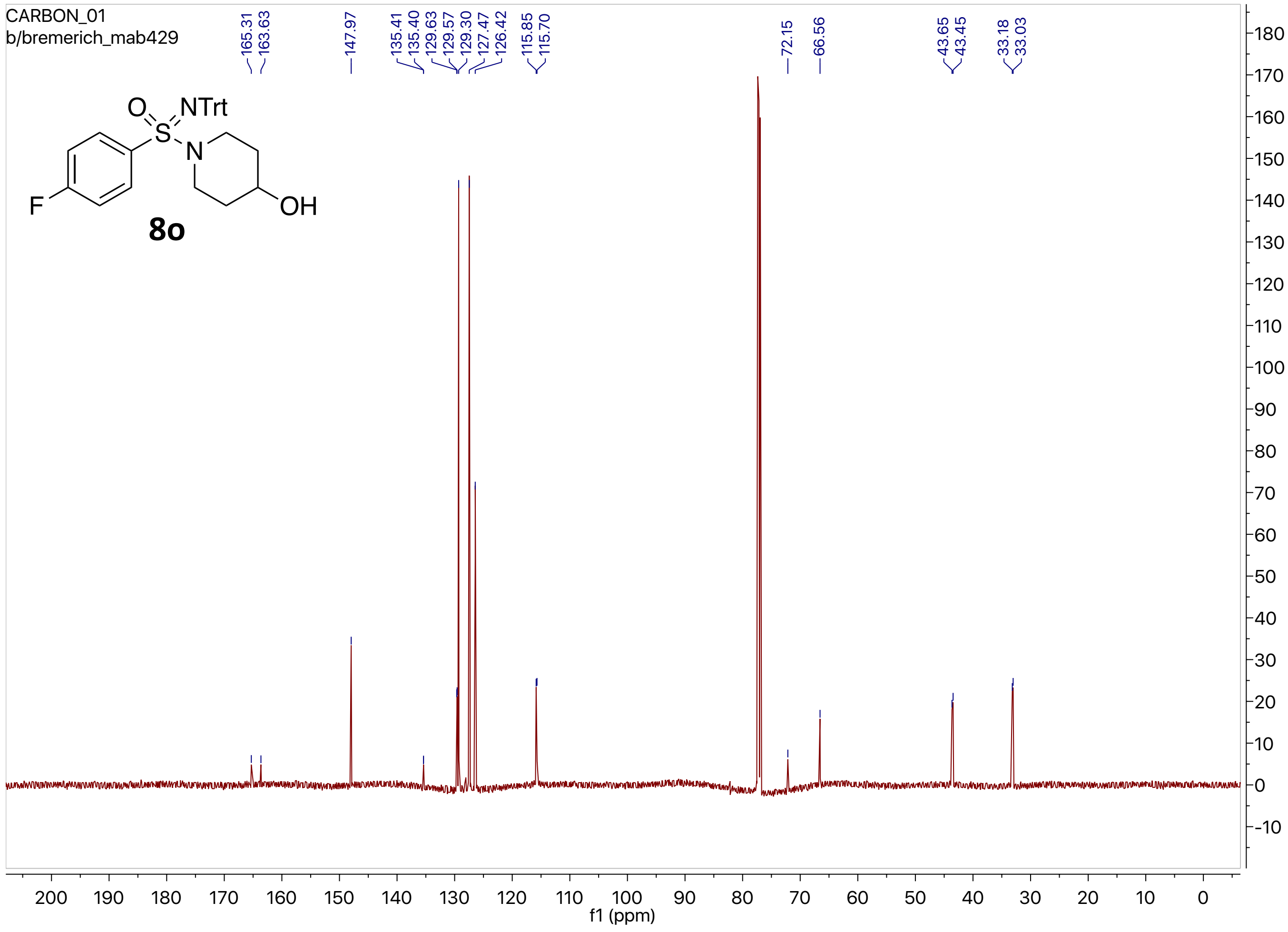

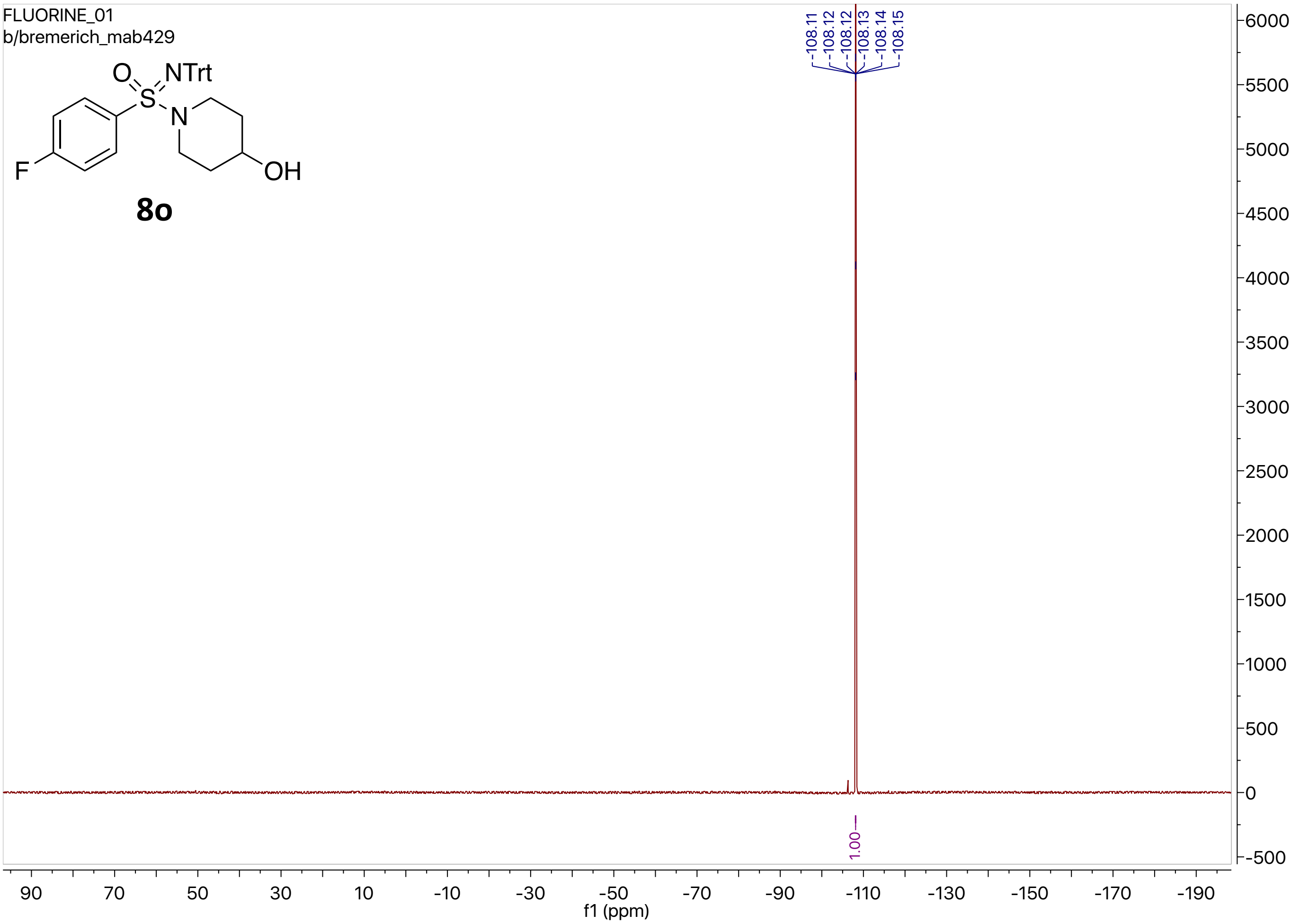
FLUORINE_01
b/bremerich_mab429
NTrt
O
S
N
F
OH
8o
-108.11
-108.12
-108.12
-108.13
-108.14
-108.15
1.00
6000
5500
5000
4500
4000
3500
3000
2500
2000
1500
1000
500
0
-500
90
70
50
30
10
-10
-30
-50
-70
-90
-110
-130
-150
-170
-190
f1 (ppm)

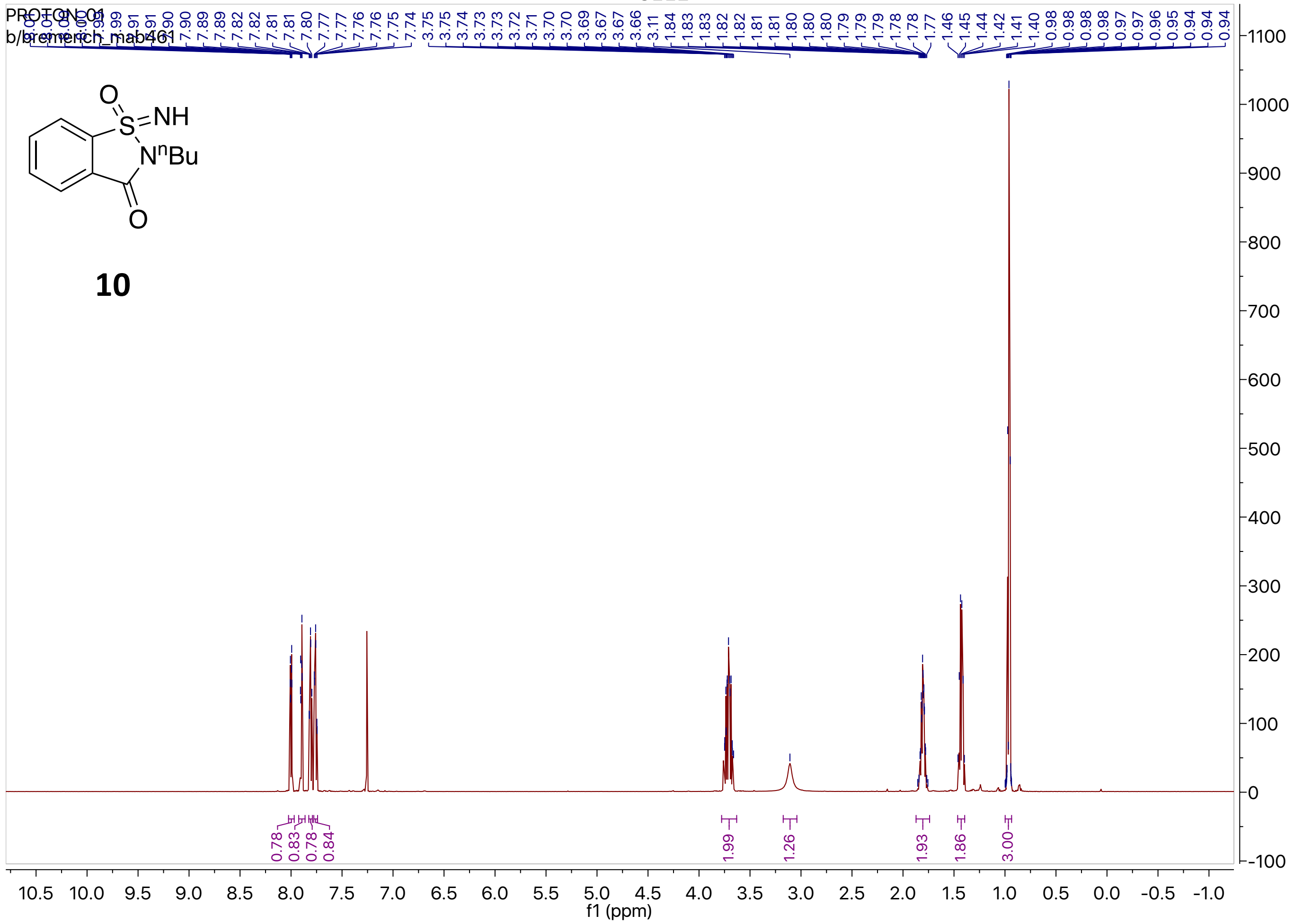
PROTON_01
b/bremerich_mab461
10
0.78
0.83
0.78
0.84
1.99
1.26
1.93
1.86
3.00
f1 (ppm)

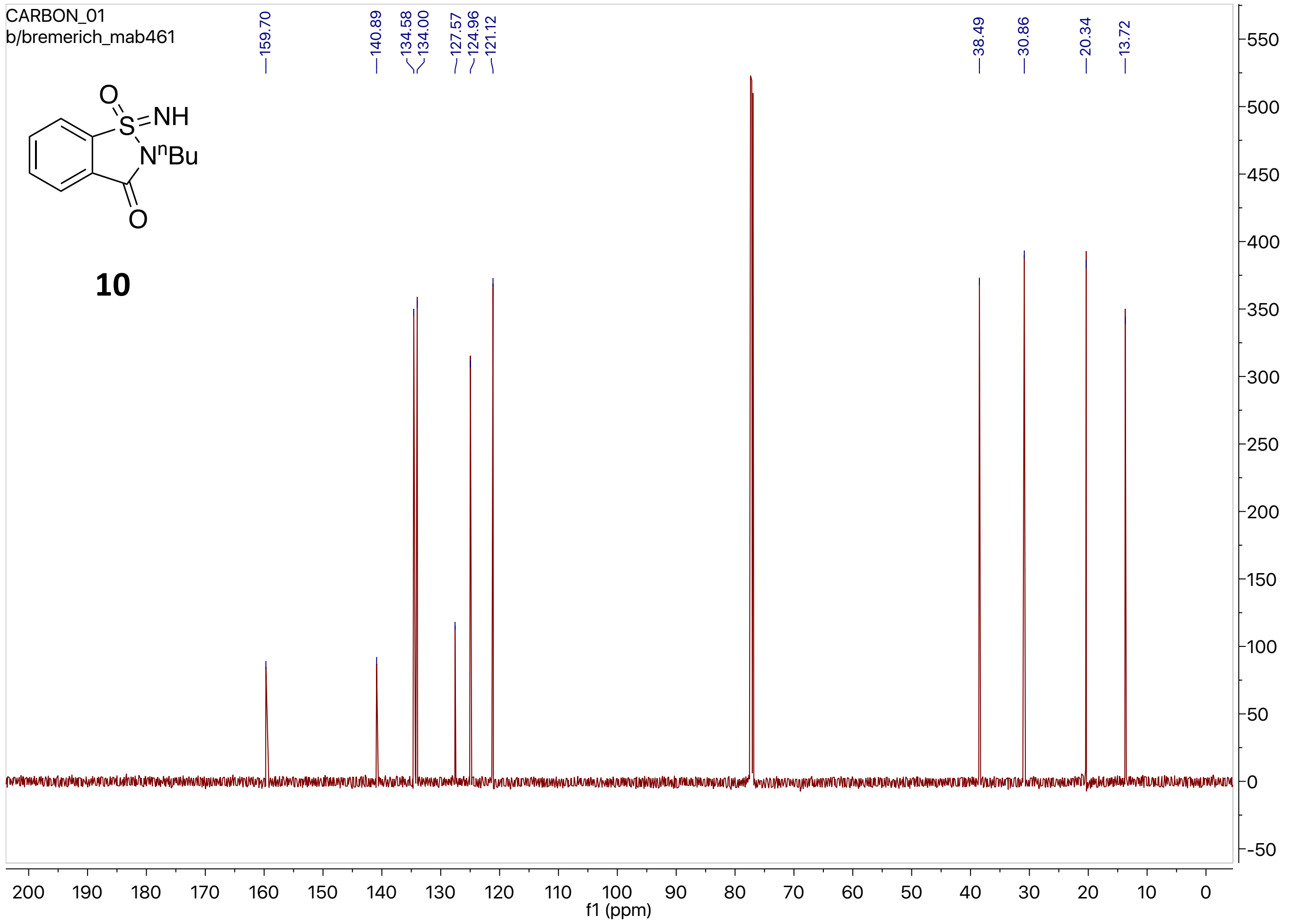
CARBON_01
b/bremerich_mab461
159.70
140.89
134.58
134.00
127.57
124.96
121.12
38.49
30.86
20.34
13.72
10
f1 (ppm)

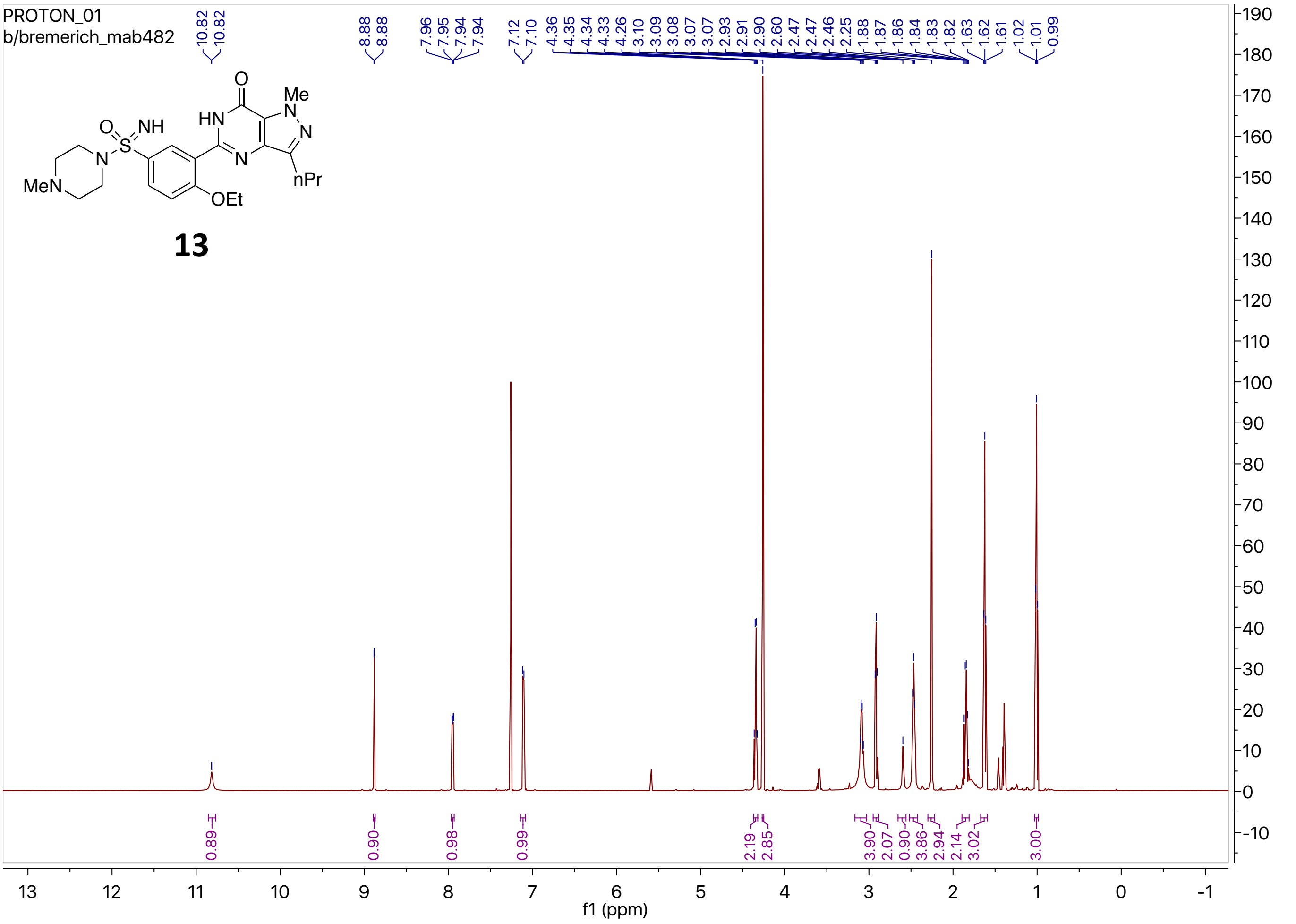
PROTON_01
b/bremerich_mab482
13
f1 (ppm)

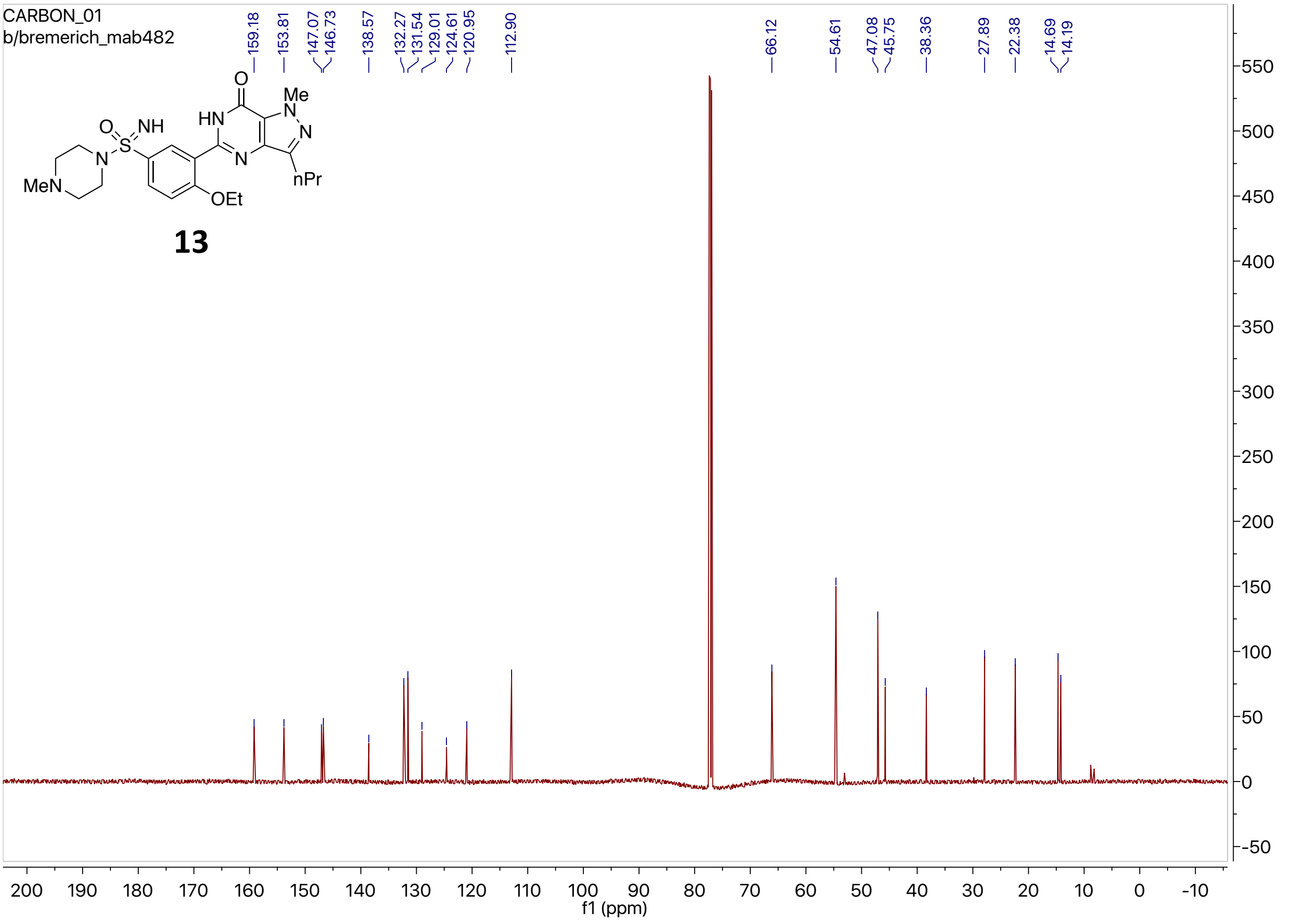
CARBON_01
b/bremerich_mab482
13
f1 (ppm)

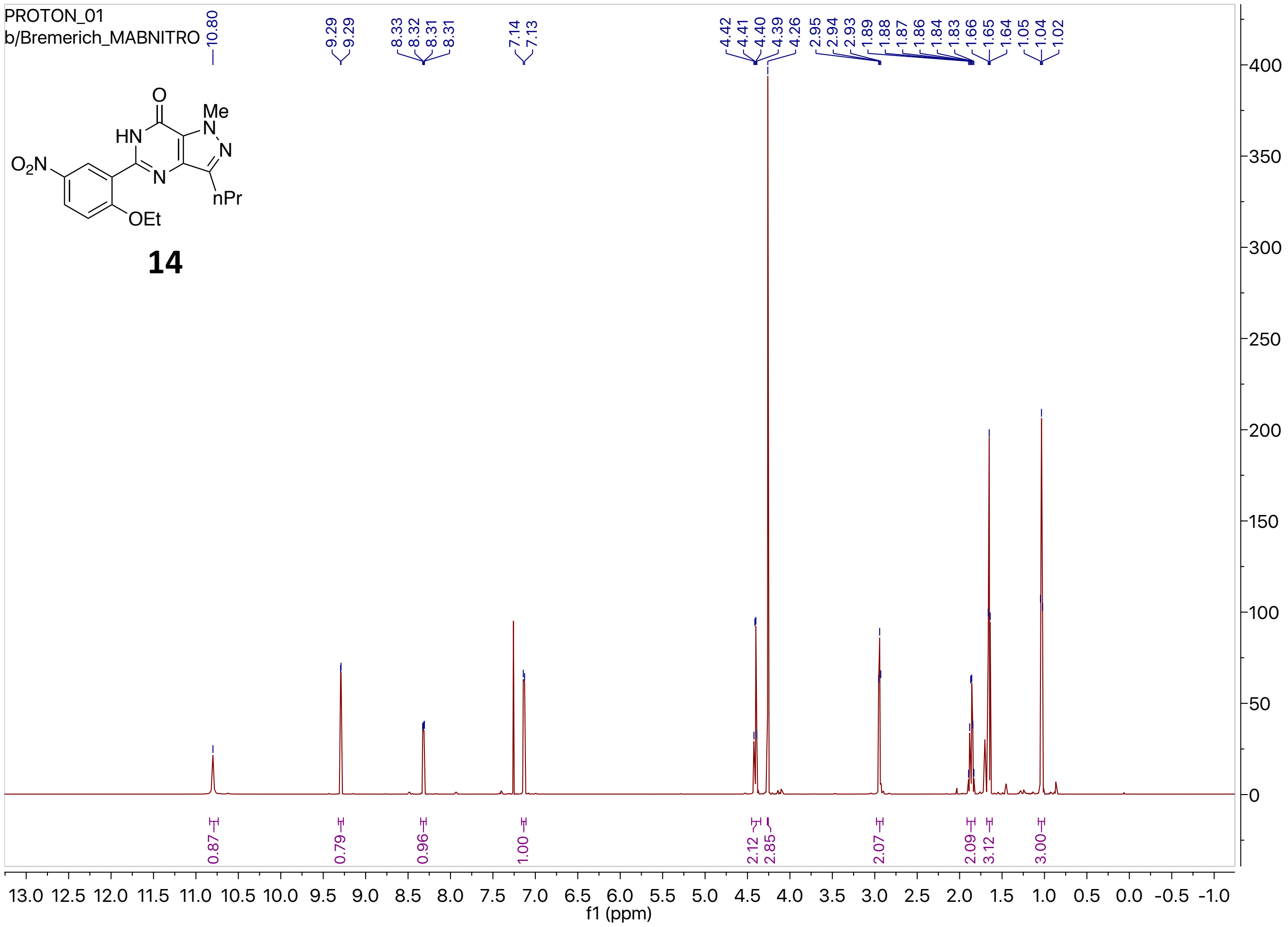
PROTON_01
b/Bremerich_MABNITRO
14
f1 (ppm)

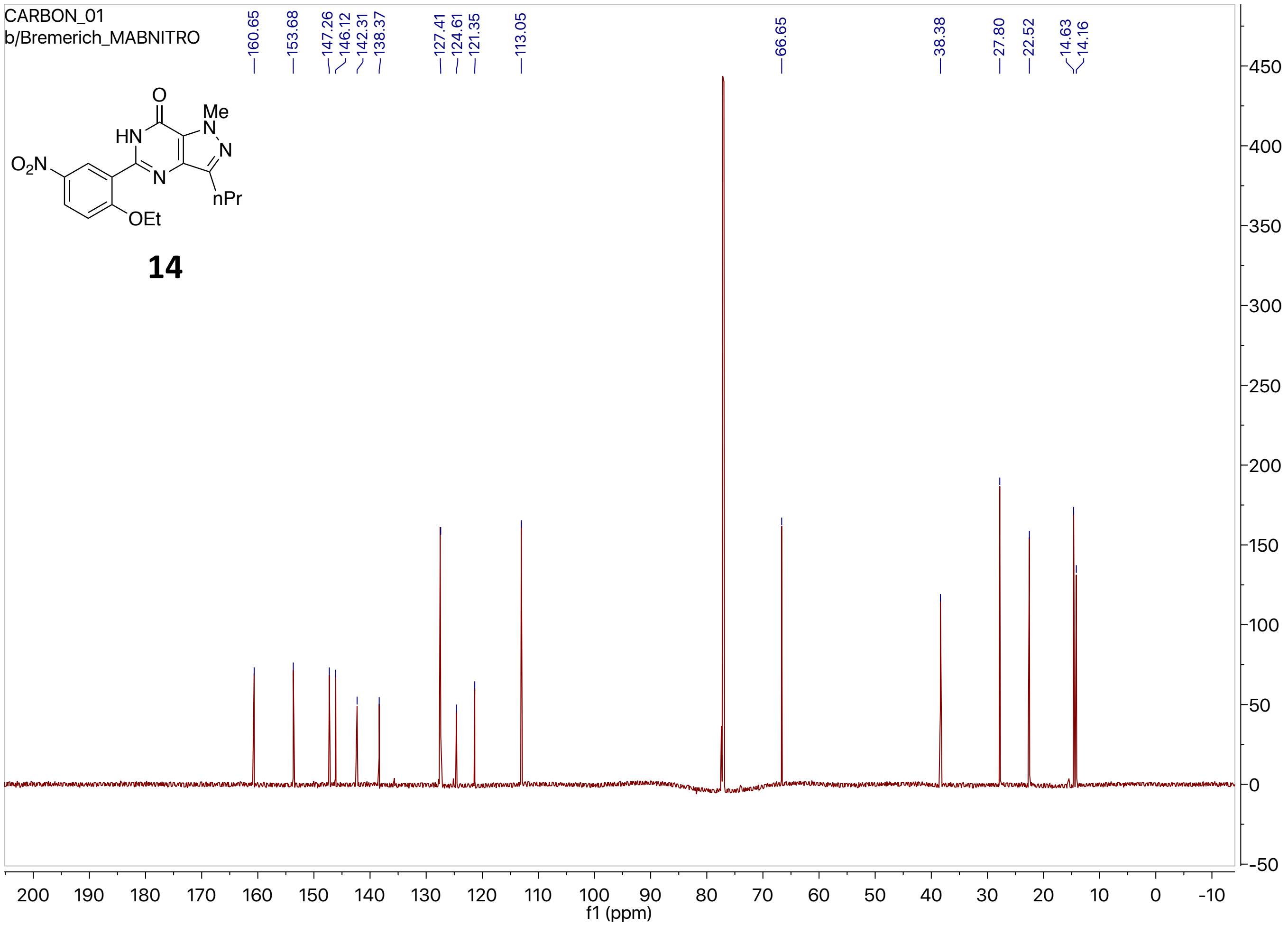
CARBON_01
b/Bremerich_MABNITRO
14
f1 (ppm)

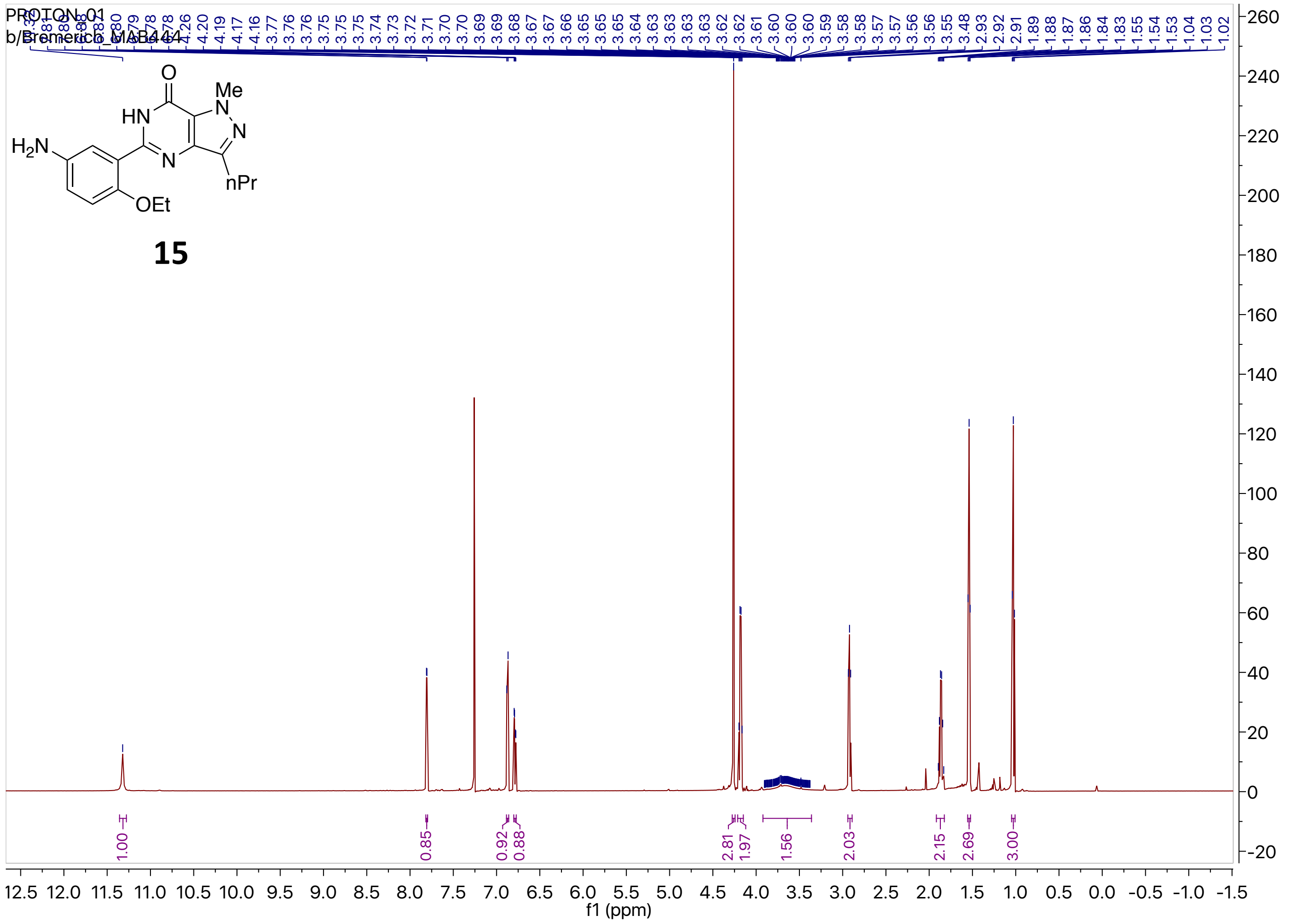
PROTON_01
b/Bremerich_MAB444
15
f1 (ppm)

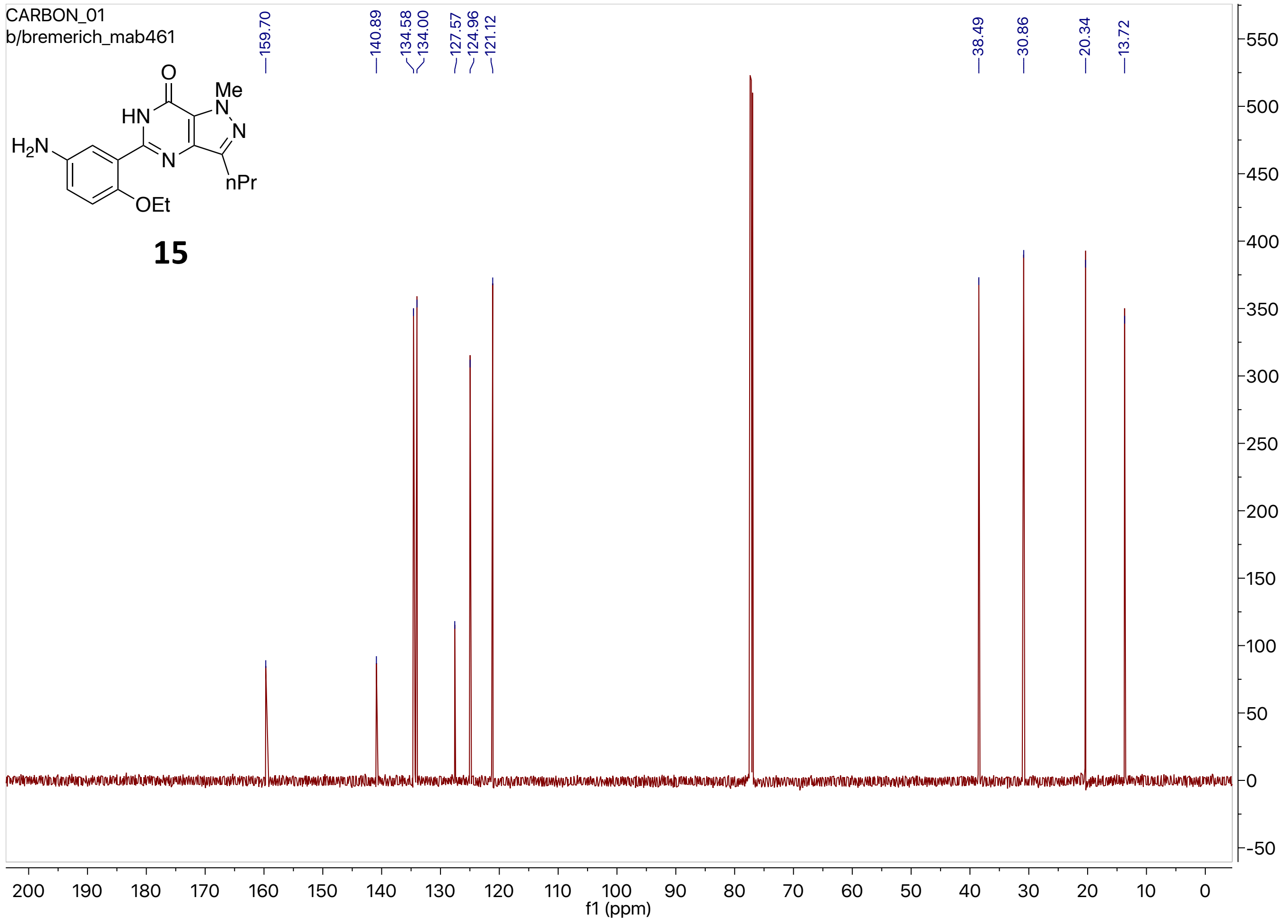
CARBON_01
b/bremerich_mab461
159.70
140.89
134.58
134.00
127.57
124.96
121.12
38.49
30.86
20.34
13.72
15
f1 (ppm)

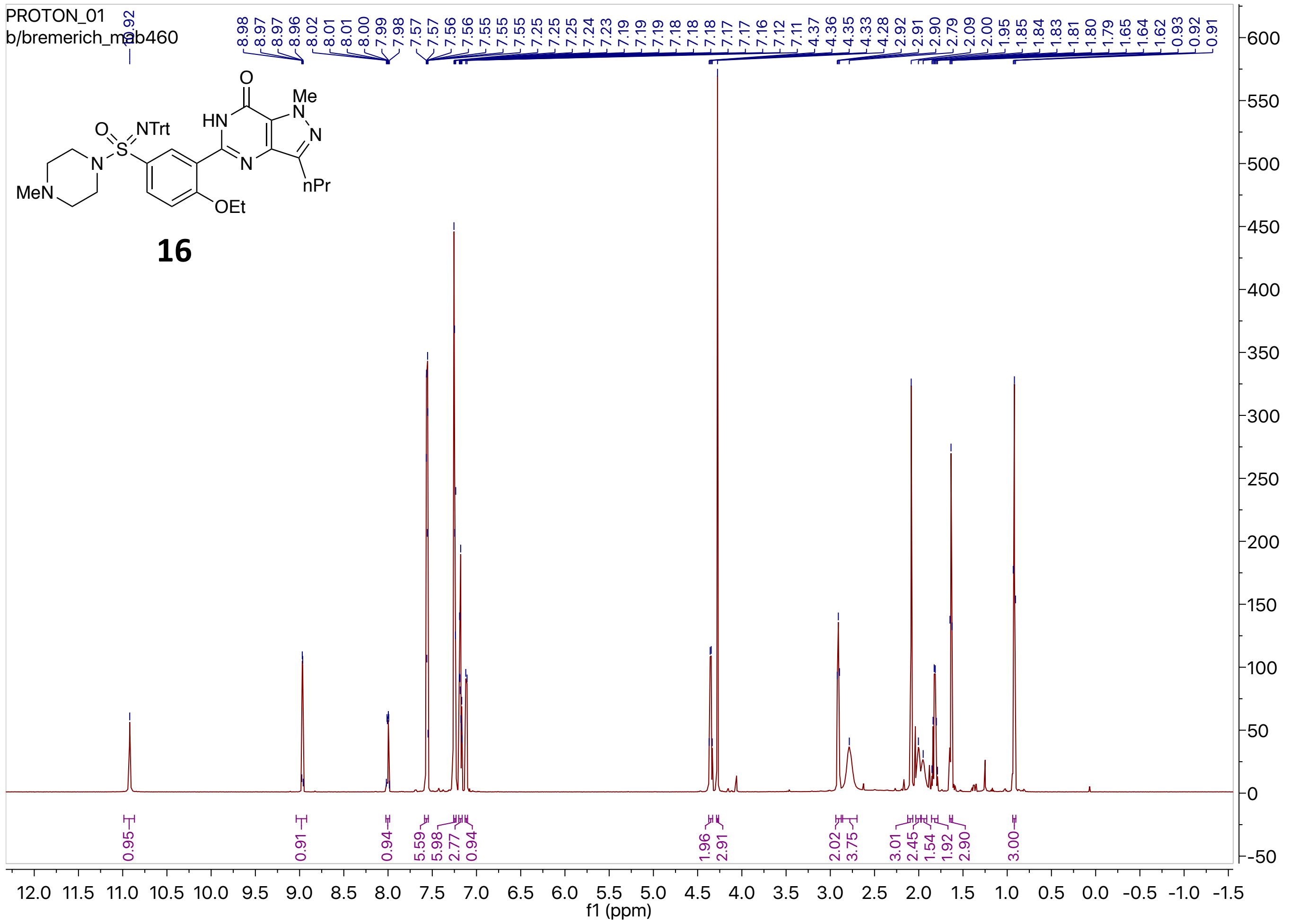
PROTON_01
b/bremerich_mab460
16
f1 (ppm)

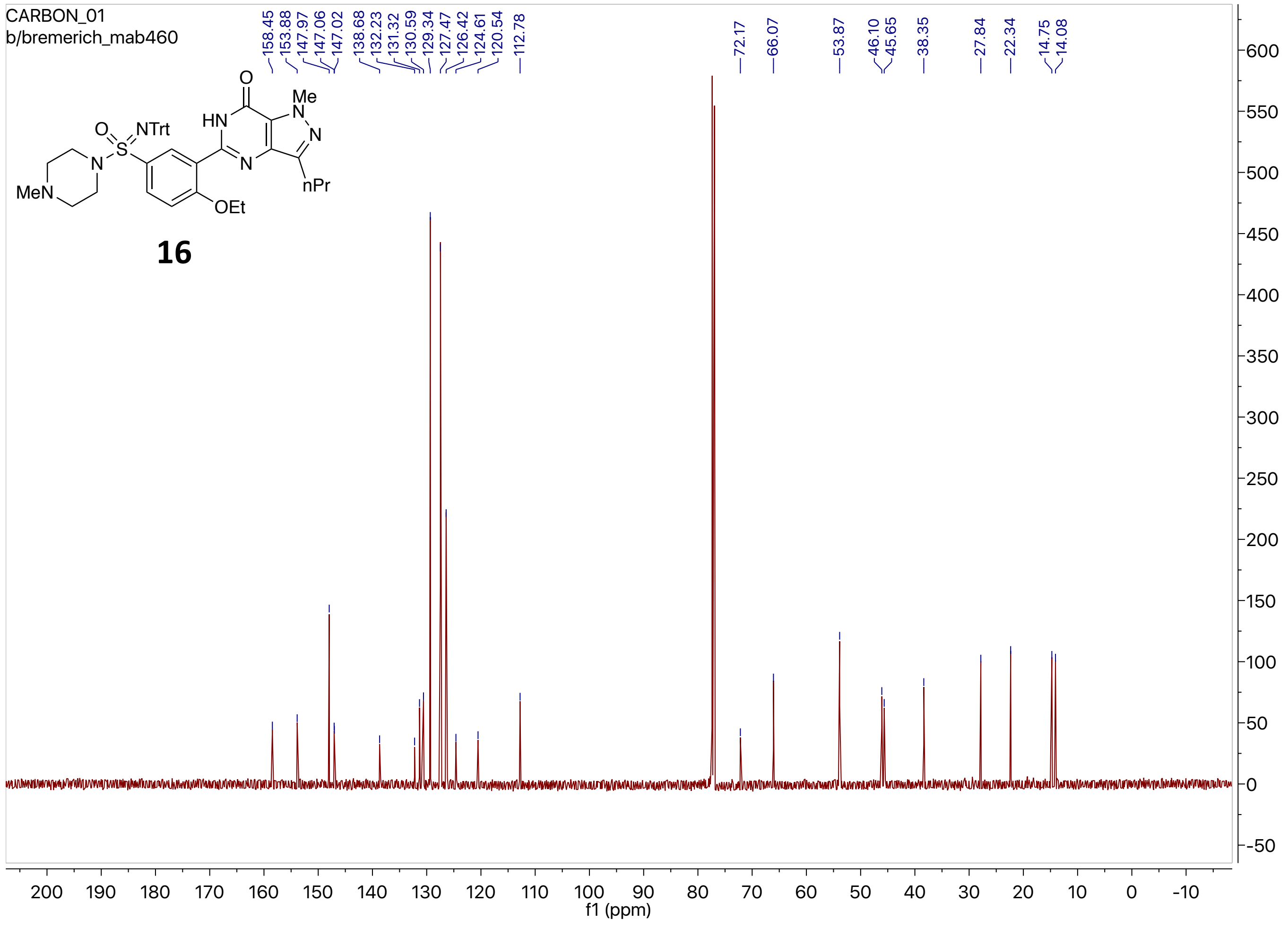
CARBON_01
b/bremerich_mab460
16
f1 (ppm)
